# HEPES in Cell Culture Alters the Multi‐Omics Profile Exhibited by Gaucher Disease Fibroblasts

**DOI:** 10.1002/jcb.70080

**Published:** 2026-01-16

**Authors:** Eleonore M. Corazolla, Bauke V. Schomakers, Maria M. Trętowicz, Jill Hermans, Michel van Weeghel, Frédéric M. Vaz, Mia L. Pras‐Raves, Karen Ghauharali‐van der Vlugt, Femke S. Beers‐Stet, Susanna M. I. Goorden, Judith Jansen‐Meijer, Georges E. Janssens, Carla E. M. Hollak, Riekelt H. Houtkooper, André B. P. van Kuilenburg

**Affiliations:** ^1^ Department of Endocrinology and Metabolism Amsterdam UMC Location University of Amsterdam Amsterdam North‐Holland Netherlands; ^2^ Laboratory Genetic Metabolic Diseases Amsterdam UMC Location University of Amsterdam Amsterdam North‐Holland Netherlands; ^3^ Amsterdam Gastroenterology Endocrinology and Metabolism Institute Amsterdam North‐Holland Netherlands; ^4^ Core Facility Metabolomics Amsterdam UMC Amsterdam North‐Holland Netherlands; ^5^ University Medical Center Utrecht Utrecht Netherlands; ^6^ Erasmus University Medical Center Rotterdam South‐Holland Netherlands; ^7^ Amsterdam Cardiovascular Sciences Institute Amsterdam North‐Holland Netherlands; ^8^ Department of Clinical Chemistry, Laboratory Genetic Metabolic Diseases Amsterdam UMC, Vrije Universiteit Amsterdam Amsterdam North‐Holland Netherlands

**Keywords:** cell culture techniques, Gaucher disease, HEPES, multi‐omics

## Abstract

Lysosomal function can be affected by components in cell culture. This in turn may influence cellular metabolism and, consequently, research and diagnostics outcomes. One such component is the commonly used pH buffer 4‐(2‐hydroxyethyl)‐1‐piperazineethanesulfonic acid (HEPES). HEPES specifically impacts the trafficking of the lysosomal enzyme glucocerebrosidase, which is deficient in Gaucher disease (GD). Understanding how HEPES affects cellular models of GD is essential, since glucocerebrosidase is central to diagnostic testing and the investigation of GD pathophysiology. Therefore, we examined the broader effects of HEPES on cultured fibroblasts from individuals with GD and healthy controls. We cultured dermal fibroblasts of eight adults with GD and seven healthy age‐ and sex‐matched controls. The cells were cultured in two culture media, Ham's F10 and DMEM, both with and without HEPES. We assessed glucocerebrosidase enzyme activity and sphingolipid concentrations using a quantitative UPLC‐MS/MS method. Additionally, we conducted multi‐omics analyses, consisting of lipidomics, metabolomics and proteomics, to explore the broader impact of HEPES in cell culture on fibroblasts. Glucocerebrosidase activity in cell lysates increased after HEPES exposure in both GD and control fibroblasts, to an extent that may influence diagnostic outcomes. In GD fibroblasts, substrate accumulation was absent and not altered by HEPES exposure. GD fibroblasts exhibited a multi‐omics profile largely overlapping with healthy controls and lacking the typical pathological features associated with GD in other cell types, such as mitochondrial dysfunction, dysregulated autophagy, disruption of intracellular calcium homeostasis, ER stress and chronic oxidative stress. In addition, the multi‐omics profile was altered by HEPES, however in a non‐specific manner. In conclusion, HEPES influences fibroblasts in culture, both from healthy controls and from patients with GD. Furthermore, GD fibroblasts lack a specific disease‐related profile. This renders cultured fibroblasts unsuitable for studying pathophysiological processes in GD. Culturing GD fibroblasts with HEPES may compromise the reliability of diagnostics.

**Abbreviations**
4‐MU: 4‐methylumbelliferone
BCA: bicinchoninic acid
CHO cells: Chinese hamster ovary cells
DMEM: Dulbecco's modified Eagle medium
DPBS: Dulbecco's Phophate Buffered Saline
EDTA: Ethylenediaminetetraacetic acid
GBA1: glucosylceramidase beta 1
GCase: glucocerebrosidase
GD: Gaucher disease
GlcCer: glucosylceramide
HEPES: 4‐(2‐hydroxyethyl)‐1‐piperazineethanesulfonic acid
iPSC: induced pluripotent stem cells
KNN: k‐nearest neighbors
Lyso‐GlcCer: glucosylsphingosine
PCA: Principal component analysis

## 1 Introduction

Lysosomal function is increasingly recognised as central to cellular metabolism [1]. As such, alterations in lysosomal function impact a wide variety of cellular processes [2]. These alterations occur in patients with lysosomal storage disorders and can also be introduced artificially by methods and components used in cell culture [3, 4, 5, 6, 7, 8]. The profound effects of cell culture components on lysosomal function can alter metabolic pathways and cellular activities, potentially influencing scientific findings [3, 5, 6, 8]. Therefore, it is essential to consider the impact of cell culture components on lysosomal function when designing experiments in metabolism research and related fields.

One commonly used cell culture agent that affects lysosomal metabolism is the chemical buffering agent 4‐(2‐hydroxyethyl)‐1‐piperazineethanesulfonic acid (HEPES) [8, 9, 10, 11, 12, 13, 14]. It is popular due to its advantageous properties in cell culture, particularly its maximum buffer capacity at physiological pH [15]. Four decades ago, cell culture with HEPES was first found to lead to false negative results in the diagnostic test at the time for the lysosomal disease Metachromatic leukodystrophy in fibroblasts, likely due to an increased lysosomal pH [9]. Since then, more effects of HEPES on cultured cells have been identified: unlike other pH buffers, HEPES promotes lysosomal biogenesis in various cultured cells, leading to lysosomal expansion and reduced lysosomal Ca^2+^ in CHO cells and iPSC‐derived neurons [13, 14]. HEPES also inhibits the transport of endocytic content into lysosomes and generates hydrogen peroxide under visible light exposure when combined with riboflavin in culture medium [10, 12], resulting in oxidative and lysosomal stress in oocytes during (sham) in vitro fertilisation [11].

In fibroblasts, HEPES affects a specific lysosomal enzyme: glucocerebrosidase (GCase; EC3.2.1.45). Culturing fibroblasts with HEPES impacts the maturation of GCase and thereby its trafficking to the lysosome by altering the enzyme's glycosylation. This leads to extra‐lysosomal accumulation, and therefore increased enzyme activity of GCase in lysates, while its lysosomal activity is reduced [13]. This occurs both in fibroblasts from healthy controls and from patients with Gaucher disease, in which GCase activity is already reduced due to a deleterious mutation in *GBA1* [13].

Gaucher disease (OMIM 230800; GD) is one of the most prevalent yet nonetheless rare lysosomal storage diseases [16]. GD is caused by pathogenic variants in the *GBA1* gene, leading to reduced function of GCase, a lysosomal enzyme responsible for degrading the sphingolipid glucosylceramide (GlcCer) [17]. Patients with GD accumulate GlcCer in tissue macrophages, resulting in a clinical phenotype of hematological, visceral, skeletal and neurological symptoms, the latter of which are more extensive in the less common primarily neuropathic form of GD (type 3 GD or GD3; OMIM 231000) [18, 19]. The diagnostic gold standard for GD involves the detection of decreased GCase activity in isolated leukocytes or cultured fibroblasts, combined with mutation analysis [20]. Accumulation of the substrate GlcCer or its derivative glycosylsphingosine (GlcSph) is very variable in cell lysates, and likely does not accurately reflect lysosomal accumulation, therefore it is not used as a diagnostic readout in cell lysates [21, 22, 23, 24, 25]. Cultured fibroblasts are often used as model systems for research on GD, although this cell type does not contribute to clinical features of GD in patients. Thus, the effect of HEPES on GCase in cultured fibroblasts is relevant for GD diagnostics and research, yet the most recent laboratory diagnostic guidelines do not specify whether fibroblasts should be cultured with or without HEPES [20].

In this study, we present the first report of the multi‐omic profile of cultured GD fibroblasts. Additionally, we examine the effect of HEPES on cultured fibroblasts from healthy controls and GD patients after culture in two commonly used culture media.

## 2 Materials and Methods

### 2.1 Fibroblast Samples and Ethics

Fibroblasts had previously been derived from skin biopsies from eight adult GD patients and seven healthy volunteers and stored in the Laboratory Genetic Metabolic Diseases biobank. The GD patients were recruited via the outpatient clinic of the Amsterdam UMC, the Dutch center of expertise for Gaucher disease. All patients had received a definite diagnosis based on enzymatic studies, genotyping or both. Healthy volunteers were recruited at the Amsterdam UMC, employees and students who had no diagnosis of an inborn error of metabolism could volunteer to donate fibroblasts. The control samples used in this study were selected to match at least one GD sample regarding age (with a maximum of 7 years age difference) and sex.

All skin biopsy donors provided written informed consent for the use of de‐identified skin‐derived fibroblasts for research purposes as part of the standard donation protocol for the Laboratory Genetic Metabolic Diseases biobank. Clinical data included in this study was obtained from the Amsterdam UMC clinical database on GD, in which the biobank samples can be linked to de‐identified clinical data, for which all GD donors also provided written informed consent. The use of fibroblast samples from the biobank for research purposes and the GD clinical database were approved by the biobank ethics committee of the Amsterdam UMC (BTC number 2014_192). The study was conducted in accordance with the Declaration of Helsinki.

### 2.2 Cell Culture

Fibroblasts were maintained in either DMEM (Gibco, cat. no. 41966) or Ham's F‐10 medium (Gibco, cat. no. 31550) supplemented with 10% fetal calf serum (Capricorn Scientific, lot nr. CP19‐2733) and 100 U/mL penicillin/100 µg/mL streptomycin (Gibco, cat. No. 15140) in a humidified atmosphere of 5% CO_2_ at 37°C. Where indicated, HEPES (VWR cat. no. J848) was added to the cell culture media in a 25 mmol/L concentration. Medium was refreshed every 48 h, cells were harvested by trypsinisation (0.05% trypsin‐EDTA solution in DPBS with Phenol Red, Thermo Fischer cat. no. 25300062) and passed every four to five days. Before each experiment, fibroblasts were passed in total four to six times on the respective medium, with total passage numbers ranging from 12 to 22 at final harvest (mean passage number 16 in GD (range 12‐21); mean passage number 17 in controls (range 13‐22); identical passage numbers between cells cultured with or without HEPES since they were cultured in parallel). Before the final harvest, fibroblasts were grown to 100% confluency and left in this state for a total of seven days without changing the culture medium. Unless stated otherwise, fibroblasts were harvested by trypsinisation, resuspended in their respective culture medium and centrifuged. The supernatant was discarded and cell pellets stored at ‐80°C until use as described below.

### 2.3 Glucocerebrosidase Enzyme Activity Assay

GCase activity was measured as described previously [26, 27, 28]. In short, cell pellets of four control samples and four GD samples were analysed using the 4‐methylumbelliferone (4‐MU) β‐d‐glucosidase activity assay. Cell pellets were homogenised in 200 μL ultrapure water (MilliQ purified) and lysed by sonification. β‐d‐glucosidase activity and protein concentration were determined in the crude extracts. For the enzyme reaction, 100 μL of 4MU‐β‐d‐glucopyranoside (5 mmol/L in 0.1 mol/L citrate/0.2 mol/L phosphate 15 g/L taurocholate (pH 5.5)) and 50 μL cell lysate (total 40 µg of protein/reaction) were combined. The reactions were incubated for 1 h at 37°C and terminated by adding 2850 μL of 0.2 M carbonate/glycine (pH 10.5) buffer solution. The fluorescence (excitation filter 360 nm and emission filter 450 nm) of the 4‐MU was measured on a fluorescence spectrometer (LS45, Perkin–Elmer). All measurements were corrected for background fluorescence, and the formed product was quantified using a 4‐MU standard curve. Enzyme activity was calculated with the software BLstudio using the following equation:

$$\frac{n_{\mathrm{sample}}-n_{\mathrm{blank}}}{m_{\mathrm{protein}}*t_{\mathrm{incubation}}}*60=enzyme\;activity\;\left[\frac{\mathrm{nmol}}{\mathrm{mg}*h}\right]$$

Protein concentration was quantified using the bicinchoninic acid (BCA) assay following the kit protocol and all GCase activity values were corrected for protein concentration (Thermo Fisher Scientific; cat. nr. 23225; absorbance measurements on Tecan Infinite M200 Microplate reader).

### 2.4 Sphingolipid Quantification

A cell suspension was prepared on ice by washing fibroblasts twice in sterile 9 g/L NaCl, and harvesting in 300 µL ultrapure water (MilliQ purified) by cell scraping. The cell suspension was sonicated in a 1.5 mL tube and 150 µL of the cell lysate was transferred to a fresh 2 mL tube and stored at –20°C until further sample preparation. For the quantification of sphingolipids, samples were prepared utilizing a chloroform and methanol extraction and measured using UPLC‐MS/MS (Xevo TQ‐XS by Waters with UPLC system by Waters) following the protocol as previously described [29]. For the quantification of lyso‐sphingolipids, samples were prepared according to the previously published protocol [30] with some adjustments since the matrix was cell lysate instead of plasma: the standard curves were generated in the presence of 50 µL pooled plasma and an extra washing step was added using 700 µL of water and 700 µL butanol to remove additional salts and other components from cell culture. Also, volumes of methanol (600 µL), chloroform (600 µL) and ammoniumformate‐buffer (450 µL) were increased to match the cell lysate volume (150 µL). Standard curve samples and the cell lysate samples were resuspended in 50 µL methanol and 50uL of ultrapure water (MilliQ purified) prior to UPLC‐MS/MS analysis. Protein quantification was performed as described above using the BCA assay.

### 2.5 Lipidomics

Lipidomics was performed as previously described, with minor adjustments [31]. Briefly, in a 2 mL tube, the following amounts of internal standards dissolved in 1:1 (vol/vol) methanol:chloroform were added to each fibroblast cell pellet: Bis(monoacylglycero)phosphate BMP (14:0) 2 (0.2 nmol), Ceramide‐1‐phosphate C1P (d18:1/12:0) (0.125 nmol), D_7_‐Cholesteryl Ester CE (16:0) (2.5 nmol), Ceramide Cer (d18:1/12:0) (0.125 nmol), Ceramide Cer (d18:1/25:0) (0.125 nmol), Cardiolipin CL (14:0)4 (0.1 nmol), Diacylglycerol DAG (14:0) 2 (0.5 nmol), Glucose Ceramide GlcCer (d18:1/12:0) (0.125 nmol), Lactose Ceramide LacCer (d18:1/12:0) (0.125 nmol), Lysophosphatidicacid LPA (14:0) (0.1 nmol), Lysophosphatidylcholine LPC (14:0) (0.5 nmol), Lysophosphatidylethanolamine LPE (14:0) (0.1 nmol), Lysophosphatidylglycerol LPG (14:0) (0.02 nmol), Phosphatidic acid PA (14:0) 2 (0.5 nmol), Phosphatidylcholine PC (14:0) 2 (2 nmol), Phosphatidylethanolamine PE (14:0) 2 (0.5 nmol), Phosphatidylglycerol PG (14:0) 2 (0.1 nmol), Phosphatidylinositol PI (8:0) 2 (0.5 nmol), Phosphatidylserine PS (14:0)2 (5 nmol), Sphinganine 1‐phosphate S1P (d17:0) (0.125 nmol), Sphinganine‐1‐phosphate S1P (d17:1) (0.125 nmol), Ceramide phosphocholines SM (d18:1/12:0) (2.125 nmol), Sphingosine SPH (d17:0) (0.125 nmol), Sphingosine SPH (d17:1) (0.125 nmol), Triacylglycerol TAG (14:0)2 (0.5 nmol). 1.5 mL 1:1 (vol/vol) methanol:chloroform was added and before thorough mixing. Samples were centrifuged for 10 min at 20.000*g* and the supernatant was transferred to a new 1.5 mL tube and evaporated under a stream of nitrogen at 30°C. The residue was dissolved in 100 μL of 1:1 (vol/vol) methanol:chloroform. Lipids were analysed using a Thermo Scientific Ultimate 3000 binary HPLC coupled to a Q Exactive Plus Orbitrap mass spectrometer. For normal phase separation, 2 μL of each sample was injected onto a Phenomenex® LUNA silica, 250 * 2 mm, 5 µm 100 Å. The column temperature was held at 25°C. The mobile phase consisted of (A) 85:15 (vol/vol) methanol:water containing 0.0125% formic acid and 3.35 mmol/L ammonia and (B) 97:3 (vol/vol) chloroform:methanol containing 0.0125% formic acid. The LC gradient was as previously described [31]. For reversed phase separation, 5 μL of each sample was injected onto a Waters HSS T3 column (150 × 2.1 mm, 1.8 μm particle size). The column temperature was held at 60°C. The mobile phase consisted of (A) 4:6 (vol/vol) methanol:water and B 1:9 (vol/vol) methanol:isopropanol, both containing 0.1% formic acid and 10 mmol/L ammonia. Using a flow rate of 0.4 mL/min, the LC gradient consisted of: Dwell at 100% A at 0 min, ramp to 80% A at 1 min, ramp to 0% A at 16 min, dwell at 0% A for 16–20 min, ramp to 100% A at 20.1 min, dwell at 100% A for 20.1–21 min. MS data were acquired using negative and positive ionisation using continuous scanning over the range of m/z 150 to m/z 2000. Data were analysed using an in‐house developed lipidomics pipeline written in the R Statistical Software (v4.4.1 [32]). Reported lipids were normalised to corresponding internal standards of the respective lipid class. Lipid identification was based on a combination of accurate mass, (relative) retention times, analysis of samples with known metabolic defects, and the injection of relevant standards. Protein quantification was performed on the dried protein pellet as described above using the BCA assay.

### 2.6 Metabolomics

Metabolomics was performed as previously described [33]. In short, the following internal standards dissolved in water were added to a 2 mL tube containing fibroblast cell pellets: Adenosine‐15N5‐monophosphate (5 nmol), Adenosine‐15N5‐triphosphate (5 nmol), Alanine‐13C3,15 N (5 nmol), Arginine‐13C6 (5 nmol), Aspartic acid‐13C4 (5 nmol), D3‐Carnitine (0.5 nmol), D4‐Citric acid (0.5 nmol), 13C1‐Citrulline (0.5 nmol), Cystine‐13C6,15 N2 (2.5 nmol), 13C6‐Fructose‐1,6‐diphosphate (1 nmol), Glutamic acid‐13C5 (5 nmol), D5‐Glutamine (0.5 nmol), Glycine‐13C2,15 N (5 nmol), Guanosine‐15N5‐monophosphate (5 nmol), Guanosine‐15N5‐triphosphate (5 nmol), 13C6‐Glucose (10 nmol), 13C6‐Glucose‐6‐phosphate (1 nmol), Histidine‐13C6 (5 nmol), Isoleucine‐13C6, 15N (5 nmol), D3‐Lactic acid (1 nmol), Leucine‐13C6,15N (5 nmol), Lysine13C6 (5 nmol), Methionine13C5,15N (5 nmol), D6‐Ornithine (0.5 nmol), Phenyl‐13C6‐alanine (5 nmol), Proline‐13C5 (5 nmol), 13C3‐Pyruvate (0.5 nmol), Serine‐13C3,15N (5 nmol), D6‐Succinic acid (0.5 nmol), Threonine‐13C4 (5 nmol), D4‐Thymine (1 nmol), Tyrosine‐(phenyl‐13C6) (5 nmol), D5‐Tryptophan (0.5 nmol), Valine‐13C5 (5 nmol). After phase separation with methanol/chloroform, the top layer was extracted and dried. After reconstitution in methanol/water, metabolites were analysed using a Waters Acquity ultra‐high performance liquid chromatography system coupled to a Bruker Impact II Ultra‐High Resolution Qq‐Time‐Of‐Flight mass spectrometer. Chromatographic separation was achieved using a Merck Millipore SeQuant ZIC‐cHILIC column (PEEK 100 × 2.1 mm, 3 µm particle size). Data were analysed using Bruker TASQ software version 2.1.22.3. All reported metabolite intensities were normalised to protein concentration, as well as to internal standards with comparable retention times and response in the MS. Metabolite identification has been based on a combination of accurate mass, (relative) retention times, ion mobility data and fragmentation spectra, compared to the analysis of a library of standards. Protein quantification was performed on the dried protein pellet as described above using the BCA assay.

### 2.7 Proteomics

Proteomics samples were prepared according to the protocol of the Thermo Scientific Easy Prep MS Sample Prep Kit (cat. no. A40006). Protein quantification was performed as described above using the BCA assay. For the analysis, 10 µL of sample was injected at 12°C, and pooled samples were injected as the first and last samples as well as at random intervals in between varying intervals throughout the series for quality control. Chromatographic separation was achieved on a Waters Acquity UPLC, using a Waters Acquity UPLC BEH C18 Column (130 Å, 1.7 µm, 2.1 mm X 50 mm; 186002350), equipped with a Waters Acquity UPLC Vanguard BEH C18 precolumn (186003975). Column temperature was held at 60°C. Mobile phase consisted of (A) water and (B) acetonitrile, both containing 0.1% formic acid. Using a starting flow rate of 0.5 ml/min, the LC gradient consisted of: Dwell at 3% B for 0–0.1 min; ramp to 40% B at 4.3 min; ramp to 80% B at 4.31 min with a flow rate of 0.85 ml/min; dwell at 80% B for 4.31–4.40 min with a flow rate of 0.85 mL/min; ramp to 3% B at 4.50 min with a flow rate of 0.6 mL/min; dwell at 3% B for 4.5–5 min with a flow rate of 0.5 mL/min. MS data were acquired with a Bruker timsTOF Pro 2 using positive ionization in DIA‐PASEF mode as previously reported [34]. DIA‐PASEF data files were processed using DIA‐NN version 1.8.1 [35], using a spectral library provided by Bruker. Data were normalised in DIA‐NN using MaxLFQ [36].

### 2.8 Data Availability, Visualisation and Statistical Analyses

Data analysis was performed using R Statistical Software (v4.5.0 [32]) in RStudio version 2024.12.1 using the “dplyr” package version 1.1.4 [37]. In data pre‐processing, missing data from lipidomics, metabolomics and proteomics were imputed using the k‐nearest neighbors (KNN) method with 10% as a threshold and the “multiUS” package in R [38].

A two‐tailed paired t‐test was used for statistical comparison of the effect of HEPES within groups (control or GD fibroblasts cultured on one culture medium), otherwise two‐tailed unpaired t‐tests were applied. *p*‐values were adjusted for multiple testing where indicated using the Bonferroni calculation. An adjusted *p*‐value of < 0.05 was considered statistically significant.

The principal component analysis (PCA) was performed using the R packages “FactoMineR” [39] and “factoextra” [40]. The package “missMDA” [41] was used to impute missing values for the PCA. Volcano plots were visualised using the R package “EnhancedVolcano” [42]. GO‐Term enrichment analysis on the proteomics data was performed using the DAVID Functional Annotation Tool [43] (https://davidbioinformatics.nih.gov/). For the metabolomics pathway analysis, metabolites were clustered into pathways based on literature and expert opinion (Supporting Information Table S1). Visualization of the pathway analysis was supported by the R packages “ggridges” [44] and “forcats” [45]. For the analysis of the lipidomics data per lipid species the mean of all measurements of lipids belonging to the respective lipid species for a group of samples (i.e. control or GD samples cultured on one culture medium) was calculated and the log2 fold change between control and GD samples depicted. Statistics were performed on the absolute measurements before log2 transformation.

**Table 1:** Characteristics of fibroblast donors.

		Control	GD	
Total participants		7 [100%]	8 [100%]	
Sex	Female	3 [43%]	5 [63%]	
	Male	4 [57%]	3 [37%]	
Age at sampling	Mean	31 years	35 years	
	Minimum	19 years	19 years	
	Maximum	55 years	62 years	
	18–29 years	5 [71%]	2 [25%]	
	30–39 years	0 [0%]	4 [30%]	
	40–49 years	0 [0%]	1 [13%]	
	50–59 years	2 [29%]	0 [0%]	
	60–69 years	0 [0%]	1 [13%]	
Mutation		n/a	N370S/other	3 [38%]
			L444P/other	2 [25%]
			N118S/N118S	2 [25%]
			L444P/L444P	1 [12%]
Clinical manifestations		n/a	Asymptomatic	2 [25%]
			Asymptomatic under ERT/SRT	2 [25%]
			Splenectomy	1 [12%]
			Hematologic involvement	1 [12%]
			Fatigue	2 [25%]
			CNS involvement	1 [12%]

Abbreviations: CNS, central nervous system; ERT, enzyme replacement therapy; GD, Gaucher disease; SRT, Substrate reduction therapy.

Data visualization was generally supported by the R package “ggplot” [46], Microsoft PowerPoint version 2504 and Adobe Illustrator version 29.3. Data in figures are presented as mean per individual donor unless stated otherwise.

## 3 Results

### 3.1 Participants

Fibroblasts of eight people with GD and seven healthy controls were included. The percentage of women and the mean age were higher and the age range larger in the GD group than in the control group (Table 1). The clinical manifestations in the GD group ranged from asymptomatic to severe neuronopathic GD (GD3) with an L444P/L444P mutation (Table 1).

### 3.2 GCase Activity is Higher in Lysates of Fibroblasts When Cultured With HEPES

**Figure 1:**
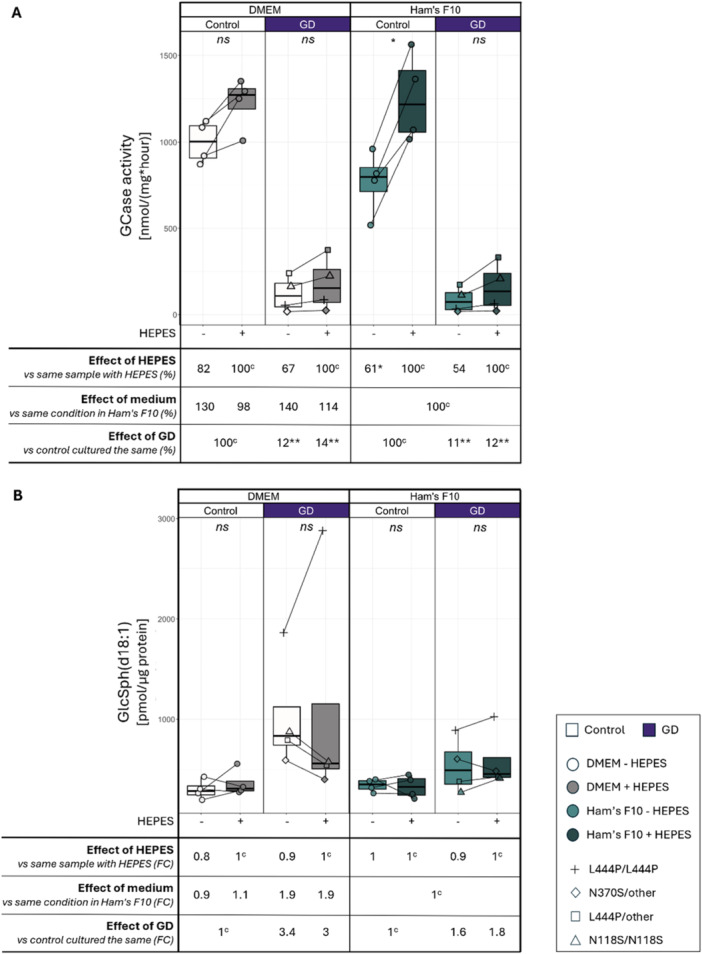
Effect of HEPES and culture medium on GCase activity and substrate accumulation. (A) GCase activity in different culture conditions. Each dot represents the GCase activity in the sample of one fibroblast donor (*n* = 4 each for controls and GD). Lines connect results of the same fibroblast donor. *p*‐values were calculated using a paired t‐test and adjusted according to Bonferroni, the level of significance is denoted by an asterisk or “ns” (not significant). In the comparisons at the bottom of the figure the mean GCase activity is compared between conditions. Superscript “c” denotes the samples that were set to 100% as a comparator. In the comparison investigating the effect of HEPES, each sample that was cultured without HEPES was compared to the identical sample cultured with HEPES (e.g. GD in DMEM without HEPES compared to GD in DMEM with HEPES). For the investigation of the effect of the culture medium, each sample cultured in DMEM was compared to the identical sample cultured in Ham's F10 (e.g. GD in DMEM without HEPES compared to GD in Ham's F10 without HEPES). For the investigation of the effect of GD, each GD sample was compared to the control sample that was cultured in identical conditions (e.g. GD in DMEM without HEPES compared to control in DMEM without HEPES). (B) Substrate accumulation in different culture conditions. Each dot represents the mean abundance of GlcSph (or lyso‐GlcCer) in the sample of one fibroblast donor (*n* = 4 each for controls and GD), lines connect results of the same donor. The comparisons at the bottom of the figure are the same as in Figure 1A and compare the fold change of the mean abundance of GlcSph in different culture conditions. *p*‐values were calculated using a paired t‐test for all analyses except the effect of GD which was calculated using an unpaired t‐test, and all *p*‐values were adjusted according to Bonferroni. DMEM, Dulbecco's modified Eagle medium; GCase, glucocerebrosidase; GD, Gaucher disease; HEPES, 4‐(2‐hydroxyethyl)‐1‐piperazineethanesulfonic acid; Lyso‐GlcSph, glucosylsphingosine.

To investigate the effect of HEPES and different culture media on GCase activity we quantified GCase activity in four culture conditions, namely one week of culture on Ham's F10 medium or DMEM with or without the addition of HEPES (Supporting Information Table 2). After culture with HEPES, GCase activity increased in all samples and in both culture media compared to culturing without HEPES (range of average 54%–82%; Figure 1A). This is consistent with previous literature [13]. This increase was statistically significant in control fibroblasts cultured on Ham's F10 medium. GCase activity appeared higher on average when cells were cultured in DMEM than after culture in Ham's F10 medium, especially in the absence of HEPES, however this did not reach statistical significance (Figure 1A). In both culture media, GD fibroblasts retained relatively less GCase activity when cultured without HEPES than control fibroblasts (GD fibroblasts retained 54% activity without HEPES in Ham's F10 compared to 61% in controls; in DMEM this was 67% and 82% respectively; Figure 1A). The GCase activity in GD compared to controls ranged from 11% in Ham's F10 without HEPES to 14% in DMEM with HEPES (Figure 1A).

To elucidate how much variation culture conditions could induce in a hypothetical diagnostic setting, we analysed the variation of GCase activity relative to the average of all controls in the same culture condition for each individual GD donor. Within the samples of one individual donor, the different culture methods led to a variation of GCase activity relative to controls in the same culture condition of on average 3.4% (0.8%–7.8%; Supporting Information Table S3). In one GD sample, the recommended diagnostic threshold of < 15% GCase activity was only fulfilled in one culture condition, namely Ham's F10 without HEPES (14.8%). In all other culture conditions higher relative GCase activity was measured (16.5%–18.4%; Supporting Information Table S3). In the other samples, the same diagnostic conclusion would have been reached in all culture conditions (Supporting Information Table S3).

In summary, the effect of HEPES on GCase activity was universal but not identical across samples and culture media. We demonstrated that HEPES tended to increase GCase activity in all samples, however in most conditions this did not reach statistical significance in this small and heterogenous cohort. Our findings indicate that in a hypothetical diagnostic analysis, the variation in GCase activity introduced by culture with HEPES may alter the conclusion, however this needs more extensive investigation.

### 3.3 Substrate Accumulation is Absent and not Altered by HEPES Exposure

We next investigated whether an enhanced GCase activity after culture with HEPES is accompanied by reduced accumulation of the substrate of GCase. To this end we quantified glucosylceramide (GlcCer) and its deacylated derivative glucosylsphingosine (GlcSph, also referred to as lyso‐GlcCer) using the method routinely used for diagnostics.

GlcSph was increased in GD fibroblasts compared to controls, however this only reached statistical significance in Ham's F10 medium without HEPES (Figure 1B; Supporting Information Table S4). Culturing with HEPES‐buffered medium for one week did not significantly alter the concentration of GlcSph in control or GD samples (Figure 1B). Notably, one sample, the L444P/L444P GD3 sample, displayed higher substrate accumulation after HEPES exposure. The type of culture medium affected substrate accumulation in GD samples but not control samples (3‐ and 3.4‐fold change of GlcSph abundance in GD compared to controls in DMEM with and without HEPES respectively; in Ham's F10 this was 1.6‐ and 1.8‐fold change) (Figure 1B). However, this did not reach statistical significance.

In summary, substrate accumulation in GD fibroblasts was absent. The concentration of GlcSph did not follow the pattern of GCase activity in cell lysates, as we observed no significant difference in GlcSph concentration in the conditions in which GCase activity was increased.

### 3.4 Cell Culture Conditions do not Alter the Abundance of Other (Lyso‐)Sphingolipids

We next quantified all (lyso‐)sphingolipids included in the diagnostic sphingolipid quantification panel at our center (Supporting Information Table S4). As expected, the largest difference in sphingolipid concentration between GD and control fibroblasts was GlcSph, which is used as a biomarker of GD in plasma (Supporting Information Figure S1A). GlcSph in Ham's F10 without HEPES was the only statistically significant difference between GD and controls (Supporting Information Figure S1A). GlcCer concentration was only slightly higher in GD compared to control fibroblasts, as previously described [47] (Supporting Information Figure S1B). The highest concentration of GlcSph and GlcCer was consistently measured in the GD3 (L444P/L444P) sample. The concentration of the other (lyso‐)sphingolipids was not statistically significantly altered (Supporting Information Figure S1A–B).

### 3.5 Lipidomics, Metabolomics and Proteomics Data Demonstrate That the Multi‐Omic Profile in Gaucher Disease Fibroblasts Hardly Differs From Healthy Controls and is Altered by Culture With HEPES

Next, we employed a multi‐omics approach comprised of lipidomics, metabolomics and proteomics to study the effect of HEPES in Ham's F10 medium in control and GD fibroblasts.

**Figure 2:**
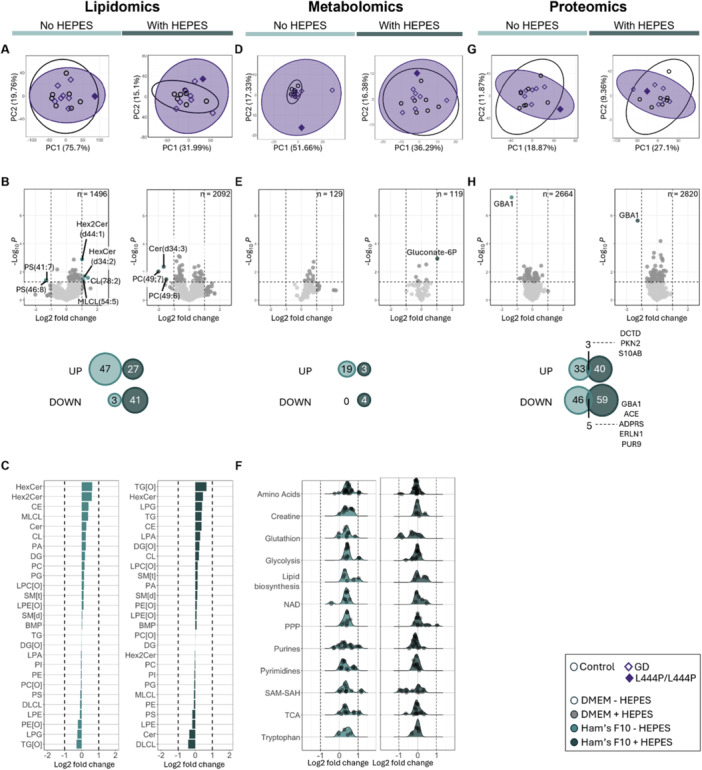
Multi‐omics analysis comparing GD fibroblasts cultured with and without HEPES regarding the lipidome (A–C), metabolome (D,E) and proteome (G,H). (A, D, G) Principal component analysis of lipidomic (A), metabolomic (D) and proteomic (G) profiles in GD compared to control fibroblasts in culture with and without HEPES. Each dot represents the average of all replicates of one fibroblast donor. PCA plots of the two culture conditions were not combined into a single graph because samples from each condition were run separately on the mass spectrometer, therefore GD versus control within a culture condition can be compared in absolute terms but between culture conditions only relative comparisons, as depicted, are valid. (B, E, H) Volcano plots and Venn diagrams depicting changed lipids (B), metabolites (E) and proteins (H) in GD compared to control fibroblasts in culture with and without HEPES. Each dot represents the log2 fold change and *p*‐value of the mean of all GD samples compared to the mean of all control samples. Analytes with a log2 FC > |1| and a *p*‐value < 0.05 are annotated. *p*‐values were not adjusted for multiple comparisons for visualization purposes. In the Venn diagrams the number of hits with an unadjusted *p*‐value < 0.05 are depicted for each culture condition split into “up” (log2 fold change > 0) or “down” (log2 fold change < 0) groups. The overlapping analytes are named. (C) Changes in lipid classes in GD compared to control fibroblasts in culture with and without HEPES. Each bar represents the mean log2 fold change of the combined lipids in this class for all GD samples compared to all control samples. No lipid class is significantly enriched (*p*‐values not depicted). (F) Ridge plot of the changes in (polar) metabolites depicted per pathway in GD compared to control fibroblasts in culture with and without HEPES. Each dot represents the average log2 fold change of a metabolite in GD compared to controls. Metabolites are grouped by the pathway with which they are associated (Supporting Information Table S1). The same metabolite may appear in multiple pathways. No pathway is statistically significantly enriched (*p*‐values not depicted). GD, Gaucher disease; HEPES, 4‐(2‐hydroxyethyl)‐1‐piperazineethanesulfonic acid; PC, principle component; PPP, pentose phosphate pathway; TCA, tricarboxylic acid cycle.

Using lipidomics we measured 2092 lipids in the condition with and without HEPES, respectively (Supporting Information Table S5). In both culture conditions, controls and GD samples were very similar, as is evident from the principal component analyses (PCAs) (Figure 2A). The abundance of only 3% of all lipids identified in both culture conditions was altered with an unadjusted *p*‐value < 0.05 comparing GD to controls and in only twelve of those lipids (0.3% of all identified lipids) the abundance was altered by more than twofold (Figure 2B). Interestingly, none of the lipids with altered abundance were similarly changed in both culture conditions, indicating distinct profiles depending on HEPES exposure (Figure 2B). The altered lipids of each profile were not enriched for any specific lipid class (Figure 2C).

The metabolomics analyses measured 129 unique metabolites in the condition with and without HEPES respectively (Supporting Information Table S6). Again, GD and control samples could not be differentiated by PCA (Figure 2D). The abundance of 3% and 17% of all identified metabolites were altered in the condition with and without HEPES respectively (Figure 2E). No metabolites with altered abundance in GD overlapped between the conditions with and without HEPES exposure (Figure 2E). When metabolites were clustered by associated metabolic pathways, no specific pathway was enriched for metabolites with altered abundance (Figure 2F).

Finally, we performed proteomics and measured 2820 unique proteins in the condition with and without HEPES respectively (Supporting Information Table S7). Similar to the lipidomics and metabolomics data, PCA could not differentiate between control and GD in either culture condition (Figure 2G). Only 3% of all identified proteins were differentially expressed in either culture conditions (Figure 2H). However, in contrast to the lipidomics and metabolomics data, the profile of differently expressed proteins in GD did have a limited overlap of three proteins that were upregulated and five that were downregulated in both conditions (Figure 2H). Of these proteins, GBA1, the protein catalyzing the GCase reaction was significantly less abundant in GD (Figure 2H). The other seven overlapping differentially expressed proteins could not be meaningfully linked to each other.

In conclusion, GD and control fibroblasts hardly differ in the lipidome, metabolome and proteome. The few observable differences were distinct depending on exposure to HEPES in cell culture, except for proteomics in which a limited number of individual differentially expressed proteins occurred in both culture conditions.

## 4 Discussion

In this study we demonstrate that HEPES increases GCase activity in fibroblast lysates cultured in Ham's F10 medium without significant changes to GlcSph concentration or alterations in other (lyso‐)sphingolipids. In addition, we demonstrate that GD fibroblasts exhibit a multi‐omics profile that largely overlaps with that of healthy controls and lacks the typical features associated with pathological processes of GD observed in other cell types. Notably, this profile is altered by HEPES exposure, however this alteration is not specific to a particular metabolic pathway, subcellular localization or class of metabolites.

As previously demonstrated using fluorescent activity‐based probes, cell culture with HEPES reduces GCase maturation, resulting in decreased lysosomal GCase activity but increased GCase activity in cell lysates, along with an accumulation of its substrate GlcSph [13]. In our study we conduct multi‐omics profiling to explore the effects of HEPES. Interestingly, we observe differences across all three employed omics methods; however, these differences do not cluster within a specific metabolic pathway, class or organelle leading to a diffuse profile of HEPES‐related changes. It cannot be excluded that the observed changes reflect an underlying functional relationship between the affected analytes that was not captured by our analyses—for example, a potential structural stabilization of certain analytes mediated by the pH‐buffering properties of HEPES. Changes in mechanisms such as cellular senescence, that may play a role specifically in cell culture with high confluence and passage numbers were not observed in our multi‐omics analysis. The pH‐buffering capacity of HEPES may have mitigated cellular stress encountered by fibroblasts during culture. However, to account for the observed multi‐omic differences between GD and control fibroblasts under HEPES‐supplemented versus non‐supplemented conditions, HEPES would need to exert differential effects on GD and control cells. At present, we have no evidence that HEPES affects control and GD fibroblasts differently.

A cellular phenotype of GD has been established in other cell types such as macrophage or neuronal cell models [48]. This phenotype includes mitochondrial dysfunction, dysregulated autophagy, disruption of intracellular calcium homeostasis, ER stress, chronic oxidative stress and low‐grade inflammation [48]. These findings were recently confirmed in an integrative multi‐omics analysis of plasma from patients with GD1 [49]. However, despite our comprehensive analyses, a similarly evident GD phenotype is not observed in fibroblasts, regardless of HEPES exposure. We therefore conclude that fibroblasts do not exhibit the expected disease‐related profile in multi‐omics. Other cell types more involved in GD pathophysiology may display a multi‐omic profile that enhances our understanding of the disease and its pathophysiology, therefore we recommend conducting further analyses in isolated or cultured macrophages and neuronal models. The mechanism leading to increased GlcSph accumulation after exposure to HEPES in the L444P/L444P fibroblast sample, in contrast to all other samples, remains unknown. A potential explanation could be that the L444P homozygous mutation leads to a protein that is more susceptible to the glycosylation changes induced by HEPES and therefore a strong decrease in lysosomal GlcCer activity. Further investigation into this mechanism may support a better understanding of the pathophysiological differences between GD with a L444P/L444P mutation and other mutations.

This study was limited with regards to sample size, and the GD donors were clinically heterogeneous. This complicates the interpretation of the substrate accumulation data, as only fibroblasts from the GD3 (L444P homozygous) patient showed increased substrate accumulation with HEPES exposure. Conversely, the homogeneity of the multi‐omics data (Supporting Information Figure S2) despite the small sample size and clinical heterogeneity supports our conclusion that fibroblasts are not a valid model for GD.

In summary, this study presents two main findings: Firstly, we confirm the effect of HEPES on GD fibroblasts, noting increased GCase activity in lysates, although the previously reported reduction in substrate accumulation was not observed. These findings raise significant concerns about whether this effect of HEPES is substantial enough to affect diagnostic outcomes in borderline cases of deficient GCase activity. This warrants extensive investigation, and depending on the results of that investigation international guideline authors should consider adding a recommendation regarding HEPES‐buffered medium in GD diagnostics. Secondly, independent of HEPES exposure a cellular phenotype of GD fibroblasts could not be detected using a broad panel of multi‐omics, and expected differences as described in literature could not be confirmed. However, the multi‐omic profile of GD varied depending on HEPES exposure.

## 5 Conclusion

Given this overall absence of expected findings, the main conclusion of this study is that cultured fibroblasts are unsuitable as models for research on the pathophysiological mechanisms of GD. Low GCase activity in GD fibroblasts means diagnostic enzyme assays that measure GCase activity are feasible, however since these are performed in cell lysates, research into the impact of cell culture with HEPES on diagnostic outcomes is necessary to prevent false‐negative results in borderline cases. More broadly, this study serves as a cautionary reminder for researchers in the field of metabolism to remain vigilant about the potential effects of commonly used cell culture components and methods on our findings.

## Author Contributions

**E. Corazolla:** conceptualization, methodology, formal analysis, investigation, writing – original draft, writing – review and editing, visualization, project administration, funding acquisition. **B. Schomakers:** methodology, formal analysis, investigation, data curation, writing – review and editing. **M. Tretowicz:** investigation, writing – review and editing. **J. Hermans:** investigation, writing – review and editing. **M. van Weeghel:** methodology, writing – review and editing. **F. Vaz:** methodology, writing – review and editing. **M. Pras‐Raves:** formal analysis, data curation, writing – review and editing. **K. Ghauharali‐van de Vlugt:** investigation, data curation, writing – review and editing. **F. Beers‐Stet:** investigation, data curation, writing – review and editing. **S. Goorden:** methodology, writing – review and editing. **J. Jansen‐Meijer:** investigation, writing – review and editing. **G. Janssens:** methodology, writing – review and editing. **C. Hollak:** conceptualization, methodology, writing – original draft, writing – review and editing, supervision, funding acquisition. **R. Houtkooper:** conceptualization, methodology, resources, writing – original draft, writing – review and editing, supervision, funding acquisition. **A. van Kuilenburg:** conceptualization, methodology, resources, writing – original draft, writing – review and editing, supervision, funding acquisition.

## Ethics Statement

The use of fibroblast samples from the biobank for research purposes and the GD clinical database were approved by the biobank ethics committee of the Amsterdam UMC (BTC number 2014_192). The study was conducted in accordance with the Declaration of Helsinki.

## Consent

All skin biopsy donors provided written informed consent for the use of de‐identified skin‐derived fibroblasts for research purposes as part of the standard donation protocol for the Laboratory Genetic Metabolic Diseases biobank. Clinical data included in this study was obtained from the Amsterdam UMC clinical database on GD, in which the biobank samples can be linked to de‐identified clinical data, for which all GD donors also provided written informed consent.

## Conflicts of Interest

C.H. is unpaid executive board member of RARE‐NL, a not‐for‐profit foundation supporting academic development and access of medicines for rare diseases and repurposing.

## Supporting information

supmat.

SupplTbl1_MetabolitesPerPathway_v2.

SupplTbl2_GCaseActivity_v2.

SupplTbl3_RelativeGCaseActivityPerPatient_v2.

SupplTbl4_SphingolipidConc_v2.

SupplTbl5_Lipidomics_v3.

SupplTbl6_Metabolomics_v3.

SupplTbl7_Proteomics_v3.

## Data Availability

The data that supports the findings of this study are available in the supporting material of this article.
