# Supplementary material for: HEPES in Cell Culture Alters the Multi‐Omics Profile Exhibited by Gaucher Disease Fibroblasts
Source: J Cell Biochem. 2026 Jan 16;127(1):e70080. doi: 10.1002/jcb.70080 (PMC12809196; doi:10.1002/jcb.70080)
Supplement: Supplementary file 1 — supmat. [file JCB-127-e70080-s006.docx]

**SUPPLEMENTAL MATERIALS**

**
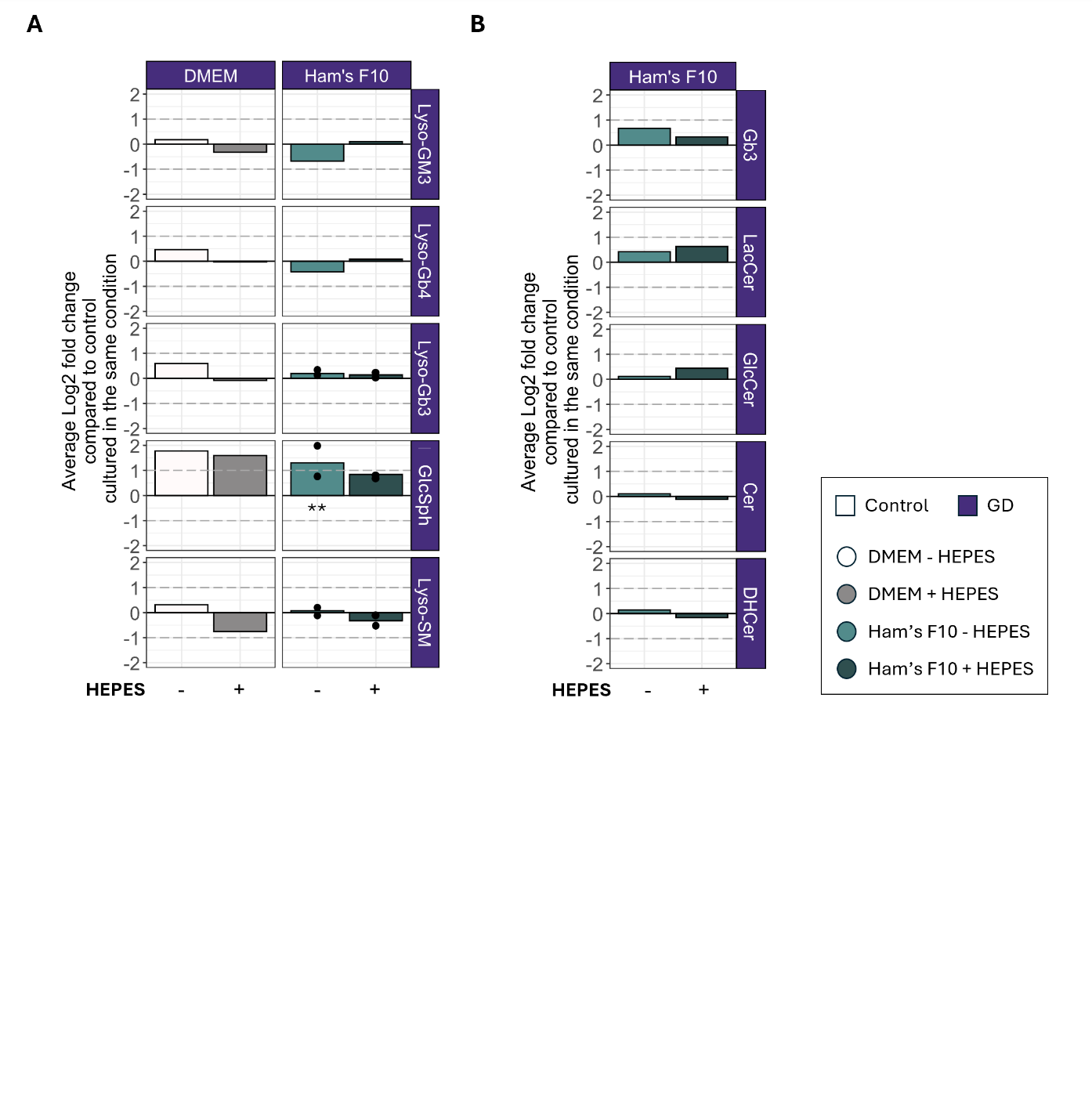
**

**Supplemental Figure 1:** **Effect of HEPES on (lyso-)sphingolipid accumulation. (A) Effect on lyso-sphingolipid abundance in GD fibroblasts compared to control fibroblasts cultured in the same conditions.** Bars represent the average log2 fold change of the respective lyso-sphingolipid of GD compared to control fibroblasts in the respective culture condition. Dots denote the averages of two separate experiments which were summarised by the bar graph where applicable. Adjusted p-values (Bonferroni) are depicted by asterisks only where statistically significant. **(B) Effect on sphingolipid abundance in GD fibroblasts compared to control fibroblasts cultured in the same conditions.** Adjusted p-values were not statistically significant (not depicted).

Abbreviations: Cer: ceramide; DHCer: dihydroceramide; DMEM: Dulbecco’s modified Eagle medium; Gb3: globotriaosylceramide, Gb4: globotetraosylceramides; GD: Gaucher disease; GlcCer: glucosylceramide; GM3: monosialodihexosylganglioside; HEPES: 4-(2-hydroxyethyl)-1-piperazineethanesulfonic acid; LacCer: lactosylceramide; Lyso-GlcCer: glucosylsphingosine; SM: sphingomyelin


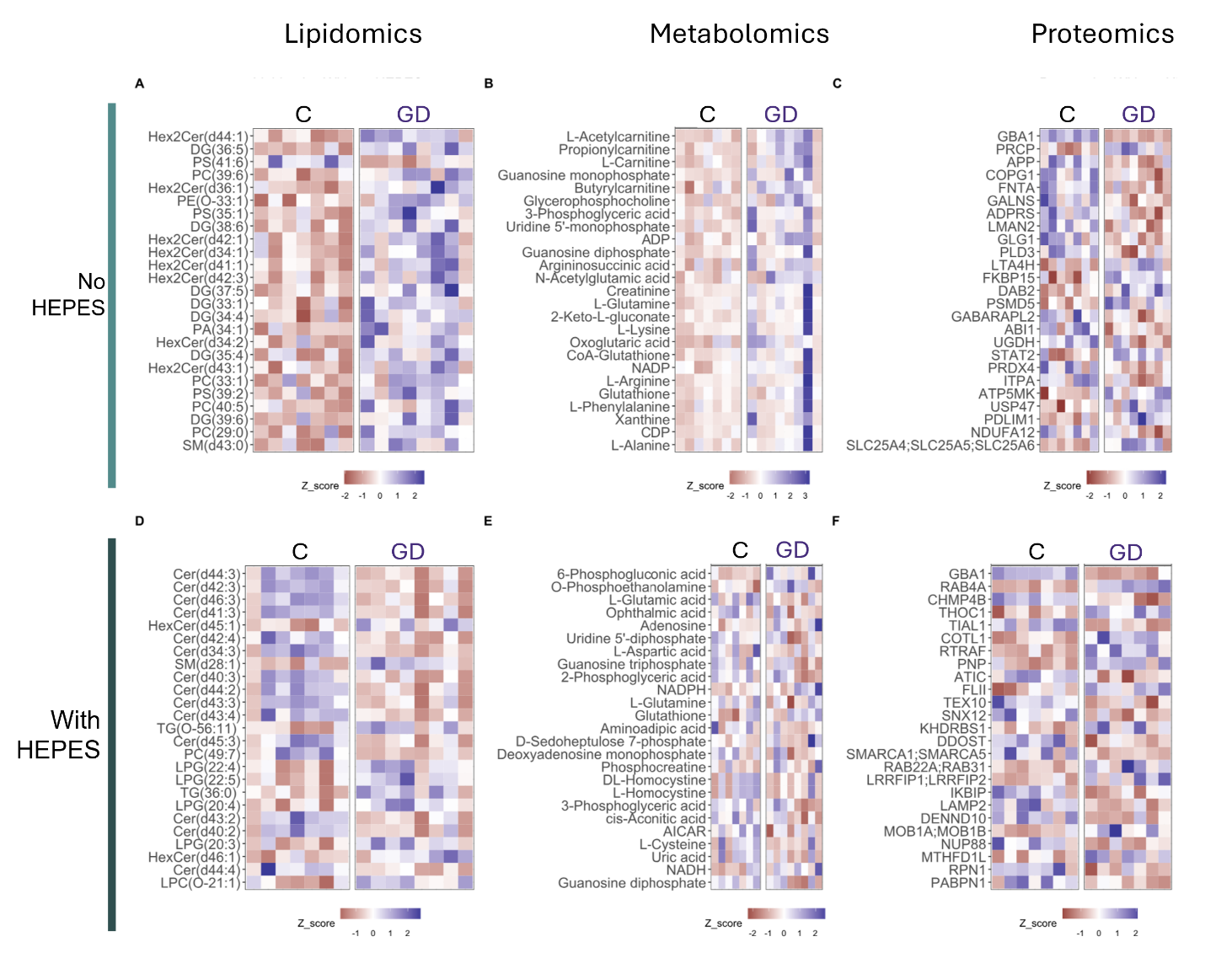


**Supplemental Figure 2:** **Heatmaps depicting z-scores of the top 25 (ranked by p-value) lipids (A,D), metabolites (B,E) and proteins (C,F) of GD and control fibroblasts cultured without HEPES (A-C) or with HEPES (D-F).**

Abbreviations: C: Control; GD: Gaucher disease; HEPES: 4-(2-hydroxyethyl)-1-piperazineethanesulfonic acid
