# Supplementary material for: HEPES in Cell Culture Alters the Multi‐Omics Profile Exhibited by Gaucher Disease Fibroblasts
Source: J Cell Biochem. 2026 Jan 16;127(1):e70080. doi: 10.1002/jcb.70080 (PMC12809196; doi:10.1002/jcb.70080)
Supplement: Supplementary file 2 — SupplTbl1_MetabolitesPerPathway_v2. [file JCB-127-e70080-s001.pdf]

Supplemental Table 1: Metabolites assigned to metabolic pathways

Analyte Name	Pathway
1,3-Diphosphoglyceric acid	Glycolysis
2-phosphoglyceric acid	Glycolysis
3-phosphoglyceric acid	Glycolysis
Fructose-1,6-diphosphate	Glycolysis
Glucose	Glycolysis
Glucose-6P	Glycolysis
Lactate	Glycolysis
Phosphoenolpyruvate	Glycolysis
Pyruvate	Glycolysis
Hexose-P	Glycolysis
Dihydroxyacetone-P	Glycolysis
Glyceraldehyde-3P	Glycolysis
Alanine	Glycolysis
Pyruvate	TCA
Acetyl-CoA	TCA
Alpha-Ketoglutarate	TCA
Citric acid	TCA
Fumarate	TCA
Glutamate	TCA
Glutamine	TCA
Malate	TCA
Oxaloacetate	TCA
Alanine	TCA
Lactate	TCA
Glucose	TCA
Succinate	TCA
Coenzyme_A	TCA
FAD	TCA
Adenine	Purines
Adenosine	Purines
ADP	Purines
AMP	Purines
GMP	Purines
Guanine	Purines
Guanosine	Purines
Hypoxanthine	Purines
IMP	Purines
Inosine	Purines
Uric acid	Purines
Xanthine	Purines
AICAR	Purines
FAICAR	Purines
Succinyl_AMP	Purines
ATP	Purines
GDP	Purines

**Analyte Name**	**Pathway**
GTP	Purines
Ribose-5P	Purines
PRPP	Purines
XMP	Purines
Xanthosine	Purines
dAMP	Purines
CTP	Pyrimidines
Orotic acid	Pyrimidines
UMP	Pyrimidines
Uridine	Pyrimidines
UTP	Pyrimidines
Ribose-5P	Pyrimidines
CMP	Pyrimidines
CDP	Pyrimidines
3-Ureidopropionic acid	Pyrimidines
Aspartate	Pyrimidines
Carbamoyl phosphate	Pyrimidines
UDP	Pyrimidines
Uracil	Pyrimidines
Dihydrouracil	Pyrimidines
Cytosine	Pyrimidines
PRPP	Pyrimidines
beta-Alanine	Pyrimidines
dCMP	Pyrimidines
TMP	Pyrimidines
TDP	Pyrimidines
TTP	Pyrimidines
6-Phosphogluconolactone	PPP
Glucose-6P	PPP
Erythrose-4-phosphate	PPP
Gluconate-6P	PPP
Hexose-P	PPP
Ribose-5P	PPP
Sedoheptulose-7P	PPP
Glyceraldehyde-3P	PPP
Xylulose-5P	PPP
Ribose	PPP
Alanine	Amino Acids
Arginine	Amino Acids
Asparagine	Amino Acids
Aspartate	Amino Acids
Cysteine	Amino Acids
Cystine	Amino Acids
Isoleucine	Amino Acids
Leucine	Amino Acids
Lysine	Amino Acids
Methionine	Amino Acids

Analyte Name	Pathway
Proline	Amino Acids
Serine	Amino Acids
Threonine	Amino Acids
Tryptophan	Amino Acids
Tyrosine	Amino Acids
Valine	Amino Acids
Glutamate	Amino Acids
Glutamine	Amino Acids
Histidine	Amino Acids
Glycine	Amino Acids
Phenylalanine	Amino Acids
3-phosphoglyceric acid	Amino Acids
Acetyl-CoA	Amino Acids
Alpha-Ketoglutarate	Amino Acids
Glucose	Amino Acids
Ornithine	Amino Acids
Phosphoserine	Amino Acids
Glutamate-5-semialdehyde	Amino Acids
Citric acid	Amino Acids
Pyruvate	Amino Acids
NAAD	NAD
NAADP	NAD
NADP+	NAD
NaMN	NAD
Nicotinamide	NAD
Nicotinamide riboside	NAD
NMN	NAD
NMNH	NAD
NRH	NAD
NAD+	NAD
NADH	NAD
NADPH	NAD
Nicotinic acid	NAD
ADP-Ribose	NAD
Tryptophan	NAD
Quinolinic acid	NAD
N-formylkynurenine	NAD
Ribose-5P	NAD
Methyl-NAM	NAD
2-PY	NAD
Kynurenic acid	Tryptophan
Kynurenine	Tryptophan
NAD+	Tryptophan
Tryptophan	Tryptophan
Melatonine	Tryptophan
Quinolinic acid	Tryptophan
Serotonine	Tryptophan

Analyte Name	Pathway
Aminoadipic acid	Tryptophan
Cysteine	Glutathion
Cystine	Glutathion
Glutamate	Glutathion
Glutathione	Glutathion
Ophthalmic acid	Glutathion
Oxiglutathione	Glutathion
Glycine	Glutathion
Pyroglutamic acid	Glutathion
Nitrosoglutathione	Glutathion
CoA-Glutathione	Glutathion
Gamma-Glutamylcysteine	Glutathion
Aminoadipic acid	Glutathion
Cystathionine	SAM-SAH
Cysteine	SAM-SAH
Cystine	SAM-SAH
Homocysteine	SAM-SAH
Homocystine	SAM-SAH
Methionine	SAM-SAH
Choline	SAM-SAH
S-adenosyl homocysteine	SAM-SAH
S-adenosyl methionine	SAM-SAH
Betaine	SAM-SAH
ATP	SAM-SAH
Adenosine	SAM-SAH
Serine	SAM-SAH
Alanine	SAM-SAH
Adenylthiomethylpentose	SAM-SAH
Taurine	SAM-SAH
Thiamine	SAM-SAH
CDP-ethanolamine	Lipid biosynthesis
CTP	Lipid biosynthesis
Glycerol-3P	Lipid biosynthesis
Phosphorylethanolamine	Lipid biosynthesis
Phosphoserine	Lipid biosynthesis
CDP-choline	Lipid biosynthesis
Choline	Lipid biosynthesis
Glycerophosphocholine	Lipid biosynthesis
Glycerophosphoinositol	Lipid biosynthesis
Arginine	Creatine
Creatine	Creatine
Creatine-P	Creatine
Creatinine	Creatine
Ornithine	Creatine
Glycine	Creatine
S-adenosyl methionine	Creatine
S-adenosyl homocysteine	Creatine

Analyte Name	Pathway
ADP	Creatine
ATP	Creatine
Guanidinoacetate	Creatine
