# Supplementary material for: HEPES in Cell Culture Alters the Multi‐Omics Profile Exhibited by Gaucher Disease Fibroblasts
Source: J Cell Biochem. 2026 Jan 16;127(1):e70080. doi: 10.1002/jcb.70080 (PMC12809196; doi:10.1002/jcb.70080)
Supplement: Supplementary file 3 — SupplTbl2_GCaseActivity_v2. [file JCB-127-e70080-s002.pdf]

Supplemental Table 2: GCase activity data in all conditions

Sample	Disease Group	Medium	HEPES	GCase activity [nmol/(mg*hour)]
C1	Control	DMEM	yes	1251
C2	Control	DMEM	yes	1351.3
C3	Control	DMEM	yes	1008.1
C4	Control	DMEM	yes	1293.1
C1	Control	DMEM	no	871.9
C2	Control	DMEM	no	1085.1
C3	Control	DMEM	no	920.5
C4	Control	DMEM	no	1120.4
C1	Control	Ham's F10	yes	1016.4
C2	Control	Ham's F10	yes	1562.7
C3	Control	Ham's F10	yes	1070.6
C4	Control	Ham's F10	yes	1363.3
C1	Control	Ham's F10	no	518.4
C2	Control	Ham's F10	no	960.1
C3	Control	Ham's F10	no	776.6
C4	Control	Ham's F10	no	815.9
G1	GD	DMEM	yes	85.7
G2	GD	DMEM	yes	24
G3	GD	DMEM	yes	374.9
G4	GD	DMEM	yes	225.4
G1	GD	DMEM	no	53.1
G2	GD	DMEM	no	17.5
G3	GD	DMEM	no	240.7
G4	GD	DMEM	no	164.7
G1	GD	Ham's F10	yes	62.3
G2	GD	Ham's F10	yes	20.9
G3	GD	Ham's F10	yes	332.1
G4	GD	Ham's F10	yes	210
G1	GD	Ham's F10	no	31.8
G2	GD	Ham's F10	no	18.7
G3	GD	Ham's F10	no	174.9
G4	GD	Ham's F10	no	113.5
