# Supplementary material for: HEPES in Cell Culture Alters the Multi‐Omics Profile Exhibited by Gaucher Disease Fibroblasts
Source: J Cell Biochem. 2026 Jan 16;127(1):e70080. doi: 10.1002/jcb.70080 (PMC12809196; doi:10.1002/jcb.70080)
Supplement: Supplementary file 4 — SupplTbl3_RelativeGCaseActivityPerPatient_v2. [file JCB-127-e70080-s007.pdf]

**Supplemental Table 3: GCase activity of individual GD samples relative to controls in the same culture condition**

| Donor | % of average C in same condition | | | | Max-Min_Difference | Average_Difference |
|---|---|---|---|---|---|---|
| | DMEM +HEPES | DMEM -HEPES | HAM +HEPES | HAM -HEPES | | |
| **GD1** | 7.00% | 5.30% | 5.00% | 4.10% | 2.80% | |
| **GD2** | 2.00% | 1.80% | 1.70% | 2.40% | 0.80% | |
| **GD3** | 30.60% | 24.10% | 26.50% | 22.80% | 7.80% | |
| **GD4** | 18.40% | 16.50% | 16.80% | 14.80% | 3.60% | |
| | | | | | | 3.80% |
