# Supplementary material for: HEPES in Cell Culture Alters the Multi‐Omics Profile Exhibited by Gaucher Disease Fibroblasts
Source: J Cell Biochem. 2026 Jan 16;127(1):e70080. doi: 10.1002/jcb.70080 (PMC12809196; doi:10.1002/jcb.70080)
Supplement: Supplementary file 5 — SupplTbl4_SphingolipidConc_v2. [file JCB-127-e70080-s004.pdf]

Supplemental Table 4: (Lyso-)Sphingolipid concentration data in all conditions

Medium	HEPES	Group	Experiment	Lipid	Log2 average compared to Control in Ham+HEPES
DMEM	No	Control	2	Lyso-Gb3(d18:1)	-0.2791
DMEM	No	Control	2	Lyso-Gb4(d18:1)	0.567238
DMEM	No	Control	2	Lyso-GlcCer(d18:1)	-0.13229
DMEM	No	Control	2	Lyso-GM3(d18:1)	0.476025
DMEM	No	Control	2	Lyso-SM(d18:1)	-0.6806
DMEM	No	Gaucher	2	Lyso-Gb3(d18:1)	0.319546
DMEM	No	Gaucher	2	Lyso-Gb4(d18:1)	1.031557
DMEM	No	Gaucher	2	Lyso-GlcCer(d18:1)	1.649662
DMEM	No	Gaucher	2	Lyso-GM3(d18:1)	0.651048
DMEM	No	Gaucher	2	Lyso-SM(d18:1)	-0.36685
DMEM	Yes	Control	2	Lyso-Gb3(d18:1)	0.095125
DMEM	Yes	Control	2	Lyso-Gb4(d18:1)	0.522585
DMEM	Yes	Control	2	Lyso-GlcCer(d18:1)	0.145826
DMEM	Yes	Control	2	Lyso-GM3(d18:1)	1.005611
DMEM	Yes	Control	2	Lyso-SM(d18:1)	0.138114
DMEM	Yes	Gaucher	2	Lyso-Gb3(d18:1)	0.012531
DMEM	Yes	Gaucher	2	Lyso-Gb4(d18:1)	0.496905
DMEM	Yes	Gaucher	2	Lyso-GlcCer(d18:1)	1.742419
DMEM	Yes	Gaucher	2	Lyso-GM3(d18:1)	0.686269
DMEM	Yes	Gaucher	2	Lyso-SM(d18:1)	-0.61455
HAM F10	No	Control	2	Lyso-Gb3(d18:1)	0.034703
HAM F10	No	Control	2	Lyso-Gb4(d18:1)	0.351649
HAM F10	No	Control	2	Lyso-GlcCer(d18:1)	0.061013
HAM F10	No	Control	2	Lyso-GM3(d18:1)	-0.0693
HAM F10	No	Control	2	Lyso-SM(d18:1)	-0.28037
HAM F10	No	Gaucher	3	Cer (mean)	0.102917
HAM F10	No	Gaucher	3	Cer(d17:1)	0.213928
HAM F10	No	Gaucher	3	Cer(d18:1)	0.06779
HAM F10	No	Gaucher	3	Cer(d18:2)	0.338854
HAM F10	No	Gaucher	3	Cer(d20:1)	0.245801
HAM F10	No	Gaucher	3	CTH(d18:1)	0.66444
HAM F10	No	Gaucher	3	CTH(d18:2)	0.836829
HAM F10	No	Gaucher	3	DHcer (mean)	0.144436
HAM F10	No	Gaucher	3	DHCer(d18:0)	0.146511
HAM F10	No	Gaucher	3	DHCer(m18:0)	-0.09671
HAM F10	No	Gaucher	3	Gb3 (mean)	0.672807
HAM F10	No	Gaucher	3	GlcCer (mean)	0.118893
HAM F10	No	Gaucher	3	GlcCer(d18:1)	0.101075
HAM F10	No	Gaucher	3	GlcCer(d18:2)	0.534057
HAM F10	No	Gaucher	3	LacCer (mean)	0.415604
HAM F10	No	Gaucher	3	LacCer(d18:1)	0.404031
HAM F10	No	Gaucher	3	LacCer(d18:2)	0.611505
HAM F10	No	Gaucher	2	Lyso-Gb3(d18:1)	0.143256

Medium	HEPES	Group	Experiment	Lipid	Log2 average compared to Control in Ham+HEPES
HAM F10	No	Gaucher	3	Lyso-Gb3(d18:1)	0.279073
HAM F10	No	Gaucher	2	Lyso-Gb4(d18:1)	-0.07459
HAM F10	No	Gaucher	2	Lyso-GlcCer(d18:1)	0.708714
HAM F10	No	Gaucher	3	Lyso-GlcCer(d18:1)	1.965306
HAM F10	No	Gaucher	2	Lyso-GM3(d18:1)	-0.74757
HAM F10	No	Gaucher	2	Lyso-SM(d18:1)	-0.08259
HAM F10	No	Gaucher	3	Lyso-SM(d18:1)	-0.05269
HAM F10	Yes	Gaucher	1	Cer (mean)	-0.11185
HAM F10	Yes	Gaucher	1	DHcer (mean)	-0.16089
HAM F10	Yes	Gaucher	1	Gb3 (mean)	0.32849
HAM F10	Yes	Gaucher	1	GlcCer (mean)	0.445139
HAM F10	Yes	Gaucher	1	LacCer (mean)	0.630575
HAM F10	Yes	Gaucher	1	Lyso-Gb3(d18:1)	0.035531
HAM F10	Yes	Gaucher	2	Lyso-Gb3(d18:1)	0.244394
HAM F10	Yes	Gaucher	2	Lyso-Gb4(d18:1)	0.084457
HAM F10	Yes	Gaucher	1	Lyso-GlcCer(d18:1)	0.849361
HAM F10	Yes	Gaucher	2	Lyso-GlcCer(d18:1)	0.836883
HAM F10	Yes	Gaucher	2	Lyso-GM3(d18:1)	0.108585
HAM F10	Yes	Gaucher	1	Lyso-SM(d18:1)	-0.57661
HAM F10	Yes	Gaucher	2	Lyso-SM(d18:1)	-0.07203
HAM F10	Yes	Gaucher	1	Lyso-SM-509	-0.21067
