# Supplementary material for: HEPES in Cell Culture Alters the Multi‐Omics Profile Exhibited by Gaucher Disease Fibroblasts
Source: J Cell Biochem. 2026 Jan 16;127(1):e70080. doi: 10.1002/jcb.70080 (PMC12809196; doi:10.1002/jcb.70080)
Supplement: Supplementary file 6 — SupplTbl5_Lipidomics_v3. [file JCB-127-e70080-s005.pdf]

Supplemental Table 5: Lipidomics data

NoH: cultured without HEPES; WithH: cultured with HEPES; all cultures in Ham's F10

Lipid	LipidClass	Log2FC_noH	PVal_noH	Log2FC_withH	PVal_withH
BMP(30:0)	BMP	0.118504	0.796489	#N/A	#N/A
BMP(30:1)	BMP	0.116863	0.798479	#N/A	#N/A
BMP(30:2)	BMP	-0.07559	0.835312	#N/A	#N/A
BMP(31:0)	BMP	0.333403	0.443263	-0.05592	0.841808
BMP(31:1)	BMP	0.327499	0.291103	-0.36565	0.462465
BMP(32:0)	BMP	0.039929	0.923798	-0.23329	0.568407
BMP(32:1)	BMP	-0.109	0.80063	-0.23405	0.475784
BMP(32:2)	BMP	-0.10538	0.79682	-0.19582	0.620449
BMP(32:3)	BMP	-0.01004	0.975889	-0.18289	0.745866
BMP(32:4)	BMP	0.536802	0.341166	#N/A	#N/A
BMP(33:0)	BMP	0.242002	0.524564	-0.19838	0.590493
BMP(33:1)	BMP	-0.01643	0.958184	-0.40491	0.281701
BMP(33:2)	BMP	-0.1048	0.72994	-0.4539	0.309805
BMP(33:3)	BMP	-0.19671	0.579988	-0.1851	0.685144
BMP(34:0)	BMP	0.228483	0.529523	-0.15056	0.599716
BMP(34:1)	BMP	-0.19937	0.592621	-0.26847	0.469189
BMP(34:2)	BMP	-0.2955	0.444747	-0.29044	0.399757
BMP(34:3)	BMP	-0.31013	0.342069	-0.15759	0.621555
BMP(34:4)	BMP	-0.05405	0.849415	#N/A	#N/A
BMP(34:5)	BMP	0.32021	0.446792	#N/A	#N/A
BMP(35:0)	BMP	#N/A	#N/A	-0.19049	0.663836
BMP(35:1)	BMP	-0.05516	0.851636	#N/A	#N/A
BMP(35:2)	BMP	-0.27727	0.353164	-0.25588	0.458294
BMP(35:3)	BMP	-0.16441	0.44592	#N/A	#N/A
BMP(35:4)	BMP	-0.01078	0.966022	#N/A	#N/A
BMP(35:5)	BMP	0.222201	0.652074	#N/A	#N/A
BMP(36:0)	BMP	0.820884	0.046982	#N/A	#N/A
BMP(36:1)	BMP	-0.09406	0.74123	-0.08634	0.780348
BMP(36:2)	BMP	-0.47021	0.207799	-0.16644	0.655372
BMP(36:3)	BMP	-0.31617	0.248408	-0.13713	0.617737
BMP(36:4)	BMP	-0.1413	0.442989	#N/A	#N/A
BMP(36:5)	BMP	-0.03482	0.848123	0.21206	0.331023
BMP(36:6)	BMP	0.417039	0.082006	#N/A	#N/A
BMP(36:7)	BMP	0.220581	0.687481	#N/A	#N/A
BMP(37:1)	BMP	0.144005	0.715681	-0.15624	0.616753
BMP(37:2)	BMP	-0.42289	0.257015	-0.0433	0.908434
BMP(37:3)	BMP	-0.36771	0.246895	#N/A	#N/A
BMP(37:4)	BMP	-0.3148	0.298561	#N/A	#N/A
BMP(37:5)	BMP	-0.18948	0.474589	#N/A	#N/A
BMP(37:6)	BMP	0.37051	0.131918	#N/A	#N/A
BMP(37:7)	BMP	0.417112	0.428574	#N/A	#N/A
BMP(38:1)	BMP	-0.18306	0.65413	0.133401	0.739436
BMP(38:2)	BMP	-0.49281	0.275521	0.026461	0.944056
BMP(38:3)	BMP	-0.57745	0.22603	0.073063	0.817105

Lipid	LipidClass	Log2FC_noH	PVal_noH	Log2FC_withH	PVal_withH
BMP(38:4)	BMP	-0.41158	0.212215	#N/A	#N/A
BMP(38:5)	BMP	-0.21947	0.365856	0.253648	0.154129
BMP(38:6)	BMP	0.33884	0.047365	#N/A	#N/A
BMP(38:7)	BMP	0.453326	0.04281	0.137774	0.411903
BMP(38:8)	BMP	0.327412	0.386943	#N/A	#N/A
BMP(38:9)	BMP	0.97078	0.25364	#N/A	#N/A
BMP(39:2)	BMP	-0.28359	0.572868	-0.17516	0.755182
BMP(39:3)	BMP	-0.52421	0.188822	#N/A	#N/A
BMP(39:4)	BMP	-0.57932	0.156286	#N/A	#N/A
BMP(39:5)	BMP	-0.34977	0.339413	#N/A	#N/A
BMP(39:6)	BMP	0.061425	0.746337	#N/A	#N/A
BMP(39:7)	BMP	0.388722	0.251367	0.117401	0.591261
BMP(39:8)	BMP	0.681681	0.17168	#N/A	#N/A
BMP(40:10)	BMP	1.565377	0.144443	#N/A	#N/A
BMP(40:3)	BMP	-0.32755	0.389454	#N/A	#N/A
BMP(40:4)	BMP	-0.51307	0.167034	0.299941	0.233666
BMP(40:5)	BMP	-0.31155	0.234465	0.275368	0.281861
BMP(40:6)	BMP	0.040623	0.818978	0.338894	0.254674
BMP(40:7)	BMP	0.202869	0.471044	0.177629	0.50696
BMP(40:8)	BMP	0.320063	0.41689	0.182587	0.28478
BMP(40:9)	BMP	0.367083	0.521012	0.307129	0.061708
BMP(41:10)	BMP	#N/A	#N/A	0.258488	0.213823
BMP(41:4)	BMP	-0.70899	0.116275	#N/A	#N/A
BMP(41:5)	BMP	-0.73043	0.10411	#N/A	#N/A
BMP(41:6)	BMP	-0.28636	0.384458	#N/A	#N/A
BMP(41:7)	BMP	-0.04203	0.859879	#N/A	#N/A
BMP(41:8)	BMP	0.202376	0.381136	#N/A	#N/A
BMP(41:9)	BMP	0.333866	0.234716	#N/A	#N/A
BMP(42:10)	BMP	0.564393	0.159353	0.460607	0.042156
BMP(42:11)	BMP	0.933133	0.315408	0.406696	0.055809
BMP(42:5)	BMP	-0.65172	0.116383	#N/A	#N/A
BMP(42:6)	BMP	-0.13193	0.600573	#N/A	#N/A
BMP(42:7)	BMP	-0.08998	0.691378	0.396931	0.155862
BMP(42:8)	BMP	0.105516	0.568953	0.418596	0.105927
BMP(42:9)	BMP	0.395813	0.115638	0.465965	0.056905
BMP(43:10)	BMP	-0.02936	0.939199	#N/A	#N/A
BMP(43:11)	BMP	0.389467	0.474809	#N/A	#N/A
BMP(43:7)	BMP	-0.56843	0.244633	#N/A	#N/A
BMP(43:8)	BMP	-0.06078	0.85321	#N/A	#N/A
BMP(43:9)	BMP	-0.08365	0.779528	#N/A	#N/A
BMP(44:10)	BMP	0.363155	0.241394	0.484501	0.080448
BMP(44:11)	BMP	0.530977	0.251296	0.5189	0.125804
BMP(44:12)	BMP	0.427462	0.580606	0.372454	0.204395
BMP(44:7)	BMP	0.15145	0.75068	#N/A	#N/A
BMP(44:8)	BMP	0.030387	0.923133	#N/A	#N/A
BMP(44:9)	BMP	0.108632	0.676143	#N/A	#N/A
BMP(46:11)	BMP	#N/A	#N/A	0.387513	0.276228

Lipid	LipidClass	Log2FC_noH	PVal_noH	Log2FC_withH	PVal_withH
BMP(46:12)	BMP	#N/A	#N/A	0.416969	0.321828
BMP(46:9)	BMP	#N/A	#N/A	1.133681	0.174803
CE(13:0)	CE	#N/A	#N/A	0.011325	0.952521
CE(14:0)	CE	0.334088	0.261942	-0.06324	0.871991
CE(14:1)	CE	0.317352	0.372841	0.463222	0.464094
CE(15:0)	CE	-0.09615	0.692891	0.200598	0.531777
CE(15:1)	CE	0.031119	0.903994	0.23246	0.678314
CE(15:2)	CE	0.193307	0.730559	#N/A	#N/A
CE(16:0)	CE	0.123264	0.631877	0.352995	0.283315
CE(16:1)	CE	0.106708	0.686471	0.205435	0.537168
CE(16:2)	CE	0.264828	0.371484	0.3216	0.52767
CE(16:3)	CE	0.29067	0.464205	0.333747	0.643226
CE(16:4)	CE	0.522403	0.498587	0.22311	0.79022
CE(17:0)	CE	0.094273	0.763864	0.413024	0.262941
CE(17:1)	CE	#N/A	#N/A	0.187421	0.404409
CE(17:2)	CE	0.172618	0.506212	0.302097	0.505104
CE(18:0)	CE	0.082908	0.782888	0.456229	0.140655
CE(18:1)	CE	0.131476	0.632163	0.469754	0.174784
CE(18:2)	CE	0.043563	0.888925	0.414897	0.18859
CE(18:3)	CE	0.041445	0.915217	0.396206	0.453199
CE(18:4)	CE	0.317947	0.600439	0.453685	0.554092
CE(19:0)	CE	0.061848	0.824516	0.418894	0.16171
CE(19:1)	CE	0.194585	0.500809	0.52439	0.241259
CE(19:2)	CE	0.369918	0.238357	0.32395	0.592363
CE(19:3)	CE	0.387276	0.24063	0.476149	0.32193
CE(19:4)	CE	0.181783	0.636714	0.452054	0.44064
CE(20:0)	CE	0.069501	0.802952	0.428817	0.157755
CE(20:1)	CE	0.330737	0.339758	0.548119	0.250299
CE(20:2)	CE	0.496195	0.23488	0.571972	0.272063
CE(20:3)	CE	0.435246	0.282687	0.522961	0.405058
CE(20:4)	CE	0.00061	0.998613	0.559961	0.17005
CE(20:5)	CE	0.16486	0.797907	0.703764	0.216162
CE(21:0)	CE	-0.12898	0.545415	0.119359	0.642126
CE(21:1)	CE	0.383014	0.206728	0.584456	0.269042
CE(21:2)	CE	0.370568	0.194123	0.399051	0.453556
CE(21:3)	CE	0.451485	0.152258	0.586741	0.240921
CE(21:4)	CE	0.377563	0.312025	0.555301	0.281879
CE(21:5)	CE	0.540239	0.398134	0.636953	0.25973
CE(22:0)	CE	0.256316	0.375012	0.527767	0.116906
CE(22:1)	CE	0.468381	0.322602	0.693537	0.206141
CE(22:2)	CE	0.587147	0.213312	0.644739	0.288772
CE(22:3)	CE	0.607071	0.128241	0.506245	0.482165
CE(22:4)	CE	0.612814	0.139366	0.655721	0.221722
CE(22:5)	CE	0.440666	0.446788	0.688904	0.200879
CE(22:6)	CE	0.062652	0.904467	0.557555	0.278871
CE(22:7)	CE	#N/A	#N/A	0.728678	0.365301
CE(23:0)	CE	0.292822	0.273643	0.264734	0.274833

Lipid	LipidClass	Log2FC_noH	PVal_noH	Log2FC_withH	PVal_withH
CE(23:1)	CE	0.455494	0.209292	0.529166	0.352242
CE(23:2)	CE	0.691167	0.168218	0.413933	0.499919
CE(23:3)	CE	0.645249	0.152976	0.601124	0.312546
CE(23:4)	CE	1.056892	0.09973	0.6878	0.20312
CE(23:5)	CE	0.747856	0.36173	0.598829	0.256914
CE(23:6)	CE	0.280825	0.740367	0.567225	0.270681
CE(24:0)	CE	0.372669	0.235224	0.651022	0.164071
CE(24:1)	CE	0.548445	0.26493	0.638107	0.285108
CE(24:2)	CE	0.596093	0.235983	0.538463	0.413285
CE(24:3)	CE	0.746021	0.103445	0.479507	0.516762
CE(24:4)	CE	0.592265	0.252801	0.695321	0.202283
CE(24:5)	CE	0.175229	0.83804	0.358303	0.545984
CE(24:6)	CE	0.446833	0.618292	0.238518	0.7644
CE(25:0)	CE	0.226841	0.200869	0.401412	0.186177
CE(25:1)	CE	0.597082	0.171491	0.545669	0.370204
CE(25:2)	CE	0.643682	0.242122	0.444863	0.496723
CE(25:3)	CE	0.877609	0.083955	0.486282	0.502412
CE(25:4)	CE	0.847922	0.146331	0.417806	0.498341
CE(25:5)	CE	0.530967	0.542404	0.318246	0.582478
CE(26:0)	CE	0.598901	0.193679	0.541603	0.313435
CE(26:1)	CE	0.75433	0.226596	0.515716	0.437098
CE(26:2)	CE	0.659038	0.263097	0.473247	0.479821
CE(26:3)	CE	0.854616	0.102859	0.394441	0.619399
CE(26:4)	CE	0.86489	0.123712	0.562668	0.382992
CE(26:5)	CE	0.872388	0.253607	0.194545	0.820895
CE(26:6)	CE	0.482234	0.606771	0.084963	0.928701
CE(27:0)	CE	#N/A	#N/A	0.201362	0.530371
CE(27:1)	CE	#N/A	#N/A	0.280671	0.620491
CE(27:2)	CE	#N/A	#N/A	0.2073	0.705986
CE(27:3)	CE	#N/A	#N/A	0.246033	0.715847
CE(27:4)	CE	#N/A	#N/A	0.358664	0.546315
CE(27:5)	CE	#N/A	#N/A	0.247065	0.708173
CE(28:0)	CE	#N/A	#N/A	0.474036	0.116465
CE(28:1)	CE	#N/A	#N/A	0.444753	0.36166
CE(28:2)	CE	#N/A	#N/A	0.237592	0.69819
CE(28:3)	CE	#N/A	#N/A	0.131975	0.883925
CE(28:4)	CE	#N/A	#N/A	0.335117	0.646384
CE(28:5)	CE	#N/A	#N/A	-0.08186	0.939166
CE(29:0)	CE	#N/A	#N/A	0.409332	0.334116
CE(29:1)	CE	#N/A	#N/A	0.074499	0.867135
CE(29:2)	CE	#N/A	#N/A	-0.05674	0.907783
CE(29:3)	CE	#N/A	#N/A	0.155246	0.796224
CE(29:4)	CE	#N/A	#N/A	0.12861	0.82924
CE(29:5)	CE	#N/A	#N/A	-0.12862	0.867412
CE(30:1)	CE	#N/A	#N/A	0.173855	0.388432
CE(30:2)	CE	#N/A	#N/A	0.36606	0.519752
CE(30:3)	CE	#N/A	#N/A	0.25034	0.740898

Lipid	LipidClass	Log2FC_noH	PVal_noH	Log2FC_withH	PVal_withH
CE(30:4)	CE	#N/A	#N/A	0.31318	0.646309
CE(30:5)	CE	#N/A	#N/A	0.202325	0.761873
CE(31:4)	CE	#N/A	#N/A	0.088974	0.849665
CE(31:5)	CE	#N/A	#N/A	0.106227	0.865369
CE(32:1)	CE	#N/A	#N/A	0.287212	0.262639
CE(32:3)	CE	#N/A	#N/A	-0.15741	0.721526
CE(32:4)	CE	#N/A	#N/A	0.103075	0.791223
CE(32:5)	CE	#N/A	#N/A	0.341922	0.628159
CE(33:5)	CE	#N/A	#N/A	-0.06465	0.910572
CE(34:3)	CE	#N/A	#N/A	0.165114	0.467617
CE(34:4)	CE	#N/A	#N/A	0.401888	0.061162
CE(34:5)	CE	#N/A	#N/A	0.077789	0.855304
Cer(d28:1)	Cer	#N/A	#N/A	0.340226	0.359715
Cer(d29:1)	Cer	#N/A	#N/A	0.24055	0.231627
Cer(d30:2)	Cer	#N/A	#N/A	0.037646	0.915715
Cer(d32:0)	Cer	0.170234	0.681609	-0.64149	0.044949
Cer(d32:1)	Cer	0.19035	0.36567	-0.44367	0.042262
Cer(d32:2)	Cer	#N/A	#N/A	-0.04992	0.786935
Cer(d33:0)	Cer	#N/A	#N/A	0.199765	0.409224
Cer(d33:1)	Cer	0.288385	0.307552	-0.49658	0.088623
Cer(d33:2)	Cer	#N/A	#N/A	0.033859	0.953729
Cer(d34:0)	Cer	0.332443	0.331274	-0.5349	0.049628
Cer(d34:1)	Cer	0.283123	0.255714	-0.56596	0.038394
Cer(d34:2)	Cer	0.448358	0.107469	-0.28782	0.18589
Cer(d34:3)	Cer	#N/A	#N/A	-1.66594	0.00408
Cer(d35:0)	Cer	0.574538	0.260828	-0.59139	0.103266
Cer(d35:1)	Cer	0.331266	0.328908	-0.5753	0.037869
Cer(d35:2)	Cer	#N/A	#N/A	-0.63109	0.034429
Cer(d35:3)	Cer	#N/A	#N/A	0.046377	0.886307
Cer(d36:0)	Cer	0.511569	0.224631	-0.70908	0.046511
Cer(d36:1)	Cer	0.23595	0.459208	-0.58208	0.045233
Cer(d36:2)	Cer	0.349649	0.283242	-0.59384	0.055359
Cer(d37:0)	Cer	0.566947	0.265069	-0.09138	0.827015
Cer(d37:1)	Cer	#N/A	#N/A	-0.36088	0.264529
Cer(d38:0)	Cer	0.479085	0.307863	-0.53844	0.107452
Cer(d38:1)	Cer	0.345124	0.301923	-0.55002	0.079682
Cer(d38:2)	Cer	0.352083	0.237744	-0.83526	0.030665
Cer(d39:0)	Cer	0.395736	0.199016	-0.11377	0.594006
Cer(d39:1)	Cer	0.441391	0.247986	-0.49357	0.063481
Cer(d39:2)	Cer	0.553276	0.145449	-0.62458	0.049575
Cer(d40:0)	Cer	0.472958	0.193575	-0.34586	0.23449
Cer(d40:1)	Cer	0.243572	0.30421	-0.56046	0.031937
Cer(d40:2)	Cer	0.326445	0.181252	-0.59746	0.013412
Cer(d40:3)	Cer	0.481568	0.0968	-0.66664	0.005415
Cer(d41:0)	Cer	0.298259	0.165431	-0.00374	0.980147
Cer(d41:1)	Cer	0.24077	0.204363	-0.50319	0.032462
Cer(d41:2)	Cer	0.312387	0.198923	-0.52773	0.017619

Lipid	LipidClass	Log2FC_noH	PVal_noH	Log2FC_withH	PVal_withH
Cer(d41:3)	Cer	0.389829	0.045631	-0.45469	0.003081
Cer(d42:0)	Cer	0.450526	0.062798	-0.106	0.639309
Cer(d42:1)	Cer	0.178558	0.183553	-0.44568	0.038241
Cer(d42:2)	Cer	0.134645	0.419418	-0.48342	0.017471
Cer(d42:3)	Cer	0.399288	0.07076	-0.60547	0.000833
Cer(d42:4)	Cer	0.441372	0.05646	-0.60045	0.003684
Cer(d42:5)	Cer	0.507231	0.085743	-0.51755	0.141396
Cer(d43:0)	Cer	0.163812	0.434548	0.000874	0.995944
Cer(d43:2)	Cer	0.065052	0.706275	-0.60393	0.012086
Cer(d43:3)	Cer	0.243029	0.092949	-0.44609	0.007403
Cer(d43:4)	Cer	0.251147	0.330375	-0.61907	0.008209
Cer(d44:0)	Cer	0.151998	0.283938	0.024083	0.854466
Cer(d44:1)	Cer	0.069549	0.715085	-0.52107	0.025689
Cer(d44:2)	Cer	-0.06927	0.792994	-0.60291	0.007245
Cer(d44:3)	Cer	0.099999	0.712242	-0.67105	0.000727
Cer(d44:4)	Cer	0.278919	0.303215	-0.58854	0.016267
Cer(d44:5)	Cer	0.456804	0.14708	-0.47976	0.024399
Cer(d44:6)	Cer	#N/A	#N/A	-1.07864	0.060396
Cer(d45:0)	Cer	0.014085	0.897709	0.0661	0.615826
Cer(d45:1)	Cer	-0.00587	0.968953	-0.19763	0.267526
Cer(d45:2)	Cer	-0.03572	0.864762	-0.6243	0.040482
Cer(d45:3)	Cer	-0.01802	0.945899	-0.61265	0.008947
Cer(d46:0)	Cer	-0.04776	0.652717	0.142737	0.356237
Cer(d46:1)	Cer	-0.08102	0.580632	0.002978	0.985639
Cer(d46:2)	Cer	0.042874	0.880461	-0.4866	0.148976
Cer(d46:3)	Cer	-0.13934	0.686692	-0.82936	0.001662
Cer(d46:4)	Cer	0.256815	0.499905	-0.6776	0.035033
Cer(d46:5)	Cer	#N/A	#N/A	-1.57297	0.078609
Cer(d47:0)	Cer	#N/A	#N/A	0.224416	0.114277
Cer(d47:1)	Cer	#N/A	#N/A	0.323794	0.070471
Cer(d48:0)	Cer	#N/A	#N/A	0.245702	0.374494
Cer(d48:1)	Cer	#N/A	#N/A	0.367225	0.06504
Cer(d48:2)	Cer	#N/A	#N/A	-0.35781	0.344261
Cer(d49:0)	Cer	#N/A	#N/A	0.212747	0.258037
Cer(d49:1)	Cer	#N/A	#N/A	0.161829	0.333502
Cer(d50:0)	Cer	#N/A	#N/A	0.412657	0.023322
Cer(d50:1)	Cer	#N/A	#N/A	0.245607	0.139121
Cer(d51:0)	Cer	#N/A	#N/A	-0.02884	0.95332
Cer(d51:1)	Cer	#N/A	#N/A	0.110433	0.804811
Cer(d52:1)	Cer	#N/A	#N/A	-0.18852	0.699233
CL(62:3)	CL	0.70682	0.531465	#N/A	#N/A
CL(64:2)	CL	1.034399	0.14393	#N/A	#N/A
CL(64:3)	CL	0.571019	0.496692	#N/A	#N/A
CL(64:4)	CL	0.014509	0.984857	#N/A	#N/A
CL(65:3)	CL	0.253183	0.715462	#N/A	#N/A
CL(65:4)	CL	0.030709	0.962	#N/A	#N/A
CL(66:2)	CL	0.542791	0.041946	#N/A	#N/A

Lipid	LipidClass	Log2FC_noH	PVal_noH	Log2FC_withH	PVal_withH
CL(66:3)	CL	0.193364	0.723738	#N/A	#N/A
CL(66:4)	CL	0.009112	0.989049	0.303086	0.567152
CL(66:5)	CL	-0.06314	0.904505	-0.15674	0.638435
CL(67:3)	CL	0.05875	0.891578	#N/A	#N/A
CL(67:4)	CL	-0.01176	0.981229	#N/A	#N/A
CL(67:5)	CL	-0.06948	0.8632	-0.07837	0.857012
CL(68:2)	CL	0.282509	0.220123	#N/A	#N/A
CL(68:3)	CL	0.134328	0.719734	#N/A	#N/A
CL(68:4)	CL	-0.06594	0.908859	0.213746	0.910439
CL(68:5)	CL	-0.07251	0.857295	0.325572	0.347941
CL(68:6)	CL	0.074565	0.784734	-0.20777	0.4503
CL(68:7)	CL	-0.02156	0.960631	#N/A	#N/A
CL(69:3)	CL	0.26338	0.392369	#N/A	#N/A
CL(69:4)	CL	-0.05082	0.900624	#N/A	#N/A
CL(69:5)	CL	0.070124	0.779374	0.309873	0.310944
CL(69:6)	CL	0.236167	0.2024	-0.01703	0.942675
CL(69:7)	CL	0.272849	0.485672	#N/A	#N/A
CL(70:3)	CL	0.105073	0.697195	#N/A	#N/A
CL(70:4)	CL	-0.08171	0.849435	0.217601	0.438083
CL(70:5)	CL	-0.06091	0.836938	1.010028	0.398897
CL(70:6)	CL	0.218902	0.187871	0.088855	0.578541
CL(70:7)	CL	0.173765	0.535384	-0.1962	0.473606
CL(70:8)	CL	0.018486	0.950173	-0.21833	0.364396
CL(71:4)	CL	0.015077	0.970333	#N/A	#N/A
CL(71:5)	CL	0.033825	0.851262	1.487813	0.062723
CL(71:6)	CL	0.359828	0.107205	0.171181	0.358835
CL(71:7)	CL	0.272122	0.503803	-0.0706	0.740324
CL(71:8)	CL	0.325067	0.224815	#N/A	#N/A
CL(72:10)	CL	0.280572	0.49286	#N/A	#N/A
CL(72:4)	CL	0.159615	0.671447	#N/A	#N/A
CL(72:5)	CL	0.032024	0.869687	#N/A	#N/A
CL(72:6)	CL	0.292241	0.209758	0.574428	0.184092
CL(72:7)	CL	0.235395	0.647939	0.123326	0.477952
CL(72:8)	CL	-0.12648	0.866876	-0.15929	0.574366
CL(72:9)	CL	0.298855	0.112362	-0.05202	0.860851
CL(73:6)	CL	0.133366	0.229882	-0.02138	0.982643
CL(73:7)	CL	0.198344	0.170492	-0.03518	0.912108
CL(73:8)	CL	0.403335	0.070727	0.162619	0.535428
CL(73:9)	CL	0.408044	0.168626	#N/A	#N/A
CL(74:10)	CL	0.433289	0.380865	0.070776	0.802028
CL(74:11)	CL	0.507604	0.310021	#N/A	#N/A
CL(74:6)	CL	0.14786	0.477189	#N/A	#N/A
CL(74:7)	CL	0.210507	0.198947	1.648156	0.311917
CL(74:8)	CL	0.445273	0.094255	0.659868	0.143802
CL(74:9)	CL	0.457196	0.280473	0.189571	0.324472
CL(75:8)	CL	0.289091	0.363213	#N/A	#N/A
CL(75:9)	CL	0.437373	0.147425	#N/A	#N/A

Lipid	LipidClass	Log2FC_noH	PVal_noH	Log2FC_withH	PVal_withH
CL(76:10)	CL	0.456605	0.141988	0.385378	0.370348
CL(76:11)	CL	0.459857	0.350315	0.086463	0.697586
CL(76:12)	CL	0.411963	0.583765	#N/A	#N/A
CL(76:8)	CL	-0.25114	0.22851	#N/A	#N/A
CL(76:9)	CL	0.289215	0.070736	-0.54562	0.576938
CL(78:11)	CL	0.846179	0.059322	#N/A	#N/A
CL(78:12)	CL	1.38952	0.025616	#N/A	#N/A
DG(24:0)	DG	#N/A	#N/A	0.090289	0.649709
DG(26:0)	DG	#N/A	#N/A	0.251211	0.165456
DG(29:0)	DG	0.35342	0.064778	-0.14813	0.609142
DG(30:0)	DG	0.140488	0.590254	-0.1521	0.464088
DG(30:1)	DG	0.096995	0.780631	0.041243	0.90784
DG(31:0)	DG	0.415961	0.04711	-0.14991	0.384515
DG(31:1)	DG	0.241717	0.187981	-0.12821	0.612381
DG(31:2)	DG	0.003859	0.993757	#N/A	#N/A
DG(32:0)	DG	0.21437	0.361497	-0.11658	0.458805
DG(32:1)	DG	0.19327	0.541594	-0.26088	0.386558
DG(32:2)	DG	0.142912	0.665419	-0.13678	0.596757
DG(32:3)	DG	0.039277	0.897973	#N/A	#N/A
DG(33:0)	DG	0.414124	0.038878	-0.03609	0.790552
DG(33:1)	DG	0.365999	0.017331	-0.18212	0.30715
DG(33:2)	DG	0.26767	0.127059	-0.12639	0.619106
DG(34:0)	DG	0.284651	0.096815	0.011526	0.953121
DG(34:1)	DG	0.19139	0.441972	-0.12258	0.412963
DG(34:2)	DG	0.255507	0.344911	-0.1193	0.548928
DG(34:3)	DG	0.188837	0.420845	-0.03092	0.916863
DG(34:4)	DG	0.550783	0.01766	0.015019	0.953527
DG(35:0)	DG	0.32765	0.101822	-0.24623	0.371772
DG(35:1)	DG	0.350401	0.026076	-0.05492	0.734087
DG(35:2)	DG	0.30734	0.084611	-0.13678	0.435952
DG(35:3)	DG	0.567582	0.070414	-0.11924	0.721452
DG(35:4)	DG	0.543247	0.020435	0.065994	0.688704
DG(36:0)	DG	0.213133	0.157785	0.156352	0.437681
DG(36:1)	DG	0.268088	0.129396	-0.05366	0.780103
DG(36:2)	DG	0.183382	0.439801	-0.15414	0.351159
DG(36:3)	DG	0.24827	0.254908	-0.03093	0.881981
DG(36:4)	DG	0.5026	0.041729	-0.09012	0.678721
DG(36:5)	DG	0.476133	0.001321	-0.11967	0.593458
DG(36:6)	DG	0.342851	0.33598	-0.01109	0.96562
DG(37:0)	DG	0.11867	0.456188	0.271588	0.366377
DG(37:1)	DG	0.191154	0.323864	-0.04833	0.817681
DG(37:2)	DG	0.147475	0.556668	-0.12363	0.543197
DG(37:3)	DG	0.188626	0.459467	-0.09787	0.613578
DG(37:4)	DG	0.621406	0.108399	-0.0468	0.802499
DG(37:5)	DG	0.860777	0.016688	-0.05358	0.86526
DG(37:6)	DG	#N/A	#N/A	-0.38236	0.238435
DG(38:0)	DG	0.084216	0.608483	-0.0093	0.972404

Lipid	LipidClass	Log2FC_noH	PVal_noH	Log2FC_withH	PVal_withH
DG(38:1)	DG	0.039635	0.879904	-0.04042	0.877292
DG(38:2)	DG	-0.00561	0.989057	-0.22683	0.390885
DG(38:3)	DG	-0.00451	0.9866	-0.01113	0.94716
DG(38:4)	DG	0.439649	0.243264	-0.04489	0.816837
DG(38:5)	DG	0.551435	0.088627	-0.12456	0.503842
DG(38:6)	DG	0.496045	0.011548	-0.10231	0.60458
DG(38:7)	DG	0.240325	0.449546	-0.16916	0.511638
DG(39:0)	DG	0.119545	0.33528	0.159585	0.413369
DG(39:1)	DG	0.209428	0.388481	-0.09832	0.687385
DG(39:2)	DG	0.067977	0.825769	-0.11114	0.624465
DG(39:3)	DG	0.003956	0.991789	-0.16244	0.580001
DG(39:4)	DG	0.24365	0.301941	-0.09862	0.598565
DG(39:5)	DG	0.501273	0.083209	-0.11388	0.477935
DG(39:6)	DG	0.701352	0.024665	-0.18117	0.349965
DG(39:7)	DG	0.457972	0.076725	#N/A	#N/A
DG(40:0)	DG	0.208308	0.249601	0.272913	0.278685
DG(40:1)	DG	0.086332	0.786288	0.052824	0.848776
DG(40:2)	DG	-0.11388	0.799991	-0.05377	0.831047
DG(40:3)	DG	-0.25966	0.553339	-0.07674	0.753419
DG(40:4)	DG	-0.00053	0.998248	-0.09559	0.685457
DG(40:5)	DG	0.283085	0.249485	-0.06302	0.714912
DG(40:6)	DG	0.32442	0.152168	-0.00063	0.996968
DG(40:7)	DG	0.333314	0.081017	-0.13795	0.486941
DG(40:8)	DG	0.319821	0.27431	-0.19479	0.353393
DG(40:9)	DG	#N/A	#N/A	-0.47083	0.244259
DG(41:0)	DG	0.073764	0.633932	0.226137	0.206751
DG(41:1)	DG	0.169056	0.459074	0.098003	0.666774
DG(41:2)	DG	0.004831	0.989909	0.005555	0.984515
DG(41:3)	DG	-0.16519	0.657947	-0.13588	0.620749
DG(41:4)	DG	-0.05458	0.889962	0.021842	0.911618
DG(41:5)	DG	0.111176	0.731633	-0.0548	0.748336
DG(41:6)	DG	#N/A	#N/A	-0.04119	0.866499
DG(41:7)	DG	#N/A	#N/A	0.012169	0.958534
DG(42:0)	DG	0.213348	0.247919	0.251035	0.250558
DG(42:1)	DG	0.156369	0.60621	0.17581	0.514664
DG(42:10)	DG	#N/A	#N/A	-0.27603	0.28251
DG(42:2)	DG	0.058294	0.883694	0.006492	0.980564
DG(42:3)	DG	-0.12701	0.775067	0.037692	0.864429
DG(42:4)	DG	-0.14717	0.701139	-0.04169	0.84187
DG(42:5)	DG	-0.03791	0.89966	-0.04121	0.835077
DG(42:6)	DG	0.013707	0.94461	0.008204	0.964741
DG(42:7)	DG	0.281388	0.415403	0.06794	0.731332
DG(42:8)	DG	0.139003	0.735451	-0.02613	0.907206
DG(42:9)	DG	#N/A	#N/A	-0.09238	0.709969
DG(43:0)	DG	0.401859	0.16208	0.07126	0.631553
DG(43:1)	DG	0.197011	0.405582	0.114456	0.639139
DG(43:2)	DG	0.136756	0.650284	0.05224	0.831521

Lipid	LipidClass	Log2FC_noH	PVal_noH	Log2FC_withH	PVal_withH
DG(43:3)	DG	-0.00508	0.990947	0.302941	0.290856
DG(43:4)	DG	#N/A	#N/A	-0.0373	0.91398
DG(43:5)	DG	#N/A	#N/A	0.374626	0.217585
DG(43:6)	DG	#N/A	#N/A	0.348331	0.348866
DG(43:7)	DG	#N/A	#N/A	0.001252	0.997333
DG(44:0)	DG	0.25741	0.142879	0.216661	0.158218
DG(44:1)	DG	0.215805	0.334908	0.19481	0.48266
DG(44:10)	DG	#N/A	#N/A	0.091479	0.88374
DG(44:12)	DG	#N/A	#N/A	-0.10175	0.586327
DG(44:2)	DG	0.057059	0.881448	0.108487	0.668631
DG(44:3)	DG	0.040282	0.906931	-0.01814	0.932874
DG(44:4)	DG	0.081347	0.848522	0.023738	0.916849
DG(44:5)	DG	-0.08524	0.84797	-0.03031	0.895238
DG(44:6)	DG	0.293259	0.537395	0.156338	0.484703
DG(44:7)	DG	0.124853	0.753231	0.093954	0.668181
DG(44:9)	DG	#N/A	#N/A	-0.03552	0.872691
DG(45:0)	DG	#N/A	#N/A	0.112716	0.753111
DG(45:4)	DG	#N/A	#N/A	0.574557	0.193715
DG(46:1)	DG	0.03838	0.889367	#N/A	#N/A
DG(46:2)	DG	0.053065	0.886933	0.091015	0.691933
DG(46:3)	DG	-0.11789	0.790866	0.247023	0.45652
DG(46:4)	DG	0.024766	0.95219	0.128389	0.673224
DG(46:5)	DG	0.009716	0.981186	0.05059	0.818152
DG(46:6)	DG	-0.18815	0.73397	-0.30583	0.307428
DG(46:7)	DG	#N/A	#N/A	0.140539	0.610401
DG(47:5)	DG	#N/A	#N/A	0.232084	0.621487
DG(48:4)	DG	#N/A	#N/A	0.236738	0.526791
DG(48:5)	DG	-0.14972	0.767688	0.15331	0.620925
DG(48:6)	DG	0.201108	0.699966	-0.00153	0.995806
DG(O-28:0)	DG[O]	#N/A	#N/A	0.215146	0.28598
DG(O-30:0)	DG[O]	0.13268	0.823191	-0.0872	0.754484
DG(O-30:1)	DG[O]	#N/A	#N/A	0.213286	0.490409
DG(O-32:0)	DG[O]	0.024821	0.973423	0.154133	0.576015
DG(O-32:1)	DG[O]	0.054295	0.935744	#N/A	#N/A
DG(O-32:1)_RT_13.136	DG[O]	#N/A	#N/A	-0.09115	0.701495
DG(O-32:1)_RT_13.458	DG[O]	#N/A	#N/A	0.320273	0.374152
DG(O-32:2)	DG[O]	#N/A	#N/A	0.237409	0.398136
DG(O-33:0)	DG[O]	0.359929	0.429726	0.260877	0.25609
DG(O-33:1)	DG[O]	0.259297	0.527627	#N/A	#N/A
DG(O-33:1)_RT_13.368	DG[O]	#N/A	#N/A	0.164944	0.449216
DG(O-33:1)_RT_13.654	DG[O]	#N/A	#N/A	0.103233	0.722921
DG(O-33:2)	DG[O]	#N/A	#N/A	0.309952	0.240025
DG(O-34:0)	DG[O]	0.294817	0.602143	0.138587	0.509556
DG(O-34:1)	DG[O]	-0.07429	0.92019	#N/A	#N/A
DG(O-34:1)_RT_13.582	DG[O]	#N/A	#N/A	0.139431	0.379227
DG(O-34:1)_RT_13.905	DG[O]	#N/A	#N/A	0.403604	0.330534
DG(O-34:2)	DG[O]	-0.1221	0.87757	#N/A	#N/A

Lipid	LipidClass	Log2FC_noH	PVal_noH	Log2FC_withH	PVal_withH
DG(O-34:2)_RT_13.261	DG[O]	#N/A	#N/A	0.05211	0.80416
DG(O-34:2)_RT_13.565	DG[O]	#N/A	#N/A	0.219333	0.434211
DG(O-34:3)	DG[O]	#N/A	#N/A	0.22643	0.50835
DG(O-35:0)	DG[O]	0.278956	0.459496	0.113349	0.685981
DG(O-35:1)	DG[O]	0.16479	0.727447	#N/A	#N/A
DG(O-35:1)_RT_13.761	DG[O]	#N/A	#N/A	0.283705	0.168027
DG(O-35:1)_RT_14.048	DG[O]	#N/A	#N/A	0.277205	0.273428
DG(O-35:2)	DG[O]	0.08926	0.92056	#N/A	#N/A
DG(O-35:2)_RT_13.44	DG[O]	#N/A	#N/A	-0.05502	0.865153
DG(O-35:2)_RT_13.743	DG[O]	#N/A	#N/A	0.343891	0.145216
DG(O-35:3)	DG[O]	#N/A	#N/A	0.356154	0.326207
DG(O-35:4)	DG[O]	#N/A	#N/A	0.067912	0.826256
DG(O-35:5)	DG[O]	#N/A	#N/A	0.145554	0.473974
DG(O-36:0)	DG[O]	0.314858	0.324925	-0.05847	0.817065
DG(O-36:1)	DG[O]	-0.18857	0.809775	#N/A	#N/A
DG(O-36:1)_RT_13.994	DG[O]	#N/A	#N/A	-0.09839	0.71116
DG(O-36:1)_RT_14.281	DG[O]	#N/A	#N/A	0.338925	0.281782
DG(O-36:2)	DG[O]	-0.43717	0.672052	#N/A	#N/A
DG(O-36:2)_RT_13.69	DG[O]	#N/A	#N/A	0.105717	0.599956
DG(O-36:2)_RT_13.976	DG[O]	#N/A	#N/A	0.320704	0.32862
DG(O-36:3)_RT_13.368	DG[O]	#N/A	#N/A	0.187825	0.616438
DG(O-36:3)_RT_13.672	DG[O]	#N/A	#N/A	0.257918	0.37444
DG(O-36:4)	DG[O]	0.192735	0.669358	0.109374	0.600188
DG(O-37:0)	DG[O]	0.460601	0.147333	0.037323	0.843666
DG(O-37:1)	DG[O]	-0.11407	0.817525	#N/A	#N/A
DG(O-37:1)_RT_14.227	DG[O]	#N/A	#N/A	0.008386	0.96392
DG(O-37:1)_RT_14.477	DG[O]	#N/A	#N/A	0.607499	0.220905
DG(O-37:2)	DG[O]	-0.85416	0.398906	0.366231	0.511361
DG(O-37:3)	DG[O]	-0.08108	0.941553	0.392975	0.443793
DG(O-37:4)	DG[O]	#N/A	#N/A	0.736957	0.06549
DG(O-37:5)	DG[O]	#N/A	#N/A	-0.05241	0.840787
DG(O-38:0)	DG[O]	0.313682	0.288875	0.305283	0.190145
DG(O-38:1)	DG[O]	-0.3851	0.66712	#N/A	#N/A
DG(O-38:1)_RT_14.352	DG[O]	#N/A	#N/A	0.348418	0.332813
DG(O-38:1)_RT_14.604	DG[O]	#N/A	#N/A	0.212162	0.420237
DG(O-38:2)	DG[O]	-0.96592	0.485288	0.221964	0.478614
DG(O-38:3)	DG[O]	-2.02154	0.398129	0.08324	0.826749
DG(O-38:4)	DG[O]	-0.40493	0.597701	0.261706	0.332238
DG(O-38:5)	DG[O]	0.065408	0.903276	0.186817	0.394025
DG(O-39:0)	DG[O]	0.367626	0.157967	0.534956	0.067136
DG(O-39:1)	DG[O]	0.142937	0.628475	0.216482	0.382495
DG(O-39:2)	DG[O]	#N/A	#N/A	0.849993	0.067335
DG(O-39:4)	DG[O]	-0.1333	0.828869	0.181839	0.65493
DG(O-39:5)	DG[O]	0.138151	0.758402	0.25244	0.336442
DG(O-39:7)	DG[O]	0.232503	0.322403	#N/A	#N/A
DG(O-40:0)	DG[O]	0.610585	0.218157	0.32235	0.328452
DG(O-40:1)	DG[O]	0.121591	0.833825	0.359423	0.374818

Lipid	LipidClass	Log2FC_noH	PVal_noH	Log2FC_withH	PVal_withH
DG(O-40:2)	DG[O]	-0.21749	0.774277	#N/A	#N/A
DG(O-40:2)_RT_14.424	DG[O]	#N/A	#N/A	0.158021	0.660971
DG(O-40:2)_RT_14.639	DG[O]	#N/A	#N/A	0.289701	0.499065
DG(O-40:3)	DG[O]	-0.69718	0.399803	0.295616	0.463547
DG(O-40:4)	DG[O]	-0.28576	0.657947	0.289634	0.300326
DG(O-40:5)	DG[O]	0.118777	0.813753	0.15701	0.441542
DG(O-40:6)	DG[O]	-0.14646	0.811953	0.337039	0.129835
DG(O-40:7)	DG[O]	-0.12955	0.700694	#N/A	#N/A
DG(O-41:1)	DG[O]	0.322709	0.41714	0.027158	0.941866
DG(O-41:2)	DG[O]	0.235999	0.505997	0.440615	0.194137
DG(O-41:3)	DG[O]	-0.08309	0.865852	0.714061	0.238081
DG(O-41:4)	DG[O]	#N/A	#N/A	0.206894	0.596217
DG(O-41:5)	DG[O]	#N/A	#N/A	0.519718	0.211538
DG(O-41:6)	DG[O]	#N/A	#N/A	0.488239	0.338589
DG(O-41:7)	DG[O]	0.409338	0.089204	#N/A	#N/A
DG(O-41:8)	DG[O]	0.231032	0.434868	#N/A	#N/A
DG(O-42:1)	DG[O]	0.21563	0.641033	0.291834	0.256566
DG(O-42:2)	DG[O]	-0.0558	0.922757	#N/A	#N/A
DG(O-42:2)_RT_14.746	DG[O]	#N/A	#N/A	0.345393	0.367717
DG(O-42:2)_RT_14.961	DG[O]	#N/A	#N/A	0.365817	0.458238
DG(O-42:3)	DG[O]	-0.11479	0.808961	0.345375	0.370596
DG(O-42:4)	DG[O]	-0.03426	0.933215	0.026678	0.910733
DG(O-42:5)	DG[O]	-0.10119	0.793878	0.005558	0.984141
DG(O-42:6)	DG[O]	0.269412	0.40516	0.818943	0.192226
DG(O-42:7)	DG[O]	0.157345	0.823598	#N/A	#N/A
DG(O-43:1)	DG[O]	#N/A	#N/A	0.268851	0.465485
DG(O-43:2)	DG[O]	#N/A	#N/A	0.387811	0.234985
DG(O-43:3)	DG[O]	#N/A	#N/A	0.334483	0.262421
DG(O-43:4)	DG[O]	#N/A	#N/A	0.145555	0.597128
DG(O-43:5)	DG[O]	#N/A	#N/A	0.528325	0.089786
DG(O-43:6)	DG[O]	#N/A	#N/A	0.299672	0.450457
DG(O-44:10)_RT_13.449	DG[O]	#N/A	#N/A	-0.15674	0.515737
DG(O-44:10)_RT_13.735	DG[O]	#N/A	#N/A	-0.24436	0.286665
DG(O-44:2)	DG[O]	0.335008	0.340621	0.126463	0.81056
DG(O-44:3)	DG[O]	#N/A	#N/A	0.270542	0.575048
DG(O-44:4)	DG[O]	-0.24372	0.647011	0.243246	0.439637
DG(O-44:5)	DG[O]	0.124616	0.640649	0.229828	0.35212
DG(O-44:6)	DG[O]	0.37779	0.172596	0.020651	0.937107
DG(O-44:7)	DG[O]	-0.03354	0.946017	0.546852	0.394232
DG(O-44:9)_RT_13.779	DG[O]	#N/A	#N/A	0.085924	0.818845
DG(O-44:9)_RT_14.012	DG[O]	#N/A	#N/A	0.052231	0.837858
DG(O-45:2)	DG[O]	#N/A	#N/A	0.099887	0.7457
DG(O-45:3)	DG[O]	#N/A	#N/A	0.176369	0.390916
DG(O-45:5)	DG[O]	#N/A	#N/A	0.175604	0.534503
DG(O-45:6)	DG[O]	#N/A	#N/A	0.356214	0.43399
DG(O-46:3)	DG[O]	#N/A	#N/A	0.200757	0.400024
DG(O-46:5)	DG[O]	#N/A	#N/A	0.33214	0.564797

Lipid	LipidClass	Log2FC_noH	PVal_noH	Log2FC_withH	PVal_withH
DG(O-46:6)	DG[O]	#N/A	#N/A	0.611277	0.290967
DG(O-46:7)	DG[O]	0.262443	0.61996	0.099514	0.800348
DG(O-46:9)	DG[O]	-0.26216	0.339419	#N/A	#N/A
DG(O-47:3)	DG[O]	#N/A	#N/A	0.185665	0.602791
DG(O-47:4)	DG[O]	#N/A	#N/A	0.108994	0.619323
DLCL(34:2)	DLCL	-0.46371	0.39551	-0.49905	0.1717
DLCL(34:3)	DLCL	#N/A	#N/A	-0.15449	0.793144
DLCL(36:2)	DLCL	-0.32653	0.505527	-0.18279	0.666906
DLCL(36:3)	DLCL	0.515609	0.435675	-0.45166	0.150203
DLCL(36:4)	DLCL	#N/A	#N/A	-0.78802	0.049422
Hex2Cer(d32:1)	Hex2Cer	0.458956	0.146151	-0.0045	0.985968
Hex2Cer(d33:1)	Hex2Cer	0.834613	0.029181	-0.02777	0.909085
Hex2Cer(d34:0)	Hex2Cer	#N/A	#N/A	-0.04495	0.850047
Hex2Cer(d34:1)	Hex2Cer	0.562751	0.015278	-0.03894	0.870703
Hex2Cer(d34:2)	Hex2Cer	#N/A	#N/A	-0.08794	0.719805
Hex2Cer(d35:1)	Hex2Cer	#N/A	#N/A	0.059976	0.836451
Hex2Cer(d36:1)	Hex2Cer	0.589739	0.00713	0.090579	0.768038
Hex2Cer(d38:1)	Hex2Cer	#N/A	#N/A	0.036562	0.960669
Hex2Cer(d40:1)	Hex2Cer	0.499389	0.025169	0.055021	0.820048
Hex2Cer(d40:2)	Hex2Cer	0.563127	0.086354	0.127924	0.701806
Hex2Cer(d41:1)	Hex2Cer	0.675517	0.015333	0.072904	0.721435
Hex2Cer(d41:2)	Hex2Cer	0.420136	0.080612	-0.00398	0.987184
Hex2Cer(d42:1)	Hex2Cer	0.609049	0.014898	0.101826	0.64424
Hex2Cer(d42:2)	Hex2Cer	0.387112	0.067591	-0.13937	0.603919
Hex2Cer(d42:3)	Hex2Cer	0.57642	0.015883	-0.14536	0.561165
Hex2Cer(d43:1)	Hex2Cer	0.72115	0.021865	0.078851	0.636232
Hex2Cer(d43:2)	Hex2Cer	0.548542	0.068113	-0.09987	0.623744
Hex2Cer(d44:1)	Hex2Cer	1.011162	0.001261	0.045191	0.835226
Hex2Cer(d44:2)	Hex2Cer	0.364567	0.295344	-0.13154	0.518177
Hex2Cer(d44:3)	Hex2Cer	0.559035	0.1591	-0.33794	0.231905
Hex2Cer(t34:1)	Hex2Cer	0.652119	0.188616	#N/A	#N/A
HexCer(d30:0)	HexCer	#N/A	#N/A	-0.18371	0.560088
HexCer(d31:1)	HexCer	#N/A	#N/A	0.588042	0.091497
HexCer(d31:2)	HexCer	#N/A	#N/A	1.11972	0.407308
HexCer(d32:1)	HexCer	0.731347	0.098189	0.545184	0.112974
HexCer(d32:2)	HexCer	#N/A	#N/A	0.154744	0.666153
HexCer(d33:1)	HexCer	0.306264	0.424824	0.644881	0.120984
HexCer(d34:0)	HexCer	#N/A	#N/A	0.228148	0.572472
HexCer(d34:1)	HexCer	0.587468	0.104722	0.461035	0.220556
HexCer(d34:2)	HexCer	1.163454	0.019827	0.586887	0.151615
HexCer(d35:1)	HexCer	#N/A	#N/A	0.546431	0.082602
HexCer(d36:1)	HexCer	0.85576	0.067827	0.425201	0.220674
HexCer(d38:1)	HexCer	0.477954	0.14198	0.597849	0.236905
HexCer(d39:1)	HexCer	0.586084	0.069333	0.419069	0.321252
HexCer(d40:0)	HexCer	0.898898	0.105561	0.257376	0.332881
HexCer(d40:1)	HexCer	0.483037	0.094136	0.442948	0.108211
HexCer(d40:2)	HexCer	0.385159	0.344771	0.46832	0.136747

Lipid	LipidClass	Log2FC_noH	PVal_noH	Log2FC_withH	PVal_withH
HexCer(d41:0)	HexCer	#N/A	#N/A	0.015155	0.962968
HexCer(d41:1)	HexCer	0.48053	0.087299	0.475648	0.055555
HexCer(d41:2)	HexCer	0.451527	0.192027	0.206883	0.646539
HexCer(d42:0)	HexCer	0.736948	0.220729	0.276409	0.438263
HexCer(d42:1)	HexCer	0.438674	0.080622	0.487889	0.071824
HexCer(d42:2)	HexCer	0.285518	0.419879	0.355574	0.248375
HexCer(d42:3)	HexCer	0.648216	0.050205	0.26837	0.444188
HexCer(d42:4)	HexCer	0.978062	0.028542	0.321928	0.360707
HexCer(d43:1)	HexCer	0.367825	0.103727	0.432719	0.050845
HexCer(d43:2)	HexCer	0.229791	0.435218	0.32568	0.207134
HexCer(d43:3)	HexCer	0.773109	0.11624	0.171007	0.611485
HexCer(d44:1)	HexCer	0.37961	0.140391	0.4955	0.041935
HexCer(d44:2)	HexCer	0.091904	0.832127	0.274346	0.27872
HexCer(d44:3)	HexCer	0.27796	0.478417	0.201774	0.476759
HexCer(d44:4)	HexCer	#N/A	#N/A	0.408198	0.396576
HexCer(d45:1)	HexCer	#N/A	#N/A	0.607839	0.003617
HexCer(d45:2)	HexCer	#N/A	#N/A	0.485294	0.194547
HexCer(d46:1)	HexCer	#N/A	#N/A	0.861916	0.01449
HexCer(d46:2)	HexCer	#N/A	#N/A	0.506064	0.121473
HexCer(d47:2)	HexCer	#N/A	#N/A	1.658055	0.335222
LPA(16:0)	LPA	#N/A	#N/A	1.074566	0.325457
LPA(18:0)	LPA	-0.03585	0.90799	0.527706	0.506291
LPA(18:1)	LPA	#N/A	#N/A	0.12912	0.488143
LPA(18:2)	LPA	#N/A	#N/A	-0.32517	0.516169
LPA(20:4)	LPA	#N/A	#N/A	-0.30167	0.343873
LPA(22:6)	LPA	#N/A	#N/A	0.586621	0.275305
LPC(O-12:0)	LPC[O]	#N/A	#N/A	0.091159	0.741317
LPC(O-12:1)	LPC[O]	#N/A	#N/A	0.152886	0.35708
LPC(O-13:0)	LPC[O]	#N/A	#N/A	0.130975	0.590646
LPC(O-13:1)	LPC[O]	#N/A	#N/A	0.437312	0.185435
LPC(O-14:0)	LPC[O]	0.121875	0.508278	0.212714	0.118362
LPC(O-14:1)	LPC[O]	#N/A	#N/A	0.084509	0.569013
LPC(O-15:0)	LPC[O]	0.160282	0.618246	0.112098	0.439063
LPC(O-15:1)	LPC[O]	#N/A	#N/A	0.112935	0.378411
LPC(O-16:0)	LPC[O]	-0.00176	0.997106	0.043792	0.811824
LPC(O-16:1)	LPC[O]	0.097674	0.774258	0.326343	0.127658
LPC(O-16:2)	LPC[O]	#N/A	#N/A	0.149064	0.434602
LPC(O-17:0)	LPC[O]	0.373077	0.048742	0.199589	0.349606
LPC(O-17:1)	LPC[O]	0.152912	0.348058	0.221058	0.136219
LPC(O-17:2)	LPC[O]	#N/A	#N/A	0.192041	0.284483
LPC(O-18:0)	LPC[O]	0.208642	0.296941	0.08901	0.50111
LPC(O-18:1)	LPC[O]	-0.08803	0.767556	0.064983	0.622756
LPC(O-18:2)	LPC[O]	-0.0089	0.981693	0.253233	0.20732
LPC(O-18:3)	LPC[O]	#N/A	#N/A	-0.02002	0.944237
LPC(O-19:0)	LPC[O]	0.21549	0.169777	-0.50493	0.141999
LPC(O-19:1)	LPC[O]	-0.0415	0.911579	0.000781	0.994842
LPC(O-19:2)	LPC[O]	#N/A	#N/A	0.540625	0.080469

Lipid	LipidClass	Log2FC_noH	PVal_noH	Log2FC_withH	PVal_withH
LPC(O-20:0)	LPC[O]	0.170875	0.23468	0.197287	0.421032
LPC(O-20:1)	LPC[O]	-0.05432	0.848249	0.066552	0.643131
LPC(O-20:2)	LPC[O]	0.008129	0.97912	0.173865	0.38519
LPC(O-20:3)	LPC[O]	#N/A	#N/A	0.251303	0.297714
LPC(O-21:0)	LPC[O]	0.201013	0.248179	0.492093	0.022396
LPC(O-21:1)	LPC[O]	#N/A	#N/A	0.305162	0.016445
LPC(O-21:2)	LPC[O]	#N/A	#N/A	0.612317	0.046542
LPC(O-22:0)	LPC[O]	0.18856	0.14406	0.087915	0.463966
LPC(O-22:1)	LPC[O]	0.036505	0.858823	0.171388	0.095836
LPC(O-22:2)	LPC[O]	0.00228	0.993695	0.232099	0.171392
LPC(O-22:3)	LPC[O]	#N/A	#N/A	-0.49429	0.196753
LPC(O-23:0)	LPC[O]	0.179424	0.125612	0.03611	0.665561
LPC(O-23:1)	LPC[O]	0.766884	0.066935	-0.03726	0.807345
LPC(O-23:2)	LPC[O]	#N/A	#N/A	0.420309	0.210298
LPC(O-24:0)	LPC[O]	0.210705	0.199623	0.040814	0.733467
LPC(O-24:1)	LPC[O]	0.149663	0.561582	0.037597	0.803613
LPC(O-24:2)	LPC[O]	#N/A	#N/A	0.34505	0.098818
LPC(O-25:0)	LPC[O]	#N/A	#N/A	0.144098	0.451723
LPC(O-25:1)	LPC[O]	-0.25061	0.597615	#N/A	#N/A
LPC(O-26:0)	LPC[O]	#N/A	#N/A	0.021028	0.933758
LPC(O-26:1)	LPC[O]	0.157678	0.582306	0.008472	0.965431
LPC(O-26:2)	LPC[O]	0.272029	0.534759	-0.12429	0.65492
LPE(12:0)	LPE	#N/A	#N/A	0.245137	0.541047
LPE(14:1)	LPE	0.316617	0.518718	#N/A	#N/A
LPE(15:0)	LPE	0.234677	0.187999	-0.09784	0.740817
LPE(15:1)	LPE	-0.21485	0.607222	#N/A	#N/A
LPE(16:0)	LPE	0.125765	0.513303	-0.25277	0.208328
LPE(16:1)	LPE	0.100803	0.790432	-0.12123	0.671223
LPE(16:2)	LPE	-0.05668	0.881408	#N/A	#N/A
LPE(17:0)	LPE	0.309823	0.109134	-0.14551	0.435254
LPE(17:1)	LPE	0.235321	0.329408	-0.26585	0.54451
LPE(18:0)	LPE	0.154721	0.420743	-0.24946	0.221563
LPE(18:1)	LPE	0.093478	0.726511	-0.04651	0.835534
LPE(18:2)	LPE	0.26898	0.162954	-0.24504	0.317143
LPE(18:3)	LPE	0.072184	0.88864	#N/A	#N/A
LPE(19:0)	LPE	0.212198	0.469951	-0.19107	0.639439
LPE(19:1)	LPE	-0.06627	0.804768	#N/A	#N/A
LPE(20:0)	LPE	0.005792	0.988684	0.007826	0.973656
LPE(20:1)	LPE	-0.24893	0.481028	-0.34807	0.163958
LPE(20:2)	LPE	-0.123	0.72194	0.219911	0.51987
LPE(20:3)	LPE	-0.15436	0.658881	0.002994	0.993491
LPE(20:4)	LPE	0.045891	0.866816	-0.05444	0.848228
LPE(20:5)	LPE	0.565107	0.107465	-0.08672	0.805585
LPE(21:3)	LPE	-0.97374	0.064107	-0.07384	0.884908
LPE(22:0)	LPE	0.168868	0.451455	-0.04899	0.781477
LPE(22:1)	LPE	-0.54001	0.298604	-0.28173	0.237002
LPE(22:2)	LPE	-0.75033	0.234873	-0.13918	0.617215

Lipid	LipidClass	Log2FC_noH	PVal_noH	Log2FC_withH	PVal_withH
LPE(22:3)	LPE	-0.80681	0.202423	-0.20561	0.485381
LPE(22:4)	LPE	-0.30192	0.390918	-0.231	0.474879
LPE(22:5)	LPE	-0.07762	0.766191	-0.18738	0.524334
LPE(22:6)	LPE	0.083578	0.689509	-0.22431	0.415451
LPE(23:0)	LPE	#N/A	#N/A	0.23516	0.240615
LPE(24:0)	LPE	0.129966	0.617555	-0.05863	0.783827
LPE(24:1)	LPE	-0.29722	0.460067	-0.25161	0.234939
LPE(24:2)	LPE	-0.68203	0.244691	-0.45524	0.05858
LPE(24:3)	LPE	-0.68267	0.167468	-0.113	0.775462
LPE(24:4)	LPE	-0.00467	0.987593	-0.26245	0.29852
LPE(24:5)	LPE	-0.14956	0.585859	-0.30295	0.414193
LPE(24:6)	LPE	-0.41732	0.36459	-0.86476	0.220309
LPE(25:0)	LPE	#N/A	#N/A	0.075977	0.836227
LPE(25:1)	LPE	#N/A	#N/A	-0.34357	0.406564
LPE(26:0)	LPE	#N/A	#N/A	0.000309	0.999296
LPE(26:1)	LPE	-0.08569	0.880213	-0.32279	0.263292
LPE(26:2)	LPE	-0.39793	0.405209	-0.76436	0.029763
LPE(O-10:1)	LPE[O]	#N/A	#N/A	-0.08017	0.854149
LPE(O-11:1)	LPE[O]	#N/A	#N/A	0.531214	0.165336
LPE(O-12:1)	LPE[O]	#N/A	#N/A	-0.04913	0.780647
LPE(O-13:1)	LPE[O]	#N/A	#N/A	0.344133	0.417318
LPE(O-14:1)	LPE[O]	#N/A	#N/A	0.050276	0.849154
LPE(O-15:0)	LPE[O]	0.794825	0.028946	#N/A	#N/A
LPE(O-15:1)	LPE[O]	#N/A	#N/A	-0.11665	0.609287
LPE(O-16:0)	LPE[O]	0.077411	0.740829	-0.28032	0.328604
LPE(O-16:1)	LPE[O]	#N/A	#N/A	0.074766	0.730318
LPE(O-16:2)	LPE[O]	#N/A	#N/A	-0.07344	0.760543
LPE(O-17:0)	LPE[O]	0.236992	0.411479	#N/A	#N/A
LPE(O-17:1)	LPE[O]	#N/A	#N/A	0.111244	0.580726
LPE(O-17:2)	LPE[O]	#N/A	#N/A	0.029995	0.856838
LPE(O-18:0)	LPE[O]	0.127172	0.727471	0.348744	0.113076
LPE(O-18:1)	LPE[O]	-0.23783	0.20302	0.165467	0.424082
LPE(O-18:2)	LPE[O]	#N/A	#N/A	0.126554	0.546829
LPE(O-18:3)	LPE[O]	#N/A	#N/A	-0.0175	0.938432
LPE(O-19:1)	LPE[O]	#N/A	#N/A	-0.02872	0.85015
LPE(O-19:2)	LPE[O]	0.03317	0.958145	0.108011	0.633981
LPE(O-20:0)	LPE[O]	0.186406	0.269718	#N/A	#N/A
LPE(O-20:1)	LPE[O]	#N/A	#N/A	0.062425	0.632123
LPE(O-20:2)	LPE[O]	#N/A	#N/A	0.022311	0.918453
LPE(O-20:3)	LPE[O]	#N/A	#N/A	-0.05514	0.767089
LPE(O-21:1)	LPE[O]	#N/A	#N/A	0.009341	0.938919
LPE(O-22:0)	LPE[O]	0.195951	0.253744	0.116457	0.336159
LPE(O-22:1)	LPE[O]	-0.40536	0.06324	0.155667	0.178103
LPE(O-22:2)	LPE[O]	#N/A	#N/A	0.086751	0.710548
LPE(O-24:1)	LPE[O]	0.073727	0.823926	0.179018	0.550986
LPE(O-24:2)	LPE[O]	#N/A	#N/A	0.212451	0.544225
LPG(15:0)	LPG	#N/A	#N/A	0.093636	0.815194

Lipid	LipidClass	Log2FC_noH	PVal_noH	Log2FC_withH	PVal_withH
LPG(16:0)	LPG	-0.14258	0.672955	0.089394	0.691486
LPG(16:1)	LPG	-0.17566	0.673489	0.243585	0.246911
LPG(16:2)	LPG	-0.58644	0.23381	0.223805	0.483294
LPG(17:0)	LPG	0.033092	0.910435	0.210285	0.451644
LPG(17:1)	LPG	-0.21527	0.552443	0.289982	0.223463
LPG(18:0)	LPG	-0.08253	0.807772	0.022815	0.940763
LPG(18:1)	LPG	-0.42775	0.301313	0.260606	0.277772
LPG(18:2)	LPG	-0.17325	0.609817	0.411581	0.044114
LPG(18:3)	LPG	-0.04824	0.877163	0.275414	0.222968
LPG(19:1)	LPG	-0.39188	0.33443	0.272821	0.331547
LPG(19:2)	LPG	-0.21358	0.591588	0.435842	0.075055
LPG(19:3)	LPG	-0.9601	0.140661	0.438003	0.376044
LPG(20:1)	LPG	-0.49078	0.30113	0.318799	0.212091
LPG(20:2)	LPG	-0.55445	0.292162	0.412237	0.07596
LPG(20:3)	LPG	-0.32872	0.411434	0.753454	0.013977
LPG(20:4)	LPG	-0.21653	0.603899	0.737994	0.01153
LPG(20:5)	LPG	0.951026	0.301699	0.667059	0.025507
LPG(21:2)	LPG	-0.46437	0.339656	#N/A	#N/A
LPG(21:3)	LPG	-0.56917	0.283449	0.691652	0.02545
LPG(21:4)	LPG	-1.03252	0.156423	#N/A	#N/A
LPG(22:3)	LPG	-0.14287	0.61827	#N/A	#N/A
LPG(22:4)	LPG	-0.21773	0.596752	0.657192	0.010532
LPG(22:5)	LPG	0.142939	0.672829	0.673691	0.010773
LPG(22:6)	LPG	0.296941	0.368656	0.560466	0.045239
MLCL(50:2)	MLCL	#N/A	#N/A	-0.18037	0.890578
MLCL(51:3)	MLCL	#N/A	#N/A	-0.06522	0.817788
MLCL(52:2)	MLCL	#N/A	#N/A	0.152176	0.446835
MLCL(52:3)	MLCL	#N/A	#N/A	0.467175	0.2532
MLCL(52:4)	MLCL	-0.30619	0.512037	0.306785	0.601843
MLCL(52:5)	MLCL	#N/A	#N/A	-0.36976	0.442636
MLCL(53:3)	MLCL	#N/A	#N/A	-0.053	0.756892
MLCL(53:4)	MLCL	#N/A	#N/A	-0.08911	0.659464
MLCL(54:2)	MLCL	#N/A	#N/A	0.007744	0.967659
MLCL(54:3)	MLCL	#N/A	#N/A	0.171029	0.533647
MLCL(54:4)	MLCL	0.337609	0.227468	-0.95246	0.44202
MLCL(54:5)	MLCL	1.088625	0.038056	-0.04629	0.850017
MLCL(54:6)	MLCL	#N/A	#N/A	-0.2594	0.517656
MLCL(55:3)	MLCL	#N/A	#N/A	-0.18319	0.340121
MLCL(56:7)	MLCL	#N/A	#N/A	-0.01278	0.971691
PA(30:0)	PA	0.223983	0.290876	0.195137	0.341571
PA(30:1)	PA	#N/A	#N/A	0.466447	0.072277
PA(31:0)	PA	#N/A	#N/A	0.231076	0.408377
PA(31:1)	PA	#N/A	#N/A	0.11188	0.720614
PA(32:0)	PA	0.311248	0.046079	0.17426	0.373722
PA(32:1)	PA	0.260422	0.164651	0.28094	0.169095
PA(32:2)	PA	#N/A	#N/A	0.343383	0.059643
PA(33:0)	PA	#N/A	#N/A	0.218238	0.275186

Lipid	LipidClass	Log2FC_noH	PVal_noH	Log2FC_withH	PVal_withH
PA(33:1)	PA	#N/A	#N/A	0.234225	0.347153
PA(33:2)	PA	#N/A	#N/A	0.116663	0.693092
PA(34:0)	PA	#N/A	#N/A	0.072281	0.786162
PA(34:1)	PA	0.27564	0.019566	0.249533	0.265234
PA(34:2)	PA	0.1307	0.514004	0.200193	0.34152
PA(34:3)	PA	#N/A	#N/A	0.419927	0.058164
PA(34:4)	PA	#N/A	#N/A	0.32294	0.321671
PA(35:0)	PA	#N/A	#N/A	-0.22261	0.559432
PA(35:1)	PA	0.449907	0.128545	0.191868	0.327418
PA(35:2)	PA	#N/A	#N/A	0.15953	0.481052
PA(35:3)	PA	#N/A	#N/A	-0.28082	0.321343
PA(36:1)	PA	0.272816	0.08283	0.161963	0.404284
PA(36:2)	PA	0.159542	0.371321	0.05826	0.801114
PA(36:3)	PA	-0.00657	0.975649	0.204067	0.292611
PA(36:4)	PA	0.491464	0.053803	0.198605	0.343305
PA(36:5)	PA	#N/A	#N/A	0.12702	0.603807
PA(37:1)	PA	#N/A	#N/A	0.084181	0.800961
PA(37:2)	PA	#N/A	#N/A	0.003277	0.992723
PA(37:3)	PA	#N/A	#N/A	0.091727	0.762514
PA(37:4)	PA	#N/A	#N/A	0.116386	0.536851
PA(38:1)	PA	#N/A	#N/A	-0.03492	0.905511
PA(38:2)	PA	#N/A	#N/A	-0.1182	0.663033
PA(38:3)	PA	-0.05499	0.879852	0.053733	0.740906
PA(38:4)	PA	0.296148	0.378613	0.147652	0.561964
PA(38:5)	PA	0.286769	0.420472	0.071827	0.662113
PA(38:6)	PA	#N/A	#N/A	0.185656	0.275945
PA(39:3)	PA	#N/A	#N/A	0.09577	0.793117
PA(39:4)	PA	#N/A	#N/A	-0.20106	0.515812
PA(39:5)	PA	#N/A	#N/A	-0.00906	0.97189
PA(40:1)	PA	#N/A	#N/A	-0.13905	0.733693
PA(40:3)	PA	#N/A	#N/A	0.156102	0.554704
PA(40:4)	PA	#N/A	#N/A	0.07916	0.667149
PA(40:5)	PA	#N/A	#N/A	0.073736	0.656513
PA(40:6)	PA	#N/A	#N/A	0.080102	0.655179
PA(40:7)	PA	#N/A	#N/A	0.749494	0.093281
PC(22:0)	PC	#N/A	#N/A	-0.85039	0.086963
PC(23:0)	PC	#N/A	#N/A	-0.91568	0.030187
PC(23:1)	PC	#N/A	#N/A	-0.88208	0.019465
PC(24:0)	PC	#N/A	#N/A	-0.59584	0.069447
PC(24:1)	PC	#N/A	#N/A	-0.73607	0.019768
PC(25:0)	PC	#N/A	#N/A	-0.35703	0.139378
PC(25:1)	PC	#N/A	#N/A	-0.63904	0.07143
PC(26:0)	PC	#N/A	#N/A	0.128964	0.106364
PC(27:0)	PC	0.269645	0.238094	0.01469	0.926493
PC(27:1)	PC	#N/A	#N/A	-0.55711	0.265701
PC(28:1)	PC	0.162075	0.653089	-0.14614	0.424206
PC(28:2)	PC	#N/A	#N/A	-0.59604	0.073104

Lipid	LipidClass	Log2FC_noH	PVal_noH	Log2FC_withH	PVal_withH
PC(29:0)	PC	0.285701	0.024731	-0.08092	0.494516
PC(29:1)	PC	0.237253	0.405207	-0.03096	0.877931
PC(29:2)	PC	0.094801	0.78533	#N/A	#N/A
PC(30:0)	PC	0.036948	0.753896	-0.08008	0.530225
PC(30:1)	PC	0.089238	0.732653	-0.0391	0.858612
PC(30:2)	PC	0.197774	0.640545	0.039135	0.875005
PC(30:3)	PC	0.31467	0.400791	-0.01761	0.968563
PC(31:0)	PC	0.189699	0.259554	-0.01678	0.862554
PC(31:1)	PC	0.18136	0.154014	0.001294	0.993862
PC(31:2)	PC	0.111254	0.660907	0.154599	0.537192
PC(31:3)	PC	0.254388	0.453687	#N/A	#N/A
PC(31:4)	PC	-0.0066	0.982699	#N/A	#N/A
PC(32:0)	PC	-0.04229	0.690262	-0.0547	0.57214
PC(32:1)	PC	0.025045	0.765418	0	1
PC(32:2)	PC	0.093266	0.707139	-0.00883	0.964228
PC(32:3)	PC	0.115294	0.617565	0.098619	0.601467
PC(32:4)	PC	0.314663	0.376616	0.058793	0.785493
PC(33:0)	PC	0.146845	0.366036	0.011843	0.914857
PC(33:1)	PC	0.186893	0.022294	0.056642	0.682366
PC(33:2)	PC	0.172814	0.056779	-0.00395	0.980506
PC(33:3)	PC	0.179213	0.152857	-0.12531	0.523666
PC(33:4)	PC	0.370832	0.186821	#N/A	#N/A
PC(33:5)	PC	0.23255	0.153365	#N/A	#N/A
PC(34:0)	PC	-0.03258	0.814934	0.031441	0.849614
PC(34:1)	PC	0.058437	0.222791	0.059877	0.65386
PC(34:2)	PC	0.045851	0.515482	-0.02219	0.876207
PC(34:3)	PC	0.12174	0.312051	0.024971	0.895036
PC(34:4)	PC	0.203938	0.072611	0.044323	0.758794
PC(34:5)	PC	0.405404	0.104281	-0.06349	0.762218
PC(34:6)	PC	0.483342	0.090957	#N/A	#N/A
PC(35:0)	PC	0.117202	0.382554	0.101316	0.432444
PC(35:1)	PC	0.218649	0.059942	0.129687	0.364916
PC(35:2)	PC	0.157064	0.201685	0.009974	0.932895
PC(35:3)	PC	-0.09868	0.786723	-0.01183	0.929925
PC(35:4)	PC	0.24263	0.123914	-0.01025	0.949741
PC(35:5)	PC	0.432487	0.152049	-0.22339	0.327168
PC(35:7)	PC	0.152449	0.594239	#N/A	#N/A
PC(36:0)	PC	0.049486	0.729133	-0.21465	0.665031
PC(36:1)	PC	0.2061	0.036216	0.200735	0.350492
PC(36:2)	PC	0.028659	0.711205	-0.00058	0.996158
PC(36:3)	PC	0.124987	0.594445	-0.00037	0.998046
PC(36:4)	PC	0.102188	0.317516	-0.05247	0.715912
PC(36:5)	PC	0.205367	0.300928	-0.0803	0.632988
PC(36:6)	PC	0.376996	0.086292	0.015414	0.934396
PC(36:7)	PC	0.325041	0.414556	0.457217	0.163795
PC(37:0)	PC	0.068943	0.599717	#N/A	#N/A
PC(37:1)	PC	0.197127	0.07843	0.148235	0.265334

Lipid	LipidClass	Log2FC_noH	PVal_noH	Log2FC_withH	PVal_withH
PC(37:2)	PC	0.006947	0.958599	0.008523	0.943571
PC(37:3)	PC	-0.01351	0.885057	0.043953	0.779377
PC(37:4)	PC	0.162524	0.149386	0.027945	0.819903
PC(37:5)	PC	0.242299	0.386053	0.017747	0.898338
PC(37:6)	PC	0.354509	0.304438	-0.015	0.974609
PC(37:7)	PC	0.375008	0.075999	#N/A	#N/A
PC(38:0)	PC	-0.02581	0.849397	0.098036	0.720192
PC(38:1)	PC	-0.00029	0.998808	0.115592	0.321923
PC(38:2)	PC	-0.04818	0.833659	0.038239	0.777872
PC(38:3)	PC	0.089347	0.297618	0.027822	0.919546
PC(38:4)	PC	0.101306	0.531133	0.038509	0.770917
PC(38:5)	PC	0.134535	0.1817	-0.00785	0.949463
PC(38:6)	PC	0.218277	0.346024	-0.02272	0.868487
PC(38:7)	PC	0.311326	0.109129	0.037227	0.864498
PC(38:8)	PC	0.481146	0.039032	0.152217	0.505982
PC(39:0)	PC	0.119665	0.342209	#N/A	#N/A
PC(39:1)	PC	-0.00453	0.981222	-0.22233	0.38683
PC(39:2)	PC	0.05692	0.765424	0.170721	0.287462
PC(39:3)	PC	-0.05371	0.757174	0.013944	0.922245
PC(39:4)	PC	-0.02267	0.879503	0.028061	0.813748
PC(39:5)	PC	0.135379	0.279673	0.019883	0.825641
PC(39:6)	PC	0.397889	0.00707	0.057013	0.642125
PC(39:7)	PC	0.271557	0.354875	-0.05936	0.83325
PC(40:0)	PC	0.146263	0.371179	0.27803	0.183403
PC(40:1)	PC	-0.02575	0.918519	0.161126	0.344511
PC(40:10)	PC	#N/A	#N/A	0.116976	0.723731
PC(40:2)	PC	-0.15027	0.650909	-0.13124	0.334955
PC(40:3)	PC	-0.20315	0.441681	-0.03029	0.814811
PC(40:4)	PC	-0.05032	0.703218	0.035909	0.852604
PC(40:5)	PC	0.143076	0.023646	0.067246	0.52865
PC(40:6)	PC	0.273519	0.038398	0.013882	0.886027
PC(40:7)	PC	0.213931	0.046449	0.08649	0.408272
PC(40:8)	PC	0.183818	0.730482	0.185867	0.12952
PC(40:9)	PC	-0.54183	0.678967	0.230151	0.259637
PC(41:0)	PC	0.243155	0.179236	#N/A	#N/A
PC(41:1)	PC	0.107082	0.611404	0.466153	0.31014
PC(41:2)	PC	0.046052	0.858539	-0.1199	0.643329
PC(41:3)	PC	-0.14383	0.592393	0.011607	0.93538
PC(41:4)	PC	-0.01818	0.925165	-0.1249	0.303235
PC(41:5)	PC	0.030647	0.764183	0.006336	0.948786
PC(41:6)	PC	0.16009	0.38354	-0.03546	0.732846
PC(41:7)	PC	0.074386	0.568832	0.031188	0.845553
PC(41:8)	PC	#N/A	#N/A	0.082085	0.492712
PC(41:9)	PC	#N/A	#N/A	0.081773	0.748104
PC(42:0)	PC	0.241468	0.287339	#N/A	#N/A
PC(42:1)	PC	0.148501	0.517499	0.15922	0.599445
PC(42:10)	PC	-0.05791	0.918735	0.130206	0.446025

Lipid	LipidClass	Log2FC_noH	PVal_noH	Log2FC_withH	PVal_withH
PC(42:11)	PC	#N/A	#N/A	0.063856	0.788002
PC(42:2)	PC	-2.8E-05	0.999928	0.027992	0.860952
PC(42:3)	PC	-0.11126	0.708334	-0.011	0.932989
PC(42:4)	PC	-0.06409	0.773006	-0.01046	0.937474
PC(42:5)	PC	0.040265	0.756334	-0.06936	0.455
PC(42:6)	PC	-0.00552	0.970422	-0.10182	0.297471
PC(42:7)	PC	0.046053	0.62522	0.081546	0.502831
PC(42:8)	PC	0.256426	0.306413	0.214664	0.180952
PC(42:9)	PC	0.327838	0.504428	0.233102	0.132276
PC(43:0)	PC	0.344877	0.207852	#N/A	#N/A
PC(43:1)	PC	0.286932	0.239387	#N/A	#N/A
PC(43:2)	PC	0.168842	0.566118	-0.14638	0.612459
PC(43:3)	PC	-0.01455	0.960762	0.045774	0.86331
PC(43:4)	PC	0.089907	0.77147	-0.0622	0.675234
PC(43:5)	PC	-0.01688	0.932549	-0.1007	0.614296
PC(43:6)	PC	-0.08203	0.673837	-0.04927	0.728882
PC(43:7)	PC	-0.11307	0.567931	0.069566	0.756559
PC(43:8)	PC	#N/A	#N/A	0.066742	0.695612
PC(43:9)	PC	#N/A	#N/A	0.301941	0.177976
PC(44:0)	PC	0.295301	0.266498	#N/A	#N/A
PC(44:1)	PC	0.355928	0.175594	0.22539	0.509598
PC(44:10)	PC	#N/A	#N/A	0.179177	0.265339
PC(44:11)	PC	#N/A	#N/A	0.1902	0.176779
PC(44:12)	PC	#N/A	#N/A	0.024946	0.843691
PC(44:2)	PC	0.15825	0.631431	0.069099	0.770039
PC(44:3)	PC	-0.03597	0.917101	-0.09954	0.613967
PC(44:4)	PC	0.015689	0.957767	0.118527	0.501805
PC(44:5)	PC	-0.09442	0.732491	0.056654	0.763127
PC(44:6)	PC	0.079441	0.683951	-0.04034	0.778166
PC(44:7)	PC	-0.05985	0.762386	0.052379	0.77652
PC(44:8)	PC	0.053475	0.792873	0.090729	0.698715
PC(44:9)	PC	0.008328	0.987968	0.081579	0.667198
PC(45:1)	PC	0.254243	0.373768	#N/A	#N/A
PC(45:2)	PC	0.307451	0.40512	#N/A	#N/A
PC(45:3)	PC	0.14691	0.676545	#N/A	#N/A
PC(45:4)	PC	-0.07127	0.844356	0.128802	0.544528
PC(45:5)	PC	-0.04173	0.897411	0.026592	0.91124
PC(45:6)	PC	0.007387	0.982049	-0.10484	0.607527
PC(45:7)	PC	0.549443	0.284993	0.029073	0.918207
PC(45:8)	PC	#N/A	#N/A	0.240935	0.397558
PC(45:9)	PC	#N/A	#N/A	0.296177	0.446024
PC(46:0)	PC	0.398889	0.332263	#N/A	#N/A
PC(46:1)	PC	0.414988	0.235725	#N/A	#N/A
PC(46:10)	PC	0.318903	0.630705	0.059479	0.751944
PC(46:11)	PC	#N/A	#N/A	-0.03375	0.857132
PC(46:12)	PC	#N/A	#N/A	0.035672	0.9107
PC(46:2)	PC	0.308604	0.457942	-0.1761	0.587198

Lipid	LipidClass	Log2FC_noH	PVal_noH	Log2FC_withH	PVal_withH
PC(46:3)	PC	0.015303	0.968103	-0.00922	0.976874
PC(46:4)	PC	0.071914	0.861856	0.135419	0.579839
PC(46:5)	PC	0.013938	0.965914	0.044981	0.834339
PC(46:6)	PC	-0.04429	0.886338	-0.05387	0.746171
PC(46:7)	PC	0.035191	0.895953	0.054476	0.793623
PC(46:8)	PC	0.128914	0.585592	0.037787	0.870052
PC(46:9)	PC	0.178487	0.502573	0.096505	0.67383
PC(47:2)	PC	1.124545	0.0584	#N/A	#N/A
PC(47:3)	PC	0.26379	0.649284	#N/A	#N/A
PC(47:4)	PC	0.224965	0.630141	#N/A	#N/A
PC(47:5)	PC	0.038776	0.893093	-0.00243	0.993206
PC(47:6)	PC	0.207337	0.561075	-0.27942	0.237031
PC(47:7)	PC	0.733575	0.317946	0.090722	0.757067
PC(47:8)	PC	#N/A	#N/A	-0.74955	0.076674
PC(48:10)	PC	0.50231	0.227433	-0.01892	0.94025
PC(48:11)	PC	#N/A	#N/A	0.032686	0.905593
PC(48:12)	PC	#N/A	#N/A	-0.08854	0.831599
PC(48:2)	PC	0.066797	0.837811	#N/A	#N/A
PC(48:3)	PC	0.225676	0.485319	#N/A	#N/A
PC(48:4)	PC	0.180483	0.650476	0.069309	0.82985
PC(48:5)	PC	0.169784	0.731987	0.054941	0.788857
PC(48:6)	PC	0.355388	0.311323	0.003996	0.984545
PC(48:7)	PC	0.385011	0.368019	-0.06799	0.76161
PC(48:8)	PC	0.185498	0.58663	0.03896	0.863062
PC(48:9)	PC	0.39598	0.226785	0.053109	0.844299
PC(49:6)	PC	#N/A	#N/A	-1.49187	0.032183
PC(49:7)	PC	#N/A	#N/A	-2.00994	0.008964
PC(50:10)	PC	0.665165	0.14804	0.05213	0.843662
PC(50:11)	PC	#N/A	#N/A	0.190622	0.697408
PC(50:4)	PC	0.40492	0.268801	#N/A	#N/A
PC(50:5)	PC	0.100117	0.787105	#N/A	#N/A
PC(50:6)	PC	0.351767	0.326235	0.285915	0.398006
PC(50:7)	PC	0.419147	0.161433	0.024755	0.94018
PC(50:8)	PC	0.311451	0.361571	0.014418	0.957375
PC(50:9)	PC	0.682244	0.167978	0.1543	0.645688
PC(52:10)	PC	0.4189	0.309409	-0.17761	0.715266
PC(52:5)	PC	0.27688	0.657821	#N/A	#N/A
PC(52:6)	PC	0.206082	0.627507	#N/A	#N/A
PC(52:7)	PC	0.268542	0.506131	#N/A	#N/A
PC(52:8)	PC	0.30079	0.448768	#N/A	#N/A
PC(52:9)	PC	0.546781	0.238497	0.453347	0.213109
PC(54:10)	PC	0.530477	0.339589	#N/A	#N/A
PC(54:9)	PC	0.398747	0.481857	#N/A	#N/A
PC(O-25:2)	PC[O]	#N/A	#N/A	0.877006	0.402218
PC(O-26:0)	PC[O]	0.075801	0.540822	#N/A	#N/A
PC(O-26:1)	PC[O]	#N/A	#N/A	-0.29517	0.419938
PC(O-27:0)	PC[O]	0.165244	0.303374	0.150359	0.541775

Lipid	LipidClass	Log2FC_noH	PVal_noH	Log2FC_withH	PVal_withH
PC(O-27:1)	PC[O]	#N/A	#N/A	0.308854	0.359093
PC(O-28:0)	PC[O]	0.052567	0.824578	0.168743	0.250066
PC(O-28:1)	PC[O]	-0.28091	0.549094	-0.01184	0.923405
PC(O-28:2)	PC[O]	#N/A	#N/A	-0.16539	0.777053
PC(O-29:0)	PC[O]	0.142145	0.308687	-0.16498	0.46503
PC(O-29:1)	PC[O]	0.115045	0.747135	-0.01525	0.917416
PC(O-30:0)	PC[O]	-0.11778	0.770712	-0.02378	0.807783
PC(O-30:1)	PC[O]	-0.10525	0.699014	0.169211	0.312842
PC(O-30:2)	PC[O]	#N/A	#N/A	0.051995	0.797051
PC(O-31:0)	PC[O]	0.126367	0.610773	-0.03229	0.850996
PC(O-31:1)	PC[O]	0.184759	0.483054	0.18558	0.325227
PC(O-31:2)	PC[O]	-0.11809	0.696846	-0.06186	0.815139
PC(O-31:3)	PC[O]	0.001159	0.995946	#N/A	#N/A
PC(O-32:0)	PC[O]	-0.16512	0.718165	-0.04865	0.697689
PC(O-32:1)	PC[O]	-0.06656	0.870513	0.202517	0.337586
PC(O-32:2)	PC[O]	#N/A	#N/A	0.204004	0.272032
PC(O-32:3)	PC[O]	0.050468	0.864589	0.162936	0.557302
PC(O-32:4)	PC[O]	0.015125	0.956209	#N/A	#N/A
PC(O-32:5)	PC[O]	0.23866	0.322232	#N/A	#N/A
PC(O-33:0)	PC[O]	0.091678	0.673161	-0.10501	0.705969
PC(O-33:1)	PC[O]	0.128861	0.328391	0.03198	0.792469
PC(O-33:2)	PC[O]	-0.00299	0.984561	0.067656	0.731781
PC(O-33:3)	PC[O]	#N/A	#N/A	-0.28774	0.466461
PC(O-34:0)	PC[O]	0.08752	0.730283	0.198864	0.294992
PC(O-34:1)	PC[O]	-0.03982	0.882117	-0.0324	0.785126
PC(O-34:2)	PC[O]	-0.15145	0.60451	0.269864	0.18089
PC(O-34:3)	PC[O]	#N/A	#N/A	0.236341	0.175
PC(O-34:4)	PC[O]	0.214057	0.428353	0.03902	0.746877
PC(O-34:5)	PC[O]	#N/A	#N/A	0.055866	0.82016
PC(O-34:6)	PC[O]	0.155547	0.668577	#N/A	#N/A
PC(O-35:0)	PC[O]	0.162368	0.393102	#N/A	#N/A
PC(O-35:1)	PC[O]	0.016273	0.951134	-0.38435	0.237085
PC(O-35:2)	PC[O]	-0.05162	0.768055	0.111337	0.456804
PC(O-35:3)	PC[O]	#N/A	#N/A	0.090234	0.787459
PC(O-35:4)	PC[O]	#N/A	#N/A	0.192824	0.25895
PC(O-35:5)	PC[O]	#N/A	#N/A	-0.0299	0.891801
PC(O-35:6)	PC[O]	#N/A	#N/A	0.060939	0.885431
PC(O-36:0)	PC[O]	-0.04461	0.866667	-0.20766	0.470873
PC(O-36:1)	PC[O]	-0.16612	0.76577	-0.05633	0.795541
PC(O-36:2)	PC[O]	-0.21897	0.61818	0.06015	0.759231
PC(O-36:3)	PC[O]	#N/A	#N/A	0.162006	0.466309
PC(O-36:4)	PC[O]	0.132777	0.616189	0.014563	0.942768
PC(O-36:5)	PC[O]	0.306988	0.205972	0.2244	0.234128
PC(O-36:6)	PC[O]	#N/A	#N/A	0.202208	0.422757
PC(O-36:7)	PC[O]	#N/A	#N/A	0.338037	0.416238
PC(O-37:0)	PC[O]	0.061213	0.732759	#N/A	#N/A
PC(O-37:1)	PC[O]	-0.3729	0.558528	#N/A	#N/A

Lipid	LipidClass	Log2FC_noH	PVal_noH	Log2FC_withH	PVal_withH
PC(O-37:2)	PC[O]	-0.53111	0.506759	#N/A	#N/A
PC(O-37:3)	PC[O]	-0.44537	0.509041	0.053226	0.87027
PC(O-37:4)	PC[O]	-0.04366	0.843903	0.039163	0.80673
PC(O-37:5)	PC[O]	0.538214	0.116714	0.115201	0.298667
PC(O-37:6)	PC[O]	#N/A	#N/A	0.138282	0.39227
PC(O-37:7)	PC[O]	#N/A	#N/A	-0.03379	0.888644
PC(O-38:0)	PC[O]	0.032536	0.873991	0.135666	0.355251
PC(O-38:1)	PC[O]	-0.3867	0.539643	0.112866	0.588426
PC(O-38:2)	PC[O]	-0.78562	0.456322	-0.26939	0.381475
PC(O-38:3)	PC[O]	-0.67476	0.476316	0.042628	0.849443
PC(O-38:4)	PC[O]	-0.13917	0.716978	0.074693	0.652948
PC(O-38:5)	PC[O]	0.097663	0.641583	-0.04235	0.644888
PC(O-38:6)	PC[O]	#N/A	#N/A	0.064431	0.624846
PC(O-38:7)	PC[O]	#N/A	#N/A	0.136311	0.408219
PC(O-38:8)	PC[O]	#N/A	#N/A	0.185648	0.460243
PC(O-39:0)	PC[O]	0.101752	0.479845	#N/A	#N/A
PC(O-39:1)	PC[O]	-0.07883	0.832394	-0.61327	0.609935
PC(O-39:2)	PC[O]	-0.29255	0.71243	#N/A	#N/A
PC(O-39:3)	PC[O]	-0.54922	0.472198	-0.98605	0.068086
PC(O-39:4)	PC[O]	-0.17231	0.694882	0.033205	0.866487
PC(O-39:5)	PC[O]	0.186919	0.335873	0.087543	0.673171
PC(O-39:6)	PC[O]	#N/A	#N/A	0.044643	0.780852
PC(O-39:7)	PC[O]	#N/A	#N/A	0.07121	0.600079
PC(O-39:8)	PC[O]	#N/A	#N/A	0.242799	0.281605
PC(O-39:9)	PC[O]	#N/A	#N/A	0.296341	0.300304
PC(O-40:0)	PC[O]	0.073178	0.777093	0.156897	0.678468
PC(O-40:1)	PC[O]	-0.08791	0.813197	0.163699	0.571245
PC(O-40:2)	PC[O]	-0.32178	0.575827	0.007266	0.975359
PC(O-40:3)	PC[O]	-0.48312	0.407458	0.010838	0.963809
PC(O-40:4)	PC[O]	#N/A	#N/A	0.032076	0.781047
PC(O-40:5)	PC[O]	0.073457	0.787578	0.067072	0.635214
PC(O-40:6)	PC[O]	0.042259	0.903128	0.026217	0.855884
PC(O-40:7)	PC[O]	0.106038	0.538651	-0.07476	0.515867
PC(O-40:8)	PC[O]	#N/A	#N/A	0.015487	0.929096
PC(O-40:9)	PC[O]	#N/A	#N/A	-0.00741	0.969249
PC(O-41:0)	PC[O]	0.18162	0.468307	#N/A	#N/A
PC(O-41:1)	PC[O]	0.031353	0.896093	-0.6197	0.444308
PC(O-41:2)	PC[O]	-0.17783	0.623525	-1.12922	0.147271
PC(O-41:3)	PC[O]	#N/A	#N/A	0.305034	0.234388
PC(O-41:4)	PC[O]	#N/A	#N/A	-0.0235	0.873562
PC(O-41:5)	PC[O]	#N/A	#N/A	-0.12669	0.489418
PC(O-41:6)	PC[O]	#N/A	#N/A	-0.08979	0.557714
PC(O-41:7)	PC[O]	0.148287	0.625398	-0.14484	0.622193
PC(O-41:8)	PC[O]	#N/A	#N/A	-0.5122	0.180872
PC(O-41:9)	PC[O]	#N/A	#N/A	0.51396	0.204305
PC(O-42:0)	PC[O]	0.226854	0.526953	#N/A	#N/A
PC(O-42:1)	PC[O]	0.036786	0.89049	-0.03849	0.891294

Lipid	LipidClass	Log2FC_noH	PVal_noH	Log2FC_withH	PVal_withH
PC(O-42:10)	PC[O]	#N/A	#N/A	0.026384	0.903189
PC(O-42:2)	PC[O]	-0.04899	0.898396	-0.20318	0.408625
PC(O-42:3)	PC[O]	#N/A	#N/A	0.14951	0.55227
PC(O-42:4)	PC[O]	#N/A	#N/A	0.004354	0.965814
PC(O-42:5)	PC[O]	#N/A	#N/A	-0.01492	0.894832
PC(O-42:6)	PC[O]	0.048517	0.821012	-0.06909	0.522626
PC(O-42:7)	PC[O]	#N/A	#N/A	-0.00087	0.994198
PC(O-42:8)	PC[O]	#N/A	#N/A	-0.03088	0.813514
PC(O-42:9)	PC[O]	#N/A	#N/A	0.202545	0.102421
PC(O-43:3)	PC[O]	#N/A	#N/A	0.052672	0.921905
PC(O-43:4)	PC[O]	#N/A	#N/A	0.10277	0.344012
PC(O-43:5)	PC[O]	#N/A	#N/A	0.082088	0.589414
PC(O-43:6)	PC[O]	#N/A	#N/A	-0.086	0.641915
PC(O-43:7)	PC[O]	#N/A	#N/A	-0.10037	0.795997
PC(O-44:10)	PC[O]	#N/A	#N/A	0.004441	0.98669
PC(O-44:2)	PC[O]	#N/A	#N/A	-0.25586	0.231946
PC(O-44:3)	PC[O]	#N/A	#N/A	0.32119	0.236343
PC(O-44:4)	PC[O]	#N/A	#N/A	0.088456	0.514639
PC(O-44:5)	PC[O]	#N/A	#N/A	0.034411	0.819115
PC(O-44:6)	PC[O]	0.090712	0.654861	-0.0167	0.90648
PC(O-44:7)	PC[O]	#N/A	#N/A	-0.00422	0.979045
PC(O-44:8)	PC[O]	#N/A	#N/A	-0.08113	0.694595
PC(O-44:9)	PC[O]	#N/A	#N/A	0.004941	0.984821
PC(O-45:4)	PC[O]	#N/A	#N/A	0.764856	0.134629
PC(O-45:5)	PC[O]	#N/A	#N/A	0.143902	0.457379
PC(O-45:6)	PC[O]	#N/A	#N/A	-0.04006	0.845968
PC(O-45:7)	PC[O]	#N/A	#N/A	0.184288	0.468301
PC(O-46:10)	PC[O]	#N/A	#N/A	-0.4962	0.175395
PC(O-46:2)	PC[O]	#N/A	#N/A	-0.17881	0.895678
PC(O-46:4)	PC[O]	#N/A	#N/A	0.071772	0.75963
PC(O-46:5)	PC[O]	#N/A	#N/A	0.161106	0.354222
PC(O-46:6)	PC[O]	#N/A	#N/A	0.027163	0.875207
PC(O-46:7)	PC[O]	#N/A	#N/A	-0.06445	0.764723
PC(O-46:8)	PC[O]	#N/A	#N/A	-0.10056	0.705433
PC(O-46:9)	PC[O]	#N/A	#N/A	-0.23176	0.39028
PC(O-48:5)	PC[O]	#N/A	#N/A	-0.73942	0.496666
PC(O-48:6)	PC[O]	#N/A	#N/A	0.436461	0.275451
PC(O-48:7)	PC[O]	#N/A	#N/A	-0.02015	0.956062
PC(O-48:8)	PC[O]	#N/A	#N/A	-0.19805	0.677433
PC(O-48:9)	PC[O]	#N/A	#N/A	-0.20695	0.739682
PE(28:1)	PE	0.039524	0.926386	-0.48224	0.124256
PE(29:0)	PE	0.021802	0.899701	-0.33424	0.05351
PE(29:1)	PE	0.347227	0.459038	#N/A	#N/A
PE(30:0)	PE	-0.15469	0.595164	-0.28919	0.072171
PE(30:1)	PE	0.041577	0.914149	-0.18668	0.365563
PE(30:2)	PE	0.220841	0.561163	-0.12831	0.583918
PE(30:3)	PE	#N/A	#N/A	-0.40796	0.334877

Lipid	LipidClass	Log2FC_noH	PVal_noH	Log2FC_withH	PVal_withH
PE(31:0)	PE	0.119415	0.49525	-0.16656	0.251223
PE(31:1)	PE	0.16167	0.432992	-0.0851	0.544865
PE(31:2)	PE	0.073642	0.792717	-0.08955	0.72553
PE(32:0)	PE	-0.17631	0.400482	-0.11835	0.489006
PE(32:1)	PE	-0.03434	0.900744	-0.08979	0.529181
PE(32:2)	PE	0.023678	0.949422	-0.09073	0.656566
PE(32:3)	PE	0.180162	0.429962	0.058581	0.796376
PE(32:4)	PE	0.513116	0.304027	0.136816	0.741152
PE(33:0)	PE	0.06695	0.663245	-0.11428	0.501317
PE(33:1)	PE	0.187856	0.096706	-0.03701	0.764721
PE(33:2)	PE	0.163647	0.445232	-0.1755	0.428111
PE(33:3)	PE	0.291131	0.191835	#N/A	#N/A
PE(33:4)	PE	#N/A	#N/A	-0.58063	0.204107
PE(34:0)	PE	-0.30762	0.340432	-0.08405	0.75464
PE(34:1)	PE	0.015818	0.913818	0.005335	0.965565
PE(34:2)	PE	0.036219	0.876229	-0.07787	0.621375
PE(34:3)	PE	0.050574	0.791196	-0.01655	0.920832
PE(34:4)	PE	0.004871	0.980871	-0.04657	0.691539
PE(34:5)	PE	0.371376	0.149585	-0.29447	0.29399
PE(35:0)	PE	-0.10504	0.643892	0.491714	0.342094
PE(35:1)	PE	0.200642	0.096668	0.052951	0.614405
PE(35:2)	PE	0.153197	0.26581	-0.01508	0.916826
PE(35:3)	PE	0.145378	0.332634	-0.0848	0.622841
PE(35:4)	PE	0.108796	0.534289	-0.11015	0.571833
PE(36:1)	PE	0.125859	0.371849	0.070389	0.530292
PE(36:2)	PE	0.007136	0.965124	-0.04231	0.730774
PE(36:3)	PE	0.083373	0.55244	-0.06484	0.680908
PE(36:4)	PE	0.029877	0.849862	-0.03866	0.829367
PE(36:5)	PE	0.151032	0.465176	-0.10505	0.60654
PE(36:6)	PE	0.26259	0.104507	-0.0381	0.871194
PE(37:1)	PE	0.021884	0.888099	-0.05448	0.63142
PE(37:2)	PE	-0.09779	0.626579	-0.09564	0.485063
PE(37:3)	PE	-0.00521	0.971172	0.054411	0.736707
PE(37:4)	PE	0.127778	0.292162	0.086273	0.576023
PE(37:5)	PE	0.192619	0.216972	-0.07278	0.703148
PE(37:6)	PE	0.237794	0.429548	-0.09176	0.670857
PE(38:1)	PE	-0.17985	0.355696	0.045438	0.815921
PE(38:2)	PE	-0.22644	0.429169	-0.10613	0.52773
PE(38:3)	PE	-0.02088	0.886221	0.113349	0.484409
PE(38:4)	PE	0.048619	0.713309	0.081857	0.567195
PE(38:5)	PE	-0.00995	0.951828	-0.01193	0.932061
PE(38:6)	PE	0.228653	0.143132	-0.09778	0.427224
PE(38:7)	PE	0.119311	0.606539	-0.05636	0.801556
PE(39:1)	PE	#N/A	#N/A	0.48462	0.130594
PE(39:2)	PE	-0.04089	0.905962	-0.23903	0.242437
PE(39:3)	PE	-0.25464	0.303225	-0.02919	0.840153
PE(39:4)	PE	-0.0783	0.686352	-0.03841	0.689856

Lipid	LipidClass	Log2FC_noH	PVal_noH	Log2FC_withH	PVal_withH
PE(39:5)	PE	0.100944	0.429102	-0.02368	0.842235
PE(39:6)	PE	0.274441	0.217504	-0.08641	0.456
PE(39:7)	PE	0.15588	0.464656	-0.21645	0.354189
PE(40:1)	PE	-0.07393	0.70354	0.114257	0.407628
PE(40:2)	PE	-0.27281	0.461927	-0.04921	0.775986
PE(40:3)	PE	-0.57302	0.128916	-0.1168	0.492706
PE(40:4)	PE	-0.13755	0.523009	-0.05714	0.720299
PE(40:5)	PE	-0.06959	0.7301	-0.11674	0.419938
PE(40:6)	PE	0.013426	0.936664	-0.17111	0.163095
PE(40:7)	PE	-0.05154	0.783686	-0.15345	0.255661
PE(40:8)	PE	0.111095	0.517221	0.004296	0.977874
PE(41:2)	PE	0.011899	0.984097	#N/A	#N/A
PE(41:3)	PE	-0.59997	0.121592	-0.02739	0.947966
PE(41:4)	PE	-0.35592	0.295703	-0.31802	0.070514
PE(41:5)	PE	-0.25129	0.376858	-0.32154	0.03518
PE(41:6)	PE	0.041557	0.853673	-0.31461	0.061696
PE(41:7)	PE	-0.0866	0.680467	-0.00484	0.978142
PE(42:1)	PE	0.111983	0.618274	0.125531	0.513295
PE(42:10)	PE	0.271075	0.257995	0.358329	0.093085
PE(42:11)	PE	#N/A	#N/A	0.241129	0.487476
PE(42:2)	PE	-0.07931	0.836888	-0.12407	0.478267
PE(42:3)	PE	-0.27517	0.419321	-0.15769	0.362852
PE(42:4)	PE	-0.19509	0.331462	-0.09501	0.553097
PE(42:5)	PE	-0.26424	0.252828	-0.1219	0.484844
PE(42:6)	PE	-0.11685	0.574375	-0.09566	0.473244
PE(42:7)	PE	-0.11627	0.621288	-0.02101	0.897484
PE(42:8)	PE	0.054915	0.806263	0.177941	0.368806
PE(42:9)	PE	0.140917	0.613783	0.333286	0.135696
PE(43:5)	PE	-0.19916	0.636902	-0.47572	0.326996
PE(43:6)	PE	-0.50393	0.354965	#N/A	#N/A
PE(43:7)	PE	-0.63371	0.134754	-0.08947	0.837735
PE(44:1)	PE	0.299644	0.436617	#N/A	#N/A
PE(44:10)	PE	0.384277	0.080507	0.24236	0.251482
PE(44:11)	PE	-0.01729	0.944946	0.138742	0.622947
PE(44:12)	PE	0.230104	0.451531	0.046399	0.825757
PE(44:2)	PE	-0.02076	0.965594	-0.29689	0.376707
PE(44:3)	PE	-0.1839	0.553772	0.047323	0.803342
PE(44:4)	PE	0.030699	0.881319	0.155626	0.433832
PE(44:5)	PE	-0.1518	0.579987	0.067788	0.752545
PE(44:6)	PE	-0.231	0.433269	-0.22875	0.199131
PE(44:7)	PE	-0.33322	0.36258	-0.09572	0.637573
PE(44:8)	PE	-0.12595	0.686721	-0.00171	0.994091
PE(44:9)	PE	0.168747	0.555877	0.151779	0.460367
PE(45:5)	PE	0.001195	0.998114	-0.85258	0.181173
PE(45:6)	PE	#N/A	#N/A	0.064698	0.935797
PE(46:10)	PE	0.381291	0.244118	-0.10027	0.666499
PE(46:11)	PE	#N/A	#N/A	-0.29257	0.497105

Lipid	LipidClass	Log2FC_noH	PVal_noH	Log2FC_withH	PVal_withH
PE(46:4)	PE	-0.24932	0.449594	0.145781	0.603352
PE(46:5)	PE	-0.21271	0.539247	-0.2652	0.29796
PE(46:6)	PE	-0.22255	0.465335	-0.33951	0.255132
PE(46:7)	PE	-0.49629	0.251068	-0.44122	0.090065
PE(46:8)	PE	-0.37386	0.420232	-0.25064	0.39079
PE(46:9)	PE	-0.07863	0.857066	#N/A	#N/A
PE(48:5)	PE	-0.45245	0.332033	-0.41387	0.520724
PE(48:6)	PE	-0.54937	0.206126	-0.56594	0.250366
PE(48:7)	PE	-0.50332	0.289658	#N/A	#N/A
PE(48:8)	PE	-0.71904	0.244218	#N/A	#N/A
PE(48:9)	PE	-0.5585	0.388581	#N/A	#N/A
PE(O-24:1)	PE[O]	#N/A	#N/A	-0.53954	0.204281
PE(O-25:0)	PE[O]	#N/A	#N/A	0.989787	0.02992
PE(O-25:1)	PE[O]	#N/A	#N/A	0.364613	0.114652
PE(O-26:0)	PE[O]	#N/A	#N/A	0.00256	0.994466
PE(O-26:1)	PE[O]	#N/A	#N/A	-0.00321	0.991539
PE(O-27:1)	PE[O]	#N/A	#N/A	0.075884	0.642523
PE(O-27:2)	PE[O]	#N/A	#N/A	0.100213	0.78093
PE(O-28:0)	PE[O]	#N/A	#N/A	0.115286	0.577095
PE(O-28:1)	PE[O]	#N/A	#N/A	-0.00962	0.959934
PE(O-28:2)	PE[O]	#N/A	#N/A	-0.08869	0.743325
PE(O-28:3)	PE[O]	#N/A	#N/A	-0.79781	0.124973
PE(O-29:1)	PE[O]	#N/A	#N/A	0.008495	0.970035
PE(O-29:2)	PE[O]	#N/A	#N/A	0.246434	0.090682
PE(O-30:0)	PE[O]	-0.26382	0.559932	-0.28944	0.310661
PE(O-30:1)	PE[O]	#N/A	#N/A	-0.16134	0.518154
PE(O-30:2)	PE[O]	#N/A	#N/A	0.115986	0.396164
PE(O-30:3)	PE[O]	#N/A	#N/A	-0.04846	0.833974
PE(O-30:4)	PE[O]	#N/A	#N/A	0.033233	0.937236
PE(O-30:5)	PE[O]	#N/A	#N/A	-0.00896	0.975505
PE(O-30:6)	PE[O]	#N/A	#N/A	0.15003	0.737184
PE(O-31:1)	PE[O]	#N/A	#N/A	-0.02206	0.928465
PE(O-31:2)	PE[O]	#N/A	#N/A	0.216933	0.257238
PE(O-31:3)	PE[O]	#N/A	#N/A	0.329687	0.330281
PE(O-31:5)	PE[O]	#N/A	#N/A	0.239262	0.314744
PE(O-32:0)	PE[O]	-0.08314	0.678717	-0.14526	0.539141
PE(O-32:1)	PE[O]	-0.004	0.986698	0.006734	0.980485
PE(O-32:2)	PE[O]	#N/A	#N/A	0.184359	0.228016
PE(O-32:3)	PE[O]	#N/A	#N/A	0.168073	0.224794
PE(O-32:4)	PE[O]	#N/A	#N/A	0.334858	0.156341
PE(O-32:5)	PE[O]	#N/A	#N/A	0.184122	0.149876
PE(O-32:6)	PE[O]	#N/A	#N/A	0.344391	0.372832
PE(O-32:7)	PE[O]	#N/A	#N/A	-0.60085	0.329273
PE(O-33:0)	PE[O]	0.145434	0.444898	0.442062	0.323552
PE(O-33:1)	PE[O]	0.482981	0.008009	0.096579	0.690338
PE(O-33:2)	PE[O]	#N/A	#N/A	0.268979	0.165277
PE(O-33:3)	PE[O]	#N/A	#N/A	0.11807	0.432176

Lipid	LipidClass	Log2FC_noH	PVal_noH	Log2FC_withH	PVal_withH
PE(O-33:4)	PE[O]	#N/A	#N/A	0.21904	0.455943
PE(O-33:5)	PE[O]	#N/A	#N/A	0.462042	0.121519
PE(O-34:0)	PE[O]	0.058648	0.770427	-0.14526	0.533463
PE(O-34:1)	PE[O]	-0.1465	0.44211	0.007232	0.977805
PE(O-34:2)	PE[O]	#N/A	#N/A	0.274901	0.144725
PE(O-34:3)	PE[O]	#N/A	#N/A	0.156932	0.296993
PE(O-34:4)	PE[O]	#N/A	#N/A	0.218976	0.248818
PE(O-34:5)	PE[O]	#N/A	#N/A	0.143447	0.520521
PE(O-34:6)	PE[O]	#N/A	#N/A	0.217927	0.444757
PE(O-34:7)	PE[O]	#N/A	#N/A	-0.11204	0.704358
PE(O-35:0)	PE[O]	-0.00307	0.987749	#N/A	#N/A
PE(O-35:1)	PE[O]	-0.07378	0.672985	0.180782	0.628372
PE(O-35:2)	PE[O]	#N/A	#N/A	0.345086	0.095021
PE(O-35:3)	PE[O]	#N/A	#N/A	0.224434	0.18206
PE(O-35:4)	PE[O]	#N/A	#N/A	0.312793	0.067128
PE(O-35:5)	PE[O]	#N/A	#N/A	0.137018	0.494362
PE(O-35:6)	PE[O]	#N/A	#N/A	0.186192	0.562325
PE(O-35:7)	PE[O]	#N/A	#N/A	0.435672	0.289843
PE(O-36:0)	PE[O]	-0.30057	0.223924	-0.04237	0.927606
PE(O-36:1)	PE[O]	-0.31556	0.354453	0.002631	0.993075
PE(O-36:2)	PE[O]	-0.36242	0.304914	0.344722	0.086518
PE(O-36:3)	PE[O]	#N/A	#N/A	0.262394	0.16025
PE(O-36:4)	PE[O]	#N/A	#N/A	0.211559	0.262458
PE(O-36:5)	PE[O]	#N/A	#N/A	0.227527	0.125019
PE(O-36:6)	PE[O]	#N/A	#N/A	0.165882	0.475995
PE(O-36:7)	PE[O]	#N/A	#N/A	0.034998	0.844278
PE(O-37:0)	PE[O]	-0.1836	0.561619	#N/A	#N/A
PE(O-37:1)	PE[O]	-0.38597	0.33778	#N/A	#N/A
PE(O-37:2)	PE[O]	#N/A	#N/A	0.173924	0.669089
PE(O-37:3)	PE[O]	#N/A	#N/A	0.281	0.207577
PE(O-37:4)	PE[O]	#N/A	#N/A	0.181263	0.324042
PE(O-37:5)	PE[O]	#N/A	#N/A	0.221673	0.056039
PE(O-37:6)	PE[O]	#N/A	#N/A	0.127572	0.405677
PE(O-37:7)	PE[O]	#N/A	#N/A	-0.01876	0.885645
PE(O-38:0)	PE[O]	-0.2943	0.308627	#N/A	#N/A
PE(O-38:1)	PE[O]	-0.59309	0.260676	0.111174	0.68465
PE(O-38:2)	PE[O]	-0.71996	0.257107	0.090298	0.664375
PE(O-38:3)	PE[O]	#N/A	#N/A	0.081734	0.713071
PE(O-38:4)	PE[O]	#N/A	#N/A	0.125505	0.433285
PE(O-38:5)	PE[O]	#N/A	#N/A	0.223467	0.085076
PE(O-38:6)	PE[O]	#N/A	#N/A	0.162077	0.172914
PE(O-38:7)	PE[O]	#N/A	#N/A	0.051281	0.678332
PE(O-38:8)	PE[O]	#N/A	#N/A	-0.02304	0.838
PE(O-39:0)	PE[O]	-0.10133	0.758147	#N/A	#N/A
PE(O-39:1)	PE[O]	-0.39089	0.326979	#N/A	#N/A
PE(O-39:2)	PE[O]	-0.56877	0.288717	-0.03874	0.911944
PE(O-39:3)	PE[O]	#N/A	#N/A	0.120349	0.819568

Lipid	LipidClass	Log2FC_noH	PVal_noH	Log2FC_withH	PVal_withH
PE(O-39:4)	PE[O]	#N/A	#N/A	-0.20374	0.364504
PE(O-39:5)	PE[O]	#N/A	#N/A	0.112136	0.338986
PE(O-39:6)	PE[O]	#N/A	#N/A	0.088809	0.459938
PE(O-39:7)	PE[O]	#N/A	#N/A	0.075602	0.510815
PE(O-39:8)	PE[O]	#N/A	#N/A	-0.05776	0.567486
PE(O-40:0)	PE[O]	-0.13234	0.7255	#N/A	#N/A
PE(O-40:1)	PE[O]	-0.39641	0.292611	#N/A	#N/A
PE(O-40:2)	PE[O]	-0.66426	0.216728	0.087844	0.723594
PE(O-40:3)	PE[O]	#N/A	#N/A	-0.0236	0.916217
PE(O-40:4)	PE[O]	#N/A	#N/A	-0.00265	0.986938
PE(O-40:5)	PE[O]	#N/A	#N/A	0.113538	0.395361
PE(O-40:6)	PE[O]	#N/A	#N/A	0.071965	0.577348
PE(O-40:7)	PE[O]	#N/A	#N/A	0.079519	0.547708
PE(O-40:8)	PE[O]	#N/A	#N/A	-0.00167	0.98767
PE(O-40:9)	PE[O]	#N/A	#N/A	-0.08181	0.5808
PE(O-41:1)	PE[O]	-0.00309	0.994305	#N/A	#N/A
PE(O-41:4)	PE[O]	#N/A	#N/A	0.966365	0.137737
PE(O-41:5)	PE[O]	#N/A	#N/A	0.108852	0.456571
PE(O-41:6)	PE[O]	#N/A	#N/A	0.132363	0.456486
PE(O-41:7)	PE[O]	#N/A	#N/A	0.042566	0.753146
PE(O-41:8)	PE[O]	#N/A	#N/A	0.034723	0.760017
PE(O-42:1)	PE[O]	-0.21171	0.498197	#N/A	#N/A
PE(O-42:10)	PE[O]	#N/A	#N/A	0.000876	0.995791
PE(O-42:11)	PE[O]	#N/A	#N/A	0.130487	0.486629
PE(O-42:2)_RT_6.824	PE[O]	#N/A	#N/A	0.884939	0.133808
PE(O-42:2)_RT_7.02	PE[O]	#N/A	#N/A	0.30095	0.450664
PE(O-42:3)	PE[O]	#N/A	#N/A	0.123081	0.704597
PE(O-42:4)	PE[O]	#N/A	#N/A	0.024773	0.913213
PE(O-42:5)	PE[O]	#N/A	#N/A	0.145337	0.299526
PE(O-42:6)	PE[O]	#N/A	#N/A	0.07463	0.594319
PE(O-42:7)	PE[O]	#N/A	#N/A	0.005632	0.966513
PE(O-42:8)	PE[O]	#N/A	#N/A	-0.00783	0.943216
PE(O-42:9)	PE[O]	#N/A	#N/A	-0.13255	0.371712
PE(O-43:5)	PE[O]	#N/A	#N/A	0.12318	0.485288
PE(O-43:6)	PE[O]	#N/A	#N/A	0.159163	0.461768
PE(O-43:7)	PE[O]	#N/A	#N/A	-0.00464	0.977908
PE(O-43:8)	PE[O]	#N/A	#N/A	0.013757	0.954176
PE(O-44:2)	PE[O]	#N/A	#N/A	0.09945	0.861285
PE(O-44:3)	PE[O]	#N/A	#N/A	0.105074	0.797246
PE(O-44:4)	PE[O]	#N/A	#N/A	-0.1431	0.685847
PE(O-44:5)	PE[O]	#N/A	#N/A	0.174719	0.43274
PE(O-44:6)	PE[O]	#N/A	#N/A	0.158753	0.453818
PE(O-44:7)	PE[O]	#N/A	#N/A	0.033795	0.813025
PE(O-44:8)	PE[O]	#N/A	#N/A	0.046653	0.790669
PE(O-44:9)	PE[O]	#N/A	#N/A	-0.0818	0.747089
PE(O-45:6)	PE[O]	#N/A	#N/A	0.003988	0.986283
PE(O-45:7)	PE[O]	#N/A	#N/A	-0.1362	0.498516

Lipid	LipidClass	Log2FC_noH	PVal_noH	Log2FC_withH	PVal_withH
PE(O-45:8)	PE[O]	#N/A	#N/A	0.072962	0.765672
PE(O-46:5)	PE[O]	#N/A	#N/A	-0.17499	0.661179
PE(O-46:6)	PE[O]	#N/A	#N/A	-0.09865	0.547005
PE(O-46:7)	PE[O]	#N/A	#N/A	-0.0694	0.663407
PE(O-46:8)	PE[O]	#N/A	#N/A	0.006715	0.975349
PE(O-46:9)	PE[O]	#N/A	#N/A	-0.1272	0.649362
PE(O-48:7)	PE[O]	#N/A	#N/A	-0.40024	0.222488
PE(O-48:8)	PE[O]	#N/A	#N/A	-0.38664	0.141677
PG(30:0)	PG	0.318574	0.385507	-0.03271	0.863383
PG(30:1)	PG	0.319239	0.61394	#N/A	#N/A
PG(32:0)	PG	0.326754	0.057026	-0.01232	0.933297
PG(32:1)	PG	0.173459	0.537142	-0.09706	0.563718
PG(32:2)	PG	0.054999	0.883553	-0.00245	0.983696
PG(33:0)	PG	#N/A	#N/A	-0.21977	0.582487
PG(33:1)	PG	0.313451	0.287399	0.011777	0.947751
PG(34:0)	PG	0.746916	0.231393	-0.04527	0.844741
PG(34:1)	PG	0.04564	0.840225	0.063304	0.776802
PG(34:2)	PG	0.051154	0.889061	-0.03818	0.806072
PG(34:3)	PG	0.152462	0.579838	-0.2371	0.129316
PG(35:1)	PG	0.361145	0.133704	0.116511	0.506695
PG(35:2)	PG	0.034587	0.871628	-0.02894	0.886855
PG(35:3)	PG	0.170579	0.457615	#N/A	#N/A
PG(36:1)	PG	0.335016	0.122318	-0.08075	0.660203
PG(36:2)	PG	-0.14685	0.574294	-0.06008	0.718625
PG(36:3)	PG	0.124069	0.548131	-0.13707	0.323291
PG(36:4)	PG	0.268571	0.292726	0.025207	0.881409
PG(36:5)	PG	0.254059	0.376611	-0.26825	0.231245
PG(37:1)	PG	0.374097	0.157393	-0.08135	0.750249
PG(37:2)	PG	#N/A	#N/A	-0.38014	0.288191
PG(37:3)	PG	0.135208	0.67402	0.174653	0.491514
PG(37:4)	PG	-0.26335	0.596321	0.034644	0.911874
PG(37:5)	PG	#N/A	#N/A	-0.24674	0.592077
PG(38:1)	PG	#N/A	#N/A	-0.22078	0.60365
PG(38:2)	PG	-0.04758	0.87494	-0.10079	0.671911
PG(38:3)	PG	0.085281	0.662412	0.118488	0.594162
PG(38:4)	PG	0.025383	0.919682	0.039632	0.82552
PG(38:5)	PG	0.040459	0.845291	-0.01457	0.943594
PG(38:6)	PG	0.36394	0.180493	-0.0715	0.690091
PG(38:7)	PG	#N/A	#N/A	-0.33696	0.419286
PG(40:1)	PG	#N/A	#N/A	0.032324	0.952141
PG(40:2)	PG	-0.26162	0.266013	-0.05525	0.779562
PG(40:3)	PG	-0.17323	0.481261	-0.22437	0.301033
PG(40:4)	PG	-0.1583	0.640138	-0.07273	0.717477
PG(40:5)	PG	-0.03052	0.925092	0.049195	0.758357
PG(40:6)	PG	0.195674	0.36994	-0.0324	0.906446
PG(40:7)	PG	0.299183	0.24525	0.053661	0.87926
PG(40:8)	PG	#N/A	#N/A	-0.15994	0.556054

Lipid	LipidClass	Log2FC_noH	PVal_noH	Log2FC_withH	PVal_withH
PG(40:9)	PG	#N/A	#N/A	0.07132	0.869298
PG(42:10)	PG	#N/A	#N/A	-0.02353	0.956782
PG(42:11)	PG	#N/A	#N/A	0.27846	0.568278
PG(42:8)	PG	#N/A	#N/A	-0.17964	0.494116
PG(44:10)	PG	#N/A	#N/A	-0.37445	0.578029
PG(44:11)	PG	#N/A	#N/A	0.204437	0.683806
PI(30:0)	PI	0.228489	0.570995	-0.06921	0.918094
PI(30:1)	PI	-0.15896	0.690438	-0.87396	0.182708
PI(31:0)	PI	0.090026	0.837803	0.039033	0.963392
PI(31:1)	PI	0.084501	0.870634	#N/A	#N/A
PI(32:0)	PI	0.064653	0.815172	#N/A	#N/A
PI(32:1)	PI	-0.0266	0.935331	-0.01307	0.977371
PI(32:2)	PI	-0.01676	0.96697	0.177666	0.675463
PI(33:1)	PI	0.113485	0.654386	-0.09456	0.787455
PI(33:2)	PI	0.132192	0.713066	-0.17071	0.572667
PI(34:1)	PI	-0.1226	0.675797	#N/A	#N/A
PI(34:2)	PI	-0.0852	0.855249	-0.10933	0.580269
PI(34:3)	PI	-0.05486	0.881743	0.016834	0.938339
PI(34:4)	PI	0.252808	0.435669	0.055717	0.837916
PI(35:1)	PI	0.076858	0.755463	-0.15373	0.340565
PI(35:2)	PI	0.112206	0.704551	-0.13856	0.556917
PI(35:3)	PI	-0.03156	0.93032	-0.09852	0.587326
PI(35:4)	PI	#N/A	#N/A	-0.00052	0.998674
PI(36:0)	PI	-0.47038	0.249433	#N/A	#N/A
PI(36:1)	PI	-0.10275	0.760242	-0.14556	0.517541
PI(36:2)	PI	-0.15137	0.711251	-0.09628	0.702936
PI(36:3)	PI	-0.1782	0.662154	-0.07199	0.767029
PI(36:4)	PI	0.105481	0.640353	0.091323	0.647092
PI(36:5)	PI	0.284155	0.145996	0.068847	0.722758
PI(37:1)	PI	-0.09069	0.857457	#N/A	#N/A
PI(37:2)	PI	-0.28612	0.586336	-0.26262	0.398555
PI(37:3)	PI	-0.14863	0.686893	0.034563	0.889071
PI(37:4)	PI	0.153502	0.419063	0.194103	0.319372
PI(37:5)	PI	0.400221	0.060944	0.167709	0.404075
PI(38:1)	PI	-0.05237	0.892459	#N/A	#N/A
PI(38:2)	PI	-0.39241	0.567344	-0.54619	0.317165
PI(38:3)	PI	-0.4307	0.362028	0.044831	0.842458
PI(38:4)	PI	-0.04138	0.832112	0.175151	0.407209
PI(38:5)	PI	0.105974	0.634915	0.106426	0.645506
PI(38:6)	PI	0.28182	0.156777	0.096532	0.665128
PI(39:2)	PI	-0.33163	0.581241	#N/A	#N/A
PI(39:3)	PI	-0.30799	0.491278	-0.17092	0.701879
PI(39:4)	PI	-0.13048	0.730051	0.031454	0.878316
PI(39:5)	PI	0.080721	0.720507	0.038888	0.878074
PI(39:6)	PI	0.245262	0.263284	-0.03196	0.897248
PI(40:2)	PI	-0.49108	0.53513	#N/A	#N/A
PI(40:3)	PI	-0.61597	0.30784	#N/A	#N/A

Lipid	LipidClass	Log2FC_noH	PVal_noH	Log2FC_withH	PVal_withH
PI(40:4)	PI	-0.28127	0.441243	0.016936	0.935576
PI(40:5)	PI	-0.01401	0.950553	0.069743	0.740445
PI(40:6)	PI	0.132315	0.546007	0.066514	0.782564
PI(40:7)	PI	0.209237	0.389729	0.029635	0.894978
PI(40:8)	PI	0.413413	0.079394	-0.03976	0.914928
PI(41:5)	PI	0.010625	0.983347	#N/A	#N/A
PI(41:6)	PI	0.125635	0.718703	-0.24471	0.573598
PI(42:4)	PI	-0.47642	0.398284	-0.19202	0.549237
PI(42:5)	PI	-0.33173	0.364287	-0.30498	0.353333
PI(42:6)	PI	-0.05786	0.868799	-0.16036	0.558297
PI(42:7)	PI	0.231178	0.467598	0.380166	0.181045
PS(30:0)	PS	-0.08294	0.823491	#N/A	#N/A
PS(30:1)	PS	0.102708	0.853177	#N/A	#N/A
PS(31:0)	PS	0.00583	0.985186	#N/A	#N/A
PS(31:1)	PS	0.242246	0.540529	#N/A	#N/A
PS(32:0)	PS	-0.27885	0.439696	-0.02311	0.958776
PS(32:1)	PS	-0.04849	0.903913	#N/A	#N/A
PS(32:2)	PS	0.328064	0.637728	#N/A	#N/A
PS(33:0)	PS	#N/A	#N/A	-0.04203	0.923616
PS(33:1)	PS	0.134951	0.313189	0.082099	0.730909
PS(33:2)	PS	0.246662	0.433691	#N/A	#N/A
PS(34:0)	PS	-0.35032	0.349457	-0.30153	0.244639
PS(34:1)	PS	0.080622	0.585502	0.126993	0.49118
PS(34:2)	PS	0.075425	0.746721	#N/A	#N/A
PS(34:3)	PS	0.053232	0.674134	#N/A	#N/A
PS(35:0)	PS	0.14088	0.499501	#N/A	#N/A
PS(35:1)	PS	0.234519	0.010445	0.106689	0.527157
PS(35:2)	PS	0.149318	0.377234	-0.10795	0.701027
PS(35:3)	PS	0.550634	0.07255	#N/A	#N/A
PS(36:0)	PS	-0.29433	0.235482	#N/A	#N/A
PS(36:1)	PS	0.165979	0.029529	0.025707	0.889941
PS(36:2)	PS	0.114033	0.475627	0.060793	0.686252
PS(36:3)	PS	0.178032	0.101694	0.162659	0.640297
PS(36:4)	PS	0.044772	0.700916	#N/A	#N/A
PS(36:5)	PS	0.157708	0.658113	#N/A	#N/A
PS(36:6)	PS	0.434092	0.189491	#N/A	#N/A
PS(37:0)	PS	0.10114	0.33666	#N/A	#N/A
PS(37:1)	PS	0.089958	0.381584	0.045475	0.856234
PS(37:2)	PS	0.011943	0.930812	-0.15091	0.479538
PS(37:3)	PS	0.105728	0.158117	-0.08344	0.787799
PS(37:4)	PS	-0.01316	0.931792	0.240928	0.51036
PS(37:5)	PS	0.068792	0.888056	#N/A	#N/A
PS(38:0)	PS	0.068485	0.544667	#N/A	#N/A
PS(38:1)	PS	-0.0204	0.886851	-0.05094	0.812254
PS(38:2)	PS	-0.16949	0.526976	-0.12468	0.533587
PS(38:3)	PS	0.182544	0.071948	0.137646	0.365995
PS(38:4)	PS	-0.05937	0.715102	0.264674	0.257279

Lipid	LipidClass	Log2FC_noH	PVal_noH	Log2FC_withH	PVal_withH
PS(38:5)	PS	0.079479	0.61262	0.24906	0.300385
PS(38:6)	PS	0.306904	0.244634	#N/A	#N/A
PS(38:7)	PS	0.135841	0.695625	#N/A	#N/A
PS(39:1)	PS	0.051499	0.661837	0.006081	0.978851
PS(39:2)	PS	0.493189	0.02317	0.00789	0.975406
PS(39:3)	PS	-0.15639	0.34192	-0.22488	0.274171
PS(39:4)	PS	-0.01041	0.927274	0.242815	0.32562
PS(39:5)	PS	-0.09432	0.757955	-0.11272	0.733284
PS(39:6)	PS	0.164234	0.655683	-0.10484	0.73799
PS(40:0)	PS	-0.39699	0.132584	#N/A	#N/A
PS(40:1)	PS	0.079306	0.524052	0.030977	0.863478
PS(40:2)	PS	-0.07174	0.809529	-0.09527	0.621449
PS(40:3)	PS	-0.37869	0.248066	-0.03456	0.875121
PS(40:4)	PS	-0.14234	0.493976	-0.01475	0.943597
PS(40:5)	PS	-0.19919	0.535668	-0.15721	0.591033
PS(40:6)	PS	-0.15956	0.577303	-0.26952	0.29724
PS(40:7)	PS	-0.20225	0.377184	-0.15531	0.624665
PS(40:8)	PS	-0.24566	0.507014	#N/A	#N/A
PS(41:0)	PS	0.177697	0.546815	#N/A	#N/A
PS(41:1)	PS	0.14586	0.476088	-0.15082	0.538451
PS(41:2)	PS	#N/A	#N/A	-0.20048	0.401696
PS(41:3)	PS	#N/A	#N/A	-0.08632	0.829083
PS(41:4)	PS	#N/A	#N/A	-0.11396	0.687643
PS(41:5)	PS	#N/A	#N/A	-0.27361	0.335268
PS(41:6)	PS	-0.59936	0.006599	-0.5244	0.083253
PS(41:7)	PS	-1.30536	0.037824	#N/A	#N/A
PS(42:0)	PS	-0.06963	0.884468	#N/A	#N/A
PS(42:1)	PS	0.166844	0.330957	0.020848	0.917133
PS(42:2)	PS	-0.05472	0.843139	-0.08141	0.677766
PS(42:3)	PS	-0.07279	0.765576	-0.11808	0.366931
PS(42:4)	PS	-0.17087	0.465612	-0.17763	0.243288
PS(42:5)	PS	-0.23529	0.277824	-0.13592	0.415792
PS(42:6)	PS	-0.52378	0.061671	-0.38692	0.085507
PS(42:7)	PS	-0.60493	0.118969	-0.50562	0.147998
PS(42:8)	PS	#N/A	#N/A	-0.26878	0.462999
PS(42:9)	PS	#N/A	#N/A	-0.17803	0.63059
PS(43:1)	PS	0.063243	0.772267	#N/A	#N/A
PS(43:2)	PS	-0.04769	0.853325	-0.48156	0.202447
PS(43:4)	PS	#N/A	#N/A	-0.13616	0.71756
PS(43:6)	PS	#N/A	#N/A	-0.74845	0.068328
PS(44:1)	PS	0.299874	0.167788	#N/A	#N/A
PS(44:2)	PS	0.073689	0.845364	-0.35374	0.24667
PS(44:3)	PS	-0.02623	0.930308	-0.27407	0.154111
PS(44:4)	PS	-0.13772	0.385256	-0.10373	0.592112
PS(44:5)	PS	0.339205	0.410301	-0.21984	0.293317
PS(44:6)	PS	-0.54602	0.097415	-0.44761	0.076606
PS(44:7)	PS	-0.84543	0.08903	-0.41977	0.126771

Lipid	LipidClass	Log2FC_noH	PVal_noH	Log2FC_withH	PVal_withH
PS(44:8)	PS	#N/A	#N/A	-0.08318	0.778717
PS(44:9)	PS	#N/A	#N/A	-0.24103	0.478347
PS(45:6)	PS	#N/A	#N/A	-1.24875	0.113659
PS(46:10)	PS	#N/A	#N/A	-0.32926	0.595236
PS(46:2)	PS	0.229158	0.643773	#N/A	#N/A
PS(46:3)	PS	-0.10907	0.807622	#N/A	#N/A
PS(46:4)	PS	-0.1897	0.486814	-0.28391	0.297184
PS(46:5)	PS	-0.50831	0.207314	-0.46333	0.201597
PS(46:6)	PS	-0.74128	0.106076	-0.30619	0.413004
PS(46:7)	PS	-0.75637	0.106271	-0.36536	0.262413
PS(46:8)	PS	-1.33914	0.038942	-0.70051	0.171396
PS(46:9)	PS	#N/A	#N/A	-0.57949	0.239977
PS(48:6)	PS	-1.05559	0.087744	#N/A	#N/A
SM(d26:1)	SM[d]	#N/A	#N/A	0.143104	0.68251
SM(d28:1)	SM[d]	#N/A	#N/A	0.341286	0.004544
SM(d29:1)	SM[d]	#N/A	#N/A	0.09347	0.353636
SM(d30:0)	SM[d]	#N/A	#N/A	0.249709	0.371266
SM(d31:0)	SM[d]	0.208744	0.35742	#N/A	#N/A
SM(d31:1)	SM[d]	0.040714	0.814871	0.114611	0.289225
SM(d32:0)	SM[d]	0.176823	0.35554	0.090121	0.623989
SM(d32:1)	SM[d]	0.030263	0.802545	0.134441	0.267436
SM(d32:2)	SM[d]	0.13106	0.396463	0.094969	0.501937
SM(d33:0)	SM[d]	0.303488	0.160993	0.062192	0.725405
SM(d33:1)	SM[d]	0.120117	0.237093	0.160946	0.199087
SM(d33:2)	SM[d]	0.185911	0.064249	0.10662	0.610112
SM(d34:0)	SM[d]	0.208082	0.313588	0.02312	0.891598
SM(d34:1)	SM[d]	0.068689	0.291717	0.100781	0.451657
SM(d34:2)	SM[d]	0.175207	0.156382	0.186756	0.249327
SM(d34:3)	SM[d]	0.218766	0.0986	0.270431	0.069296
SM(d35:0)	SM[d]	0.222532	0.404772	0.099373	0.59837
SM(d35:1)	SM[d]	0.021864	0.953848	0.113163	0.445938
SM(d35:2)	SM[d]	0.174349	0.561914	0.19731	0.121395
SM(d36:0)	SM[d]	0.085777	0.805247	0.153765	0.436875
SM(d36:1)	SM[d]	-0.24781	0.420423	0.056655	0.794825
SM(d36:2)	SM[d]	-0.15503	0.474819	0.010535	0.955644
SM(d36:3)	SM[d]	0.101693	0.512679	0.163338	0.515964
SM(d37:0)	SM[d]	0.027574	0.951808	0.057985	0.779816
SM(d37:1)	SM[d]	-0.26115	0.476799	-0.01331	0.959222
SM(d37:2)	SM[d]	-0.25842	0.514977	0.089474	0.637994
SM(d38:0)	SM[d]	0.156095	0.56868	0.240133	0.213942
SM(d38:1)	SM[d]	-0.18946	0.564378	0.190728	0.444008
SM(d38:2)	SM[d]	-0.22065	0.462549	0.089047	0.708991
SM(d38:3)	SM[d]	-0.15547	0.657979	-0.03295	0.88175
SM(d39:0)	SM[d]	0.356722	0.193954	0.281685	0.124405
SM(d39:1)	SM[d]	-0.01042	0.954658	0.182641	0.285375
SM(d39:2)	SM[d]	-0.13678	0.426398	0.086654	0.526159
SM(d39:3)	SM[d]	#N/A	#N/A	0.276001	0.120977

Lipid	LipidClass	Log2FC_noH	PVal_noH	Log2FC_withH	PVal_withH
SM(d40:0)	SM[d]	0.441382	0.10185	0.313578	0.065434
SM(d40:1)	SM[d]	0.10369	0.45841	0.16779	0.271492
SM(d40:2)	SM[d]	-0.13083	0.418427	0.085849	0.476335
SM(d40:3)	SM[d]	-0.13904	0.430061	-0.06785	0.597926
SM(d40:4)	SM[d]	-0.05524	0.829639	0.053714	0.723466
SM(d41:0)	SM[d]	0.447392	0.099718	0.242705	0.093474
SM(d41:1)	SM[d]	0.012306	0.932641	0.196385	0.190695
SM(d41:2)	SM[d]	-0.12196	0.403196	0.103713	0.37945
SM(d41:3)	SM[d]	-0.07086	0.517486	0.020023	0.850221
SM(d41:4)	SM[d]	#N/A	#N/A	-0.04144	0.791255
SM(d42:0)	SM[d]	0.245843	0.50579	0.352636	0.124181
SM(d42:1)	SM[d]	0.033361	0.776264	0.255835	0.134623
SM(d42:2)	SM[d]	-0.08044	0.483475	0.087463	0.429563
SM(d42:3)	SM[d]	-0.09693	0.429696	-0.02462	0.828969
SM(d42:4)	SM[d]	-0.03108	0.854437	-0.01946	0.894104
SM(d42:5)	SM[d]	0.159394	0.538582	0.085829	0.609801
SM(d42:6)	SM[d]	#N/A	#N/A	0.144967	0.519827
SM(d43:0)	SM[d]	0.633209	0.024909	0.224216	0.290279
SM(d43:1)	SM[d]	-0.05011	0.814924	0.145152	0.402694
SM(d43:2)	SM[d]	-0.1648	0.413764	0.010809	0.943034
SM(d43:3)	SM[d]	-0.04801	0.735456	-0.05592	0.698884
SM(d43:4)	SM[d]	-0.10354	0.715463	0.226986	0.22893
SM(d43:5)	SM[d]	0.338438	0.552257	#N/A	#N/A
SM(d44:0)	SM[d]	0.709873	0.072834	0.146804	0.571992
SM(d44:1)	SM[d]	0.051255	0.845587	0.150668	0.452133
SM(d44:2)	SM[d]	-0.14158	0.628475	0.047225	0.769635
SM(d44:3)	SM[d]	-0.06884	0.794001	-0.06011	0.683577
SM(d44:4)	SM[d]	-0.01963	0.94578	0.017808	0.924858
SM(d44:5)	SM[d]	0.035023	0.911022	-0.04583	0.845436
SM(d44:6)	SM[d]	0.382093	0.412072	0.362865	0.191066
SM(d44:7)	SM[d]	#N/A	#N/A	0.746008	0.026194
SM(d45:1)	SM[d]	-0.01814	0.955955	0.147986	0.640227
SM(d45:2)	SM[d]	-0.19192	0.552379	0.245641	0.302581
SM(d45:3)	SM[d]	-0.22501	0.501029	-0.165	0.424927
SM(d45:4)	SM[d]	-0.0164	0.958233	-0.10566	0.592608
SM(d45:5)	SM[d]	-0.22986	0.695122	#N/A	#N/A
SM(d46:1)	SM[d]	-0.00968	0.978145	#N/A	#N/A
SM(d46:2)	SM[d]	-0.1066	0.791274	-0.00408	0.98964
SM(d46:3)	SM[d]	-0.14257	0.71682	-0.03323	0.882883
SM(d46:4)	SM[d]	0.097535	0.793668	-0.11574	0.7389
SM(d46:5)	SM[d]	-0.06555	0.865861	#N/A	#N/A
SM(d46:6)	SM[d]	#N/A	#N/A	0.071271	0.820838
SM(t28:1)	SM[t]	#N/A	#N/A	-0.24693	0.376718
SM(t30:1)	SM[t]	-0.09807	0.767799	0.204078	0.239908
SM(t30:2)	SM[t]	#N/A	#N/A	-0.07189	0.829437
SM(t32:0)	SM[t]	0.108026	0.843913	0.251283	0.367225
SM(t32:1)	SM[t]	0.202334	0.359312	0.200366	0.279249

Lipid	LipidClass	Log2FC_noH	PVal_noH	Log2FC_withH	PVal_withH
SM(t32:2)	SM[t]	#N/A	#N/A	0.183044	0.361328
SM(t33:0)	SM[t]	#N/A	#N/A	0.21965	0.466161
SM(t33:1)	SM[t]	0.211603	0.400544	0.212792	0.19054
SM(t33:2)	SM[t]	0.291634	0.440436	#N/A	#N/A
SM(t34:0)	SM[t]	0.265693	0.556058	0.095825	0.609983
SM(t34:1)	SM[t]	0.305771	0.376537	0.583973	0.084833
SM(t34:2)	SM[t]	0.236235	0.082037	0.155278	0.26097
SM(t34:3)	SM[t]	#N/A	#N/A	-0.14964	0.743397
SM(t35:0)	SM[t]	#N/A	#N/A	-0.09608	0.757887
SM(t35:1)	SM[t]	#N/A	#N/A	0.080052	0.716817
SM(t35:2)	SM[t]	0.154441	0.465804	-0.06162	0.693586
SM(t36:0)	SM[t]	#N/A	#N/A	-0.29459	0.385729
SM(t36:1)	SM[t]	#N/A	#N/A	0.123361	0.54024
SM(t36:2)	SM[t]	#N/A	#N/A	0.146266	0.638804
SM(t36:3)	SM[t]	#N/A	#N/A	0.070596	0.851853
SM(t36:4)	SM[t]	#N/A	#N/A	0.229391	0.427647
SM(t36:5)	SM[t]	#N/A	#N/A	0.262329	0.188868
SM(t38:1)	SM[t]	#N/A	#N/A	0.453987	0.191987
SM(t39:1)	SM[t]	#N/A	#N/A	0.884796	0.045971
SM(t40:0)	SM[t]	#N/A	#N/A	0.027646	0.929482
SM(t40:1)	SM[t]	-0.01198	0.966954	0.078602	0.704924
SM(t40:2)	SM[t]	#N/A	#N/A	0.126525	0.532769
SM(t41:0)	SM[t]	#N/A	#N/A	0.823477	0.196975
SM(t41:1)	SM[t]	#N/A	#N/A	0.158424	0.417703
SM(t41:2)	SM[t]	#N/A	#N/A	0.158661	0.457908
SM(t42:0)	SM[t]	#N/A	#N/A	0.244276	0.437319
SM(t42:1)	SM[t]	-0.00489	0.97775	0.093357	0.6667
SM(t42:2)	SM[t]	-0.08046	0.651261	-0.02627	0.854809
SM(t42:3)	SM[t]	-0.11362	0.681348	-0.26258	0.329539
SM(t43:1)	SM[t]	#N/A	#N/A	-0.20033	0.629645
TG(30:0)	TG	#N/A	#N/A	0.256364	0.243871
TG(32:0)	TG	#N/A	#N/A	0.319043	0.08731
TG(33:0)	TG	#N/A	#N/A	0.755144	0.067931
TG(34:0)	TG	#N/A	#N/A	0.590882	0.044132
TG(34:1)	TG	#N/A	#N/A	0.588618	0.246245
TG(35:0)	TG	#N/A	#N/A	0.606967	0.0406
TG(35:1)	TG	#N/A	#N/A	0.845637	0.133472
TG(35:2)	TG	#N/A	#N/A	0.300472	0.415227
TG(36:0)	TG	#N/A	#N/A	0.400796	0.011252
TG(36:1)	TG	#N/A	#N/A	0.780021	0.15608
TG(36:2)	TG	#N/A	#N/A	0.679117	0.21728
TG(36:3)	TG	#N/A	#N/A	0.853777	0.18116
TG(37:0)	TG	#N/A	#N/A	0.257685	0.306866
TG(37:1)	TG	#N/A	#N/A	0.677036	0.150057
TG(37:2)	TG	#N/A	#N/A	0.881544	0.177695
TG(38:0)	TG	#N/A	#N/A	0.19956	0.101152
TG(38:1)	TG	#N/A	#N/A	0.714466	0.118169

Lipid	LipidClass	Log2FC_noH	PVal_noH	Log2FC_withH	PVal_withH
TG(38:2)	TG	#N/A	#N/A	0.742461	0.214287
TG(38:3)	TG	#N/A	#N/A	0.805923	0.195171
TG(38:4)	TG	#N/A	#N/A	0.763488	0.265084
TG(38:5)	TG	#N/A	#N/A	1.056567	0.200241
TG(39:0)	TG	#N/A	#N/A	0.123367	0.504551
TG(39:1)	TG	#N/A	#N/A	0.235384	0.30384
TG(39:2)	TG	#N/A	#N/A	0.386874	0.36762
TG(39:3)	TG	#N/A	#N/A	0.866055	0.168219
TG(40:0)	TG	0.038089	0.811521	0.259178	0.041176
TG(40:1)	TG	0.004082	0.971842	0.068438	0.735425
TG(40:2)	TG	-0.2235	0.331071	0.218525	0.52601
TG(40:3)	TG	-0.48997	0.295545	0.695109	0.159178
TG(40:4)	TG	#N/A	#N/A	0.9698	0.14817
TG(40:5)	TG	#N/A	#N/A	0.899402	0.206529
TG(40:6)	TG	#N/A	#N/A	0.750478	0.258475
TG(41:0)	TG	-0.02539	0.711799	0.169741	0.109396
TG(41:1)	TG	-0.00153	0.989861	-0.09564	0.565325
TG(41:2)	TG	-0.02842	0.887512	-0.02465	0.950412
TG(41:3)	TG	-0.05498	0.940342	0.446792	0.505044
TG(41:4)	TG	#N/A	#N/A	0.975801	0.180714
TG(41:5)	TG	#N/A	#N/A	1.318765	0.108256
TG(42:1)	TG	-0.04157	0.726291	0.111206	0.525401
TG(42:2)	TG	-0.09942	0.478288	-0.00721	0.971431
TG(42:3)	TG	-0.14642	0.710594	-0.12705	0.672362
TG(42:4)	TG	#N/A	#N/A	0.5315	0.323347
TG(42:5)	TG	#N/A	#N/A	0.629659	0.213787
TG(42:6)	TG	#N/A	#N/A	0.728482	0.193834
TG(42:7)	TG	#N/A	#N/A	0.376509	0.23707
TG(43:0)	TG	-0.07655	0.542997	0.147691	0.216861
TG(43:1)	TG	0.14926	0.44108	0.027644	0.89657
TG(43:2)	TG	-0.07259	0.660103	-0.06345	0.64905
TG(43:3)	TG	-0.01369	0.950514	-0.13733	0.501798
TG(43:4)	TG	#N/A	#N/A	-0.15938	0.768377
TG(44:0)	TG	-0.06508	0.740216	0.223224	0.465197
TG(44:1)	TG	-0.06636	0.698512	0.233943	0.447522
TG(44:2)	TG	-0.1196	0.481705	0.18829	0.489183
TG(44:3)	TG	-0.00171	0.991372	0.107439	0.736244
TG(44:4)	TG	#N/A	#N/A	0.194747	0.486988
TG(44:5)	TG	#N/A	#N/A	0.328218	0.365538
TG(44:6)	TG	#N/A	#N/A	0.517934	0.47276
TG(44:7)	TG	#N/A	#N/A	0.300153	0.050607
TG(45:0)	TG	-0.0405	0.814241	0.176286	0.484465
TG(45:1)	TG	0.00381	0.979889	0.156602	0.487395
TG(45:2)	TG	0.01146	0.954947	0.055026	0.766946
TG(45:3)	TG	0.048504	0.827716	-0.00242	0.990789
TG(45:4)	TG	#N/A	#N/A	-0.02016	0.948094
TG(46:0)	TG	0.079907	0.67499	0.114487	0.757644

Lipid	LipidClass	Log2FC_noH	PVal_noH	Log2FC_withH	PVal_withH
TG(46:1)	TG	-0.07243	0.784053	0.218759	0.575285
TG(46:2)	TG	-0.13177	0.582424	0.323193	0.419778
TG(46:3)	TG	-0.03248	0.873825	0.374585	0.478139
TG(46:4)	TG	#N/A	#N/A	0.324927	0.515665
TG(46:5)	TG	#N/A	#N/A	0.297486	0.47523
TG(46:6)	TG	#N/A	#N/A	0.373287	0.285892
TG(47:0)	TG	0.096023	0.536916	0.1433	0.595028
TG(47:1)	TG	0.052775	0.651979	0.182582	0.534712
TG(47:2)	TG	-0.031	0.845824	0.234371	0.415658
TG(47:3)	TG	0.010594	0.958082	0.246873	0.443314
TG(47:4)	TG	-0.0775	0.847456	0.148943	0.675101
TG(47:5)	TG	#N/A	#N/A	0.105057	0.770115
TG(47:6)	TG	#N/A	#N/A	0.12019	0.74823
TG(48:0)	TG	0.276515	0.20646	0.121956	0.666619
TG(48:1)	TG	0.023038	0.929694	0.145728	0.635909
TG(48:2)	TG	-0.11731	0.713317	0.265247	0.492091
TG(48:3)	TG	-0.13971	0.588845	0.416713	0.388326
TG(48:4)	TG	0.027013	0.933467	0.462169	0.34832
TG(48:5)	TG	#N/A	#N/A	0.547591	0.381136
TG(48:6)	TG	#N/A	#N/A	0.481324	0.306039
TG(48:7)	TG	#N/A	#N/A	0.113503	0.574153
TG(49:0)	TG	0.281252	0.302936	0.178015	0.459478
TG(49:1)	TG	0.178375	0.215528	0.128994	0.579445
TG(49:2)	TG	0.032525	0.816422	0.193457	0.475709
TG(49:3)	TG	-0.03188	0.837623	0.318485	0.372508
TG(49:4)	TG	-0.07825	0.634139	0.40785	0.323127
TG(49:5)	TG	#N/A	#N/A	0.401666	0.387809
TG(49:6)	TG	#N/A	#N/A	0.322068	0.320811
TG(49:7)	TG	#N/A	#N/A	0.064389	0.894932
TG(50:0)	TG	0.326949	0.422354	0.20079	0.261638
TG(50:1)	TG	0.118657	0.5287	0.15123	0.423484
TG(50:2)	TG	-0.0817	0.785664	0.183774	0.437999
TG(50:3)	TG	-0.13382	0.68653	0.312302	0.357969
TG(50:4)	TG	-0.09927	0.711546	0.380461	0.32608
TG(50:5)	TG	0.009414	0.970146	0.483019	0.260017
TG(50:6)	TG	0.048541	0.921158	0.465249	0.316341
TG(50:7)	TG	#N/A	#N/A	0.590571	0.316848
TG(51:0)	TG	0.161414	0.720934	0.234787	0.17189
TG(51:1)	TG	0.229361	0.337143	0.208685	0.251665
TG(51:2)	TG	0.11561	0.417011	0.156241	0.414726
TG(51:3)	TG	0.009716	0.954149	0.253305	0.286553
TG(51:4)	TG	0.026678	0.882821	0.327806	0.234575
TG(51:5)	TG	0.128399	0.521817	0.411958	0.193359
TG(51:6)	TG	0.315464	0.402238	0.401798	0.264216
TG(51:7)	TG	#N/A	#N/A	0.21634	0.555832
TG(51:8)	TG	#N/A	#N/A	0.270694	0.577086
TG(52:0)	TG	0.165238	0.774446	0.310524	0.150289

Lipid	LipidClass	Log2FC_noH	PVal_noH	Log2FC_withH	PVal_withH
TG(52:1)	TG	0.173972	0.512215	0.24781	0.185243
TG(52:10)	TG	#N/A	#N/A	1.104643	0.247548
TG(52:2)	TG	-0.02909	0.882461	0.182717	0.296348
TG(52:3)	TG	-0.09981	0.736444	0.228568	0.293556
TG(52:4)	TG	-0.0458	0.863114	0.262771	0.265778
TG(52:5)	TG	0.033553	0.899995	0.333255	0.218774
TG(52:6)	TG	0.104847	0.714057	0.412844	0.217075
TG(52:7)	TG	0.089449	0.718745	0.501096	0.250001
TG(52:8)	TG	#N/A	#N/A	0.624256	0.277047
TG(52:9)	TG	#N/A	#N/A	0.763245	0.269742
TG(53:0)	TG	0.066064	0.783868	0.248359	0.168348
TG(53:1)	TG	0.205968	0.514075	0.340495	0.114433
TG(53:2)	TG	0.101252	0.516045	0.248224	0.183756
TG(53:3)	TG	0.01944	0.912011	0.246446	0.195353
TG(53:4)	TG	0.013807	0.952025	0.285159	0.187928
TG(53:5)	TG	0.150226	0.532567	0.286623	0.127844
TG(53:6)	TG	0.268728	0.522971	0.389148	0.127358
TG(53:7)	TG	0.22963	0.404139	0.419448	0.209241
TG(53:8)	TG	#N/A	#N/A	0.472362	0.241376
TG(53:9)	TG	#N/A	#N/A	0.405711	0.550226
TG(54:0)	TG	0.087604	0.72166	0.390447	0.099842
TG(54:1)	TG	0.240929	0.397203	0.413864	0.143895
TG(54:10)	TG	#N/A	#N/A	0.80839	0.179739
TG(54:11)	TG	#N/A	#N/A	0.743255	0.320727
TG(54:2)	TG	0.04093	0.749513	0.290015	0.153044
TG(54:3)	TG	-0.04098	0.556347	0.254972	0.172353
TG(54:4)	TG	-0.01084	0.951908	0.288765	0.170036
TG(54:5)	TG	0.043515	0.837217	0.264602	0.146805
TG(54:6)	TG	0.069981	0.767662	0.310858	0.122797
TG(54:7)	TG	0.045014	0.864575	0.381761	0.166794
TG(54:8)	TG	0.002867	0.991493	0.496124	0.171543
TG(54:9)	TG	#N/A	#N/A	0.59064	0.183578
TG(55:0)	TG	0.001038	0.994741	0.227587	0.248758
TG(55:1)	TG	0.125737	0.437198	0.336003	0.153298
TG(55:10)	TG	#N/A	#N/A	0.618685	0.168195
TG(55:11)	TG	#N/A	#N/A	0.32342	0.335392
TG(55:2)	TG	0.109582	0.422139	0.331273	0.145138
TG(55:3)	TG	-0.01784	0.93055	0.284632	0.185982
TG(55:4)	TG	0.01326	0.965787	0.335628	0.112607
TG(55:5)	TG	0.108309	0.656187	0.284355	0.111533
TG(55:6)	TG	0.183602	0.592124	0.319888	0.079803
TG(55:7)	TG	0.198512	0.552701	0.333206	0.11564
TG(55:8)	TG	0.136245	0.642438	0.417364	0.11358
TG(55:9)	TG	#N/A	#N/A	0.584419	0.093037
TG(56:0)	TG	0.122669	0.447068	0.441901	0.219023
TG(56:1)	TG	0.098947	0.519683	0.428085	0.188008
TG(56:10)	TG	0.288907	0.550684	0.57545	0.083151

Lipid	LipidClass	Log2FC_noH	PVal_noH	Log2FC_withH	PVal_withH
TG(56:11)	TG	#N/A	#N/A	0.598358	0.100051
TG(56:12)	TG	#N/A	#N/A	0.280014	0.387094
TG(56:2)	TG	-0.00473	0.974958	0.349787	0.2046
TG(56:3)	TG	-0.09882	0.677183	0.387222	0.113555
TG(56:4)	TG	-0.10489	0.711585	0.389992	0.083525
TG(56:5)	TG	-0.00711	0.978991	0.31713	0.086838
TG(56:6)	TG	0.081099	0.792092	0.308442	0.083579
TG(56:7)	TG	0.066723	0.834198	0.323684	0.101249
TG(56:8)	TG	0.035764	0.903518	0.355805	0.102668
TG(56:9)	TG	0.075272	0.824424	0.471642	0.090067
TG(57:0)	TG	0.029669	0.836138	0.285018	0.232861
TG(57:1)	TG	0.079998	0.549715	0.355595	0.21931
TG(57:10)	TG	#N/A	#N/A	0.50279	0.073163
TG(57:11)	TG	#N/A	#N/A	0.569815	0.113872
TG(57:2)	TG	0.040905	0.73126	0.359043	0.199581
TG(57:3)	TG	-0.08673	0.620019	0.312468	0.244434
TG(57:4)	TG	-0.12956	0.665225	0.365837	0.119525
TG(57:5)	TG	-0.07007	0.815247	0.350281	0.075973
TG(57:6)	TG	0.163168	0.58831	0.332296	0.088536
TG(57:7)	TG	0.044606	0.891089	0.308708	0.104927
TG(57:8)	TG	0.141008	0.654401	0.32774	0.118464
TG(57:9)	TG	0.149243	0.730562	0.435218	0.075106
TG(58:0)	TG	0.178062	0.450145	0.471145	0.228757
TG(58:1)	TG	0.145299	0.475827	0.479309	0.193649
TG(58:10)	TG	0.138006	0.729693	0.425932	0.094248
TG(58:11)	TG	0.061105	0.875586	0.537468	0.094177
TG(58:12)	TG	#N/A	#N/A	0.543451	0.147566
TG(58:13)	TG	#N/A	#N/A	0.834405	0.157389
TG(58:2)	TG	-0.05612	0.630019	0.407896	0.21606
TG(58:3)	TG	-0.2067	0.446105	0.386324	0.22782
TG(58:4)	TG	-0.23406	0.491306	0.416875	0.175839
TG(58:5)	TG	-0.1885	0.585502	0.349471	0.133796
TG(58:6)	TG	-0.02064	0.949317	0.374311	0.095902
TG(58:7)	TG	0.098554	0.757752	0.344317	0.103029
TG(58:8)	TG	0.055938	0.860389	0.293783	0.148642
TG(58:9)	TG	0.166183	0.669527	0.37981	0.092119
TG(59:0)	TG	0.058521	0.576324	0.313911	0.197381
TG(59:1)	TG	0.121739	0.408848	0.428043	0.204112
TG(59:10)	TG	0.114443	0.839725	0.386766	0.092889
TG(59:11)	TG	#N/A	#N/A	0.495237	0.08361
TG(59:12)	TG	#N/A	#N/A	0.468991	0.15493
TG(59:13)	TG	#N/A	#N/A	0.492407	0.353379
TG(59:2)	TG	0.018683	0.87613	0.384588	0.231318
TG(59:3)	TG	-0.09588	0.522419	0.380046	0.239967
TG(59:4)	TG	-0.29233	0.164078	0.384217	0.21176
TG(59:5)	TG	-0.20556	0.577392	0.35634	0.17711
TG(59:6)	TG	-0.14241	0.677839	0.327189	0.155139

Lipid	LipidClass	Log2FC_noH	PVal_noH	Log2FC_withH	PVal_withH
TG(59:7)	TG	-0.02956	0.922438	0.316504	0.160321
TG(59:8)	TG	-0.02248	0.948551	0.338133	0.139512
TG(59:9)	TG	-0.03831	0.936291	0.374659	0.089325
TG(60:0)	TG	0.128001	0.670905	0.450595	0.177461
TG(60:1)	TG	0.145738	0.611325	0.57921	0.169471
TG(60:10)	TG	0.177951	0.680021	0.381388	0.118379
TG(60:11)	TG	0.154639	0.731818	0.42716	0.123237
TG(60:12)	TG	-0.04464	0.909633	0.427889	0.175613
TG(60:13)	TG	0.105815	0.846002	0.480754	0.191955
TG(60:2)	TG	-0.02793	0.851244	0.469091	0.17673
TG(60:3)	TG	-0.20942	0.381805	0.426392	0.213914
TG(60:4)	TG	-0.26314	0.34152	0.455026	0.205312
TG(60:5)	TG	-0.23607	0.486485	0.40215	0.233031
TG(60:6)	TG	-0.16336	0.662344	0.364561	0.216653
TG(60:7)	TG	-0.04404	0.908566	0.363718	0.186942
TG(60:8)	TG	0.053391	0.889647	0.339003	0.176302
TG(60:9)	TG	0.160102	0.701589	0.364601	0.111212
TG(61:0)	TG	0.054217	0.586723	0.155877	0.344712
TG(61:1)	TG	0.077938	0.60005	0.484278	0.188255
TG(61:10)	TG	#N/A	#N/A	0.386572	0.136404
TG(61:11)	TG	#N/A	#N/A	0.414528	0.135811
TG(61:12)	TG	#N/A	#N/A	0.374651	0.230803
TG(61:13)	TG	#N/A	#N/A	0.428678	0.270622
TG(61:2)	TG	0.062846	0.605336	0.439697	0.210572
TG(61:3)	TG	-0.06532	0.642985	0.390944	0.256589
TG(61:4)	TG	-0.03472	0.879073	0.435861	0.23519
TG(61:5)	TG	-0.16877	0.656863	0.449445	0.170387
TG(61:6)	TG	-0.20742	0.740353	0.367517	0.261667
TG(61:7)	TG	-0.12957	0.812234	0.330146	0.247394
TG(61:8)	TG	-0.23499	0.57611	0.415832	0.101158
TG(61:9)	TG	#N/A	#N/A	0.389223	0.131066
TG(62:0)	TG	0.017547	0.878415	0.174169	0.30463
TG(62:1)	TG	0.103103	0.662196	0.675509	0.219121
TG(62:10)	TG	0.226345	0.61445	0.325879	0.226579
TG(62:11)	TG	0.224663	0.625518	0.338459	0.221059
TG(62:12)	TG	-0.09561	0.812682	0.421826	0.193903
TG(62:13)	TG	-0.16576	0.714852	0.46533	0.23373
TG(62:14)	TG	#N/A	#N/A	0.514435	0.226928
TG(62:15)	TG	#N/A	#N/A	0.515182	0.23635
TG(62:2)	TG	0.023757	0.88728	0.508931	0.219359
TG(62:3)	TG	-0.17112	0.453139	0.462358	0.240154
TG(62:4)	TG	-0.2371	0.330663	0.500388	0.221654
TG(62:5)	TG	-0.29503	0.357328	0.495171	0.193529
TG(62:6)	TG	-0.28877	0.438437	0.450338	0.215711
TG(62:7)	TG	-0.2124	0.582614	0.406009	0.255171
TG(62:8)	TG	0.024473	0.957664	0.337358	0.268013
TG(62:9)	TG	0.082251	0.857775	0.335879	0.239018

Lipid	LipidClass	Log2FC_noH	PVal_noH	Log2FC_withH	PVal_withH
TG(63:0)	TG	0.042117	0.731194	-0.05638	0.802854
TG(63:1)	TG	0.069643	0.768497	0.314553	0.200845
TG(63:10)	TG	#N/A	#N/A	0.346376	0.250235
TG(63:11)	TG	#N/A	#N/A	0.239519	0.426775
TG(63:12)	TG	#N/A	#N/A	0.473348	0.243629
TG(63:13)	TG	#N/A	#N/A	0.627837	0.233206
TG(63:2)	TG	0.014399	0.886142	0.434547	0.175757
TG(63:3)	TG	-0.08349	0.518193	0.397229	0.269573
TG(63:4)	TG	-0.09067	0.612826	0.474379	0.227732
TG(63:5)	TG	-0.19479	0.550709	0.472236	0.210129
TG(63:6)	TG	-0.20986	0.645187	0.435309	0.225626
TG(63:7)	TG	#N/A	#N/A	0.40479	0.208237
TG(63:8)	TG	0.027623	0.839736	0.367302	0.203507
TG(63:9)	TG	#N/A	#N/A	0.331246	0.289059
TG(64:0)	TG	0.028155	0.820314	0.118328	0.384053
TG(64:1)	TG	0.065021	0.665757	0.471352	0.168665
TG(64:10)	TG	#N/A	#N/A	0.311473	0.361488
TG(64:11)	TG	#N/A	#N/A	0.29689	0.37996
TG(64:12)	TG	#N/A	#N/A	0.350642	0.300235
TG(64:13)	TG	#N/A	#N/A	0.484483	0.192842
TG(64:14)	TG	#N/A	#N/A	0.540356	0.18268
TG(64:15)	TG	#N/A	#N/A	0.496601	0.276165
TG(64:16)	TG	#N/A	#N/A	0.513496	0.287527
TG(64:17)	TG	#N/A	#N/A	0.415911	0.435223
TG(64:2)	TG	0.018948	0.847413	0.494915	0.163954
TG(64:3)	TG	-0.11872	0.468564	0.465295	0.270261
TG(64:4)	TG	-0.11416	0.628402	0.508359	0.278503
TG(64:5)	TG	-0.19722	0.502766	0.485068	0.243414
TG(64:6)	TG	-0.17735	0.579505	0.44836	0.255671
TG(64:7)	TG	-0.20329	0.591149	0.421765	0.256474
TG(64:8)	TG	-0.29716	0.55107	0.351622	0.361503
TG(64:9)	TG	0.177331	0.778074	0.357097	0.3374
TG(65:0)	TG	-0.13586	0.284813	-0.17518	0.375905
TG(65:1)	TG	-0.03872	0.83987	0.33486	0.127597
TG(65:10)	TG	#N/A	#N/A	0.37414	0.287154
TG(65:11)	TG	#N/A	#N/A	0.546433	0.134574
TG(65:12)	TG	#N/A	#N/A	0.514515	0.316953
TG(65:2)	TG	-0.0505	0.674971	0.345156	0.189638
TG(65:3)	TG	0.134364	0.361447	0.35684	0.233008
TG(65:4)	TG	0.126155	0.336056	0.401294	0.294999
TG(65:5)	TG	-0.32702	0.366702	0.408586	0.325792
TG(65:6)	TG	#N/A	#N/A	0.37442	0.337043
TG(65:7)	TG	#N/A	#N/A	0.399737	0.284368
TG(65:8)	TG	#N/A	#N/A	0.408119	0.276569
TG(65:9)	TG	#N/A	#N/A	0.276509	0.479348
TG(66:0)	TG	0.081769	0.65564	0.103516	0.407093
TG(66:1)	TG	0.117561	0.400636	0.511065	0.208125

Lipid	LipidClass	Log2FC_noH	PVal_noH	Log2FC_withH	PVal_withH
TG(66:10)	TG	#N/A	#N/A	0.329363	0.442383
TG(66:11)	TG	#N/A	#N/A	0.316344	0.39004
TG(66:12)	TG	#N/A	#N/A	0.33009	0.399787
TG(66:13)	TG	#N/A	#N/A	0.421599	0.251703
TG(66:14)	TG	#N/A	#N/A	0.502749	0.231136
TG(66:15)	TG	#N/A	#N/A	0.523299	0.253659
TG(66:16)	TG	#N/A	#N/A	0.634491	0.200314
TG(66:17)	TG	#N/A	#N/A	0.65265	0.243071
TG(66:18)	TG	#N/A	#N/A	0.546616	0.375722
TG(66:2)	TG	0.03624	0.787463	0.506523	0.177936
TG(66:3)	TG	-0.15781	0.229918	0.445766	0.200297
TG(66:4)	TG	-0.12185	0.36103	0.468079	0.314363
TG(66:5)	TG	-0.19017	0.380174	0.444785	0.339224
TG(66:6)	TG	-0.17706	0.588466	0.406537	0.360311
TG(66:7)	TG	-0.11716	0.723199	0.374078	0.390374
TG(66:8)	TG	-0.11813	0.759807	0.388023	0.368402
TG(66:9)	TG	0.169324	0.686742	0.370412	0.382687
TG(67:0)	TG	-0.07393	0.536181	-0.10272	0.657585
TG(67:1)	TG	0.20166	0.570144	0.07777	0.776726
TG(67:10)	TG	#N/A	#N/A	0.444364	0.327072
TG(67:11)	TG	#N/A	#N/A	0.248955	0.613325
TG(67:2)	TG	-0.20493	0.405288	0.359251	0.266445
TG(67:3)	TG	-0.01801	0.946449	0.298249	0.381226
TG(67:4)	TG	-0.16543	0.481513	0.298164	0.420398
TG(67:5)	TG	#N/A	#N/A	0.374373	0.436632
TG(67:6)	TG	#N/A	#N/A	0.569195	0.131371
TG(67:7)	TG	#N/A	#N/A	0.309713	0.471249
TG(67:8)	TG	#N/A	#N/A	0.408425	0.327152
TG(67:9)	TG	#N/A	#N/A	0.322222	0.458353
TG(68:0)	TG	-0.33427	0.295998	0.067735	0.782968
TG(68:1)	TG	0.3927	0.114705	0.611713	0.200993
TG(68:10)	TG	#N/A	#N/A	0.335959	0.462977
TG(68:11)	TG	#N/A	#N/A	0.257262	0.569043
TG(68:12)	TG	#N/A	#N/A	0.214639	0.620192
TG(68:13)	TG	#N/A	#N/A	0.41012	0.353499
TG(68:15)	TG	#N/A	#N/A	0.396482	0.448107
TG(68:17)	TG	#N/A	#N/A	0.681356	0.264652
TG(68:2)	TG	-0.01449	0.942937	0.536924	0.227577
TG(68:3)	TG	-0.23361	0.296819	0.401227	0.322584
TG(68:4)	TG	0.098345	0.579699	0.460814	0.285437
TG(68:5)	TG	-0.21581	0.380845	0.373998	0.398986
TG(68:6)	TG	-0.44595	0.213561	0.314898	0.505506
TG(68:7)	TG	-0.17242	0.651325	0.334902	0.481822
TG(68:8)	TG	#N/A	#N/A	0.35123	0.464161
TG(68:9)	TG	#N/A	#N/A	0.366751	0.457516
TG(69:0)	TG	#N/A	#N/A	-0.45519	0.42638
TG(69:10)	TG	#N/A	#N/A	0.176911	0.769519

Lipid	LipidClass	Log2FC_noH	PVal_noH	Log2FC_withH	PVal_withH
TG(69:11)	TG	#N/A	#N/A	0.077992	0.889725
TG(69:2)	TG	#N/A	#N/A	0.49397	0.233144
TG(69:3)	TG	#N/A	#N/A	0.546447	0.193814
TG(69:4)	TG	#N/A	#N/A	0.252875	0.416367
TG(69:5)	TG	#N/A	#N/A	0.402294	0.364013
TG(69:6)	TG	#N/A	#N/A	0.154526	0.737623
TG(69:7)	TG	#N/A	#N/A	0.387237	0.429825
TG(69:8)	TG	#N/A	#N/A	0.814518	0.09133
TG(69:9)	TG	#N/A	#N/A	0.568993	0.242306
TG(70:0)	TG	#N/A	#N/A	-0.69447	0.339589
TG(70:10)	TG	#N/A	#N/A	0.347538	0.495614
TG(70:11)	TG	#N/A	#N/A	0.254094	0.610375
TG(70:12)	TG	#N/A	#N/A	0.118955	0.827148
TG(70:13)	TG	#N/A	#N/A	0.089431	0.868932
TG(70:2)	TG	#N/A	#N/A	0.467416	0.306105
TG(70:3)	TG	#N/A	#N/A	0.313215	0.484931
TG(70:4)	TG	#N/A	#N/A	0.190225	0.682706
TG(70:5)	TG	#N/A	#N/A	0.316453	0.516375
TG(70:6)	TG	#N/A	#N/A	0.251776	0.630102
TG(70:7)	TG	#N/A	#N/A	0.257569	0.631421
TG(70:8)	TG	#N/A	#N/A	0.347508	0.50541
TG(70:9)	TG	#N/A	#N/A	0.31628	0.570258
TG(71:5)	TG	#N/A	#N/A	0.831074	0.235304
TG(71:7)	TG	#N/A	#N/A	0.510647	0.376694
TG(71:8)	TG	#N/A	#N/A	0.468563	0.462856
TG(71:9)	TG	#N/A	#N/A	0.397265	0.579113
TG(72:10)	TG	#N/A	#N/A	0.100077	0.864338
TG(72:11)	TG	#N/A	#N/A	0.161631	0.799136
TG(72:12)	TG	#N/A	#N/A	0.235891	0.731736
TG(72:4)	TG	#N/A	#N/A	0.744953	0.470247
TG(72:5)	TG	#N/A	#N/A	0.21741	0.723523
TG(72:6)	TG	#N/A	#N/A	0.273151	0.635947
TG(72:7)	TG	#N/A	#N/A	0.100567	0.866483
TG(72:8)	TG	#N/A	#N/A	0.172357	0.782798
TG(72:9)	TG	#N/A	#N/A	0.201007	0.76281
TG(74:6)	TG	#N/A	#N/A	-0.37394	0.800046
TG(O-42:0)	TG[O]	#N/A	#N/A	0.351794	0.486985
TG(O-42:1)	TG[O]	0.08829	0.789476	#N/A	#N/A
TG(O-43:0)	TG[O]	-0.18528	0.528134	0.440233	0.42493
TG(O-43:1)	TG[O]	-0.11572	0.707413	0.633311	0.236818
TG(O-44:0)	TG[O]	0.171731	0.627445	0.343932	0.423243
TG(O-44:1)	TG[O]	-0.2921	0.461094	0.431536	0.337821
TG(O-44:2)	TG[O]	0.057319	0.89035	0.523497	0.291836
TG(O-45:0)	TG[O]	0.029564	0.884379	0.574965	0.342879
TG(O-45:1)	TG[O]	-0.15652	0.661857	0.426648	0.266454
TG(O-45:2)	TG[O]	#N/A	#N/A	0.539325	0.266605
TG(O-46:0)	TG[O]	0.016116	0.983491	0.460418	0.496202

Lipid	LipidClass	Log2FC_noH	PVal_noH	Log2FC_withH	PVal_withH
TG(O-46:1)	TG[O]	-0.36792	0.529638	0.252163	0.463136
TG(O-46:2)	TG[O]	-0.38083	0.39099	0.435912	0.203876
TG(O-46:3)	TG[O]	#N/A	#N/A	0.53033	0.273103
TG(O-47:0)	TG[O]	0.114851	0.760532	0.63046	0.421803
TG(O-47:1)	TG[O]	-0.03749	0.906082	0.270564	0.647596
TG(O-47:2)	TG[O]	#N/A	#N/A	0.515602	0.371891
TG(O-47:3)	TG[O]	#N/A	#N/A	1.11362	0.255528
TG(O-48:0)	TG[O]	0.033515	0.966542	0.612576	0.519444
TG(O-48:1)	TG[O]	-0.21986	0.788193	0.457973	0.491287
TG(O-48:2)	TG[O]	-0.20236	0.751093	0.455897	0.397748
TG(O-48:3)	TG[O]	0.092257	0.857128	0.524033	0.32673
TG(O-48:4)	TG[O]	#N/A	#N/A	0.338162	0.275507
TG(O-48:5)	TG[O]	#N/A	#N/A	0.694828	0.092008
TG(O-49:0)	TG[O]	0.535493	0.142616	0.713487	0.411967
TG(O-49:1)	TG[O]	0.029366	0.941107	0.489808	0.41473
TG(O-49:2)	TG[O]	-0.26726	0.478363	0.492559	0.286631
TG(O-49:3)	TG[O]	#N/A	#N/A	0.557561	0.414944
TG(O-49:4)	TG[O]	#N/A	#N/A	0.729516	0.210082
TG(O-49:5)	TG[O]	#N/A	#N/A	0.686613	0.173634
TG(O-49:6)	TG[O]	#N/A	#N/A	0.773723	0.076636
TG(O-50:0)	TG[O]	0.353246	0.475483	0.755906	0.380273
TG(O-50:1)	TG[O]	-0.07336	0.928969	0.625853	0.39781
TG(O-50:2)	TG[O]	-0.333	0.700213	0.517974	0.356934
TG(O-50:3)	TG[O]	-0.41684	0.574442	0.482883	0.338881
TG(O-50:4)	TG[O]	#N/A	#N/A	0.481259	0.281202
TG(O-50:5)	TG[O]	#N/A	#N/A	0.446026	0.152468
TG(O-50:6)	TG[O]	#N/A	#N/A	0.518807	0.099538
TG(O-50:7)	TG[O]	#N/A	#N/A	0.591214	0.109583
TG(O-50:8)	TG[O]	#N/A	#N/A	0.653811	0.176532
TG(O-51:0)	TG[O]	0.343209	0.286909	0.820307	0.284388
TG(O-51:1)	TG[O]	0.116547	0.751534	0.680028	0.333631
TG(O-51:2)	TG[O]	-0.05101	0.914314	0.563814	0.269763
TG(O-51:3)	TG[O]	-0.17331	0.733229	0.468224	0.292479
TG(O-51:4)	TG[O]	#N/A	#N/A	0.702911	0.257235
TG(O-51:5)	TG[O]	#N/A	#N/A	0.541244	0.236266
TG(O-51:6)	TG[O]	#N/A	#N/A	0.43387	0.4397
TG(O-51:7)	TG[O]	#N/A	#N/A	1.154699	0.173529
TG(O-52:0)	TG[O]	0.347283	0.422542	0.82085	0.24913
TG(O-52:1)	TG[O]	0.104982	0.86285	0.763665	0.251136
TG(O-52:2)	TG[O]	-0.32793	0.717658	0.663377	0.263122
TG(O-52:3)	TG[O]	-0.47823	0.619305	0.608076	0.245404
TG(O-52:4)	TG[O]	-0.50345	0.597885	0.568313	0.292038
TG(O-52:5)	TG[O]	-0.37995	0.641568	0.3797	0.275351
TG(O-52:6)	TG[O]	-0.19999	0.773446	0.381457	0.183209
TG(O-52:7)	TG[O]	#N/A	#N/A	0.559901	0.051529
TG(O-52:8)	TG[O]	#N/A	#N/A	0.439247	0.251701
TG(O-52:9)	TG[O]	#N/A	#N/A	0.56472	0.215325

Lipid	LipidClass	Log2FC_noH	PVal_noH	Log2FC_withH	PVal_withH
TG(O-53:0)	TG[O]	0.008705	0.97642	0.719069	0.255463
TG(O-53:1)	TG[O]	0.037584	0.897759	0.66658	0.217449
TG(O-53:2)	TG[O]	-0.08502	0.889456	0.571901	0.264564
TG(O-53:3)	TG[O]	-0.65895	0.507282	0.57124	0.263625
TG(O-53:4)	TG[O]	-0.70223	0.529424	0.55432	0.272703
TG(O-53:5)	TG[O]	#N/A	#N/A	0.416537	0.206385
TG(O-53:6)	TG[O]	#N/A	#N/A	0.325589	0.4438
TG(O-53:7)	TG[O]	#N/A	#N/A	0.471878	0.329343
TG(O-54:0)	TG[O]	0.30774	0.384484	0.787079	0.284808
TG(O-54:1)	TG[O]	-0.09372	0.865128	0.713921	0.236635
TG(O-54:10)	TG[O]	#N/A	#N/A	0.543073	0.032651
TG(O-54:2)	TG[O]	-0.60054	0.581998	0.735142	0.212791
TG(O-54:3)	TG[O]	-0.91667	0.506551	0.728014	0.273559
TG(O-54:4)	TG[O]	-0.997	0.484735	0.679687	0.324841
TG(O-54:5)	TG[O]	-0.42537	0.656797	0.500933	0.259398
TG(O-54:6)	TG[O]	-0.35494	0.597643	0.407747	0.215882
TG(O-54:7)	TG[O]	-0.31954	0.61333	0.412923	0.275609
TG(O-54:8)	TG[O]	#N/A	#N/A	0.394684	0.316715
TG(O-54:9)	TG[O]	#N/A	#N/A	0.650707	0.044706
TG(O-55:0)	TG[O]	0.318931	0.291958	0.847883	0.23957
TG(O-55:1)	TG[O]	#N/A	#N/A	0.729954	0.229297
TG(O-55:2)	TG[O]	-0.37686	0.491962	0.746558	0.163612
TG(O-55:3)	TG[O]	-1.11327	0.373645	0.684373	0.228004
TG(O-55:4)	TG[O]	#N/A	#N/A	0.692122	0.283539
TG(O-55:5)	TG[O]	-0.64361	0.554165	0.533633	0.262649
TG(O-55:6)	TG[O]	-0.05753	0.920799	0.383191	0.260242
TG(O-55:7)	TG[O]	#N/A	#N/A	0.313985	0.346608
TG(O-55:8)	TG[O]	#N/A	#N/A	0.171891	0.609208
TG(O-56:0)	TG[O]	0.167027	0.691108	0.979473	0.204196
TG(O-56:1)	TG[O]	-0.03585	0.937035	0.820754	0.176207
TG(O-56:10)	TG[O]	#N/A	#N/A	0.578214	0.07554
TG(O-56:11)	TG[O]	#N/A	#N/A	0.767537	0.008685
TG(O-56:12)	TG[O]	#N/A	#N/A	0.492424	0.204765
TG(O-56:2)	TG[O]	-0.52533	0.561617	0.759096	0.185827
TG(O-56:3)	TG[O]	-0.96996	0.457533	0.695776	0.228724
TG(O-56:4)	TG[O]	-1.252	0.464741	0.769859	0.287826
TG(O-56:5)	TG[O]	-0.70248	0.545973	0.621271	0.29546
TG(O-56:6)	TG[O]	-0.36697	0.642169	0.512394	0.224052
TG(O-56:7)	TG[O]	-0.23677	0.697696	0.412912	0.218019
TG(O-56:8)	TG[O]	-0.24001	0.624473	0.350977	0.309563
TG(O-56:9)	TG[O]	#N/A	#N/A	0.453442	0.176088
TG(O-57:0)	TG[O]	#N/A	#N/A	0.77306	0.272812
TG(O-57:1)	TG[O]	0.172284	0.54928	0.807074	0.178124
TG(O-57:2)	TG[O]	-0.15733	0.73258	0.819315	0.13659
TG(O-57:3)	TG[O]	-0.53329	0.530524	0.690706	0.181262
TG(O-57:4)	TG[O]	-0.63954	0.640101	0.675207	0.313048
TG(O-57:5)	TG[O]	#N/A	#N/A	0.611972	0.266608

Lipid	LipidClass	Log2FC_noH	PVal_noH	Log2FC_withH	PVal_withH
TG(O-57:6)	TG[O]	-1.23267	0.458825	0.54657	0.247853
TG(O-57:7)	TG[O]	-0.49102	0.558705	0.348775	0.312036
TG(O-57:8)	TG[O]	#N/A	#N/A	0.410203	0.246917
TG(O-57:9)	TG[O]	#N/A	#N/A	0.141294	0.742555
TG(O-58:0)	TG[O]	0.120615	0.774127	0.946143	0.21469
TG(O-58:1)	TG[O]	-0.04692	0.912522	0.933509	0.149096
TG(O-58:10)	TG[O]	#N/A	#N/A	0.434216	0.129866
TG(O-58:11)	TG[O]	#N/A	#N/A	0.377857	0.208689
TG(O-58:12)	TG[O]	#N/A	#N/A	0.290545	0.441102
TG(O-58:2)	TG[O]	-0.27794	0.658133	0.861532	0.130594
TG(O-58:3)	TG[O]	-0.61092	0.493464	0.834909	0.177434
TG(O-58:4)	TG[O]	-1.03745	0.430788	0.745397	0.239268
TG(O-58:5)	TG[O]	-0.92463	0.476427	0.714453	0.23743
TG(O-58:6)	TG[O]	-0.69517	0.515963	0.570105	0.284265
TG(O-58:7)	TG[O]	-0.48313	0.590504	0.486525	0.321681
TG(O-58:8)	TG[O]	-0.33086	0.650212	0.456224	0.289735
TG(O-58:9)	TG[O]	-0.35892	0.586895	0.416856	0.246845
TG(O-59:0)	TG[O]	#N/A	#N/A	0.508151	0.378914
TG(O-59:1)	TG[O]	-0.00736	0.978729	0.697815	0.244609
TG(O-59:10)	TG[O]	#N/A	#N/A	0.395982	0.221354
TG(O-59:11)	TG[O]	#N/A	#N/A	0.287053	0.384913
TG(O-59:2)	TG[O]	-0.05143	0.902041	0.818328	0.121296
TG(O-59:3)	TG[O]	-1.01086	0.334143	0.693942	0.138175
TG(O-59:4)	TG[O]	#N/A	#N/A	0.878578	0.128094
TG(O-59:5)	TG[O]	#N/A	#N/A	0.743154	0.171498
TG(O-59:6)	TG[O]	#N/A	#N/A	0.65018	0.146726
TG(O-59:7)	TG[O]	#N/A	#N/A	0.407618	0.374272
TG(O-59:8)	TG[O]	#N/A	#N/A	0.608429	0.192645
TG(O-59:9)	TG[O]	#N/A	#N/A	0.367408	0.31605
TG(O-60:0)	TG[O]	#N/A	#N/A	0.852459	0.297679
TG(O-60:1)	TG[O]	0.090312	0.789428	0.963784	0.200273
TG(O-60:10)	TG[O]	#N/A	#N/A	0.473707	0.165673
TG(O-60:11)	TG[O]	#N/A	#N/A	0.38974	0.212759
TG(O-60:12)	TG[O]	#N/A	#N/A	0.344916	0.232648
TG(O-60:13)	TG[O]	#N/A	#N/A	0.155792	0.672419
TG(O-60:2)	TG[O]	-0.29177	0.598826	0.81341	0.207564
TG(O-60:3)	TG[O]	-0.54386	0.494176	0.802921	0.179774
TG(O-60:4)	TG[O]	-0.77155	0.446767	0.882938	0.159697
TG(O-60:5)	TG[O]	-0.54343	0.554287	0.802254	0.136129
TG(O-60:6)	TG[O]	-0.50991	0.488355	0.65318	0.15788
TG(O-60:7)	TG[O]	-0.6636	0.431731	0.597814	0.198504
TG(O-60:8)	TG[O]	#N/A	#N/A	0.553283	0.299789
TG(O-60:9)	TG[O]	-0.59311	0.569126	0.549152	0.218121
TG(O-61:1)	TG[O]	#N/A	#N/A	0.436701	0.45383
TG(O-61:10)	TG[O]	#N/A	#N/A	0.35523	0.365832
TG(O-61:11)	TG[O]	#N/A	#N/A	0.544887	0.26046
TG(O-61:2)	TG[O]	#N/A	#N/A	0.707009	0.239941

Lipid	LipidClass	Log2FC_noH	PVal_noH	Log2FC_withH	PVal_withH
TG(O-61:3)	TG[O]	#N/A	#N/A	0.691447	0.236528
TG(O-61:4)	TG[O]	#N/A	#N/A	0.750794	0.19923
TG(O-61:5)	TG[O]	#N/A	#N/A	0.819566	0.148576
TG(O-61:6)	TG[O]	#N/A	#N/A	0.636203	0.17657
TG(O-61:7)	TG[O]	#N/A	#N/A	0.791386	0.094468
TG(O-61:8)	TG[O]	#N/A	#N/A	0.691722	0.22975
TG(O-61:9)	TG[O]	#N/A	#N/A	0.460703	0.387172
TG(O-62:0)	TG[O]	#N/A	#N/A	1.073854	0.333527
TG(O-62:1)	TG[O]	#N/A	#N/A	0.901729	0.212074
TG(O-62:10)	TG[O]	#N/A	#N/A	0.616833	0.171729
TG(O-62:11)	TG[O]	#N/A	#N/A	0.472868	0.195801
TG(O-62:12)	TG[O]	#N/A	#N/A	0.374813	0.235511
TG(O-62:13)	TG[O]	#N/A	#N/A	0.153509	0.591879
TG(O-62:2)	TG[O]	#N/A	#N/A	0.719928	0.239428
TG(O-62:3)	TG[O]	#N/A	#N/A	0.59039	0.207016
TG(O-62:4)	TG[O]	#N/A	#N/A	0.947984	0.184623
TG(O-62:5)	TG[O]	#N/A	#N/A	0.895724	0.133253
TG(O-62:6)	TG[O]	-0.4982	0.523285	0.806896	0.094074
TG(O-62:7)	TG[O]	-0.83016	0.296494	0.694473	0.113665
TG(O-62:8)	TG[O]	#N/A	#N/A	0.671848	0.142
TG(O-62:9)	TG[O]	#N/A	#N/A	0.634352	0.235813
TG(O-63:1)	TG[O]	#N/A	#N/A	0.959012	0.232488
TG(O-63:10)	TG[O]	#N/A	#N/A	0.035512	0.932015
TG(O-63:2)	TG[O]	#N/A	#N/A	1.483822	0.099208
TG(O-63:3)	TG[O]	#N/A	#N/A	0.84778	0.430091
TG(O-63:4)	TG[O]	#N/A	#N/A	1.172674	0.2536
TG(O-63:5)	TG[O]	#N/A	#N/A	0.891131	0.201063
TG(O-63:6)	TG[O]	#N/A	#N/A	0.911852	0.084674
TG(O-63:7)	TG[O]	#N/A	#N/A	0.699456	0.177378
TG(O-63:8)	TG[O]	#N/A	#N/A	0.857663	0.104145
TG(O-63:9)	TG[O]	#N/A	#N/A	0.61997	0.290178
TG(O-64:0)	TG[O]	#N/A	#N/A	1.244128	0.396677
TG(O-64:1)	TG[O]	#N/A	#N/A	1.235481	0.162034
TG(O-64:10)	TG[O]	#N/A	#N/A	0.665997	0.153399
TG(O-64:11)	TG[O]	#N/A	#N/A	0.578712	0.214084
TG(O-64:12)	TG[O]	#N/A	#N/A	0.384336	0.411512
TG(O-64:2)	TG[O]	#N/A	#N/A	1.250038	0.108837
TG(O-64:3)	TG[O]	#N/A	#N/A	1.105855	0.19039
TG(O-64:4)	TG[O]	#N/A	#N/A	0.997857	0.303331
TG(O-64:5)	TG[O]	#N/A	#N/A	0.934785	0.220822
TG(O-64:6)	TG[O]	#N/A	#N/A	0.761805	0.191161
TG(O-64:7)	TG[O]	#N/A	#N/A	0.717895	0.157399
TG(O-64:8)	TG[O]	#N/A	#N/A	0.768216	0.11387
TG(O-64:9)	TG[O]	#N/A	#N/A	0.71423	0.166325
TG(O-65:10)	TG[O]	#N/A	#N/A	0.904191	0.260589
TG(O-65:11)	TG[O]	#N/A	#N/A	0.310553	0.623126
TG(O-65:5)	TG[O]	#N/A	#N/A	0.859593	0.466263

Lipid	LipidClass	Log2FC_noH	PVal_noH	Log2FC_withH	PVal_withH
TG(O-65:6)	TG[O]	#N/A	#N/A	0.903475	0.257332
TG(O-65:7)	TG[O]	#N/A	#N/A	0.862959	0.173369
TG(O-65:8)	TG[O]	#N/A	#N/A	0.810167	0.157401
TG(O-65:9)	TG[O]	#N/A	#N/A	0.953649	0.217734
TG(O-66:10)	TG[O]	#N/A	#N/A	0.656798	0.186272
TG(O-66:11)	TG[O]	#N/A	#N/A	0.629266	0.16112
TG(O-66:2)	TG[O]	#N/A	#N/A	1.135393	0.16574
TG(O-66:4)	TG[O]	#N/A	#N/A	1.339114	0.257837
TG(O-66:5)	TG[O]	#N/A	#N/A	1.058508	0.23063
TG(O-66:6)	TG[O]	#N/A	#N/A	0.890438	0.257321
TG(O-66:7)	TG[O]	#N/A	#N/A	0.724461	0.265633
TG(O-66:8)	TG[O]	#N/A	#N/A	0.817164	0.162815
TG(O-66:9)	TG[O]	#N/A	#N/A	0.786045	0.174979
TG(O-68:10)	TG[O]	#N/A	#N/A	0.89457	0.176576
TG(O-68:11)	TG[O]	#N/A	#N/A	0.770177	0.115384
TG(O-68:6)	TG[O]	#N/A	#N/A	1.40805	0.2355
TG(O-68:7)	TG[O]	#N/A	#N/A	1.160808	0.204941
TG(O-68:8)	TG[O]	#N/A	#N/A	1.213678	0.127511
TG(O-68:9)	TG[O]	#N/A	#N/A	1.238418	0.109532
