# Supplementary material for: HEPES in Cell Culture Alters the Multi‐Omics Profile Exhibited by Gaucher Disease Fibroblasts
Source: J Cell Biochem. 2026 Jan 16;127(1):e70080. doi: 10.1002/jcb.70080 (PMC12809196; doi:10.1002/jcb.70080)
Supplement: Supplementary file 7 — SupplTbl6_Metabolomics_v3. [file JCB-127-e70080-s008.pdf]

Supplemental Table 6: Metabolomics data

NoH: cultured without HEPES; WithH: cultured with HEPES; all cultures in Ham's F10

Metabolite	Log2FC_NoH	PVal_NoH	Log2FC_ WithH	PVal_WithH	HMDB_name
2-Dehydrogluconate	0.738493	0.045257	-0.16438	0.166732	2-Keto-L-gluconate
2-Dehydrogluconate-6P	n/a	n/a	-0.09151	0.658022	D-Glucuronic acid 1-phosphate
2-Hydroxybutyric acid	n/a	n/a	-0.10717	0.541922	2-Hydroxybutyric acid
2-Hydroxyglutarate	0.141239	0.344478	-0.15447	0.369592	D-2-Hydroxyglutaric acid
2-Hydroxyglutarate	0.141239	0.344478	-0.15447	0.369592	L-2-Hydroxyglutaric acid
2-Hydroxyglutarate	0.141239	0.344478	-0.15447	0.369592	2-Hydroxyglutarate
2-phosphoglyceric acid	0.372291	0.164486	-0.29861	0.052677	2-Phosphoglyceric acid
3-phosphoglyceric acid	0.456646	0.028774	-0.27957	0.079613	3-Phosphoglyceric acid
3-Ureidopropionic acid	n/a	n/a	-0.17873	0.408735	Ureidopropionic acid
6-Phosphogluconolactone	n/a	n/a	0.42043	0.122125	6-Phosphonoglucono-D-lactone
Acetyl-CoA	0.352461	0.356264	0.188427	0.180381	Acetyl-CoA
Acetylglutamic acid	0.283575	0.043493	0.039917	0.738826	N-Acetylglutamic acid
Adenine	-0.29818	0.456343	-0.06011	0.674598	Adenine
Adenosine	0.149375	0.542775	0.327229	0.03573	Adenosine
Adenylthiomethylpentose	0.037471	0.878952	n/a	n/a	5'-Methylthioadenosine
Adipic acid	0.830491	0.268477	n/a	n/a	Adipic acid
ADP	0.21686	0.039823	-0.00609	0.644875	ADP
ADP-Ribose	0.401067	0.079338	-0.06564	0.479344	Adenosine diphosphate ribose
AICAR	0.043171	0.899387	-0.1644	0.082242	AICAR
Alanine	0.350827	0.057402	-0.02662	0.715506	L-Alanine
Allantoin	0.612277	0.059373	-0.16798	0.344863	Allantoin
Alpha-Ketoglutarate	0.264695	0.045931	-0.03442	0.795111	Oxoglutaric acid
Aminoadipic acid	-0.03302	0.898992	-0.3101	0.064382	Aminoadipic acid
AMP	0.711306	0.171001	-0.2768	0.198338	Adenosine monophosphate
Arginine	0.663838	0.051417	-0.09963	0.492416	L-Arginine
Argininosuccinate	0.435439	0.041697	n/a	n/a	Argininosuccinic acid
Asparagine	0.495222	0.142812	0.101625	0.456957	L-Asparagine
Aspartate	0.264213	0.302786	-0.22085	0.04363	L-Aspartic acid
ATP	0.285777	0.148806	0.075808	0.148015	Adenosine triphosphate
beta-Alanine	0.188318	0.191398	-0.07112	0.554032	beta-Alanine
Betaine	0.368893	0.175247	n/a	n/a	Betaine
Carnitine	0.525264	0.008363	n/a	n/a	L-Carnitine
Carnitine (C2:0)	0.802478	0.001873	n/a	n/a	L-Acetylcarnitine
Carnitine (C3:0)	0.761741	0.00486	n/a	n/a	Propionylcarnitine
Carnitine (C4:0)	0.393537	0.016431	n/a	n/a	Butyrylcarnitine
Carnosine	0.26	0.218132	n/a	n/a	Carnosine
CDP	0.730865	0.056974	-0.10132	0.578412	CDP
CDP-choline	0.220712	0.487125	0.48095	0.405964	Citicoline
Cis-Aconitate	n/a	n/a	-0.16268	0.080095	cis-Aconitic acid

Metabolite	Log2FC_NoH	PVal_NoH	Log2FC_ WithH	PVal_WithH	HMDB_name
Citric acid	-0.02996	0.948146	-0.01078	0.918622	Citric acid
CMP	0.464426	0.066609	0.002581	0.989795	Cytidine monophosphate
CoA-Glutathione	0.459504	0.046628	n/a	n/a	CoA-Glutathione
Coenzyme_A	0.250079	0.239918	0.337765	0.20792	Coenzyme A
Creatine	0.647241	0.181534	0.033387	0.786266	Creatine
Creatine-P	0.250353	0.274996	0.392324	0.070893	Phosphocreatine
Creatinine	0.751303	0.043963	-0.09716	0.626364	Creatinine
CTP	0.276751	0.338905	-0.02822	0.795227	Cytidine triphosphate
Cystathionine	2.105944	0.188135	-0.28813	0.416166	Melilotocarpan A
Cysteine	2.132578	0.191222	-0.84016	0.084987	L-Cysteine
Cystine	n/a	n/a	-0.96622	0.225517	L-Cystine
dAMP	0.268664	0.256683	-0.26318	0.068926	Deoxyadenosine monophosphate
Dihydroxyacetone-P	n/a	n/a	-0.02372	0.939797	Dihydroxyacetone phosphate
Erythrose-4-phosphate	n/a	n/a	0.170094	0.367404	D-Erythrose 4-phosphate
FAD	0.10669	0.502994	0.154245	0.160761	FAD
FADH2	n/a	n/a	0.348594	0.483603	FADH
FAICAR	0.641268	0.139477	-0.15743	0.197273	Phosphoribosyl formamidocarboxamide
Folic acid	1.145822	0.154738	-0.1139	0.57967	Folic acid
Fructose-1,6-diphosphate	0.479851	0.123971	0.084035	0.672888	Fructose 1,6-bisphosphate
Fumarate	0.777219	0.282468	-0.08641	0.295188	Fumaric acid
GDP	0.588329	0.04012	-0.23554	0.104308	Guanosine diphosphate
Gluconate	0.717245	0.096049	n/a	n/a	Gluconic acid
Gluconate-6P	0.588039	0.14739	1.030963	0.001118	6-Phosphogluconic acid
Glucose	1.069572	0.065919	-0.12754	0.419657	D-Glucose
Glucose-6P	0.241719	0.281059	-0.07578	0.632457	Glucose 6-phosphate
Glutamate	0.271854	0.102192	-0.15017	0.003698	L-Glutamic acid
Glutamate-5-semialdehyde	-0.24965	0.468345	n/a	n/a	L-Glutamic gamma-semialdehyde
Glutamine	0.366569	0.044938	-0.2187	0.061064	L-Glutamine
Glutathione	0.49353	0.052618	0.19078	0.061423	Glutathione
Glyceraldehyde-3P	n/a	n/a	-0.01959	0.926984	D-Glyceraldehyde 3-phosphate
Glycerate	0.487558	0.314291	-0.01104	0.950869	Glyceric acid
Glycerol-2P	0.270883	0.207501	-0.09248	0.675268	Beta-Glycerophosphoric acid
Glycerol-3P	n/a	n/a	-0.16289	0.407259	Glycerol 3-phosphate
Glycerophosphocholine	0.765144	0.018682	n/a	n/a	Glycerophosphocholine
Glycerophosphoinositol	0.288664	0.155217	-0.00165	0.990763	1-(sn-Glycero-3-phospho)-1D-myo-inositol
Glycine	0.387174	0.070669			Glycine
GMP	0.57284	0.012636	-0.17727	0.385229	Guanosine monophosphate
GTP	0.27444	0.140233	-0.0719	0.052157	Guanosine triphosphate

Metabolite	Log2FC_NoH	PVal_NoH	Log2FC_ WithH	PVal_WithH	HMDB_name
Hexose-P	0.418359	0.238283	-0.00433	0.982995	Galactose 1-phosphate
Hexose-P	0.418359	0.238283	-0.00433	0.982995	Mannose 6-phosphate
Hexose-P	0.418359	0.238283	-0.00433	0.982995	Glucose 1-phosphate
Hexose-P	0.418359	0.238283	-0.00433	0.982995	D-Mannose 1-phosphate
Hippuric acid	1.036427	0.083525	-0.05402	0.828376	Hippuric acid
Homocysteine	2.248981	0.198556	n/a	n/a	Homocysteine
Homocystine	1.171843	0.098852	-0.60417	0.077892	DL-Homocystine
Homocystine	1.171843	0.098852	-0.60417	0.077892	L-Homocystine
Hydroxyphenyllactic acid	0.552096	0.235363	-0.13896	0.537409	Hydroxyphenyllactic acid
Hypoxanthine	1.029878	0.19594	-0.12427	0.493045	Hypoxanthine
IMP	-0.06906	0.759759	-0.07599	0.695955	Inosinic acid
Inosine	0.051275	0.850745	-0.09591	0.665579	Inosine
Isoleucine	0.500313	0.072339	-0.12896	0.377921	L-Isoleucine
Kynurenic acid	n/a	n/a	0.021131	0.920522	Kynurenic acid
Kynurenine	0.608229	0.100806	0.009399	0.915182	L-Kynurenine
Lactate	n/a	n/a	-0.6317	0.368642	L-Lactic acid
Leucine	0.531911	0.068325	-0.08693	0.517416	L-Leucine
Lysine	0.669124	0.045816	-0.10183	0.483138	L-Lysine
Malate	0.45607	0.219458	0.020236	0.84012	L-Malic acid
Malonic acid	n/a	n/a	-0.15533	0.316078	Malonic acid
Methionine	0.387911	0.110289	-0.01432	0.872105	L-Methionine
NAD+	0.268813	0.216528	0.058881	0.570877	NAD
NADH	0.561349	0.091986	0.243821	0.099825	NADH
NADP+	0.319955	0.048062	-0.10522	0.458156	NADP
NADPH	-0.42709	0.075579	0.466325	0.05299	NADPH
Nicotinamide	0.344548	0.343607	n/a	n/a	Nicotinamide
Nitrosoglutathione	0.329126	0.064332	n/a	n/a	S-Nitrosoglutathione
Ophthalmic acid	-0.03037	0.863811	-0.3526	0.022274	Ophthalmic acid
Ornithine	0.580171	0.072562	-0.06835	0.642976	Ornithine
Oxiglutathione	0.393285	0.098229	0.021248	0.966207	Oxidized glutathione
Pantothenic acid	0.039805	0.9212	-0.14502	0.423829	Pantothenic acid
Phenylalanine	0.515289	0.053359	-0.06321	0.611919	L-Phenylalanine
Phosphoenolpyruvate	0.45274	0.238486	0.148677	0.350279	Phosphoenolpyruvic acid
Phosphorylethanolamine	0.410343	0.615584	0.31324	0.002917	O-Phosphoethanolamine
Phosphoserine	1.034495	0.182155	n/a	n/a	Phosphoserine
Proline	0.258324	0.081554	-0.03211	0.754781	L-Proline
Pyridoxine	0.643569	0.079344	n/a	n/a	Pyridoxine
Pyroglutamic acid	0.842372	0.123823	n/a	n/a	Pyroglutamic acid
Pyruvate	1.035194	0.06388	-0.0798	0.667983	Pyruvic acid
D-Ribose	n/a	n/a	-0.19313	0.346386	
Ribose-5P	0.38133	0.332581	n/a	n/a	D-Ribose 5-phosphate
S-adenosyl homocysteine	0.353778	0.075425	n/a	n/a	S-Adenosylhomocysteine
S-adenosyl methionine	0.002992	0.988354	n/a	n/a	S-Adenosylmethionine

Metabolite	Log2FC_NoH	PVal_NoH	Log2FC_ WithH	PVal_WithH	HMDB_name
Saccharopine	0.13936	0.653136	n/a	n/a	Saccharopine
Sedoheptulose-7P	0.130462	0.658525	0.598848	0.065618	D-Sedoheptulose 7-phosphate
Serine	0.645312	0.1016	0.255826	0.268277	L-Serine
Succinate	0.768101	0.109956	-0.08086	0.583498	Succinic acid
Taurine	0.344609	0.16073	0.047537	0.576706	Taurine
Threonine	0.363189	0.141709	-0.05175	0.292304	L-Threonine
Tryptophan	0.446435	0.078448	-0.08181	0.520816	Tryptophan
TTP	0.040143	0.926547	n/a	n/a	Thymidine 5'-triphosphate
Tyrosine	0.459054	0.063297	-0.05004	0.686694	L-Tyrosine
UDP	0.107908	0.52981	-0.2103	0.042432	Uridine 5'-diphosphate
UDP-HexNac	0.38155	0.444319	-0.20599	0.297585	Uridine diphosphate-N-acetylglucosamine
UDP-HexNac	0.38155	0.444319	-0.20599	0.297585	UDP-N-acetyl-D-mannosamine
UDP-HexNac	0.38155	0.444319	-0.20599	0.297585	UDP-N-acetyl-alpha-D-galactosamine
UDP-hexose	-0.03703	0.84502	-0.0396	0.797011	Uridine diphosphate glucose
UDP-hexose	-0.03703	0.84502	-0.0396	0.797011	Uridine diphosphategalactose
UMP	0.756306	0.03036	n/a	n/a	Uridine 5'-monophosphate
Uracil	0.85593	0.170412	0.076191	0.827755	Uracil
Uric acid	1.096266	0.169444	-0.45831	0.093969	Uric acid
Uridine	0.186252	0.641802	-0.05752	0.786288	Uridine
UTP	0.130073	0.523514	0.078818	0.480645	Uridine triphosphate
Valine	0.417501	0.101678	-0.06777	0.561921	L-Valine
Xanthine	0.917058	0.054013	-0.13594	0.409113	Xanthine
Xylulose-5P	n/a	n/a	0.130514	0.144489	Xylulose 5-phosphate
