# Supplementary material for: HEPES in Cell Culture Alters the Multi‐Omics Profile Exhibited by Gaucher Disease Fibroblasts
Source: J Cell Biochem. 2026 Jan 16;127(1):e70080. doi: 10.1002/jcb.70080 (PMC12809196; doi:10.1002/jcb.70080)
Supplement: Supplementary file 8 — SupplTbl7_Proteomics_v3. [file JCB-127-e70080-s003.pdf]

Supplemental Table 7: Proteomics data

NoH: cultured without HEPES; WithH: cultured with HEPES; all cultures in Ham's F10

Protein IDs	Log2FC_NoH	PVal_NoH	Log2FC_WithH	PVal_WithH	Gene
P01023	-0.3646	0.2898	-0.2461	0.0794	A2M
P01023;P20742	-0.3259	0.4073	-0.1674	0.4767	A2M;PZP
Q2M2I8	0.101	0.6385	0.0687	0.6425	AAK1
Q9H7C9	0.1059	0.3528	-0.0096	0.9471	AAMDC
P49588	-0.0307	0.8143	0.0577	0.7114	AARS1
P33527	0.0763	0.6259	0.0342	0.7825	ABCC1
P61221	-0.0907	0.2743	-0.0324	0.5566	ABCE1
Q8NE71	0.0004	0.9955	-0.1287	0.0511	ABCF1
Q9UG63	0.0486	0.6764	0.0218	0.9171	ABCF2
Q9NUQ8	0.0074	0.9554	-0.0489	0.6495	ABCF3
Q9NUJ1	-0.1377	0.1064	-0.1821	0.269	ABHD10
Q96IU4	0.0632	0.5578	-0.0008	0.991	ABHD14B
Q8IZP0;Q9NYB9	-0.2324	0.0216	-0.0688	0.4574	ABI1
Q15018	0.0041	0.9725	-0.1957	0.0614	ABRAXAS2
P09110	0.0845	0.5498	-0.2578	0.107	ACAA1
P42765	-0.1496	0.2132	-0.0163	0.9399	ACAA2
Q9H845	-0.1273	0.1666	-0.046	0.7435	ACAD9
P11310	0.0585	0.6119	-0.0518	0.6218	ACADM
P16219	-0.0395	0.7395	-0.1318	0.6248	ACADS
P45954	-0.027	0.8422	0.1996	0.3733	ACADSB
P49748	0.0848	0.358	0.0837	0.5729	ACADVL
Q15057	-0.1199	0.252	0.2516	0.1504	ACAP2
P24752	-0.1276	0.3939	-0.0776	0.7049	ACAT1
Q9BWD1	-0.2529	0.2527	-0.0715	0.6572	ACAT2
Q9H3P7	-0.1626	0.0512	0.1039	0.2614	ACBD3
P12821	-0.8173	0.047	-0.4907	0.047	ACE
Q9UKV3	0.0279	0.8957	-0.1856	0.1651	ACIN1
P53396	-0.0212	0.7726	0.1775	0.2038	ACLY
P21399	0.1509	0.3917	-0.1611	0.1863	ACO1
Q99798	0.0169	0.7719	-0.0571	0.6335	ACO2
P49753;Q86TX2	-0.3107	0.0751	-0.1103	0.3414	ACOT1;ACOT2
Q9NPJ3	-0.0252	0.7799	0.1151	0.5083	ACOT13
O00154	-0.0208	0.8982	0.2423	0.0402	ACOT7
Q9Y305	-0.0047	0.9563	0.0348	0.7342	ACOT9
Q15067	-0.0218	0.9104	-0.0493	0.7571	ACOX1
P24666	0.1084	0.2808	0.1435	0.0884	ACP1
P11117	0.0059	0.9794	-0.1083	0.3641	ACP2
P33121	-0.089	0.5407	-0.0095	0.945	ACSL1
O95573	-0.2119	0.1548	0.037	0.7669	ACSL3
O60488;O95573	-0.0475	0.837	-0.054	0.775	ACSL3;ACSL4
O60488	-0.2646	0.2803	-0.085	0.2513	ACSL4
P62736;P63267;P68032;P68133	0.078	0.489	0.1325	0.5123	ACTA1
P60709;P62736;P63261;P63267;P68032;P68133;Q562R1	0.1236	0.3457	0.0646	0.7826	ACTA1;ACTBL2

P60709;P62736;P63261;P63267;P68032;P68133;Q6S8J3;Q9BYX7;A5A3E0;Q562R1	0.1555	0.1956	0.1114	0.5327	ACTA1;ACTBL2;POTEF
P60709;P63261;P68032;P68133;Q6S8J3;Q9BYX7;A5A3E0;P0CG38;P0CG39	-0.0255	0.8175	0.0684	0.7092	ACTA1;POTEF
P60709;P63261	0.0393	0.6626	0.1028	0.602	ACTB;ACTG1
Q562R1	0.1351	0.2118	-0.0096	0.9658	ACTBL2
P62736;P63267	0.1876	0.3687	0.0403	0.8722	ACTG2
O96019	0.0444	0.7213	-0.0692	0.3504	ACTL6A
P12814	-0.0217	0.8989	0.2048	0.5042	ACTN1
O43707;P12814;P35609;Q08043	0.1213	0.5565	0.2586	0.4074	ACTN1;ACTN3;ACTN4
O43707;P12814	0.0487	0.768	0.1723	0.56	ACTN1;ACTN4
O43707	0.0706	0.6833	0.0179	0.9279	ACTN4
Q9NZ32	-0.3171	0.1346	0.0737	0.4992	ACTR10
P61163	-0.0266	0.672	0.0395	0.4293	ACTR1A
P42025;P61163	-0.0223	0.7483	0.1356	0.1444	ACTR1A;ACTR1B
P61160	0.0148	0.6669	0.0013	0.9759	ACTR2
P61158	0.0422	0.3059	0.0518	0.3735	ACTR3
Q03154	0.0351	0.7634	0.0572	0.485	ACY1
P00813	-0.0646	0.7277	-0.1735	0.2628	ADA
O14672	-0.2006	0.1291	0.0136	0.928	ADAM10
P78536	-0.0286	0.6873	0.0088	0.9506	ADAM17
P55265	-0.1882	0.119	-0.0035	0.9619	ADAR
P35611	-0.1828	0.2696	-0.0856	0.6854	ADD1
Q9UEY8	-0.1771	0.2411	-0.0334	0.873	ADD3
P48960	-0.0261	0.8749	0.1146	0.5114	ADGRE5
P11766	0.0398	0.6931	-0.0115	0.8996	ADH5
Q15847	-0.187	0.6413	0.0924	0.7535	ADIRF
Q9GZN8	-0.1273	0.3219	0.024	0.839	ADISSP
P55263	-0.0859	0.5078	-0.1055	0.2694	ADK
Q9BRR6	-0.1778	0.3754	0.1268	0.689	ADPGK
Q9NX46	-0.363	0.0087	-0.145	0.0249	ADPRS
Q16186	0.1445	0.3327	0.0486	0.8068	ADRM1
P30566	0.0309	0.7972	0.1164	0.3978	ADSL
P30520	0.0173	0.7963	0.158	0.1038	ADSS2
Q9Y4W6	0.0018	0.9843	0.0629	0.2614	AFG3L2
P20933	0.0033	0.9875	-0.2382	0.136	AGA
P52594	-0.0144	0.9334	-0.2461	0.0415	AGFG1
Q53H12	0.0608	0.6083	-0.153	0.1408	AGK
P35573	-0.1096	0.3623	0.143	0.3119	AGL
Q9UKV8	0.2267	0.176	0.0252	0.8991	AGO2
O00116	0.0611	0.5598	0.0317	0.7388	AGPS
Q6RW13	-0.0509	0.756	-0.0899	0.5374	AGTRAP
P23526	0.1871	0.0632	0.0645	0.3523	AHCY
O43865;Q96HN2	-0.0006	0.9908	0.076	0.1706	AHCYL1
Q09666	0.0154	0.8142	0.0377	0.689	AHNAK
Q8IVF2	-0.0945	0.5658	-0.0507	0.7663	AHNAK2
O95433	0.0742	0.2271	0.012	0.8393	AHSA1

Q96BJ3	-0.0219	0.8439	-0.0751	0.4345	AIDA
O95831	-0.0329	0.7133	-0.1137	0.2998	AIFM1
Q9BRQ8	-0.0831	0.6173	-0.2763	0.0535	AIFM2
Q12904	-0.0057	0.951	-0.0158	0.8207	AIMP1
Q13155	-0.0382	0.7015	0.0078	0.9434	AIMP2
O00170	0.0915	0.2567	-0.0621	0.4529	AIP
P00568	-0.0646	0.5195	-0.0329	0.7333	AK1
P00568;Q9Y6K8	-0.1339	0.2373	-0.0689	0.508	AK1;AK5
P54819	-0.0933	0.2549	-0.0457	0.6935	AK2
Q9UIJ7	-0.0541	0.5703	0.0352	0.7804	AK3
P27144	-0.0752	0.6881	-0.1186	0.4092	AK4
Q02952	-0.2901	0.0274	-0.1171	0.5378	AKAP12
Q9Y2D5	0.1025	0.2829	-0.0228	0.9244	AKAP2
P14550	-0.0557	0.6844	-0.0715	0.57	AKR1A1
P15121	0.0311	0.8018	-0.017	0.7932	AKR1B1
P52895	-0.3157	0.2823	-0.3206	0.4234	AKR1C2
P17516;P42330;P52895;Q04828	-0.1332	0.5041	-0.0023	0.9927	AKR1C2;AKR1C3
P42330	-0.0479	0.851	-0.0708	0.8298	AKR1C3
O43488	-0.2406	0.0553	-0.2545	0.0191	AKR7A2
Q96B36	-0.0788	0.6638	0.3448	0.0401	AKT1S1
P13716	-0.0124	0.913	-0.0512	0.6039	ALAD
P02768	-0.1767	0.441	0.1923	0.2129	ALB
Q13740	0.2331	0.1511	0.1956	0.1818	ALCAM
Q8IZ83	0.0691	0.552	0.229	0.1457	ALDH16A1
P54886	-0.0284	0.7257	0.0004	0.996	ALDH18A1
P47895	0.241	0.4374	-0.0385	0.8758	ALDH1A3
P30837	-0.1707	0.5448	-0.2182	0.3338	ALDH1B1
O75891;Q3SY69	0.1038	0.5626	0.1719	0.4264	ALDH1L1;ALDH1L2
Q3SY69	0.0558	0.8415	0.0863	0.6715	ALDH1L2
P05091	-0.0268	0.9112	0.2218	0.3101	ALDH2
P51648	0.1292	0.5228	-0.1742	0.6662	ALDH3A2
P43353;P48448	-0.0467	0.8122	-0.4066	0.0473	ALDH3B1;ALDH3B2
P30038	0.1225	0.1954	0.0859	0.441	ALDH4A1
P49419	0.1133	0.3652	0.0944	0.3312	ALDH7A1
P49189	-0.128	0.2812	-0.1787	0.0663	ALDH9A1
P04075	0.0963	0.3233	0.055	0.5318	ALDOA
P04075;P09972	0.1353	0.3765	0.1297	0.2369	ALDOA;ALDOC
P09972	0.0036	0.9669	0.104	0.2377	ALDOC
Q86V81	-0.2147	0.4972	-0.1113	0.4047	ALYREF
Q9Y303	-0.1187	0.3619	-0.1152	0.4422	AMDHD2
Q01484	-0.152	0.4073	0.0599	0.8068	ANK2
Q9P2R3	-0.0296	0.6109	-0.0404	0.3981	ANKFY1
Q8IZ07	0.137	0.4933	0.0107	0.9243	ANKRD13A
Q4KMQ2	-0.0356	0.8032	-0.0345	0.7406	ANO6
P39687;O43423	0.0106	0.9252	-0.072	0.3041	ANP32A
P39687;Q92688	-0.1238	0.5467	0.0256	0.9004	ANP32A;ANP32B
Q92688	0.0645	0.4669	-0.0378	0.6057	ANP32B
P15144	-0.1381	0.3947	-0.2502	0.1899	ANPEP

Q9H6X2	0.1616	0.5166	0.0273	0.8621	ANTXR1
P04083	-0.0981	0.388	-0.0847	0.4064	ANXA1
P50995	0.0595	0.4687	0.2033	0.0439	ANXA11
A6NMY6;P07355	-0.0302	0.6234	-0.0552	0.3594	ANXA2
P09525	-0.1641	0.2166	-0.0502	0.7272	ANXA4
P08758	0.0211	0.8397	-0.1012	0.4453	ANXA5
P08133	0.0363	0.653	0.0815	0.2777	ANXA6
P20073	-0.0985	0.2038	-0.0065	0.9324	ANXA7
Q10567	0.0167	0.8579	-0.1413	0.3094	AP1B1
P63010;Q10567	0.0535	0.2573	0.064	0.2474	AP1B1;AP2B1
O43747	-0.0018	0.9775	0.0264	0.6246	AP1G1
Q9BXS5	0.0112	0.8492	-0.0274	0.6215	AP1M1
O95782	0.1073	0.0436	0.0358	0.5323	AP2A1
O94973;O95782	0.1269	0.0988	0.0723	0.2511	AP2A1;AP2A2
O94973	0.0852	0.2983	0.0134	0.9006	AP2A2
P63010	0.0085	0.8436	0.0203	0.6638	AP2B1
Q96CW1	0.0673	0.1437	0.0971	0.0435	AP2M1
P53680	-0.1016	0.3604	0.0335	0.7249	AP2S1
O00203;Q13367	0.0144	0.6621	-0.0203	0.7443	AP3B1
O14617	-0.0121	0.8127	0.0189	0.7299	AP3D1
Q9Y2T2	-0.0204	0.882	0.1025	0.1089	AP3M1
P53677;Q9Y2T2	-0.1435	0.2456	0.0851	0.4456	AP3M1;AP3M2
Q92572	-0.0941	0.2167	-0.1005	0.2707	AP3S1
Q7Z5R6	-0.1275	0.4738	-0.1877	0.2779	APBB1IP
P13798	-0.103	0.2484	-0.2243	0.0617	APEH
P27695	0.0149	0.8789	0.0147	0.8021	APEX1
Q9BZZ5	0.1494	0.2762	-0.1507	0.0852	API5
Q96GX9	-0.0636	0.7343	-0.1092	0.611	APIP
Q9HDC9	0.0756	0.1778	0.0356	0.7574	APMAP
Q9NRW3	0.0309	0.8232	0.0918	0.5263	APOBEC3C
Q9BQE5	0.0202	0.8353	-0.1027	0.3521	APOL2
Q6UXV4	-0.2029	0.2576	0.1802	0.1503	APOOL
Q9UKG1	-0.1977	0.2726	-0.0536	0.5974	APPL1
Q8NEU8	-0.0174	0.853	-0.0988	0.4315	APPL2
P07741	0.0965	0.3218	-0.1545	0.0919	APRT
Q96P48	-0.082	0.5551	0.1619	0.3417	ARAP1
P48444	-0.0291	0.4223	-0.0018	0.9724	ARCN1
P61204;P84077	-0.1035	0.4857	-0.0806	0.2398	ARF1;ARF3
P18085	-0.1386	0.1454	0.0066	0.9607	ARF4
P18085;P61204;P84077;P84085	-0.021	0.797	0.0371	0.6243	ARF4;ARF5
P84085	-0.1926	0.1698	-0.0194	0.8129	ARF5
P62330	0.0075	0.9245	0.0242	0.8016	ARF6
Q9NP61	0.005	0.9634	-0.028	0.7522	ARFGAP3
P53367	-0.0434	0.6045	0.0316	0.5117	ARFIP1
Q07960	-0.1825	0.0795	-0.0068	0.9203	ARHGAP1
Q68EM7	0.0935	0.4366	-0.0331	0.7764	ARHGAP17
Q8N392	0.2051	0.0882	0.0342	0.7957	ARHGAP18
P52565	-0.0395	0.5297	0.0096	0.8438	ARHGDIA

Q92888	-0.1382	0.1445	0.0215	0.7339	ARHGEF1
Q9NZN5	-0.0374	0.7588	0.0367	0.7223	ARHGEF12
Q92974	0.1402	0.4145	0.0334	0.8267	ARHGEF2
Q14155	0.1991	0.2984	-0.1787	0.3503	ARHGEF7
Q9Y4X5	-0.2657	0.0489	0.2087	0.3924	ARIH1
P40616	0.0688	0.5684	-0.1132	0.4957	ARL1
P36405	-0.1035	0.3231	0.023	0.8575	ARL3
O75915	-0.1107	0.3211	-0.0427	0.8205	ARL6IP5
Q8N6S5	-0.2947	0.2443	-0.2459	0.2166	ARL6IP6
Q96BM9	-0.2747	0.2336	0.1847	0.5172	ARL8A
Q96BM9;Q9NVJ2	-0.2553	0.0604	-0.1389	0.2448	ARL8A;ARL8B
Q9NVJ2	-0.1593	0.075	0.0334	0.3924	ARL8B
Q9NVT9	0.2125	0.1663	0.0913	0.4358	ARMC1
Q8IUR7	-0.0886	0.6051	-0.0263	0.9044	ARMC8
Q9UH62	-0.1796	0.0771	-0.034	0.5685	ARMCX3
Q92747	0.0649	0.7054	0.1013	0.411	ARPC1A
O15143	0.0451	0.3103	-0.0276	0.6927	ARPC1B
O15144	0.032	0.4837	0.0268	0.5941	ARPC2
O15145	0.0287	0.7471	0.0178	0.8216	ARPC3
P59998	0.006	0.8699	-0.0161	0.6932	ARPC4
O15511	-0.0171	0.66	-0.0458	0.5679	ARPC5
Q9BPX5	-0.0409	0.7619	-0.1277	0.0709	ARPC5L
P15289	0.4537	0.094	0.1936	0.3837	ARSA
P15848	0.2725	0.1876	0.2137	0.3868	ARSB
Q13510	0.1723	0.3768	-0.0539	0.7174	ASAH1
P04424	0.225	0.0706	0.1399	0.2503	ASL
P08243	0.0191	0.954	-0.0426	0.942	ASNS
Q12797	-0.0261	0.8481	-0.018	0.8733	ASPH
P00966	0.3434	0.3535	-0.0318	0.8722	ASS1
Q8NBU5	-0.0339	0.8512	-0.0756	0.3137	ATAD1
Q5T9A4;Q9NVI7	0.0257	0.8127	0.0141	0.9058	ATAD3A
Q9NT62	0.105	0.2081	-0.0125	0.8747	ATG3
O95352	-0.0427	0.7665	0.1371	0.3511	ATG7
P31939	-0.107	0.045	-0.1146	0.0038	ATIC
Q6DD88	-0.0428	0.4329	0.0745	0.1128	ATL3
O00244	-0.0147	0.8712	0.0678	0.5363	ATOX1
Q9HD20	0.14	0.2825	0.031	0.6859	ATP13A1
P05023	-0.0414	0.7703	-0.1128	0.4105	ATP1A1
P54709	-0.1099	0.4694	-0.2161	0.2535	ATP1B3
P16615	0.0661	0.3966	0.0227	0.6603	ATP2A2
P20020	-0.1013	0.2794	-0.1146	0.3724	ATP2B1
P20020;P23634;Q01814;Q16720	0.0115	0.945	-0.0491	0.6864	ATP2B1;ATP2B2;ATP2B4
P20020;P23634	-0.0947	0.6321	-0.0903	0.5409	ATP2B1;ATP2B4
P23634;Q01814;Q16720	0.1882	0.3538	-0.0362	0.8543	ATP2B2;ATP2B4
P23634	0.0256	0.9016	0.0142	0.9265	ATP2B4
P25705	0.0016	0.9786	-0.0519	0.5275	ATP5F1A
P06576	0.0087	0.8877	-0.0592	0.5118	ATP5F1B
P36542	0.0452	0.4559	-0.0361	0.6192	ATP5F1C

P30049	-0.0019	0.984	-0.0929	0.3836	ATP5F1D
P56385	0.0887	0.4359	0.0228	0.7877	ATP5ME
P56134	-0.0794	0.5012	0.0836	0.4874	ATP5MF
O75964	0.0369	0.537	-0.0157	0.8817	ATP5MG
Q96IX5	0.2814	0.0172	0.0379	0.7296	ATP5MK
P24539	-0.0193	0.8045	-0.0763	0.4294	ATP5PB
O75947	0.0691	0.2729	-0.0278	0.7857	ATP5PD
P18859	0.2399	0.2674	0.1027	0.6531	ATP5PF
P48047	0.0435	0.4884	0.0031	0.9736	ATP5PO
Q15904	-0.0893	0.5521	0.0365	0.6908	ATP6AP1
O75787	-0.108	0.4171	-0.1161	0.1745	ATP6AP2
Q93050	-0.0436	0.724	-0.1756	0.1259	ATP6V0A1
P27449	0.1016	0.4576	-0.0183	0.9172	ATP6V0C
P61421	0.1476	0.099	0.0651	0.2669	ATP6V0D1
P38606	-0.0432	0.5688	-0.0554	0.4522	ATP6V1A
P21281	0.0787	0.1804	-0.0325	0.6894	ATP6V1B2
P21283	-0.0619	0.519	0.0691	0.5556	ATP6V1C1
P36543	0.0696	0.4643	0.0207	0.7397	ATP6V1E1
O75348	0.1223	0.148	0.0068	0.9362	ATP6V1G1
Q9UI12	0.0885	0.414	-0.1268	0.2641	ATP6V1H
Q8WWM7	-0.0207	0.7642	0.0462	0.5329	ATXN2L
P61769	-0.1676	0.334	-0.2986	0.3197	B2M
Q9NWV8	0.0535	0.5835	-0.0262	0.7609	BABAM1
O95816	0.1433	0.2321	-0.1454	0.3089	BAG2
O95817	0.0576	0.6736	0.1138	0.0873	BAG3
P46379	-0.0647	0.468	-0.0097	0.9375	BAG6
O75531	0.2133	0.1432	-0.1168	0.4693	BANF1
P80723	-0.1591	0.2532	-0.3012	0.125	BASP1
Q07812	-0.0309	0.7845	-0.0915	0.2833	BAX
P51572	0.0396	0.668	-0.0645	0.3212	BCAP31
P12694	0.1338	0.4478	0.0311	0.888	BCKDHA
Q9BXK5	0.08	0.6167	-0.1676	0.3796	BCL2L13
Q9NYF8	0.1116	0.3948	-0.1689	0.2018	BCLAF1
Q9BUT1	0.1624	0.2184	0.1451	0.2416	BDH2
O15155	-0.1184	0.5241	0.0438	0.8106	BET1
Q9H694	0.585	0.0302	-0.0445	0.7557	BICC1
O00499	-0.2264	0.2363	0.0386	0.8002	BIN1
Q6QNY1	0.2092	0.2747	0.1312	0.5703	BLOC1S2
Q6QNY0	-0.0797	0.5903	0.0644	0.6545	BLOC1S3
Q6BDS2	-0.0931	0.8049	-0.0731	0.4637	BLTP3A
P53004	-0.0952	0.4724	-0.0961	0.2777	BLVRA
P30043	-0.0653	0.5566	-0.1181	0.4189	BLVRB
Q9NSY1	0.1598	0.458	-0.2833	0.2823	BMP2K
Q9H3K6	-0.033	0.581	0.0741	0.3541	BOLA2
O95861	-0.0527	0.5775	0.0912	0.1212	BPNT1
Q9NX62	-0.0935	0.4812	0.008	0.9705	BPNT2
Q8TDN6	0.1211	0.4812	-0.1383	0.3589	BRIX1
Q5VW32	0.1522	0.1555	-0.0829	0.6233	BROX

P35613	0.0594	0.6715	0.0328	0.8506	BSG
Q10588	0.1033	0.5641	0.1998	0.5493	BST1
P20290	-0.1158	0.1806	0.1135	0.0971	BTF3
Q96K17	-0.0145	0.9279	0.0834	0.5153	BTF3L4
O43684	-0.1017	0.29	-0.11	0.0603	BUB3
Q7L1Q6	0.1159	0.1978	0.1599	0.1263	BZW1
Q9H0W9	-0.2568	0.0211	-0.2826	0.1267	C11orf54
Q9H3H3	0.0145	0.8153	-0.0276	0.7436	C11orf68
Q9H425	-0.2319	0.4488	0.2977	0.5735	C1orf198
Q07021	0.0814	0.3083	-0.0251	0.8623	C1QBP
P0C0L4;P0C0L5	-0.4557	0.0665	-0.2133	0.1258	C4A;C4B
Q9Y376	-0.0738	0.7157	-0.2281	0.1526	CAB39
Q9Y376;Q9H9S4	0.2599	0.1641	0.0383	0.7928	CAB39;CAB39L
P54289	0.1651	0.2804	0.1403	0.4026	CACNA2D1
Q9HB71	-0.1496	0.1904	0.0389	0.6783	CACYBP
P27708	-0.0055	0.9431	-0.0461	0.3817	CAD
Q05682	0.1432	0.7131	0.2152	0.6345	CALD1
P0DP23;P0DP24;P0DP25	-0.0827	0.3124	-0.0921	0.1904	CALM1;CALM2;CALM3
P27797	0.0113	0.8316	-0.0748	0.4081	CALR
O43852	0.0318	0.75	-0.0142	0.9133	CALU
Q14012;Q8IU85	-0.162	0.3226	0.1905	0.2331	CAMK1
Q13557	-0.1161	0.3972	-0.003	0.9833	CAMK2D
Q13554;Q13555;Q13557	-0.1063	0.4948	0.0994	0.4677	CAMK2D;CAMK2G
Q86VP6	0.0766	0.143	-0.0031	0.9535	CAND1
P27824	-0.0486	0.3251	-0.1289	0.0657	CANX
Q01518	-0.0059	0.9344	0.106	0.2946	CAP1
P40123	0.0818	0.6791	-0.0363	0.921	CAP2
P40121	-0.4741	0.0396	-0.3615	0.1266	CAPG
P07384	-0.0141	0.8566	0.0021	0.9816	CAPN1
P17655	-0.1453	0.2696	-0.1262	0.2887	CAPN2
P04632	-0.0444	0.7148	-0.0245	0.8213	CAPNS1
Q14444	0.036	0.5192	0.0346	0.5954	CAPRIN1
P52907	-0.0852	0.1799	-0.0704	0.2206	CAPZA1
P47755;P52907	0.0125	0.7887	-0.009	0.8888	CAPZA1;CAPZA2
P47755	-0.0643	0.3042	-0.0623	0.1498	CAPZA2
P47756	0.014	0.6825	-0.0271	0.5719	CAPZB
Q9Y2V2	0.0759	0.5632	-0.0872	0.5105	CARHSP1
Q86X55	-0.1365	0.1736	-0.0365	0.7248	CARM1
P49589	0.2058	0.1173	0.0698	0.633	CARS1
Q9HA77	0.0664	0.6324	-0.0354	0.7977	CARS2
P42574	-0.0896	0.5122	0.0978	0.2796	CASP3
P20810	-0.086	0.2838	-0.0325	0.5308	CAST
P04040	-0.1099	0.5699	-0.2374	0.1391	CAT
Q03135	0.0304	0.8393	-0.1326	0.5348	CAV1
P51636	-0.1964	0.3555	-0.1768	0.3938	CAV2
Q6NZI2	0.0273	0.7949	-0.0739	0.6603	CAVIN1
O95810	0.2226	0.5122	-0.0685	0.7969	CAVIN2
Q969G5	0.0117	0.9028	-0.2358	0.2771	CAVIN3

P16152	0.0627	0.5248	0.0833	0.4988	CBR1
O75828;P16152	-0.0322	0.8171	0.0363	0.7268	CBR1;CBR3
O75828	-0.0164	0.904	0.0364	0.7867	CBR3
Q13185	0.1288	0.2795	-0.1526	0.139	CBX3
Q6P1N0	0.0046	0.9875	0.0686	0.8547	CC2D1A
Q8IX12	0.0142	0.8847	-0.0247	0.8128	CCAR1
Q8N163	0.1754	0.1453	-0.1203	0.3188	CCAR2
Q96CT7	-0.0949	0.3188	-0.1207	0.3738	CCDC124
O60826	-0.0575	0.3894	-0.145	0.0185	CCDC22
Q96A33	-0.1206	0.3831	-0.0506	0.6085	CCDC47
Q8IVM0	0.0159	0.9069	0.1886	0.1198	CCDC50
Q16204	-0.093	0.4367	-0.1877	0.0424	CCDC6
Q9Y6R9	-0.1623	0.7675	-0.18	0.4659	CCDC61
Q567U6	-0.0496	0.6934	-0.4507	0.038	CCDC93
P78371	0.0125	0.7472	-0.0103	0.7702	CCT2
P49368	-0.0058	0.8605	-0.0145	0.729	CCT3
P50991	0.0029	0.9647	0.0233	0.6385	CCT4
P48643	-0.0033	0.9453	-0.011	0.7667	CCT5
P40227	-0.0349	0.3845	0.0515	0.3919	CCT6A
P40227;Q92526	-0.1088	0.0361	0.0312	0.4204	CCT6A;CCT6B
Q99832	-0.0313	0.3933	0.0488	0.381	CCT7
P50990	-0.0143	0.7106	0.0274	0.4297	CCT8
Q5ZPR3	-0.2259	0.1551	-0.1009	0.492	CD276
P16070	-0.105	0.4209	-0.0604	0.7028	CD44
Q08722	-0.323	0.0965	-0.4063	0.1283	CD47
P13987	-0.2865	0.1723	-0.2158	0.1498	CD59
P08962	-0.1962	0.3666	-0.3008	0.1665	CD63
P60033	-0.1836	0.1531	-0.1504	0.3429	CD81
P21926	-0.0591	0.819	0.091	0.6607	CD9
P14209	-0.0797	0.7207	-0.2108	0.4629	CD99
Q16543	0.0544	0.2996	0.0388	0.4306	CDC37
P60953	0.0153	0.7865	-0.0082	0.8938	CDC42
P15153;P17081;P60763;P60953;P63000;P84095;Q9H4E5	0.057	0.7342	-0.0246	0.7881	CDC42;RAC1;RHOG
Q9Y5S2	-0.1011	0.1653	-0.0218	0.7622	CDC42BPB
Q99459	-0.251	0.1343	-0.2028	0.319	CDC5L
O14735	0.0911	0.4074	0.0518	0.6113	CDIPT
P11802	0.1294	0.31	-0.1151	0.3106	CDK4
P42771	-0.188	0.584	0.0827	0.7852	CDKN2A
Q9UKY7	0.1061	0.3798	-0.0589	0.7361	CDV3
O95319	0.0371	0.8073	-0.1159	0.6341	CELF2
Q5SW79	0.0312	0.7883	0.0392	0.7347	CEP170
Q8TEP8	0.3308	0.4986	0.3245	0.1327	CEP192
Q5T4B2	-0.0833	0.5644	0.0483	0.6851	CERCAM
Q9Y5P4	0.1352	0.425	0.1344	0.5129	CERT1
P23528	0.0001	0.9989	0.0189	0.858	CFL1
P23528;Q9Y281	0.1934	0.4192	-0.0147	0.9522	CFL1;CFL2
Q9Y281	0.1194	0.1644	-0.0211	0.8719	CFL2

Q9Y6H1;Q5T1J5	-0.1542	0.6996	0.3804	0.1543	CHCHD2;CHCHD2P9
Q9NX63	0.0387	0.6151	-0.0532	0.5114	CHCHD3
Q14839	-0.1185	0.4865	-0.0479	0.6187	CHD4
Q9BWS9	0.0631	0.661	-0.0572	0.6823	CHID1
Q9HD42	-0.1587	0.297	0.0198	0.829	CHMP1A
Q9Y3E7	-0.0326	0.6258	0.0564	0.4472	CHMP3
Q9BY43	0.0765	0.665	0.0004	0.998	CHMP4A
Q9H444	0.0042	0.9576	-0.1978	0.0006	CHMP4B
Q9NZZ3	0.0326	0.8452	-0.067	0.6601	CHMP5
Q96FZ7	-0.059	0.6459	-0.1042	0.3749	CHMP6
Q9UHD1	0.0218	0.9196	0.0658	0.6269	CHORDC1
Q99653	-0.1036	0.6113	-0.1437	0.4759	CHP1
Q9Y3Y2	0.082	0.6269	0.1771	0.1945	CHTOP
Q6FI81	-0.0039	0.9837	0.0738	0.6075	CIAPIN1
Q14011	0.1029	0.4692	-0.0058	0.9596	CIRBP
Q9NZ45	-0.1196	0.6243	-0.0846	0.5769	CISD1
Q8N5K1	-0.045	0.6974	-0.1974	0.0883	CISD2
Q07065	0.0433	0.5537	0.0258	0.7411	CKAP4
Q14008	-0.0463	0.4867	-0.0225	0.7471	CKAP5
P12277	0.0979	0.8127	0.1994	0.3399	CKB
O00299	-0.0104	0.8215	-0.007	0.9237	CLIC1
Q9Y696	-0.0523	0.7393	0.1804	0.4161	CLIC4
Q14677	0.0033	0.9688	0.0906	0.4571	CLINT1
P30622	0.083	0.4151	0.1042	0.3907	CLIP1
P54105	-0.2748	0.0827	-0.0706	0.5735	CLNS1A
Q16740	-0.019	0.8473	0.0569	0.6678	CLPP
P09496	-0.0334	0.5945	0.0022	0.9713	CLTA
P09497	0.0005	0.9961	0.0367	0.4225	CLTB
Q00610	0.0096	0.8307	-0.0164	0.6683	CLTC
P53675;Q00610	0.0045	0.908	0.0068	0.8737	CLTC;CLTCL1
P53675	-0.0887	0.5875	0.2804	0.1295	CLTCL1
P30085	-0.0016	0.9891	0.1695	0.0336	CMPK1
P62633	-0.096	0.578	-0.3027	0.1838	CNBP
Q96KP4	-0.1025	0.5082	-0.0056	0.964	CNDP2
Q99439	0.5101	0.0964	0.3019	0.4651	CNN2
Q15417;Q99439	0.5551	0.1801	0.3957	0.5115	CNN2;CNN3
Q15417	0.2845	0.3323	0.3181	0.4856	CNN3
A5YKK6	0.1951	0.1068	0.0273	0.6886	CNOT1
P09543	-0.1139	0.1966	-0.2214	0.0334	CNP
Q9Y2B0	-0.0224	0.8648	0.0016	0.9857	CNPY2
Q9BT09	-0.2067	0.0856	0.0055	0.9724	CNPY3
Q96F85	-0.1117	0.3956	-0.0944	0.5563	CNRIP1
P78357	-0.0506	0.7191	-0.052	0.7931	CNTNAP1
Q5JTJ3	-0.0538	0.7439	-0.0102	0.9638	COA6
Q13057	-0.0959	0.5638	0.2003	0.2419	COASY
Q99715	0.1752	0.521	-0.2364	0.6589	COL12A1
Q05707	-0.381	0.1546	0.3655	0.126	COL14A1
P02452	0.1847	0.7032	-0.0362	0.9331	COL1A1

P08123	-0.1118	0.6973	-0.0453	0.8789	COL1A2
P12109	-0.0509	0.8325	-0.0169	0.9374	COL6A1
P12110	-0.128	0.5771	-0.2131	0.2546	COL6A2
Q8NBJ5	-0.1055	0.3211	0.0224	0.7853	COLGALT1
Q9UBI1	-0.0122	0.9594	0.1449	0.5294	COMMD3
Q9H0A8	-0.131	0.1891	-0.2443	0.1102	COMMD4
Q7Z4G1	-0.0341	0.7459	-0.0847	0.2751	COMMD6
Q86VX2	0.1527	0.3602	-0.1893	0.1081	COMMD7
P21964	-0.0492	0.5805	-0.0213	0.8058	COMT
P53621	-0.0618	0.112	-0.0623	0.1473	COPA
P53618	-0.0059	0.8151	-0.0163	0.7901	COPB1
P35606	-0.0165	0.6611	0.0048	0.9209	COPB2
O14579	-0.0682	0.2707	-0.0779	0.234	COPE
Q9Y678	-0.1444	0.0076	-0.0857	0.1301	COPG1
Q9UBF2;Q9Y678	-0.0697	0.449	-0.1149	0.3743	COPG1;COPG2
Q9UBF2	0.2198	0.232	0.2743	0.1672	COPG2
P61201	0.0212	0.8816	0.0706	0.5258	COPS2
Q9UNS2	0.0207	0.8309	0.019	0.7965	COPS3
Q9BT78	0.0068	0.9064	0.0329	0.3651	COPS4
Q92905	0.2057	0.0326	0.0109	0.9174	COPS5
Q7L5N1	0.1906	0.1807	-0.1515	0.0644	COPS6
Q9UBW8	-0.2966	0.1481	0.0423	0.7219	COPS7A
Q99627	-0.0115	0.9004	0.0891	0.5351	COPS8
P61923	0.0147	0.9274	-0.1004	0.3514	COPZ1
Q9BR76	-0.0586	0.2821	-0.0203	0.6506	CORO1B
Q9ULV4	0.0463	0.6903	0.0541	0.63	CORO1C
Q14019	0.0775	0.3152	0.2896	0.0013	COTL1
P13073	0.063	0.4099	-0.0028	0.9815	COX4I1
P20674	-0.0681	0.615	-0.0559	0.7637	COX5A
P10606	-0.013	0.8739	0.1033	0.3498	COX5B
P14854	-0.0929	0.4291	0.0054	0.9648	COX6B1
P14406	0.1276	0.4067	0.1991	0.2479	COX7A2
O75976	0.0962	0.4473	0.2202	0.1855	CPD
Q99829	0.0354	0.7826	0.0669	0.5967	CPNE1
O75131	-0.0042	0.9643	0.0001	0.9995	CPNE3
P36551	0.0984	0.4325	-0.0203	0.9056	CPOX
Q9BRF8	0.123	0.4894	0.0506	0.78	CPPED1
Q9Y646	0.0475	0.7769	-0.2611	0.2058	CPQ
Q16630	-0.0847	0.447	0.0187	0.8115	CPSF6
Q8N684	0.0487	0.7041	-0.0006	0.9928	CPSF7
P50416	0.0012	0.9894	0.04	0.5262	CPT1A
P29373	-0.2103	0.4789	-0.1028	0.7256	CRABP2
P43155	-0.0945	0.2007	0.1583	0.1976	CRAT
P52943	-0.0886	0.7316	0.0605	0.7875	CRIP2
P46108	-0.1069	0.1841	0.0205	0.7821	CRK
P46109	0.0357	0.3511	0.0526	0.25	CRKL
Q5TZA2	-0.8457	0.044	-0.1272	0.2274	CROCC
O75718	-0.0258	0.8279	-0.1123	0.2615	CRTAP

Q08257	0.1119	0.4695	0.1831	0.327	CRYZ
O75390	-0.0034	0.9568	-0.0259	0.8592	CS
O75534	0.0875	0.3867	-0.0074	0.9476	CSDE1
P55060	0.0317	0.6371	0.0533	0.4574	CSE1L
P41240	0.0292	0.7267	-0.0805	0.3523	CSK
P48729;Q8N752	0.1226	0.3861	-0.086	0.7547	CSNK1A1
P68400;Q8NEV1	-0.0193	0.8483	0.1314	0.0851	CSNK2A1
P19784	0.2524	0.0289	0.0611	0.6947	CSNK2A2
P67870	-0.1451	0.2065	-0.1395	0.2598	CSNK2B
P21291	-0.286	0.5636	0.2239	0.6623	CSRP1
P04080	0.0188	0.8115	0.057	0.5012	CSTB
P33240;Q9H0L4	0.064	0.7282	-0.0682	0.6605	CSTF2
Q13363	-0.1612	0.315	-0.042	0.7341	CTBP1
Q96CG8	-0.1201	0.7355	0.2142	0.5248	CTHRC1
P35221	0.0318	0.7308	0.2002	0.3612	CTNNA1
P26232;P35221	-0.0197	0.8816	0.2257	0.4069	CTNNA1;CTNNA2
P35222	-0.0692	0.6033	0.0528	0.7675	CTNNB1
P14923;P35222	0.3407	0.1812	0.3832	0.2463	CTNNB1;JUP
O60716	0.2114	0.1858	0.1429	0.509	CTNND1
P17812;Q9NRF8	0.076	0.7296	0.0533	0.7931	CTPS1;CTPS2
P10619	0.0533	0.6328	-0.0476	0.8393	CTSA
P07858	0.019	0.798	-0.1098	0.437	CTSB
P07339	-0.2582	0.1242	-0.0678	0.6346	CTSD
P43235	-0.2667	0.4439	-0.2698	0.554	CTSK
P07711	0.2206	0.2156	-0.202	0.287	CTSL
Q9UBR2	0.1979	0.2897	0.0379	0.822	CTSZ
Q14247	0.0535	0.4927	0.0017	0.9639	CTTN
Q13617	0.1413	0.2488	0.1834	0.0187	CUL2
Q13618	-0.0602	0.6592	-0.0074	0.8725	CUL3
Q13619;Q13620	0.0369	0.8311	0.1055	0.5439	CUL4A;CUL4B
Q13620	0.1989	0.2038	0.0112	0.9386	CUL4B
Q93034	-0.0294	0.8538	0.0761	0.6393	CUL5
O60888	0.1103	0.3242	-0.065	0.5103	CUTA
P00167	0.2853	0.1413	-0.2957	0.2743	CYB5A
O43169	-0.1039	0.2002	-0.0667	0.4646	CYB5B
Q9UHQ9	-0.1609	0.0221	-0.2145	0.1545	CYB5R1
P00387	-0.0566	0.5127	0.0394	0.7161	CYB5R3
Q53TN4	-0.1487	0.4195	-0.2217	0.3922	CYBRD1
P08574	0.1922	0.2423	0.03	0.7671	CYC1
P99999	-0.1889	0.3952	-0.1551	0.4215	CYCS
Q7L576	-0.0784	0.2849	-0.0068	0.902	CYFIP1
Q7L576;Q96F07	0.0366	0.6925	0.0257	0.7092	CYFIP1;CYFIP2
Q6UW02	-0.0623	0.7046	0.1107	0.3269	CYP20A1
Q16850	-0.0526	0.6427	0.3336	0.0729	CYP51A1
Q9NUQ9;Q9H0Q0	0.1621	0.1894	0.3231	0.0173	CYRIB
Q9NWV4	-0.0694	0.3311	-0.0148	0.8559	CZIB
P98082	0.2176	0.0111	0.0504	0.7701	DAB2
P61803	0.0854	0.237	-0.1261	0.1166	DAD1

Q14118	-0.0665	0.5365	-0.1614	0.2086	DAG1
P14868	-0.0387	0.4865	0.0493	0.5407	DARS1
Q96EP5	0.1641	0.3001	-0.0685	0.5523	DAZAP1
P07108	-0.2484	0.1844	-0.2596	0.2171	DBI
Q16643	0.0591	0.6625	0.2273	0.125	DBN1
Q9UJU6	0.0843	0.1262	0.001	0.9871	DBNL
Q5TAQ9	-0.1064	0.3613	-0.0108	0.9175	DCAF8
O15075	-0.4096	0.0522	-0.2388	0.5288	DCLK1
P32321	0.1701	0.1498	0.2461	0.055	DCTD
Q14203	-0.0351	0.5265	0.0147	0.7364	DCTN1
Q13561	-0.0857	0.1613	-0.0066	0.9059	DCTN2
Q9UJW0	-0.0692	0.4486	0.0041	0.9638	DCTN4
Q9BTE1	0.071	0.7445	0.1959	0.1723	DCTN5
O94760	-0.1124	0.3856	-0.2383	0.1934	DDAH1
O95865	-0.2194	0.1709	-0.0595	0.6306	DDAH2
Q16531	0.0255	0.6101	-0.0323	0.589	DDB1
P39656	-0.0319	0.5645	-0.0678	0.0086	DDOST
Q16832	-0.0491	0.6985	0.0797	0.6141	DDR2
Q96HY6	0.0723	0.62	-0.1654	0.3963	DDRGK1
P30046;A6NHG4	-0.147	0.2097	-0.1041	0.4391	DDT
Q92499	0.0015	0.9761	0.0081	0.8149	DDX1
Q92841	0.0517	0.5629	-0.0295	0.6795	DDX17
O00571;O15523;P17844;Q92841	0.0374	0.6914	0.0625	0.3206	DDX17;DDX3X;DDX5
P17844;Q92841	-0.0557	0.4504	0.0054	0.9103	DDX17;DDX5
Q9NUU7;Q9UMR2	-0.0721	0.5285	-0.154	0.1239	DDX19A
Q9NR30	0.2567	0.059	-0.0113	0.9494	DDX21
Q9BUQ8	-0.175	0.2181	-0.08	0.697	DDX23
O00148;Q13838	-0.0198	0.8093	-0.0049	0.8996	DDX39A;DDX39B
Q13838	0.0525	0.5529	0.0144	0.8424	DDX39B
O00571;O15523;Q9NQI0	0.0512	0.577	0.013	0.8941	DDX3X
Q86XP3	-0.1369	0.2947	-0.109	0.419	DDX42
Q7L014	0.0259	0.709	-0.0155	0.8091	DDX46
P17844	-0.0146	0.8261	-0.0649	0.4164	DDX5
P26196	-0.0213	0.7076	-0.0277	0.6571	DDX6
Q16698	0.1244	0.3007	0.026	0.8756	DECR1
O15121	-0.0001	0.9997	0.2334	0.2509	DEGS1
P35659	0.0394	0.6481	-0.0136	0.8844	DEK
Q8TCE6	0.1054	0.477	-0.3154	0.0104	DENND10
O43583	-0.0931	0.2099	0.1051	0.465	DENR
Q9Y315	0.0462	0.7087	-0.0652	0.6915	DERA
Q9BUN8	-0.0341	0.7831	-0.0913	0.4342	DERL1
P08670;P17661	-0.1368	0.3782	-0.1485	0.3631	DES;VIM
P23743	0.0647	0.6699	0.219	0.2153	DGKA
Q9UBM7	-0.1438	0.4105	0.3347	0.2312	DHCR7
Q9BTZ2;P0CG22	-0.0042	0.9554	-0.1906	0.1979	DHRS4
Q9Y394	-0.0344	0.8454	-0.1496	0.0682	DHRS7
Q6IAN0	0.0884	0.6769	-0.0566	0.6722	DHRS7B
O43143	0.0018	0.9828	-0.0262	0.5472	DHX15

Q6P158;Q7Z478	0.0484	0.7578	0.0688	0.5768	DHX29
Q7L2E3	-0.1581	0.4748	-0.0886	0.5209	DHX30
Q08211	-0.0562	0.5755	-0.0056	0.9262	DHX9
Q9NR28	-0.0356	0.7125	-0.0541	0.5909	DIABLO
O60610	0.0171	0.8834	0.0528	0.6328	DIAPH1
Q9Y2L1	-0.1796	0.2367	0.1217	0.3521	DIS3
O60832	0.0498	0.7375	0.0507	0.748	DKC1
P10515	-0.2969	0.4286	0.1449	0.4059	DLAT
P09622	-0.0177	0.8419	-0.0447	0.7299	DLD
P36957	-0.213	0.0524	-0.0084	0.9598	DLST
P31689	-0.0299	0.7444	-0.0497	0.6219	DNAJA1
O60884	-0.021	0.6514	-0.0503	0.5387	DNAJA2
Q96EY1	0.0134	0.921	-0.1116	0.2241	DNAJA3
P25685	-0.0499	0.6502	-0.1124	0.2488	DNAJB1
P25685;Q9UDY4	-0.1235	0.4097	-0.1521	0.2775	DNAJB1;DNAJB4
Q9UBS4	0.0656	0.6062	-0.0488	0.4759	DNAJB11
Q8IXB1	-0.0593	0.6804	0.0997	0.23	DNAJC10
Q9NVH1	-0.1246	0.2639	-0.0709	0.2586	DNAJC11
O75165	-0.0207	0.7502	-0.0272	0.5456	DNAJC13
Q13217	0.0467	0.5556	0.1926	0.0851	DNAJC3
Q99615	0.0997	0.5278	0.013	0.9403	DNAJC7
O00115	0.2223	0.1185	-0.0562	0.6585	DNASE2
Q05193	-0.2936	0.2222	-0.1573	0.3275	DNM1
P50570;Q05193;Q9UQ16	-0.121	0.3542	-0.0669	0.483	DNM1;DNM2
O00429	0.1425	0.1342	0.0582	0.558	DNM1L
P50570	0.1757	0.1291	0.0108	0.8605	DNM2
Q9ULA0	-0.0355	0.5639	-0.0065	0.8936	DNPEP
Q96N67	-0.0245	0.7765	-0.1359	0.2162	DOCK7
O60762	0.0029	0.979	-0.0824	0.2582	DPM1
Q9NY33	0.0161	0.8758	0.0789	0.5532	DPP3
P27487	-0.2	0.4022	-0.3481	0.252	DPP4
Q9UHL4	0.1096	0.5351	-0.2762	0.2539	DPP7
Q86TI2	0.2426	0.1833	-0.0069	0.9674	DPP9
Q12882	0.0051	0.9741	0.0707	0.4798	DPYD
Q16555	-0.0974	0.3266	-0.1361	0.14	DPYSL2
Q14194;Q14195;Q16555	-0.0688	0.3602	-0.0716	0.4252	DPYSL2;DPYSL3
Q14195	-0.005	0.9769	0.0419	0.7383	DPYSL3
Q01658	-0.0835	0.5253	0.1018	0.4228	DR1
Q9Y295	0.1174	0.0434	-0.0776	0.3092	DRG1
P55039	-0.1087	0.2703	-0.0186	0.8628	DRG2
Q03001	-0.2163	0.4233	0.1447	0.3982	DST
P60981	0.2067	0.1216	0.1686	0.2894	DSTN
Q8TEA8	0.0919	0.4696	0.097	0.3506	DTD1
Q8TDB6	0.1086	0.4908	-0.2533	0.2389	DTX3L
P23919	-0.128	0.1078	0.0289	0.8836	DTYMK
Q8WTR2	0.0683	0.8752	-0.116	0.3954	DUSP19
P51452	0.191	0.2487	0.1332	0.246	DUSP3
P33316	0.0138	0.8951	-0.0475	0.7439	DUT

O14641	-0.1187	0.5022	0.0398	0.769	DVL2
Q14204	-0.0073	0.9261	0.0349	0.4133	DYNC1H1
Q13409	-0.0572	0.6041	0.0885	0.2661	DYNC1I2
Q9Y6G9	0.0971	0.3656	0.0644	0.4072	DYNC1LI1
O43237	0.1442	0.1733	-0.0624	0.4648	DYNC1LI2
P63167;Q96FJ2	0.226	0.2553	-0.052	0.7499	DYNLL1
Q9NP97	0.0387	0.7929	0.1299	0.1275	DYNLRB1
P63172	-0.1015	0.7045	0.0129	0.9459	DYNLT1
P51808	-0.0148	0.9367	0.0246	0.8461	DYNLT3
Q15125	-0.3237	0.1137	-0.0611	0.638	EBP
P42892	0.0303	0.8649	-0.1076	0.5262	ECE1
Q13011	-0.1296	0.1211	0.0156	0.8847	ECH1
Q9NTX5	0.0758	0.5014	-0.1068	0.4254	ECHDC1
P30084	-0.0653	0.3937	0.0019	0.9892	ECHS1
P42126	0.1761	0.2899	0.1363	0.1824	ECI1
O75521	-0.0508	0.5509	-0.0267	0.837	ECI2
Q5VYK3	0.1366	0.278	0.1502	0.3152	ECPAS
O60869	0.0471	0.6833	-0.1657	0.2251	EDF1
Q15075	-0.0486	0.6744	-0.0783	0.2907	EEA1
P68104;Q5VTE0	0.1274	0.1688	0.0749	0.4094	EEF1A1;EEF1A1P5
P68104;Q05639;Q5VTE0	0.0873	0.1765	0.027	0.7632	EEF1A2
P24534	-0.0751	0.1104	-0.1442	0.1098	EEF1B2
P24534;P29692	0.001	0.9904	0.0149	0.8601	EEF1B2;EEF1D
P29692	-0.0378	0.2955	0.0186	0.6574	EEF1D
O43324	0.0677	0.4601	0.0938	0.3523	EEF1E1
P26641	0.037	0.5002	-0.0007	0.9913	EEF1G
P13639	0.0498	0.4391	0.0189	0.8297	EEF2
Q9BUP0;Q96C19	-0.0902	0.6755	0.2451	0.2013	EFHD1;EFHD2
Q96C19	0.0005	0.9954	-0.0205	0.8408	EFHD2
Q15029	0.0461	0.7104	-0.0672	0.397	EFTUD2
P00533	-0.0602	0.6389	0.0534	0.7777	EGFR
Q8NDI1	0.2637	0.0412	-0.013	0.9184	EHBP1
Q9H4M9	0.0162	0.8515	-0.1139	0.2739	EHD1
Q9H4M9;Q9NZN3;Q9NZN4	0.1494	0.3816	-0.0743	0.5339	EHD1;EHD2;EHD3
Q9H4M9;Q9NZN3	0.0608	0.6584	0.0145	0.8555	EHD1;EHD3
Q9H223;Q9H4M9;Q9NZN3	0.1659	0.2389	-0.0834	0.4753	EHD1;EHD3;EHD4
Q9NZN4	0.1294	0.2647	0.0412	0.7775	EHD2
Q9H223;Q9NZN3	0.1054	0.5598	0.0608	0.7395	EHD3;EHD4
Q9H223	0.1707	0.2616	0.1596	0.2237	EHD4
O14681	0.0439	0.8197	-0.1687	0.1944	EI24
P41567;O60739	0.0573	0.3575	0.0592	0.7462	EIF1;EIF1B
P47813;O14602	0.0925	0.077	-0.0214	0.8648	EIF1AX
Q9BY44	0.2161	0.2001	0.1816	0.0621	EIF2A
P19525	0.0366	0.7159	-0.0299	0.7002	EIF2AK2
Q14232	0.0164	0.8565	-0.2487	0.0216	EIF2B1
Q9NR50	-0.1199	0.2067	0.024	0.7893	EIF2B3
Q9UI10	-0.1033	0.2889	0.0034	0.9617	EIF2B4
Q13144	-0.0646	0.5308	-0.019	0.8484	EIF2B5

P05198	0.0083	0.8405	0.0113	0.8585	EIF2S1
P20042	0.0673	0.5174	-0.0288	0.7828	EIF2S2
P41091;Q2VIR3	0.0329	0.6774	0.0177	0.8821	EIF2S3
Q14152	0.0857	0.3057	0.0123	0.8465	EIF3A
P55884	0.0812	0.2868	0.0419	0.4541	EIF3B
Q99613;B5ME19	-0.0195	0.8217	0.0383	0.5147	EIF3C;EIF3CL
O15371	0.018	0.841	0.0904	0.0795	EIF3D
P60228	-0.0116	0.8962	0.0576	0.4757	EIF3E
O00303	0.0633	0.3193	-0.0336	0.5427	EIF3F
O75821	0.0223	0.8391	-0.0266	0.427	EIF3G
O15372	-0.1562	0.2885	0.1142	0.6786	EIF3H
Q13347	0.1216	0.0452	-0.0042	0.9383	EIF3I
O75822	-0.0478	0.5832	0.2063	0.1166	EIF3J
Q9UBQ5	-0.0268	0.8731	-0.0123	0.9267	EIF3K
Q9Y262	-0.0494	0.5698	-0.1034	0.1693	EIF3L
Q7L2H7	0.1513	0.096	0.0049	0.9469	EIF3M
P60842	0.1084	0.3163	0.0092	0.9403	EIF4A1
P60842;Q14240	-0.0196	0.7017	-0.0087	0.9094	EIF4A1;EIF4A2
P38919;P60842;Q14240	0.0794	0.2228	0.0517	0.4191	EIF4A1;EIF4A2;EIF4A3
Q14240	0.2982	0.1993	-0.0137	0.9087	EIF4A2
P38919	0.0488	0.6896	-0.0673	0.2358	EIF4A3
P23588	-0.2858	0.0576	0.0096	0.9036	EIF4B
P06730	0.036	0.5361	-0.0181	0.7661	EIF4E
Q13541	0.1057	0.6602	-0.1505	0.5411	EIF4EBP1
Q04637	0.0206	0.7162	-0.0328	0.448	EIF4G1
O43432;Q04637	0.0069	0.949	0.0151	0.9016	EIF4G1;EIF4G3
P78344	0.1394	0.0986	-0.0225	0.7599	EIF4G2
Q15056	0.087	0.4237	0.0774	0.3663	EIF4H
P55010	0.1548	0.1184	0.0235	0.8402	EIF5
P63241;Q9GZV4;Q6IS14	-0.1081	0.3341	-0.0198	0.844	EIF5A
O60841	0.1494	0.1983	-0.0647	0.3524	EIF5B
P56537	0.0274	0.8029	0.0106	0.8846	EIF6
Q53HC9	-0.272	0.0547	-0.0133	0.9363	EIPR1
Q15717	0.0671	0.3046	-0.0188	0.7681	ELAVL1
Q96JJ3	0.1589	0.2497	0.0731	0.6272	ELMO2
Q8IZ81	-0.3046	0.3429	-0.0126	0.889	ELMOD2
Q15370	-0.0445	0.363	-0.0253	0.6487	ELOB
Q15369	0.0901	0.1832	0.0165	0.8318	ELOC
Q8N766	-0.0045	0.949	0.0228	0.7454	EMC1
Q9P0I2	0.1096	0.4556	-0.175	0.3596	EMC3
Q5J8M3	-0.1185	0.1639	-0.2234	0.0332	EMC4
Q9NPA0	-0.0629	0.6979	-0.0171	0.8706	EMC7
P50402	0.1169	0.3135	-0.0467	0.5686	EMD
O00423	-0.3583	0.1669	-0.0764	0.5996	EML1
O95834	0.0049	0.9703	0.0356	0.7541	EML2
Q32P44	-0.141	0.2795	0.1403	0.3117	EML3
Q9HC35	0.1423	0.2503	0.0398	0.8156	EML4
Q8N8S7	0.539	0.1767	0.4917	0.4603	ENAH

O94919	0.132	0.5494	-0.0532	0.7692	ENDOD1
Q14249	0.2559	0.2005	0.0914	0.4997	ENDOG
P17813	0.0969	0.5304	-0.3263	0.2058	ENG
P06733	0.0596	0.3032	0.0281	0.7919	ENO1
P06733;P09104;P13929	0.0121	0.9187	0.0402	0.718	ENO1;ENO2;ENO3
P09104	0.0672	0.6065	0.1021	0.326	ENO2
Q9UHY7	0.0649	0.4951	0.0604	0.5757	ENOPH1
Q6UWV6;Q9Y6X5	-0.1867	0.3973	-0.0871	0.7589	ENPP4;ENPP7
O43768;P56211	0.2149	0.3174	-0.0672	0.3349	ENSA
Q9H4G0	0.0431	0.8719	0.0946	0.6697	EPB41L1
O43491	-0.0104	0.9245	-0.017	0.8468	EPB41L2
Q9Y2J2	0.062	0.6914	-0.0792	0.6593	EPB41L3
P07099	-0.0671	0.7154	-0.2021	0.5096	EPHX1
Q9Y6I3	0.0162	0.8548	0.0216	0.7019	EPN1
P07814	0.0164	0.8212	0.0975	0.3811	EPRS1
P42566	0.0795	0.6044	0.0514	0.5981	EPS15
Q9UBC2	-0.0255	0.8633	-0.1495	0.2131	EPS15L1
Q12929	-0.0315	0.8107	0.0587	0.6167	EPS8
Q9NZ08	-0.0864	0.5102	-0.1475	0.2048	ERAP1
Q96RT1	-0.0238	0.8773	-0.096	0.3892	ERBIN
Q969X5	-0.0983	0.3832	0.044	0.7028	ERGIC1
P84090	0.1866	0.4262	-0.0744	0.6289	ERH
O43414	-0.0367	0.7484	-0.0229	0.751	ERI3
O75477	-0.2031	0.0275	-0.1978	0.0283	ERLIN1
O75477;O94905	-0.0677	0.4275	-0.0713	0.4519	ERLIN1;ERLIN2
O94905	-0.0075	0.9283	-0.0552	0.5492	ERLIN2
Q96HE7	-0.07	0.3707	0.1287	0.0799	ERO1A
Q86YB8;Q96HE7	0.0022	0.9941	0.0081	0.9427	ERO1A;ERO1B
P30040	-0.134	0.3022	-0.1185	0.2249	ERP29
Q9BS26	-0.0681	0.3457	-0.0021	0.9692	ERP44
P10768	-0.0206	0.7992	-0.0997	0.211	ESD
Q9BSJ8	-0.0731	0.478	-0.1062	0.0462	ESYT1
A0FGR8	-0.0225	0.7452	-0.0903	0.1765	ESYT2
P62495	-0.0201	0.736	0.1056	0.1493	ETF1
P13804	-0.0963	0.3695	-0.032	0.8508	ETFA
P38117	-0.0639	0.4613	0.0509	0.7578	ETFB
Q16134	-0.1263	0.1799	0.039	0.7956	ETFDH
O95571	0.0483	0.8136	-0.1637	0.5359	ETHE1
Q01844	0.0244	0.8613	-0.0381	0.7678	EWSR1
Q9NV70	0.1864	0.1379	-0.0409	0.7619	EXOC1
Q96KP1	-0.136	0.351	-0.0093	0.9561	EXOC2
O60645	-0.1632	0.1936	0.0169	0.9053	EXOC3
Q96A65	0.0333	0.6302	-0.0136	0.8981	EXOC4
Q9Y2D4	0.098	0.2903	0.0639	0.6017	EXOC6B
Q9UPT5	0.0483	0.6942	0.1595	0.1587	EXOC7
Q8IYI6	0.2721	0.1193	-0.0642	0.7362	EXOC8
Q13868	-0.194	0.1377	0.1012	0.6655	EXOSC2
Q5RKV6	-0.0521	0.7508	-0.214	0.1383	EXOSC6

Q06265	-0.2101	0.0718	-0.0192	0.9228	EXOSC9
P15311	0.0238	0.89	0.0492	0.765	EZR
P15311;P26038;P35241	0	0.9996	0.0601	0.5418	EZR;MSN;RDX
P05413	-0.4389	0.3253	0.0916	0.8269	FABP3
Q01469	0.2667	0.2332	0.1553	0.4065	FABP5
Q96CS3	0.245	0.0736	-0.2233	0.1528	FAF2
P16930	0.0281	0.8424	-0.0619	0.6527	FAH
Q6P587	-0.0873	0.4097	-0.1654	0.2566	FAHD1
Q96GK7;Q6P2I3	0.1792	0.2004	0.1852	0.0892	FAHD2A
Q8IWE2	-0.1068	0.2058	0.0874	0.1922	FAM114A1
Q9NZB2	0.0007	0.9915	-0.0749	0.2792	FAM120A
Q92520	-0.1018	0.5169	-0.0813	0.5318	FAM3C
Q14320;Q9Y247	0.1451	0.2631	-0.0273	0.7902	FAM50A
Q9UBU6	-0.3378	0.1475	-0.0274	0.8731	FAM8A1
Q8NCA5	-0.0989	0.5868	-0.1053	0.5315	FAM98A
Q52LJ0	-0.0714	0.4876	-0.0214	0.806	FAM98B
Q9Y4F1	0.0026	0.991	-0.0775	0.5649	FARP1
Q9Y285	0.1149	0.4922	0.2799	0.022	FARSA
Q9NSD9	0.1162	0.149	0.0823	0.3547	FARSB
P49327	-0.1845	0.3933	0.0246	0.8251	FASN
P62861	-0.0277	0.7379	0.1059	0.2501	FAU
P22087	0.0441	0.6975	0.0084	0.9429	FBL
A6NHQ2	-0.0373	0.581	0.042	0.7378	FBLL1
P23142	0.2924	0.0912	-0.1737	0.3314	FBLN1
P14324	-0.1962	0.3125	0.0155	0.8532	FDPS
P22570	-0.0719	0.6942	-0.0377	0.8542	FDXR
P22830	0.046	0.7857	0.0106	0.9383	FECH
Q96AC1;Q9BQL6	0.0775	0.5538	0.0985	0.5516	FERMT2
P09038	0.2099	0.4869	0.3142	0.3212	FGF2
P07954	0.0773	0.4207	0.0422	0.6882	FH
Q5W0V3	0.1392	0.4941	0.0261	0.825	FHIP2A
Q13642	-0.4327	0.3401	-0.0931	0.7388	FHL1
Q14192	0.1024	0.6984	0.0107	0.9696	FHL2
Q13643	0.3609	0.1242	0.284	0.3918	FHL3
O75351;Q6PIW4;Q9UN37	0.0264	0.7526	-0.0439	0.4669	FIGNL1;VPS4A;VPS4B
Q9Y3D6	0.1389	0.2011	-0.0377	0.7221	FIS1
Q96AY3	0.0391	0.8258	-0.129	0.271	FKBP10
Q5T1M5	0.2428	0.0223	-0.1176	0.3108	FKBP15
P62942	0.1906	0.1691	0.0977	0.3753	FKBP1A
P26885	-0.0746	0.3881	0.0494	0.6333	FKBP2
Q00688	-0.0243	0.7251	-0.0065	0.9135	FKBP3
Q02790	0.0254	0.5986	-0.1139	0.1122	FKBP4
Q9Y680	0.0495	0.6239	0.0818	0.6601	FKBP7
Q14318	-0.014	0.8479	0.0283	0.6554	FKBP8
O95302	-0.0015	0.993	-0.0123	0.9145	FKBP9
Q13045	0.0454	0.62	0.2556	0.0042	FLII
P21333	0.113	0.4888	0.1889	0.4715	FLNA
O75369;P21333	0.1443	0.522	0.23	0.336	FLNA;FLNB

O75369;P21333;Q14315	0.1472	0.388	0.1842	0.4119	FLNA;FLNB;FLNC
P21333;Q14315	0.0409	0.8283	0.1563	0.4934	FLNA;FLNC
O75369	-0.1119	0.5013	-0.0815	0.5877	FLNB
O75369;Q14315	0.0728	0.7319	0.1116	0.5268	FLNB;FLNC
Q14315	0.0942	0.7374	0.2174	0.2518	FLNC
O75955	-0.0469	0.5876	-0.0812	0.4668	FLOT1
Q14254	0.0184	0.8247	-0.0177	0.8335	FLOT2
P02751	-0.1329	0.6645	-0.1028	0.7885	FN1
Q9Y2H6	-0.2415	0.2953	-0.54	0.0347	FNDC3A
P49354	-0.3413	0.0064	-0.0738	0.3667	FNTA
Q96NE9	-0.2509	0.3641	0.1428	0.5092	FRMD6
Q9BZ67;Q9BZ68	0.0494	0.8303	-0.211	0.1611	FRMD8
Q16658	-0.1358	0.3844	-0.0186	0.7627	FSCN1
Q12841	0.2158	0.1167	0.0106	0.9586	FSTL1
P02794	-0.2623	0.1105	-0.6509	0.0322	FTH1
P02792	-0.2327	0.129	-0.589	0.1028	FTL
Q9C0B1	0.0383	0.7541	-0.1107	0.533	FTO
Q8IY81	0.0608	0.7413	-0.2087	0.4175	FTSJ3
Q96AE4	0.2268	0.0246	-0.0212	0.6798	FUBP1
Q92945;Q96AE4	-0.063	0.5748	0.0014	0.9846	FUBP1;KHSRP
Q96I24	0.1636	0.1092	-0.0311	0.4823	FUBP3
P35637	0.0578	0.678	-0.0849	0.4769	FUS
P35637;Q92804	-0.0439	0.7045	-0.0506	0.4683	FUS;TAF15
P51114	0.0443	0.6965	0.0151	0.848	FXR1
Q9BQS8	-0.1478	0.4603	0.0309	0.8961	FYCO1
Q13283	-0.0247	0.624	0.0904	0.093	G3BP1
Q9UN86	0.1883	0.1685	0.0872	0.3006	G3BP2
P11413	0.0465	0.7565	0.0958	0.4901	G6PD
P10253	-0.1017	0.625	0.051	0.7991	GAA
P51570	-0.0988	0.5583	-0.2328	0.0563	GALK1
P34059	-0.3783	0.0125	-0.2364	0.2723	GALNS
Q10471	-0.0342	0.7638	0.1554	0.1634	GALNT2
Q14353	-0.056	0.626	0.2599	0.1167	GAMT
Q9H2C0	-0.0796	0.6668	0.0905	0.5142	GAN
Q14697	-0.1029	0.1562	-0.0657	0.2309	GANAB
P04406	0.0564	0.5172	0.0399	0.6187	GAPDH
P41250	0.0241	0.8911	0.1813	0.4891	GARS1
P22102	0.0658	0.3958	0.1414	0.2262	GART
Q8NB37	0.0821	0.617	-0.0387	0.7975	GATD1
A0A0B4J2D5;P0DPI2	0.0777	0.4153	-0.0736	0.6495	GATD3;GATD3B
P04062	-1.3515	0	-1.2612	0	GBA1
Q04446	-0.051	0.6029	-0.0353	0.6867	GBE1
Q92538	-0.0285	0.8194	0.1638	0.2278	GBF1
P32455;P32456	0.2259	0.3338	-0.0043	0.9806	GBP1
P30047	-0.0009	0.9931	0.0348	0.7511	GCHFR
P48507	0.2305	0.162	0.1947	0.1744	GCLM
Q92616	0.0361	0.5763	-0.0117	0.8318	GCN1
Q9NXN4	0.0579	0.6888	-0.0204	0.8987	GDAP2

P31150	-0.078	0.2926	0.0897	0.1357	GDI1
P31150;P50395	-0.0393	0.1985	0.0207	0.5628	GDI1;GDI2
P50395	-0.0468	0.2978	-0.0766	0.0232	GDI2
P57678	0.1054	0.7442	-0.3421	0.239	GEMIN4
Q8TEQ6	0.2877	0.448	0.0746	0.9014	GEMIN5
O43681	0.0712	0.3657	0.0889	0.3194	GET3
Q96RP9	0.0099	0.9409	-0.0809	0.6	GFM1
Q06210	0.048	0.8208	0.0255	0.92	GFPT1
Q13630	0.0015	0.9867	-0.0183	0.8206	GFUS
O75223	0.0454	0.85	0.0478	0.693	GGCT
Q92820	0.1455	0.378	-0.006	0.9655	GGH
Q9H3K2	-0.0262	0.7478	0.1141	0.3511	GHITM
Q14161	0.0753	0.3262	0.061	0.6164	GIT2
P17302	-0.2734	0.5081	-0.0169	0.9565	GJA1
P16278	0.0943	0.5057	0.0636	0.6618	GLB1
Q92896	-0.229	0.0157	0.0601	0.5959	GLG1
Q9H4G4	-0.3924	0.0543	0.005	0.9669	GLIPR2
Q04760	-0.0284	0.5647	0.0557	0.5665	GLO1
Q9HC38	-0.0763	0.1954	-0.0098	0.864	GLOD4
P35754	0.5608	0.0681	0.0492	0.8495	GLRX
O76003	0.0407	0.5325	0.0036	0.9465	GLRX3
O94925	0.0571	0.8361	0.3783	0.4295	GLS
Q68CQ7	0.0565	0.8201	0.269	0.0486	GLT8D1
P00367;P49448	-0.1358	0.3346	-0.0282	0.9003	GLUD1
P60983	0.0524	0.5978	-0.0246	0.7528	GMFB
Q96IJ6	-0.0186	0.8534	0.2253	0.0782	GMPPA
Q9Y5P6	0.042	0.574	-0.012	0.873	GMPPB
Q9P2T1	0.1307	0.1526	0.1082	0.4661	GMPR2
P49915	0.0247	0.7869	0.0604	0.3204	GMPS
P29992	-0.07	0.4393	-0.0418	0.5585	GNA11
Q14344	-0.0854	0.4772	-0.0596	0.4219	GNA13
P04899;P08754;P09471;P63096	0.0551	0.6509	-0.1454	0.2296	GNAI1;GNAI2;GNAI3
P04899	0.001	0.989	-0.046	0.6109	GNAI2
P08754	-0.1158	0.4859	-0.104	0.2723	GNAI3
P50148	0.0689	0.4822	0.049	0.6101	GNAQ
P63092;Q5JWF2	0.0019	0.9784	-0.1153	0.1602	GNAS
P62873	0.0471	0.4142	-0.0236	0.6852	GNB1
P62873;P62879	0.0583	0.6823	-0.1203	0.3036	GNB1;GNB2
P16520;P62873;P62879;Q9HAV0	-0.0764	0.2028	-0.0035	0.9338	GNB1;GNB2;GNB4
P62879	0.0148	0.8654	0.0038	0.9601	GNB2
Q9HAV0	-0.0015	0.9903	0.0286	0.8421	GNB4
Q9UBI6	-0.0574	0.451	0.0087	0.8677	GNG12
P59768	0.0729	0.8422	-0.2673	0.2969	GNG2
P36915	0.0231	0.8733	-0.0176	0.7428	GNL1
P46926	-0.0262	0.7924	-0.0803	0.4024	GNPDA1
P46926;Q8TDQ7	-0.0328	0.7237	-0.126	0.217	GNPDA1;GNPDA2
Q8TDQ7	-0.0365	0.7353	-0.0613	0.5639	GNPDA2
Q96EK6	0.0416	0.8534	0.0812	0.4691	GNPNAT1

P15586	0.1126	0.1142	-0.1811	0.2784	GNS
Q08379	0.069	0.6789	0.1209	0.245	GOLGA2
Q08378	-0.0251	0.8791	0.0385	0.7774	GOLGA3
O00461	-0.1322	0.4076	0.1469	0.3153	GOLIM4
Q6P4E1	-0.1046	0.596	0.0237	0.8891	GOLM2
Q9HD26	0.0936	0.5475	0.2811	0.0861	GOPC
Q9H8Y8	-0.1232	0.1146	0.0624	0.4451	GORASP2
P17174	0.0361	0.7971	0.1061	0.5438	GOT1
P00505	0.0547	0.1905	0.0459	0.6538	GOT2
P43304	-0.1562	0.2906	-0.0586	0.7376	GPD2
P06744	-0.026	0.4686	-0.1075	0.1335	GPI
Q13098	-0.1349	0.1565	-0.0033	0.9654	GPS1
P07203	0.0357	0.8076	0.1588	0.1172	GPX1
P36969	-0.0212	0.9014	0.0186	0.8533	GPX4
Q8TED1	0.0461	0.6742	0.0785	0.3544	GPX8
P62993	-0.1294	0.4271	0.0986	0.3534	GRB2
Q9UBQ7	-0.0752	0.4324	-0.0687	0.4779	GRHPR
Q12849	0.0084	0.9586	-0.3208	0.2392	GRSF1
O60443	0.2174	0.2419	0.047	0.7131	GSDME
P49840	0.0933	0.6179	-0.1713	0.3288	GSK3A
P49841	-0.0616	0.6568	0.0312	0.8099	GSK3B
P06396	-0.0059	0.972	0.0659	0.7712	GSN
P15170;Q8IYD1	-0.1032	0.2944	-0.0186	0.7533	GSPT1
P00390	0.0864	0.3125	-0.0847	0.1441	GSR
P48637	-0.0932	0.3331	-0.0571	0.435	GSS
Q9Y2Q3	-0.0876	0.4464	-0.2104	0.2241	GSTK1
P09488;P28161;Q03013	-0.1497	0.4669	0.137	0.467	GSTM1;GSTM2
P28161	0.0206	0.8972	0.1726	0.3877	GSTM2
P21266;P28161;P46439	-0.2657	0.2153	-0.4024	0.1817	GSTM2;GSTM3
P21266	-0.3285	0.2624	-0.5961	0.1123	GSTM3
P78417	0.1928	0.1541	0.0246	0.7501	GSTO1
P09211	-0.0655	0.4089	-0.021	0.6672	GSTP1
O00178	0.6199	0.059	0.07	0.6918	GTPBP1
Q16774	0.1364	0.3261	-0.0157	0.904	GUK1
P08236	-0.7919	0.0025	-0.064	0.7275	GUSB
P46976	0.1365	0.3306	0.2134	0.0733	GYG1
P13807	0.1954	0.4528	0.307	0.1469	GYS1
P07305	-0.1948	0.398	-0.0243	0.9242	H1-0
Q92522	-0.1276	0.6214	-0.0497	0.7085	H1-10
P10412;P16402;P16403	-0.0809	0.7151	0.0534	0.7985	H1-2;H1-3;H1-4
P16401	-0.0194	0.9274	-0.1188	0.3971	H1-5
P04908;P0C0S8;P20671;Q16777;Q6FI13;Q7L7L0;Q93077;Q96KK5;Q99878;Q9BTM1	0.0515	0.7715	-0.0498	0.7377	H2AC11;H2AC12;H2AC14;H2AC18;H2AC20;H2AC25;H2AC4;H2AC6;H2AC7;
P0C0S5;Q71UI9	0.1323	0.468	-0.1762	0.3808	H2AZ1;H2AZ2

O60814;P06899;P23527;P33778;P57053;P58876;P62807;Q16778;Q5QNW6;Q8N257;Q93079;Q96A08;Q99877;Q99879;Q99880	0.0034	0.9858	-0.0667	0.5657	H2BC12;H2BC12L;H2BC13;H2BC14;H2BC15;H2BC18;H2BC4;H2BC5;H2BC9
P06899;P23527;P33778;Q16778;Q6DN03;Q6DRA6;Q8N257	-0.016	0.9353	0.0452	0.7871	H2BC19P;H2BC20P
P68431;P84243;Q16695;Q6NXT2;Q71DI3;Q5TEC6	-0.161	0.3429	-0.1231	0.3348	H3-3A;H3-5;H3-7;H3C1;H3C15
P62805	0.0409	0.8171	-0.1408	0.2518	H4C1
O95479	-0.1845	0.3258	-0.184	0.1956	H6PD
Q9P035	-0.1189	0.1932	-0.0236	0.8109	HACD3
Q16836	-0.2055	0.1192	-0.3434	0.1637	HADH
P40939	-0.0938	0.1958	-0.052	0.6906	HADHA
P55084	-0.0513	0.314	-0.0402	0.7439	HADHB
P12081;P49590	-0.0437	0.2533	0.0617	0.111	HARS1
P69905	0.057	0.7876	-0.1772	0.4288	HBA1
P02042;P02100;P68871;P69891;P69892	-0.1343	0.4659	-0.2923	0.1728	HBB;HBD;HBE1;HBG1;HBG2
Q9Y450	-0.1945	0.0569	0.2448	0.0284	HBS1L
P51610	0.0758	0.3396	0.0102	0.9291	HCFC1
Q13547;Q92769	-0.0398	0.7526	-0.1352	0.1842	HDAC1;HDAC2
P51858	-0.0673	0.5065	0.0137	0.8739	HDGF
Q7Z4V5	0.058	0.7248	-0.1499	0.3247	HDGFL2
Q9Y3E1	-0.0894	0.5776	0.2376	0.2137	HDGFL3
Q00341	0.0805	0.1531	0.0027	0.976	HDLBP
Q9P2D3	0.1754	0.3167	0.1496	0.5086	HEATR5B
Q9NRV9	-0.1238	0.4154	-0.2215	0.0604	HEBP1
Q9Y5Z4	0.1978	0.1218	0.2655	0.2011	HEBP2
Q5GLZ8	-0.1019	0.5264	-0.0541	0.533	HERC4
P06865	0.1071	0.3598	-0.025	0.8688	HEXA
P07686	0.0959	0.4126	-0.0357	0.8433	HEXB
Q9BTY7	0.0827	0.4622	-0.0329	0.7813	HGH1
O14964	-0.1181	0.2497	-0.0959	0.2513	HGS
Q68CP4	-0.249	0.1835	-0.0546	0.7229	HGSNAT
P31937	-0.1418	0.3412	0.0299	0.8834	HIBADH
Q6NVY1	-0.2168	0.2491	-0.0757	0.3152	HIBCH
P49773	0.3017	0.026	-0.002	0.9891	HINT1
Q9BX68	0.0565	0.6841	0.0812	0.6363	HINT2
P54198	0.0795	0.5419	-0.0017	0.9762	HIRA
P19367	-0.0777	0.523	-0.0238	0.8332	HK1
P19367;P52789;P52790	0.1098	0.5039	-0.02	0.8428	HK1;HK2
P04439	0.212	0.3598	-0.3325	0.1682	HLA-A
P10321	-0.2694	0.3117	-0.4559	0.2782	HLA-C
P17693	-0.2964	0.1441	-0.4956	0.1742	HLA-G
Q16534	-0.1099	0.5249	-0.0891	0.6418	HLF
Q8TCT9	-0.0116	0.8807	-0.0137	0.8399	HM13
P17096	0.0386	0.8432	-0.0962	0.6923	HMGA1
P09429	0.078	0.6695	-0.0394	0.7284	HMGB1

P26583	0.0074	0.9648	-0.0495	0.7207	HMGB2
O15347	-0.0761	0.5953	0.0592	0.7278	HMGB3
P35914	-0.1202	0.163	-0.1172	0.2317	HMGCL
P09601	0.0844	0.7087	-0.3886	0.1206	HMOX1
Q13151	0.0258	0.8212	-0.112	0.2132	HNRNPA0
P09651;Q32P51	0.0689	0.3469	-0.0466	0.3371	HNRNPA1
P22626	0.0365	0.6693	-0.0971	0.1119	HNRNPA2B1
P51991	0.0321	0.7234	-0.0617	0.3217	HNRNPA3
Q99729	-0.0107	0.9354	0.0644	0.4561	HNRNPAB
P07910	-0.019	0.8379	-0.068	0.2238	HNRNPC
Q14103	0.0427	0.6036	-0.0752	0.2489	HNRNPD
O14979	0.0619	0.3462	-0.0241	0.6396	HNRNPDL
P52597	0.0204	0.8696	-0.1408	0.1372	HNRNPF
P31943;P52597	0.0122	0.8784	-0.0401	0.6336	HNRNPF;HNRNPH1
P31943;P52597;P55795	-0.0583	0.5786	-0.1335	0.0757	HNRNPF;HNRNPH1;HNR
P31943	-0.0135	0.8775	-0.0371	0.6443	HNRNPH1
P31943;P55795	0.2157	0.153	0.0467	0.7052	HNRNPH1;HNRNPH2
P55795	-0.1199	0.092	-0.0573	0.5466	HNRNPH2
P31942	0.046	0.6705	0.029	0.6878	HNRNPH3
P61978	-0.0111	0.8905	-0.0754	0.1288	HNRNPK
P14866	0.0112	0.856	-0.0079	0.8608	HNRNPL
P52272	0.0727	0.2595	-0.0176	0.7544	HNRNPM
O43390	0.0572	0.5199	-0.0071	0.8899	HNRNPR
O43390;O60506	-0.0972	0.3649	-0.0251	0.781	HNRNPR;SYNCRIP
Q00839	0.0135	0.8612	-0.0491	0.2709	HNRNPU
Q9BUJ2	0.0202	0.8488	-0.0974	0.2306	HNRNPUL1
Q1KMD3	-0.0368	0.7383	-0.0112	0.8917	HNRNPUL2
Q86VS8	0.1664	0.1504	0.0497	0.5766	HOOK3
Q5SSJ5	0.0801	0.5176	-0.0168	0.8522	HP1BP3
P37235;P61601;P84074	0.0306	0.8749	-0.0087	0.9496	HPCA;HPCAL1;NCALD
P00492	0.1074	0.2124	0.0345	0.6382	HPRT1
P01112	-0.1971	0.0485	-0.0662	0.4705	HRAS
Q53T59	-0.0612	0.5683	-0.3101	0.0196	HS1BP3
Q99714	-0.0083	0.9369	0.0851	0.5056	HSD17B10
Q8NBQ5	0.1582	0.3959	-0.1466	0.101	HSD17B11
Q53GQ0	-0.0203	0.7436	0.1019	0.0691	HSD17B12
P51659	-0.0505	0.5326	-0.1437	0.2364	HSD17B4
Q6YN16	-0.0665	0.5984	-0.1624	0.3712	HSDL2
P07900	-0.0342	0.4958	-0.0274	0.5686	HSP90AA1
P07900;P08238	-0.0337	0.6418	-0.0531	0.3982	HSP90AA1;HSP90AB1
P07900;P08238;Q14568;Q58FF8	0.02	0.6837	-0.0615	0.2004	HSP90AA1;HSP90AB1;HS
P08238	0.0053	0.9072	-0.0025	0.9293	HSP90AB1
P08238;Q58FF8	0.0428	0.4504	0.0368	0.3299	HSP90AB1;HSP90AB2P
P08238;P14625	0.0452	0.5478	-0.0203	0.715	HSP90AB1;HSP90B1
Q58FF8	-0.1951	0.2138	0.0871	0.3019	HSP90AB2P
P14625	-0.0737	0.2982	-0.0671	0.2343	HSP90B1
O43301	0.0229	0.8625	0.0522	0.7867	HSPA12A
P0DMV8;P0DMV9	-0.0168	0.8469	-0.0983	0.405	HSPA1A;HSPA1B

P0DMV8;P0DMV9;P17066	-0.0766	0.4326	0.0034	0.98	HSPA1A;HSPA1B;HSPA6
P0DMV8;P0DMV9;P34931	-0.0642	0.4211	-0.0043	0.9671	HSPA1L
P0DMV8;P0DMV9;P11021;P11142;P34931;P54652	0.0593	0.1677	0.0263	0.7324	HSPA1L;HSPA2;HSPA5;HSPA8
P0DMV8;P0DMV9;P11142;P17066;P34931;P54652;P48741	-0.0113	0.8248	0.009	0.8084	HSPA1L;HSPA2;HSPA8
P11142;P54652	-0.002	0.9631	-0.0393	0.4586	HSPA2;HSPA8
P34932	0.0108	0.6943	0.0427	0.2336	HSPA4
P34932;O95757	0.0754	0.4903	0.0326	0.8019	HSPA4;HSPA4L
O95757	-0.0409	0.7474	-0.0259	0.8567	HSPA4L
P11021	-0.0026	0.9579	-0.0272	0.719	HSPA5
P11142	0.0259	0.4302	0.007	0.8583	HSPA8
P38646	0.0163	0.8253	0.0545	0.3603	HSPA9
P04792	0.0818	0.2526	0.1733	0.2613	HSPB1
Q9NZL4	0.0084	0.9254	-0.1094	0.4953	HSPBP1
P10809	0.0028	0.9742	-0.0048	0.9748	HSPD1
P61604	-0.0828	0.4808	-0.0592	0.7396	HSPE1
Q92598	-0.0468	0.5989	0.1483	0.1099	HSPH1
O43464	0.0864	0.3329	0.0839	0.2097	HTRA2
Q7Z6Z7	0.0309	0.6339	0.0635	0.5441	HUWE1
Q9BYI3;Q8IXS8	0.0163	0.9122	-0.2151	0.1187	HYCC1
Q9Y4L1	0.0522	0.5602	-0.0365	0.8013	HYOU1
Q2TAA2	-0.1261	0.664	-0.2805	0.2901	IAH1
P41252	0.0336	0.7051	0.1006	0.499	IARS1
Q9NSE4	-0.0526	0.4075	-0.013	0.8967	IARS2
P05362	-0.2888	0.3605	-0.3704	0.1038	ICAM1
P14735	0.0464	0.6257	-0.06	0.4997	IDE
O75874	-0.1712	0.382	-0.0349	0.831	IDH1
P48735	0.1859	0.2418	-0.1069	0.3432	IDH2
P50213	-0.1472	0.0419	0.0338	0.7687	IDH3A
Q9Y5U9	-0.0127	0.8877	-0.1611	0.4728	IER3IP1
Q16666	-0.0367	0.7986	-0.1315	0.2786	IFI16
P80217	0.0421	0.6014	-0.0203	0.8376	IFI35
O14879	0.1535	0.1953	0.2404	0.2016	IFIT3
Q13325	0.0436	0.7911	-0.0022	0.9813	IFIT5
P13164;Q01628;Q01629	0.1148	0.3792	-0.2902	0.167	IFITM2;IFITM3
Q01628	0.168	0.1942	-0.2152	0.233	IFITM3
Q9Y547	0.0808	0.6286	0.006	0.9571	IFT25
Q9NZI8	-0.0607	0.6985	-0.0622	0.6883	IGF2BP1
O00425;Q9NZI8;Q9Y6M1	0.1049	0.5071	0.0911	0.5257	IGF2BP1;IGF2BP2;IGF2BP
Q9Y6M1	0.0253	0.7557	0.0206	0.7937	IGF2BP2
O00425	0.1772	0.5549	0.3641	0.094	IGF2BP3
P11717	0.0019	0.9857	-0.0431	0.7256	IGF2R
Q70UQ0	-0.1224	0.1922	-0.2039	0.0092	IKBIP
Q12905	0.0658	0.4033	-0.0543	0.3475	ILF2
Q12906	-0.0364	0.6002	-0.0726	0.0716	ILF3
Q12906;Q96SI9	0.0873	0.4855	-0.1685	0.0502	ILF3;STRBP
Q13418	-0.0478	0.5942	0.0879	0.5712	ILK

A1L0T0	-0.047	0.7588	0.0358	0.6771	ILVBL
Q16891	0.0266	0.7081	0.0179	0.8043	IMMT
P29218	-0.0731	0.4288	0.0335	0.7889	IMPA1
P12268	0.0114	0.9258	0.0115	0.897	IMPDH2
Q27J81	-0.0046	0.9597	0.0686	0.3568	INF2
Q8TEX9	-0.2454	0.4163	-0.0045	0.9843	IPO4
O00410	-0.0586	0.4621	0.0234	0.7646	IPO5
O00410;O60518	0.0682	0.3937	-0.0132	0.9058	IPO5;RANBP6
O95373	-0.0987	0.2095	0.0292	0.7381	IPO7
Q96P70	0.0522	0.5711	0.1122	0.4535	IPO9
P46940	0.0047	0.9113	-0.046	0.246	IQGAP1
Q9NWZ3	-0.0635	0.8069	0.0411	0.8476	IRAK4
P05161	0.3417	0.147	-0.2267	0.3998	ISG15
P53990	-0.0674	0.483	-0.0633	0.3087	IST1
P56199	-0.3329	0.2303	-0.0896	0.636	ITGA1
P17301	0.3923	0.1931	0.43	0.0678	ITGA2
P13612	0.0227	0.9485	0.1565	0.3994	ITGA4
P08648	-0.0803	0.282	0.0215	0.8064	ITGA5
P06756	-0.0819	0.2807	0.0346	0.6315	ITGAV
P05556	-0.0225	0.8937	0.1228	0.0646	ITGB1
P18084	0.0046	0.9813	0.0054	0.9755	ITGB5
Q06033	-0.1428	0.7869	-0.181	0.2987	ITIH3
Q9BY32	-0.4968	0.0168	-0.0957	0.5203	ITPA
Q14573	0.0723	0.6225	-0.0883	0.5965	ITPR3
Q15811	0.0784	0.5289	-0.1086	0.1178	ITSN1
P26440	-0.0919	0.6763	-0.1806	0.2408	IVD
Q8N5M9	0.1555	0.2164	0.1353	0.3296	JAGN1
P23458	0.0154	0.9442	-0.1809	0.41	JAK1
Q9UK76	0.2023	0.0829	0.1228	0.3232	JPT1
Q9H910	-0.0718	0.3883	-0.0908	0.3556	JPT2
Q63ZY3	0.0563	0.4539	0.0404	0.5848	KANK2
Q15046	0.0282	0.7314	-0.071	0.4044	KARS1
Q8IYT4	-0.0666	0.2574	-0.0587	0.3868	KATNAL2
Q96CX2	-0.4192	0.2675	0.1199	0.7526	KCTD12
Q06136	-0.044	0.727	-0.1151	0.4302	KDSR
Q07666	0.192	0.5109	0.461	0.012	KHDRBS1
Q92945	-0.0611	0.4956	-0.0428	0.4632	KHSRP
O15066	-0.3351	0.4682	0.6721	0.4161	KIF3B
O60282;P33176;Q12840	-0.0819	0.1917	0.043	0.4907	KIF5B
Q07866	-0.0252	0.7558	-0.0152	0.7964	KLC1
Q07866;Q9H0B6;Q9NSK0	0.0442	0.6186	0.0625	0.5836	KLC1;KLC4
P52294	-0.0106	0.9633	0.2068	0.0169	KPNA1
P52292	0.1744	0.5284	0.2245	0.3825	KPNA2
O00505	0.0647	0.6385	-0.0752	0.5862	KPNA3
O00505;O00629	-0.3224	0.04	0.0089	0.9398	KPNA3;KPNA4
O60684	0.0497	0.462	-0.0505	0.6409	KPNA6
Q14974	-0.0085	0.8427	0.0451	0.3196	KPNB1
P04264	0.7386	0.2092	0.0574	0.8384	KRT1

P04264;P35908;Q7Z794	1.016	0.2171	0.2416	0.5292	KRT1;KRT2;KRT77
P13645	0.502	0.2062	0.2588	0.6162	KRT10
P02533;P08779;P13645;Q7Z3Y7;Q7Z3Y8;Q7Z3Y9;Q7Z3Z0	0.3736	0.3222	0.1827	0.657	KRT10;KRT14;KRT16;KRT28
P35908	0.2588	0.3339	0.1161	0.7803	KRT2
O95678;P02538;P04259;P13647;P35908;P48668;Q5XKE5	0.8012	0.1608	0.5333	0.314	KRT2;KRT5;KRT6B
P35527	0.723	0.2321	-0.071	0.6864	KRT9
Q8N6L1	0.0383	0.7904	-0.0731	0.4219	KRTCAP2
Q86UP2	0.0419	0.3669	0.0271	0.5811	KTN1
Q6YP21	-0.268	0.0829	-0.0253	0.8758	KYAT3
Q53H82	0.0877	0.4578	0.1425	0.1196	LACTB2
P55268	0.0727	0.5439	-0.0638	0.5343	LAMB2
P11047	0.106	0.4788	0.044	0.69	LAMC1
P11279	-0.1202	0.3343	-0.2026	0.0925	LAMP1
P13473	-0.2587	0.1127	-0.2661	0.0123	LAMP2
Q6IAA8	0.0041	0.9565	-0.0056	0.9372	LAMTOR1
Q9Y2Q5	-0.0531	0.523	0.0959	0.2385	LAMTOR2
Q9UHA4	-0.0647	0.6814	-0.0125	0.9112	LAMTOR3
O43504	0.1187	0.383	0.0191	0.8611	LAMTOR5
O43813	0.1117	0.2998	0.024	0.8165	LANCL1
P28838	0.066	0.4988	-0.1061	0.3735	LAP3
Q659C4;Q6PKG0	-0.1271	0.2779	-0.0521	0.6788	LARP1
Q92615	-0.11	0.8354	0.0668	0.7946	LARP4B
Q9P2J5	0.0544	0.4639	0.0297	0.8272	LARS1
Q14847	0.1136	0.2878	0.0385	0.6182	LASP1
P00338	-0.1018	0.6371	0.0434	0.6598	LDHA
P00338;P07195;P07864;Q6ZMR3	-0.1192	0.5764	0.1378	0.2408	LDHA;LDHB;LDHC
P07195	0.033	0.5231	0.1193	0.079	LDHB
Q8NC56	-0.0293	0.8095	-0.0227	0.8109	LEMD2
O95202	0.0487	0.5093	0.0804	0.2185	LETM1
P09382	-0.0831	0.3216	-0.038	0.7025	LGALS1
P17931	-0.1552	0.2542	-0.199	0.1679	LGALS3
Q9H008	0.0314	0.8674	-0.1323	0.2389	LHPP
Q9UHB6	0.0549	0.6776	0.0359	0.8923	LIMA1
P48059	-0.0118	0.9224	0.0946	0.4189	LIMS1
Q9NUP9	-0.0229	0.8943	-0.0699	0.6296	LIN7C
P49257	0.0069	0.946	-0.0784	0.2045	LMAN1
Q12907	-0.1905	0.0092	-0.0415	0.5213	LMAN2
Q9NZU5	0.3092	0.3144	0.1515	0.6793	LMCD1
Q9BU23	-0.0608	0.664	-0.0522	0.5988	LMF2
P02545	0.028	0.8205	-0.0818	0.5019	LMNA
P02545;P20700;Q03252	0.0053	0.9746	-0.2715	0.0932	LMNA;LMNB1;LMNB2
P20700	0.0442	0.7695	-0.1085	0.2235	LMNB1
Q03252	0.0133	0.8529	-0.0019	0.9723	LMNB2
Q8WWI1	0.0866	0.7511	0.2341	0.4692	LMO7
Q9UIQ6	-0.0056	0.9622	-0.0158	0.8902	LNPEP
Q9C0E8	-0.1003	0.1628	0.1366	0.1248	LNPK

P36776	-0.0236	0.8101	0.02	0.8	LONP1
Q93052	0.0111	0.9096	0.072	0.5306	LPP
O60711	0.0427	0.8479	0	0.9999	LPXN
Q07954	-0.1279	0.1945	-0.0513	0.6411	LRP1
P30533	-0.0929	0.507	-0.1425	0.3689	LRPAP1
P42704	0.027	0.6488	0.014	0.868	LRPPRC
Q9H9A6	0.0858	0.3726	0.1402	0.1497	LRRC40
Q8N1G4	-0.0271	0.7338	0.0301	0.527	LRRC47
Q8N9N7	-0.1798	0.5258	-0.3682	0.0152	LRRC57
Q96AG4	0.0265	0.6475	0.0107	0.9121	LRRC59
Q32MZ4	0.1498	0.2692	-0.0009	0.9955	LRRFIP1
Q32MZ4;Q9Y608	0.2898	0.2164	0.5787	0.0127	LRRFIP1;LRRFIP2
Q9Y608	0.1751	0.1381	0.0361	0.7674	LRRFIP2
Q3MHD2	0.1941	0.1273	0.0171	0.8919	LSM12
Q9Y333	-0.0959	0.5422	0.0744	0.1625	LSM2
P62310	0.0126	0.8896	0.0241	0.7004	LSM3
Q9Y4Z0	-0.1037	0.4613	-0.0354	0.7457	LSM4
P62312	0.1462	0.2032	0.0896	0.3438	LSM6
O95777	-0.1368	0.3788	-0.0411	0.6713	LSM8
P48449	-0.1338	0.4168	-0.2091	0.0572	LSS
P09960	0.1914	0.0116	0.1287	0.1724	LTA4H
P02788	0.0225	0.8996	-0.2735	0.0353	LTF
Q9NQ29;Q9Y383	0.0822	0.416	0.0151	0.8826	LUC7L2
O95232	0.0916	0.7359	-0.0268	0.9308	LUC7L3
Q86V48	-0.104	0.5109	0.1845	0.4414	LUZP1
O95372	0.36	0.093	0.0156	0.9266	LYPLA2
Q5VWZ2	0.195	0.1339	0.3039	0.1113	LYPLAL1
Q8WZA0	0.1253	0.2991	-0.1427	0.1833	LZIC
Q9UPN3	-0.0403	0.8123	0.0012	0.996	MACF1
O75367	-0.0546	0.4248	0.001	0.9873	MACROH2A1
O75367;Q9P0M6	-0.0317	0.9047	-0.1055	0.3402	MACROH2A1;MACROH2A
Q9P0M6	0.0112	0.9513	-0.1045	0.3784	MACROH2A2
Q9UNF1;Q9Y5V3	0.0237	0.8857	0.3212	0.068	MAGED2
Q16706	-0.0444	0.748	0.044	0.6947	MAN2A1
O00754	0.1426	0.2089	-0.172	0.2186	MAN2B1
P55145	0.0493	0.5403	0.067	0.4807	MANF
P78559	0.0194	0.7958	0.0951	0.5665	MAP1A
P46821;P78559	0.1258	0.4125	0.2874	0.0905	MAP1A;MAP1B
P46821	0.0944	0.5913	0.3202	0.0288	MAP1B
Q9GZQ8;A6NCE7	-0.0239	0.8708	0.1487	0.4264	MAP1LC3B;MAP1LC3B2
Q66K74	0.035	0.747	0.1523	0.2856	MAP1S
Q02750	0.1474	0.4653	0.0035	0.9776	MAP2K1
P36507;Q02750	-0.4399	0.0876	0.0715	0.5664	MAP2K1;MAP2K2
P36507	-0.0333	0.7248	-0.0486	0.6185	MAP2K2
P46734	0.1407	0.602	0.0298	0.8001	MAP2K3
P27816	0.069	0.3446	0.0819	0.1031	MAP4
O95819;Q8N4C8;Q9UKE5	-0.1653	0.0757	-0.0033	0.9497	MAP4K4
Q14244;Q3KQU3	-0.0684	0.7849	0.4123	0.4518	MAP7D1

P28482	-0.0198	0.704	-0.0421	0.5856	MAPK1
P27361;P28482	-0.1148	0.3578	-0.0398	0.6935	MAPK1;MAPK3
Q16539	0.0588	0.5259	-0.0135	0.9453	MAPK14
P27361	-0.0125	0.8383	0.0996	0.0972	MAPK3
Q15691	0.1207	0.1141	-0.1261	0.1899	MAPRE1
P29966	-0.1575	0.5715	-0.1976	0.2537	MARCKS
P56192	-0.1351	0.2644	-0.0298	0.7578	MARS1
P31153	-0.0525	0.2125	-0.0512	0.3737	MAT2A
Q9NZL9	0.2116	0.0275	0.0186	0.7959	MAT2B
P43243	0.046	0.3919	-0.051	0.3228	MATR3
Q7Z434	0.0785	0.5045	-0.1824	0.1859	MAVS
Q5VZF2;Q9NR56	0.0359	0.6382	-0.0432	0.5108	MBNL1;MBNL2
Q96N66	0.0188	0.8925	0.0354	0.7884	MBOAT7
Q9HCC0	-0.0048	0.9732	0.1409	0.3883	MCCC2
Q8NI22	-0.1557	0.3446	0.0293	0.8052	MCFD2
Q9ULC4	-0.0719	0.5007	0.0441	0.6464	MCTS1
Q8NE86	-0.0797	0.4764	0.0704	0.5068	MCU
P40925	-0.0499	0.5002	-0.0414	0.4855	MDH1
P40926	0.0327	0.6542	0.036	0.7676	MDH2
P48163	0.2756	0.2433	0.3497	0.1894	ME1
P23368	-0.0619	0.403	0.0059	0.9582	ME2
Q6P9B6	0.0748	0.5356	0.0571	0.7162	MEAK7
P51608	0.4121	0.1071	0.0263	0.9138	MECP2
Q9Y316	0.1811	0.1848	0.0332	0.8595	MEMO1
Q14696	-0.075	0.6836	-0.0341	0.7207	MESD
P50579	-0.1585	0.177	0.0663	0.503	METAP2
P26572	-0.2024	0.1623	0.0533	0.4949	MGAT1
P10620	0.0442	0.8394	0.1044	0.471	MGST1
O14880	0.0118	0.8818	-0.0373	0.8266	MGST3
Q96PC5;A4D2H0;Q86UF2;Q96RT6	0.1763	0.395	0.4243	0.1819	MIA2
Q5JRA6	-0.1906	0.1272	-0.1182	0.1096	MIA3
Q8TDZ2	-0.0677	0.4582	-0.0303	0.7691	MICAL1
P14174	0.1313	0.2642	0.0361	0.8686	MIF
Q14165	-0.1256	0.1747	0.0326	0.544	MLEC
P08473	-0.1532	0.4786	-0.3805	0.0777	MME
P50281	-0.1572	0.3547	-0.2491	0.1588	MMP14
P22033	0.1037	0.1927	-0.0205	0.8988	MMUT
Q7L9L4;Q9H8S9	0.0296	0.8164	0.1838	0.014	MOB1A;MOB1B
Q9Y3A3	0.0641	0.5506	-0.0727	0.5559	MOB4
Q13724	-0.0027	0.9773	0.0073	0.9142	MOGS
Q6UVY6	-0.2735	0.2208	-0.1664	0.5074	MOXD1
O95563	0.1394	0.5234	0.1241	0.6062	MPC2
O75352	-0.1545	0.3715	-0.0552	0.7125	MPDU1
P34949	-0.0527	0.5739	0.029	0.8015	MPI
Q6WCQ1	0.1136	0.5974	0.1362	0.4036	MPRIP
O95297	-0.1226	0.6622	-0.1216	0.5804	MPZL1
Q9UBG0	-0.2161	0.2669	-0.1123	0.2882	MRC2
P49959	0.1498	0.2037	0.0067	0.9454	MRE11

Q9BV20	0.2432	0.2333	0.2621	0.1851	MRI1
Q9BYC9	0.2917	0.0976	0.4309	0.057	MRPL20
Q9BZE1	0.0567	0.6504	0.0873	0.5885	MRPL37
Q9Y3D9	0.0833	0.5439	-0.3351	0.0557	MRPS23
Q92552	0.1701	0.3368	0.0032	0.9875	MRPS27
P26038	0.0367	0.6144	0.0285	0.7229	MSN
P26038;P35241	-0.1139	0.4062	0.2081	0.3959	MSN;RDX
P00846	-0.0274	0.6334	0.032	0.7418	MT-ATP6
P03928	-0.0596	0.5048	-0.0303	0.6344	MT-ATP8
P00403	-0.0051	0.9676	0.078	0.6166	MT-CO2
P03905	0.019	0.8281	-0.0521	0.775	MT-ND4
P03915	0.0014	0.9905	0.0024	0.9911	MT-ND5
O94776;Q13330;Q9BTC8	-0.2907	0.1671	-0.0243	0.8958	MTA2
Q13126	0.1669	0.0498	0.0599	0.619	MTAP
Q9NZJ7	0.0462	0.8271	0.0681	0.5552	MTCH1
Q9Y6C9	-0.0152	0.8604	-0.0325	0.7469	MTCH2
Q86UE4	0.0263	0.6113	-0.0503	0.4734	MTDH
P11586	-0.0399	0.6719	-0.0247	0.7005	MTHFD1
Q6UB35	-0.0406	0.7911	0.3106	0.0131	MTHFD1L
Q9Y217	0.05	0.7345	0.0266	0.7974	MTMR6
P42345	0.2072	0.1702	0.1215	0.5185	MTOR
P58546	-0.0534	0.7288	-0.0245	0.796	MTPN
O75431	0.2427	0.1897	0.2244	0.1597	MTX2
Q14764	0.0534	0.5809	0.0623	0.5806	MVP
Q96S97	0.0682	0.5564	0.1478	0.3833	MYADM
Q9BQG0	-0.0305	0.8481	-0.0012	0.9932	MYBBP1A
Q969H8	-0.1172	0.1324	0.0671	0.297	MYDGF
Q9HB07	-0.155	0.299	-0.1891	0.0774	MYG1
P35580	0.2286	0.3036	0.4108	0.2632	MYH10
P35580;P35749	0.2387	0.1813	0.4139	0.2213	MYH10;MYH11
P35579;P35580;P35749;Q7Z406	-0.0025	0.9889	0.1338	0.6611	MYH10;MYH11;MYH14;MY
P35579;P35580;P35749	0.0312	0.9145	0.1848	0.6038	MYH10;MYH11;MYH9
P35579;P35580	0.1056	0.6489	0.2561	0.4601	MYH10;MYH9
P35579;P35749;Q7Z406	0.0099	0.962	0.2487	0.4705	MYH11;MYH14;MYH9
P35579;P35749	0.0763	0.7397	0.1173	0.6336	MYH11;MYH9
P35579	0.0216	0.9187	0.1154	0.6975	MYH9
P05976;P08590	0.1477	0.2749	0.206	0.3067	MYL1;MYL3
O14950;P19105	-0.0038	0.9844	0.0127	0.952	MYL12A;MYL12B
P60660	0.0577	0.713	0.1482	0.5693	MYL6
P24844	0.0989	0.6031	0.1756	0.6063	MYL9
Q92614	0.2747	0.1476	-0.0436	0.8521	MYO18A
O43795	0.1511	0.4331	-0.0048	0.9786	MYO1B
O00159	0.0593	0.371	0.0103	0.786	MYO1C
B0I1T2;O94832	-0.1603	0.4119	-0.1383	0.4217	MYO1D
Q12965	-0.124	0.276	0.1144	0.3922	MYO1E
Q9UM54	-0.0563	0.327	0.0742	0.4113	MYO6
Q9NZM1	-0.2183	0.0631	-0.2736	0.0489	MYOF
P41227;Q9BSU3	-0.2763	0.2159	0.3047	0.17	NAA10

Q9BXJ9	-0.0389	0.6828	-0.0408	0.6792	NAA15
Q13765;E9PAV3	-0.0899	0.2522	-0.0074	0.9019	NACA
Q13765;E9PAV3;Q9BZK3	0.1375	0.2998	0.2611	0.0484	NACA4P
Q4G0N4	-0.2929	0.1057	-0.0581	0.6182	NADK2
Q13564	0.0488	0.5963	-0.1255	0.1432	NAE1
P17050	0.1784	0.3136	-0.174	0.3408	NAGA
Q9UJ70	-0.1036	0.2492	-0.0677	0.4659	NAGK
P54802	0.1815	0.2864	-0.1757	0.2074	NAGLU
P43490	-0.0488	0.791	-0.1063	0.5181	NAMPT
Q9NR45	-0.0196	0.8592	-0.0643	0.478	NANS
P55209	-0.0132	0.8346	0.056	0.4675	NAP1L1
P55209;Q99733	-0.3004	0.08	0.0734	0.6762	NAP1L1;NAP1L4
Q99733	-0.0081	0.9274	-0.0138	0.8483	NAP1L4
P54920	0.0565	0.4094	-0.0458	0.5742	NAPA
Q99747	0.201	0.4076	-0.0328	0.8255	NAPG
O43776	0.0602	0.5683	0.0306	0.8508	NARS1
P49321	0.1155	0.677	0.161	0.2819	NASP
Q8IW45	-0.1334	0.4625	-0.1732	0.0758	NAXD
Q8NCW5	0.0327	0.7183	-0.1937	0.0173	NAXE
A2RRP1	-0.0826	0.4113	0.1744	0.2325	NBAS
Q09161	0.1204	0.3807	0.0513	0.6932	NCBP1
Q6PIU2	0.0812	0.7426	-0.0853	0.5494	NCEH1
P16333	-0.1263	0.1259	0.0354	0.7001	NCK1
Q9Y2A7	-0.0234	0.7095	-0.0637	0.2232	NCKAP1
P19338	-0.0546	0.5896	-0.0124	0.8592	NCL
Q969V3	-0.0419	0.5012	0.0249	0.7834	NCLN
Q92542	-0.0776	0.4714	-0.1337	0.2524	NCSTN
Q92597	0.0603	0.5576	-0.119	0.2706	NDRG1
Q9UGV2	0.0446	0.6723	0.068	0.6954	NDRG3
O95299	-0.0413	0.7587	-0.004	0.9675	NDUFA10
Q86Y39	-0.0732	0.5673	0.1309	0.4472	NDUFA11
Q9UI09	-0.2589	0.0174	-0.018	0.8504	NDUFA12
O43678	-0.0351	0.7124	0.0035	0.973	NDUFA2
O00483	0.1056	0.192	0.0685	0.5552	NDUFA4
Q16718	0.0224	0.8408	0.0387	0.6985	NDUFA5
O95182	-0.0788	0.446	-0.1208	0.1534	NDUFA7
P51970	-0.003	0.9761	-0.0257	0.7874	NDUFA8
Q16795	-0.0036	0.9649	0.0018	0.9874	NDUFA9
O14561	0.0935	0.452	-0.0638	0.652	NDUFAB1
O96000	-0.0271	0.8382	-0.0032	0.9759	NDUFB10
O43676	-0.0406	0.6053	-0.164	0.185	NDUFB3
O95168	-0.0518	0.6313	-0.107	0.3361	NDUFB4
O43674	0.1299	0.1186	-0.1153	0.3443	NDUFB5
O95169	-0.076	0.5284	-0.0686	0.5244	NDUFB8
Q9Y6M9	0.0169	0.8828	-0.2427	0.0452	NDUFB9
P28331	0.0338	0.7031	-0.0347	0.7163	NDUFS1
O75306	-0.0314	0.6608	0.0315	0.5908	NDUFS2
O75489	-0.0214	0.861	-0.0391	0.6074	NDUFS3

O43920	0.0374	0.8059	0.038	0.8013	NDUFS5
O75251	-0.12	0.3058	0.041	0.8225	NDUFS7
O00217	-0.1909	0.1133	-0.2923	0.1088	NDUFS8
P49821	-0.1043	0.3094	0.0273	0.8037	NDUFV1
P19404	-0.0008	0.9938	0.0336	0.843	NDUFV2
Q9NVZ3	-0.1598	0.0449	0.0674	0.5583	NECAP2
Q15843	-0.2714	0.0953	-0.0089	0.9649	NEDD8
Q8TDX7	0.0463	0.8954	0.0364	0.8831	NEK7
Q8TD19	-0.063	0.3921	-0.0008	0.9875	NEK9
Q9UMX5	-0.1094	0.5303	-0.1354	0.2027	NENF
Q99519	0.1128	0.484	-0.0371	0.8595	NEU1
Q0ZGT2	0.1427	0.6098	0.3103	0.3913	NEXN
O00712;P08651;Q12857;Q14938	-0.1753	0.251	-0.0906	0.176	NFIB;NFIC;NFIX
Q14938	0.1698	0.2327	-0.0237	0.8551	NFIX
P19838	-0.0052	0.9534	0.0293	0.809	NFKB1
Q9UMS0	0.0492	0.6873	-0.1056	0.6569	NFU1
O14745	-0.1	0.2186	-0.0463	0.6541	NHERF1
Q8NBF2	-0.0529	0.661	-0.02	0.8437	NHLRC2
Q9NX24	-0.0435	0.7955	0.0446	0.7421	NHP2
Q9BZQ8	-0.3064	0.1944	0.1129	0.5896	NIBAN1
Q96TA1	0.0293	0.8183	-0.01	0.8901	NIBAN2
Q9GZT8	0.1646	0.23	0.3457	0.032	NIF3L1
Q8N4C6	0.0076	0.9794	0.1804	0.3782	NIN
Q9BPW8	0.0231	0.8371	0.0578	0.6881	NIPSNAP1
O75323	-0.197	0.122	0.0558	0.6814	NIPSNAP2
Q9UFN0	-0.0575	0.4505	-0.0084	0.9239	NIPSNAP3A
Q86X76	-0.0072	0.9727	-0.3244	0.2505	NIT1
Q9NQR4	0.0354	0.5725	-0.0472	0.3839	NIT2
P15531	-0.035	0.6934	-0.0101	0.8857	NME1
P15531;P22392;O60361	-0.0761	0.193	-0.0573	0.4363	NME1;NME2
P22392;O60361	-0.0173	0.672	-0.0406	0.5502	NME2
Q13232	-0.2531	0.3392	-0.1404	0.5054	NME3
P30419	0.0226	0.6157	0.0442	0.6197	NMT1
P40261	0.2718	0.1752	-0.1542	0.6244	NNMT
Q13423	0.0936	0.0273	0.0616	0.5887	NNT
P69849;Q15155;Q5JPE7	-0.0163	0.8114	0.0417	0.207	NOMO1;NOMO2;NOMO3
Q15233	-0.0309	0.6747	-0.0412	0.4073	NONO
P46087	-0.1231	0.5443	-0.034	0.8355	NOP2
O00567	0.0304	0.6696	-0.0023	0.9685	NOP56
Q9Y2X3	0.0577	0.5145	-0.0694	0.2583	NOP58
Q04721	0.0396	0.8236	-0.0116	0.9148	NOTCH2
O15118	0.044	0.7187	-0.2927	0.2074	NPC1
P61916	-0.221	0.1579	-0.1861	0.1516	NPC2
Q8NDH3	-0.0502	0.6908	0.0313	0.6867	NPEPL1
P55786;A6NEC2	0.0277	0.5681	0.0014	0.9772	NPEPPS
Q8TAT6	0.0164	0.9191	0.2444	0.1401	NPLOC4
P06748	0.0159	0.8874	0.085	0.2887	NPM1
Q9Y639	-0.028	0.8599	0.0221	0.8659	NPTN

P16083	-0.4518	0.233	-0.2041	0.4157	NQO2
P04150	-0.0324	0.8045	0.1381	0.448	NR3C1
P01111;P01116	-0.0283	0.8547	-0.0297	0.8895	NRAS
Q9UHY1	-0.0397	0.8127	-0.0592	0.6062	NRBP1
O43847	0.1926	0.1586	-0.0814	0.4606	NRDC
Q15738	0.0344	0.7492	0.0171	0.86	NSDHL
P46459	-0.0612	0.1083	-0.0667	0.2674	NSF
Q9UNZ2	-0.0474	0.657	0.0683	0.3458	NSFL1C
Q08J23	-0.1652	0.3518	0.0058	0.9454	NSUN2
P21589	-0.2439	0.3198	-0.113	0.6933	NT5E
Q9BSD7	-0.1544	0.4068	-0.0637	0.5519	NTPCR
Q02818	0.0035	0.9764	-0.1785	0.0204	NUCB1
P80303	-0.2963	0.1003	-0.1159	0.3815	NUCB2
Q9Y266	0.0839	0.4207	0.0898	0.3718	NUDC
Q8WVJ2	-0.3006	0.1481	-0.119	0.4223	NUDCD2
Q8IVD9	-0.0606	0.5137	-0.1021	0.4495	NUDCD3
O43809	0.1004	0.3707	-0.1124	0.2894	NUDT21
Q9NZJ9;A0A024RBG1	-0.0781	0.6953	-0.6266	0.0425	NUDT4
Q9UKK9	0.0099	0.8893	0.0179	0.8158	NUDT5
Q14980	-0.0567	0.5883	-0.1809	0.0877	NUMA1
Q9Y6R0	0.2472	0.2178	0.1136	0.6171	NUMBL
P49790	0.058	0.7274	-0.0889	0.5585	NUP153
O75694	-0.0837	0.5045	0.0975	0.1827	NUP155
Q12769	-0.1832	0.0681	-0.0801	0.4825	NUP160
Q92621	0.1355	0.2047	-0.037	0.6589	NUP205
Q8NFH5	0.108	0.3978	-0.0551	0.6156	NUP35
Q7Z3B4	0.0667	0.5699	-0.062	0.5502	NUP54
Q99567	0.0652	0.6374	-0.4219	0.0113	NUP88
Q8N1F7	-0.0188	0.8004	0.0133	0.8358	NUP93
P52948	0.167	0.1807	0.0204	0.6916	NUP98
P61970	0.0389	0.6896	-0.0925	0.4059	NUTF2
P04181	-0.095	0.1814	0.0228	0.8142	OAT
Q8TAD7	-0.0158	0.9442	-0.2525	0.4109	OCC1
Q9NX40	-0.0679	0.6147	0.0575	0.6566	OCIAD1
O60502	0.2716	0.2368	0.1988	0.2169	OGA
Q02218	-0.011	0.8856	0.0052	0.9728	OGDH
O15294	-0.0121	0.9054	-0.1946	0.2412	OGT
Q9NTK5	-0.0462	0.35	-0.0377	0.5925	OLA1
O60313	-0.0137	0.8907	-0.0333	0.7804	OPA1
Q96CV9	0.0524	0.5802	0.1252	0.3216	OPTN
P22059	0.0226	0.7053	0.0894	0.1294	OSBP
Q9H4L5	-0.0684	0.6623	-0.1057	0.5871	OSBPL3
Q9BZF1	0.1209	0.3537	0.1405	0.3733	OSBPL8
Q96SU4	0.0001	0.9992	0.0796	0.536	OSBPL9
Q9NPF4	0.0329	0.7872	-0.028	0.8584	OSGEP
Q9NRP0	0.0121	0.8908	-0.1566	0.1429	OSTC
Q92882	-0.0872	0.5343	-0.003	0.9728	OSTF1
Q96FW1	-0.0268	0.6055	0.0088	0.9001	OTUB1

Q8WZ82	-0.2835	0.1445	0.0437	0.8072	OVCA2
P55809;Q9BYC2	-0.0807	0.551	-0.0144	0.933	OXCT1
O95747	0.1114	0.4455	-0.086	0.6519	OXSR1
Q99571	-0.2464	0.2912	-0.4163	0.1427	P2RX4
Q32P28	-0.0723	0.6427	-0.1119	0.305	P3H1
Q92791	0.2339	0.1633	0.2539	0.2474	P3H4
P13674	0.0521	0.5337	0.0325	0.7595	P4HA1
O15460	0.0413	0.8201	0.022	0.8959	P4HA2
P07237	-0.0523	0.2435	-0.0283	0.7302	P4HB
Q9UQ80	0.0177	0.7554	0.01	0.8049	PA2G4
P11940;Q4VXU2	0.061	0.2824	0.0649	0.4559	PABPC1
P11940;Q13310	0.0506	0.2803	0.0003	0.9962	PABPC1;PABPC4
Q13310	0.1123	0.1087	-0.0369	0.7195	PABPC4
Q86U42	0.0477	0.5846	-0.1792	0.0173	PABPN1
Q6VY07	-0.0123	0.9597	-0.118	0.2021	PACS1
Q9UNF0	-0.1059	0.4177	-0.0333	0.6915	PACSIN2
P43034	0.0223	0.5435	0.0868	0.2291	PAFAH1B1
P68402	0.0526	0.5545	0.0134	0.8917	PAFAH1B2
Q15102	-0.0016	0.9917	0.0061	0.9668	PAFAH1B3
P22234	-0.0512	0.4707	0.0113	0.895	PAICS
Q9H074	0.0489	0.7215	0.1217	0.3536	PAIP1
O75914;Q13153;Q13177	0.0954	0.3866	0.1198	0.1381	PAK1;PAK2
Q13177	0.0416	0.6076	-0.0562	0.1836	PAK2
Q8WX93	0.5184	0.1005	0.5707	0.1451	PALLD
O75781	-0.7499	0.014	0.1791	0.5439	PALM
O43252	-0.1993	0.0837	-0.3153	0.0207	PAPSS1
Q99497	-0.0646	0.2953	-0.0188	0.6959	PARK7
P09874	-0.034	0.7085	-0.0257	0.6953	PARP1
Q9UKK3	0.0033	0.9695	0.076	0.4597	PARP4
Q8IXQ6	-0.0146	0.9459	-0.319	0.114	PARP9
Q9HBI1;Q9NVD7	-0.1371	0.4291	-0.0008	0.9958	PARVA
Q9BUH6	-0.0605	0.6961	0.0305	0.716	PAXX
Q9BVG4	0.0369	0.7068	-0.129	0.035	PBDC1
Q15365	-0.0588	0.2913	-0.0412	0.3549	PCBP1
P57721;Q15365;Q15366	0.0008	0.9949	-0.0833	0.1855	PCBP1;PCBP2
Q15366	0.0389	0.522	0.0112	0.8495	PCBP2
Q16822	-0.0582	0.8244	0.0725	0.79	PCK2
P22061	0.0159	0.8399	-0.0064	0.9328	PCMT1
P12004	-0.0845	0.5111	0.0706	0.2806	PCNA
Q8WW12	-0.128	0.4642	-0.0563	0.72	PCNP
Q15113	0.2168	0.3842	-0.0979	0.7085	PCOLCE
Q9UHG3	-0.1351	0.1921	-0.1298	0.4407	PCYOX1
P49585	-0.1506	0.2488	-0.0239	0.7713	PCYT1A
Q99447	-0.1446	0.3454	-0.0047	0.9715	PCYT2
Q13442	-0.0432	0.5811	-0.112	0.1309	PDAP1
Q9BUL8	0.0249	0.7522	-0.1138	0.1604	PDCD10
Q53EL6	-0.0506	0.7261	-0.1	0.6681	PDCD4
O14737	0.1935	0.0584	0.0916	0.2064	PDCD5

O75340	0.0109	0.9296	-0.0205	0.7814	PDCD6
Q8WUM4	-0.048	0.3853	0.0763	0.3013	PDCD6IP
P09619	-0.3768	0.0386	-0.0926	0.4423	PDGFRB
P08559	-0.0549	0.4266	-0.0262	0.8021	PDHA1
P11177	-0.0517	0.5865	-0.0336	0.6664	PDHB
O00330	-0.0932	0.3902	0.0655	0.7397	PDHX
P30101	-0.0717	0.2702	-0.0649	0.2815	PDIA3
P13667	-0.0953	0.2076	-0.048	0.2814	PDIA4
Q14554	-0.128	0.4105	-0.1243	0.2612	PDIA5
Q15084	-0.0719	0.3592	-0.0464	0.4483	PDIA6
O00151	0.6436	0.0154	0.0681	0.7645	PDLIM1
Q96JY6	0.4104	0.028	0.1649	0.5082	PDLIM2
P50479	0.1818	0.5332	0.3791	0.3475	PDLIM4
Q96HC4	-0.0284	0.929	0.2334	0.4485	PDLIM5
Q9NR12	0.162	0.6722	0.421	0.4451	PDLIM7
Q9P0J1	-0.05	0.7427	-0.0364	0.8318	PDP1
Q8NCN5	-0.1396	0.2427	-0.0322	0.8414	PDPR
Q6P996	-0.1304	0.204	-0.154	0.2506	PDXDC1
O00764	-0.018	0.8265	0.0553	0.4354	PDXK
Q15121	-0.0492	0.7484	0.0483	0.5549	PEA15
Q9H792	-0.292	0.5989	0.0632	0.5608	PEAK1
P30086	-0.0351	0.5876	-0.0482	0.3911	PEBP1
Q9UBV8	0.0763	0.4601	-0.0051	0.9692	PEF1
P12955	-0.0183	0.8481	-0.1278	0.0677	PEPD
O75381	0.0253	0.8253	0.0973	0.4609	PEX14
O15067	-0.0516	0.6097	-0.0032	0.9681	PFAS
O60925	0.1229	0.345	0.0293	0.8083	PFDN1
Q9UHV9	-0.0331	0.7482	-0.011	0.9109	PFDN2
Q9NQP4	-0.3294	0.1243	-0.1292	0.2242	PFDN4
Q99471	0.3278	0.0336	-0.0876	0.382	PFDN5
O15212	0.0568	0.4203	0.0391	0.4603	PFDN6
P17858	0.0817	0.2737	0.0709	0.5172	PFKL
P08237;P17858	-0.151	0.2561	-0.0415	0.7094	PFKL;PFKM
P08237;P17858;Q01813	-0.0305	0.8415	0.0909	0.4271	PFKL;PFKM;PFKP
P17858;Q01813	-0.0317	0.8171	0.1408	0.2174	PFKL;PFKP
P08237	-0.0878	0.3244	-0.1355	0.1674	PFKM
Q01813	-0.1117	0.4832	0.1919	0.1228	PFKP
P07737	0.0305	0.7369	0.0857	0.4549	PFN1
P35080	0.3287	0.1419	0.2548	0.0211	PFN2
P18669	-0.0027	0.944	0.0141	0.8464	PGAM1
P52209	-0.3194	0.0613	-0.2634	0.1314	PGD
P00558	0.0962	0.2518	0.0599	0.5839	PGK1
O95336	0.0136	0.8411	0.0835	0.3396	PGLS
P36871	0.0755	0.437	0.0904	0.4743	PGM1
Q96G03	0.0696	0.7432	-0.0889	0.6677	PGM2
O95394	-0.0536	0.5933	-0.0747	0.5213	PGM3
A6NDG6	-0.0266	0.8824	0.1118	0.4742	PGP
O00264	0.0031	0.9825	-0.1655	0.2584	PGRMC1

O00264;O15173	-0.0658	0.6299	-0.2737	0.0773	PGRMC1;PGRMC2
O15173	-0.044	0.6461	-0.0312	0.8433	PGRMC2
P35232	-0.0145	0.8687	-0.0873	0.4924	PHB1
Q99623	-0.0297	0.5935	-0.0774	0.4654	PHB2
O43175	0.0451	0.8752	0.3015	0.2822	PHGDH
Q9BTU6	-0.0477	0.7324	-0.1048	0.4243	PI4K2A
P42356	0.264	0.1332	-0.0441	0.5483	PI4KA
Q13492	-0.0655	0.4066	0.1949	0.2148	PICALM
Q96S52	-0.0449	0.7514	-0.0082	0.9473	PIGS
Q8NEB9	-0.0742	0.3234	0.1019	0.4075	PIK3C3
Q13526	0.0284	0.865	0.046	0.5299	PIN1
P48426	-0.266	0.0009	-0.1503	0.0505	PIP4K2A
P48426;P78356	-0.027	0.82	-0.0889	0.5537	PIP4K2A;PIP4K2B
Q99755;A2A3N6	0.0752	0.7017	0.1074	0.406	PIP5K1A
O60331	-0.2489	0.2816	-0.0928	0.5488	PIP5K1C
Q9GZP4	0.0244	0.8398	-0.1662	0.2489	PITHD1
Q00169	0.104	0.6708	-0.0376	0.8291	PITPNA
P48739;Q00169	0.125	0.28	-0.0831	0.5849	PITPNA;PITPNB
P48739	0.0666	0.5117	-0.0117	0.9136	PITPNB
Q5JRX3	-0.0365	0.7081	0.0655	0.7195	PITRM1
P14618;P30613	-0.0059	0.9585	0.0443	0.6747	PKLR;PKM
P14618	0.0195	0.8524	-0.0184	0.822	PKM
Q16512	0.0817	0.4831	-0.1342	0.2691	PKN1
Q16513	0.1567	0.0712	0.1429	0.0148	PKN2
Q9Y263	0.1074	0.3252	0.011	0.8892	PLAA
Q8NHP8	-0.1106	0.4111	-0.0824	0.5944	PLBD2
P51178	-0.0177	0.9193	-0.0954	0.6552	PLCD1
Q8N3E9	-0.0898	0.6215	-0.0644	0.6775	PLCD3
Q8IV08	-0.3769	0.0092	-0.0935	0.4103	PLD3
Q15149	0.1511	0.1035	0.1184	0.1531	PLEC
Q8TD55	0.1717	0.2225	0.0032	0.9811	PLEKHO2
O60664	0.04	0.5801	0.0399	0.6029	PLIN3
Q02809	-0.0072	0.9181	0.1439	0.2742	PLOD1
O00469	-0.1025	0.5549	0.4425	0.4576	PLOD2
O60568	-0.1177	0.1191	0.0421	0.5559	PLOD3
Q04941	0.0026	0.9667	-0.0654	0.1347	PLP2
O94903	0.077	0.5072	0.0732	0.606	PLPBP
O14495	0.0719	0.6978	0.0194	0.957	PLPP3
O43660	-0.0677	0.7556	-0.0161	0.9247	PLRG1
Q14651	-0.5059	0.0538	-0.2706	0.2433	PLS1
P13797;Q14651	-0.1551	0.3879	0.0503	0.8194	PLS1;PLS3
P13797	-0.1257	0.3069	0.005	0.9788	PLS3
O15031	0.0534	0.4635	-0.0762	0.3722	PLXNB2
P29590	0.1084	0.2889	0.0804	0.3168	PML
O15305	0.1539	0.1997	-0.0074	0.9365	PMM2
O75439	-0.0423	0.5251	0.021	0.8856	PMPCB
Q15126	-0.1767	0.2339	0.1474	0.4875	PMVK
Q9H307	0.0903	0.6568	-0.0596	0.7043	PNN

P00491	0.2236	0.2166	0.3636	0.0016	PNP
Q9NVS9	-0.0271	0.8495	0.0439	0.6403	PNPO
Q9H488	-0.1228	0.1194	0.0278	0.7119	POFUT1
Q7Z4H8	-0.0012	0.9951	-0.0774	0.5355	POGLUT3
O95602	0.1692	0.3149	0.0487	0.7981	POLR1A
P30876	0.0581	0.6445	-0.0508	0.4698	POLR2B
P52434	-0.1358	0.3616	0.0198	0.8474	POLR2H
P0CAP2	-0.0197	0.8235	-0.3079	0.0349	POLR2M
Q6EEV4	-0.1926	0.3099	0.0566	0.6373	POLR2M_1
Q15165	-0.0943	0.6612	0.1778	0.2447	PON2
Q99575	0.2207	0.5599	0.15	0.4621	POP1
P16435	-0.109	0.4812	-0.1069	0.3313	POR
A5A3E0	0.054	0.7134	0.2066	0.3746	POTEF
Q15181	-0.1019	0.4303	-0.0466	0.7101	PPA1
Q9H2U2	0.0304	0.8002	0.0545	0.6658	PPA2
Q9HAB8	-0.1529	0.2346	-0.0111	0.8746	PPCS
P62937	0.0002	0.9968	-0.017	0.7238	PPIA
P23284	-0.1079	0.2343	0.003	0.9674	PPIB
Q08752	0.2553	0.3463	-0.0588	0.6386	PPID
Q9Y3C6	-0.0554	0.6049	0.0636	0.6486	PPIL1
Q9H2H8	-0.0775	0.5171	-0.0925	0.4452	PPIL3
O60437	-0.1247	0.7389	-0.1171	0.7176	PPL
P35813	0.0058	0.9663	-0.0247	0.8518	PPM1A
P49593	-0.0217	0.8596	-0.0132	0.9189	PPM1F
O15355	0.0327	0.6127	-0.0238	0.8144	PPM1G
Q9Y570	0.2507	0.1404	0.2306	0.2974	PPME1
P62136	0.0153	0.9282	-0.0766	0.6087	PPP1CA
P62136;P62140	-0.0971	0.395	0.0046	0.9687	PPP1CA;PPP1CB
P36873;P62136;P62140	0.1234	0.1674	0.067	0.4223	PPP1CA;PPP1CB;PPP1CC
P36873;P62136	0.0187	0.8449	0.1061	0.3512	PPP1CA;PPP1CC
P62140	0.0168	0.8104	0.0015	0.9802	PPP1CB
P36873	0.087	0.3555	0.0037	0.973	PPP1CC
O14974	0.078	0.6014	0.1859	0.1442	PPP1R12A
Q9BZL4	-0.0707	0.62	0.0586	0.6636	PPP1R12C
P41236;Q6NXS1	0.0642	0.4347	0.0627	0.2382	PPP1R2
Q15435	-0.0718	0.467	0.0562	0.465	PPP1R7
P62714;P67775	-0.0906	0.0988	-0.0646	0.1647	PPP2CA;PPP2CB
P60510;P62714;P67775	0.1253	0.4122	-0.0328	0.8108	PPP2CA;PPP2CB;PPP4C
P30153	0.0148	0.76	0.0695	0.0717	PPP2R1A
P63151	-0.0903	0.2699	-0.2148	0.1386	PPP2R2A
Q14738	-0.0374	0.7526	-0.015	0.8967	PPP2R5D
Q16537	-0.1337	0.4527	0.0065	0.9495	PPP2R5E
Q08209	-0.1105	0.3193	-0.0328	0.8237	PPP3CA
P16298;P48454;Q08209	0.062	0.6526	-0.068	0.664	PPP3CA;PPP3CB;PPP3CC
P63098	-0.0003	0.9979	0.006	0.9643	PPP3R1
P53041	-0.0657	0.5285	-0.0895	0.3864	PPP5C
O00743	-0.119	0.607	0.1309	0.3277	PPP6C
Q96BP3	-0.0605	0.7261	-0.0126	0.9379	PPWD1

O60831	-0.1609	0.0982	0.0438	0.4654	PRAF2
P42785	0.5668	0.0032	0.1659	0.6037	PRCP
Q06830	0.0062	0.8929	0.0266	0.5579	PRDX1
Q06830;Q13162	-0.009	0.926	0.1203	0.3481	PRDX1;PRDX4
P32119	0.0494	0.4723	0.0982	0.3731	PRDX2
P30048	-0.0995	0.1481	-0.1813	0.1588	PRDX3
Q13162	-0.2717	0.0272	-0.1132	0.1075	PRDX4
P30044	-0.165	0.2257	-0.0658	0.3233	PRDX5
P30041	0.1864	0.3215	0.1196	0.5057	PRDX6
Q9HCU5	0.007	0.9643	-0.1087	0.5983	PREB
P48147	0.0979	0.1582	0.029	0.7801	PREP
Q13131	0.1472	0.1204	0.1838	0.0512	PRKAA1
Q9Y478	0.172	0.1886	-0.0569	0.6219	PRKAB1
O43741;Q9Y478	0.0494	0.8525	0.0359	0.8765	PRKAB1;PRKAB2
P17612;P22694	-0.437	0.1833	-0.224	0.4048	PRKACA;PRKACB
P54619	0.0469	0.6875	0.0951	0.3875	PRKAG1
P10644	0.0985	0.163	0.0222	0.8518	PRKAR1A
P13861	0.0426	0.4619	-0.0176	0.8078	PRKAR2A
P05129;P05771;P17252	0.1028	0.5923	0.1332	0.3024	PRKCA
P14314	-0.1197	0.0488	-0.0686	0.2129	PRKCSH
P78527	-0.0212	0.7725	-0.0317	0.532	PRKDC
Q13976	0.1322	0.3279	-0.0757	0.5945	PRKG1
O75569	-0.1023	0.28	-0.1598	0.0696	PRKRA
Q99873	0.0916	0.3303	0.0293	0.6423	PRMT1
O14744	0.0864	0.2901	0.0022	0.9786	PRMT5
P04156	0.0171	0.9126	0.0671	0.5679	PRNP
Q9UMS4	0.006	0.9259	-0.0096	0.886	PRPF19
O94906	-0.0541	0.5821	-0.1108	0.2224	PRPF6
Q6P2Q9	-0.0027	0.9459	-0.0207	0.7398	PRPF8
P60891	0.5353	0.1204	0.4655	0.2509	PRPS1
P11908;P21108;P60891	0.3797	0.301	0.0948	0.6986	PRPS1;PRPS2
P11908;P21108	-0.1162	0.3406	0.0033	0.9813	PRPS2
Q14558	0.1844	0.2708	0.0744	0.5724	PRPSAP1
O60256	-0.0307	0.9079	-0.3947	0.0506	PRPSAP2
Q96M27	-0.1617	0.233	0.0266	0.6734	PRRC1
Q9Y520	0.0569	0.335	-0.1687	0.0768	PRRC2C
P07602	-0.1727	0.2514	0.0371	0.698	PSAP
Q9Y617	0.2238	0.7346	0.4274	0.4988	PSAT1
O75475	0.0601	0.7014	-0.1403	0.2972	PSIP1
P25786	0.0082	0.8991	-0.027	0.4943	PSMA1
P25787	0.0207	0.7817	0.0131	0.8521	PSMA2
P25788	-0.0019	0.9683	0.0227	0.6798	PSMA3
P25789	0.0135	0.8213	-0.0179	0.7564	PSMA4
P28066	0.0477	0.4757	-0.0409	0.5307	PSMA5
P60900	0.0353	0.3604	0.0235	0.6426	PSMA6
O14818	0.0366	0.321	-0.0105	0.7707	PSMA7
P20618	-0.0318	0.6061	-0.0217	0.5522	PSMB1
P40306	-0.0505	0.6556	-0.4546	0.0666	PSMB10

P49721	-0.033	0.5553	0.0087	0.8627	PSMB2
P49720	-0.1554	0.1174	-0.0446	0.6627	PSMB3
P28070	0.0229	0.7069	0.0527	0.4373	PSMB4
P28074	0.003	0.9631	0.133	0.0998	PSMB5
P28072	0.0709	0.3147	0.1047	0.2071	PSMB6
Q99436	-0.0394	0.6887	0.0272	0.7027	PSMB7
P28062	-0.03	0.7822	-0.1719	0.146	PSMB8
P62191	0.0635	0.2311	0.0027	0.9581	PSMC1
P35998	0.0219	0.6653	0.0546	0.2213	PSMC2
P17980	0.0238	0.4935	0.0249	0.6297	PSMC3
P43686	0.0872	0.2223	0.0382	0.2887	PSMC4
P62195	0.0396	0.136	0.0912	0.0623	PSMC5
P62333	0.0761	0.133	0.0701	0.1451	PSMC6
Q99460	0.0646	0.1765	-0.0122	0.7115	PSMD1
O75832	-0.1019	0.4962	0.0562	0.635	PSMD10
O00231	0.0355	0.3932	0.0624	0.3383	PSMD11
O00232	0.0686	0.2316	0.0108	0.783	PSMD12
Q9UNM6	0.0666	0.1271	0.0625	0.2443	PSMD13
O00487	0.0526	0.2119	0.0234	0.6972	PSMD14
Q13200	0.0406	0.2316	0.0069	0.9074	PSMD2
O43242	-0.0144	0.7699	0.0481	0.3169	PSMD3
P55036;A2A3N6	-0.0368	0.5248	0.0132	0.7901	PSMD4
Q16401	0.133	0.0114	0.1084	0.1391	PSMD5
Q15008	0.0115	0.899	0.0212	0.7461	PSMD6
P51665	0.1414	0.1329	0.0764	0.4266	PSMD7
P48556	0.0124	0.8535	0.0996	0.1335	PSMD8
O00233	0.0477	0.3486	0.08	0.2492	PSMD9
Q06323	-0.053	0.5354	-0.144	0.2415	PSME1
Q9UL46	-0.0042	0.9663	-0.1694	0.2366	PSME2
P61289	0.1622	0.0903	0.112	0.0773	PSME3
O95456	-0.1875	0.1977	0.153	0.4013	PSMG1
Q8WXF1	0.0628	0.5089	-0.0303	0.7341	PSPC1
P26599	0.101	0.1491	-0.0975	0.0761	PTBP1
P48651	-0.0043	0.9832	0.0626	0.7292	PTDSS1
O14684	-0.2904	0.5128	-0.4303	0.4016	PTGES
Q9H7Z7	0.0603	0.5695	-0.0583	0.6701	PTGES2
Q15185	-0.0623	0.5259	0.0514	0.5692	PTGES3
Q16647	-0.0492	0.849	0.0016	0.993	PTGIS
Q14914	0.1678	0.26	0.0917	0.5688	PTGR1
Q05397	-0.0125	0.9505	-0.0272	0.8985	PTK2
Q13308	0.0136	0.9494	-0.0904	0.6168	PTK7
P06454	-0.0118	0.9351	-0.1815	0.3902	PTMA
P20962	0.0769	0.5175	0.0177	0.88	PTMS
Q15257	0.0967	0.3204	-0.0246	0.8859	PTPA
Q06124	0.0559	0.458	0.0212	0.8123	PTPN11
Q9H3S7	0.0427	0.6667	-0.1147	0.4347	PTPN23
Q9Y3E5	-0.3822	0.1134	-0.0537	0.7881	PTRH2
Q6GMV3	-0.1201	0.2373	-0.1277	0.3793	PTRHD1

Q08623	0.239	0.4235	0.0214	0.9296	PUDP
Q9UHX1	-0.0127	0.827	-0.1051	0.0507	PUF60
Q14671	0.0893	0.607	0.0158	0.8884	PUM1
Q00577	-0.0968	0.4144	0.1299	0.137	PURA
Q7Z7A4	-0.3401	0.1705	-0.08	0.6308	PXK
P49023	0.2172	0.2131	0.0147	0.8536	PXN
Q9ULZ3	-0.0063	0.9784	-0.0651	0.7825	PYCARD
P11216	-0.0602	0.3772	0.0806	0.4207	PYGB
P06737;P11216;P11217	0.0928	0.3138	0.1636	0.1244	PYGB;PYGL;PYGM
P11216;P11217	-0.2553	0.2092	-0.091	0.5232	PYGB;PYGM
P06737	0.3489	0.0307	0.1802	0.2834	PYGL
Q9BRP8	-0.1062	0.2511	-0.1831	0.1441	PYM1
P47897	-0.1029	0.1887	-0.0611	0.2866	QARS1
P09417	-0.1719	0.0197	-0.0741	0.4036	QDPR
Q96PU8	0.0102	0.918	-0.0648	0.3239	QKI
Q5T6V5	-0.1274	0.205	0.1527	0.234	QNG1
P61026	-0.1372	0.1495	0.0038	0.9664	RAB10
P51153;P59190;P61006;P61026;P62820;Q92930;Q9H0U4;Q92928	0.1386	0.1486	-0.0696	0.5639	RAB10;RAB13;RAB15;RAB1A;RAB1B;RAB1C;RAB8A;
P62491;Q15907	-0.0013	0.9833	-0.0402	0.5492	RAB11B
Q9BXF6	-0.0599	0.7205	-0.2121	0.124	RAB11FIP5
Q6IQ22	-0.0198	0.858	-0.0026	0.9664	RAB12
P51153	0.0182	0.8606	-0.0731	0.5669	RAB13
O95716;P20336;P20337;P51153;P61006;Q92930;Q96E17	-0.227	0.0431	-0.0703	0.4741	RAB13;RAB3A;RAB3B;RAB8A;RAB8B
P61106	-0.1041	0.169	-0.1391	0.0974	RAB14
Q9NP72	0.0303	0.7228	-0.0557	0.5316	RAB18
P62820	-0.1177	0.4799	-0.0052	0.9604	RAB1A
P62820;Q9H0U4	-0.1683	0.2165	-0.2028	0.1465	RAB1A;RAB1B
P62820;Q9H0U4;Q92928	-0.043	0.6844	-0.074	0.1338	RAB1A;RAB1B;RAB1C
Q9H0U4	-0.0371	0.8401	-0.0824	0.6571	RAB1B
Q9H0U4;Q92928	-0.1481	0.5478	0.0083	0.9687	RAB1B;RAB1C
Q92928	-0.3186	0.1351	-0.0575	0.7668	RAB1C
Q9UL25	0.0287	0.8282	0.0122	0.9252	RAB21
Q9UL26	-0.2023	0.2575	0.0327	0.7356	RAB22A
Q13636;Q9UL26	0.2365	0.0897	0.4045	0.015	RAB22A;RAB31
Q9ULC3	0.018	0.7835	-0.0322	0.6959	RAB23
O14966;P57729;Q13637	-0.1616	0.2305	-0.0346	0.8387	RAB29;RAB32
P61019;Q8WUD1	-0.1057	0.1225	0.0195	0.7729	RAB2A
Q13637	0.0163	0.8876	-0.0735	0.6799	RAB32
Q9BZG1	0.0679	0.3901	-0.0632	0.477	RAB34
Q15286	-0.0175	0.8279	-0.1161	0.0347	RAB35
P20336	-0.1074	0.651	0.3276	0.3347	RAB3A
P20337	0.1733	0.2142	0.4094	0.1747	RAB3B
Q15042	-0.1699	0.157	0.0998	0.381	RAB3GAP1
Q9H2M9	0.0619	0.5869	-0.073	0.6746	RAB3GAP2
P20338	0.0745	0.6591	0.2258	0.0002	RAB4A
P20339	0.1473	0.3395	-0.0307	0.7336	RAB5A

P20339;P51148;P61020	-0.0487	0.4305	-0.0992	0.2051	RAB5A;RAB5B;RAB5C
P61020	-0.0752	0.39	-0.08	0.3292	RAB5B
P51148	0.0474	0.5084	-0.0261	0.5929	RAB5C
P20340	0.028	0.6286	0.0213	0.7761	RAB6A
Q9H0N0;Q53S08	0.1801	0.4108	0.1889	0.4711	RAB6C;RAB6D
P51149	-0.1602	0.1325	-0.1161	0.3061	RAB7A
P61006	0.0671	0.6864	0.0025	0.9914	RAB8A
Q92930	0.1608	0.3121	0.1507	0.4458	RAB8B
P51151;Q9NP90	0.0068	0.9487	-0.1777	0.213	RAB9A
Q15276	-0.054	0.3011	0.0922	0.4667	RABEP1
Q3YEC7	0.0248	0.8198	0.0299	0.8245	RABL6
P15153;P60763;P63000	-0.0129	0.7161	-0.031	0.6914	RAC1
P63244	0.0004	0.9904	0.0229	0.6126	RACK1
P54725	-0.1122	0.3548	-0.039	0.7757	RAD23A
P54725;P54727	0.036	0.758	-0.0787	0.3587	RAD23A;RAD23B
P54727	0.0976	0.1169	0.0026	0.9779	RAD23B
Q92878	0.1553	0.1552	0.066	0.373	RAD50
P78406	0.0477	0.514	0.1065	0.2318	RAE1
Q9P0K7	0.1662	0.1703	0.0676	0.4637	RAI14
P11233	-0.0041	0.9652	-0.1063	0.292	RALA
P11233;P11234	-0.1296	0.4492	-0.1549	0.4267	RALA;RALB
P11234	-0.1468	0.3405	-0.0052	0.9607	RALB
Q9UKM9	-0.0359	0.7267	-0.0676	0.406	RALY
P62826	-0.0445	0.1758	0.0072	0.8553	RAN
P43487	0.0361	0.5586	0.126	0.2109	RANBP1
P49792;Q99666;A6NKT7;O14715;P0DJD0;P0DJD1;Q7Z3J3	-0.125	0.1091	0.0597	0.4805	RANBP2
O60518	-0.1101	0.4004	0.0948	0.3234	RANBP6
P46060	0.159	0.0262	0.0129	0.8796	RANGAP1
P62834	-0.0366	0.8293	-0.0719	0.6543	RAP1A
P61224;P62834	-0.09	0.1317	-0.0127	0.8649	RAP1A;RAP1B
P61224;A6NIZ1	-0.1046	0.468	0.0477	0.6487	RAP1B
P52306	0.0844	0.3119	0.1103	0.3815	RAP1GDS1
P10114;P61225;Q9Y3L5	-0.1808	0.2779	-0.1423	0.4024	RAP2B
Q70E73	-0.2367	0.0657	-0.0122	0.9249	RAPH1
P54136	0.0344	0.4941	0.006	0.9161	RARS1
P20936	0.0709	0.54	0.0742	0.5683	RASA1
Q09028	-0.0617	0.6471	-0.0789	0.4275	RBBP4
Q09028;Q16576	0.1744	0.2972	-0.0212	0.8604	RBBP4;RBBP7
O43251;Q9NWB1	0.1277	0.5132	0.1041	0.5598	RBFOX2
Q9NTZ6	-0.0349	0.6469	-0.0124	0.9131	RBM12
Q96PK6	0.2198	0.1657	-0.0583	0.369	RBM14
Q96I25	-0.2894	0.1441	-0.1025	0.4875	RBM17
Q5T481	-0.193	0.3062	-0.0744	0.7277	RBM20
P49756	0.2622	0.0785	0.0394	0.7707	RBM25
P98179	0.1726	0.2316	0.0928	0.4303	RBM3
Q14498	0.1194	0.2275	-0.1217	0.1236	RBM39
Q9BQ04;Q9BWF3	0.1301	0.4607	-0.1203	0.1175	RBM4;RBM4B

Q9Y5S9	-0.145	0.2073	0.0658	0.2724	RBM8A
P29558	-0.1371	0.2829	0.1255	0.2063	RBMS1
P38159	0.1298	0.2665	-0.0018	0.9761	RBMX
P18754	0.1355	0.5018	0.1681	0.0387	RCC1
Q9P258	-0.083	0.7303	-0.0657	0.5534	RCC2
Q15293	-0.0854	0.4472	-0.1722	0.0326	RCN1
Q14257	0.0368	0.6878	-0.1916	0.2829	RCN2
Q96D15	-0.1667	0.2924	-0.1478	0.4001	RCN3
Q8IZV5	0.3037	0.439	0.2766	0.5631	RDH10
Q8TC12	-0.0875	0.4523	-0.0344	0.689	RDH11
Q9HBH5	0.1058	0.3425	0.0114	0.9342	RDH14
P35241	0.0277	0.6103	0.0375	0.4899	RDX
O95980	0.0056	0.9735	0.1705	0.2957	RECK
P46063	-0.0054	0.9572	0.107	0.3066	RECQL
Q00765	0.0322	0.7114	-0.0611	0.5361	REEP5
Q04206	-0.102	0.3572	0.0609	0.6142	RELA
Q9Y3B8	0.0478	0.4207	0.0915	0.0711	REXO2
Q14699	-0.1233	0.6111	-0.171	0.1455	RFTN1
Q15382	0.0287	0.8109	0.0886	0.2866	RHEB
P61586	-0.1398	0.0358	-0.0024	0.9799	RHOA
P08134;P61586;P62745	-0.1079	0.1953	-0.0741	0.4344	RHOA;RHOC
P84095	-0.0554	0.4589	0.046	0.5196	RHOG
Q8IXI1	0.2769	0.1074	-0.0516	0.624	RHOT2
Q9NPQ8	0.1574	0.551	-0.1345	0.3839	RIC8A
P52758	-0.0214	0.9249	-0.2632	0.494	RIDA
Q96DB5	0.0596	0.5188	0.0495	0.6521	RMDN1
Q96TC7	0.0049	0.9761	0.0371	0.7681	RMDN3
Q8TDP1	0.1144	0.7177	0.1177	0.6088	RNASEH2C
P61587	0.0622	0.7418	0.0238	0.8851	RND3
O43567	-0.2147	0.3139	-0.2226	0.1368	RNF13
P13489	-0.102	0.251	-0.0063	0.9422	RNH1
O43148	0.0017	0.9942	0.15	0.1643	RNMT
Q9H4A4	0.0991	0.2326	0.0237	0.6815	RNPEP
Q15287	0.0633	0.4411	-0.0941	0.2401	RNPS1
P10155	-0.004	0.9703	-0.0427	0.4997	RO60
Q13464	-0.0971	0.1206	-0.1385	0.2218	ROCK1
O75116;Q13464	0.0074	0.937	0.1263	0.1291	ROCK1;ROCK2
O75116	0.1029	0.2113	0.0487	0.3533	ROCK2
O75695	0.2377	0.0557	-0.1253	0.236	RP2
P27694	0.0502	0.7519	-0.2021	0.1634	RPA1
P35244	-0.2754	0.0471	0.1034	0.5271	RPA3
Q96AT9	0.0552	0.4864	0.0096	0.9225	RPE
P27635;Q96L21	-0.0927	0.3152	-0.0837	0.1691	RPL10
P62906	0.1109	0.0342	0.0284	0.663	RPL10A
P62913	-0.0539	0.3992	0.0133	0.8586	RPL11
P30050	-0.0053	0.9526	-0.021	0.5371	RPL12
P26373	0.0337	0.2108	0.0466	0.1156	RPL13
P40429;Q6NVV1	0.0135	0.8667	0.0222	0.5447	RPL13A

P50914	0.0117	0.8124	-0.0529	0.2873	RPL14
P61313	-0.029	0.6862	0.0817	0.2179	RPL15
P18621	0.0658	0.2728	0.0639	0.1482	RPL17
Q07020	0.0505	0.3302	0.0317	0.4081	RPL18
Q02543	0.0248	0.6017	0.036	0.5174	RPL18A
P84098	-0.1319	0.1281	0.0789	0.1988	RPL19
P46778	0.1277	0.0493	0.0103	0.8501	RPL21
P35268	0.1185	0.3998	-0.0509	0.4488	RPL22
P62829	0.0986	0.3806	-0.0895	0.3709	RPL23
P62750	0.05	0.1324	-0.0209	0.6765	RPL23A
P83731	0.0678	0.3559	0.0131	0.7113	RPL24
P61254;Q9UNX3	0.0785	0.2427	-0.031	0.5852	RPL26
P61353	-0.0099	0.859	0.0188	0.5981	RPL27
P46776	0.0536	0.2111	0.0298	0.4711	RPL27A
P46779	0.0466	0.729	0.038	0.5126	RPL28
P47914	0.0733	0.7464	-0.0057	0.9537	RPL29
P39023	0.0303	0.6551	-0.0372	0.5101	RPL3
P62888	0.0361	0.4664	0.0774	0.2702	RPL30
P62899	-0.1295	0.3647	0.0067	0.9243	RPL31
P62910	0.0458	0.5308	-0.021	0.7419	RPL32
P49207	-0.0957	0.4479	-0.0788	0.4182	RPL34
P42766	-0.0372	0.4353	-0.0322	0.6928	RPL35
P18077	-0.0858	0.3455	0.0101	0.9133	RPL35A
Q9Y3U8	0.0736	0.1583	0.0156	0.8616	RPL36
P61513	0.0197	0.6791	0.0093	0.8971	RPL37A
P63173	0.0991	0.3424	0.1071	0.4719	RPL38
P36578	-0.0054	0.8788	0.0111	0.8203	RPL4
P46777	0.0742	0.0831	-0.0469	0.4652	RPL5
Q02878	-0.0013	0.9784	-0.0396	0.3353	RPL6
P18124	0.0525	0.1185	0.0117	0.7285	RPL7
P62424	0.0205	0.7368	0.0294	0.5796	RPL7A
P62917	-0.045	0.5583	-0.0741	0.357	RPL8
P32969	-0.0602	0.177	-0.0521	0.5874	RPL9
P05388	0.0531	0.2476	-0.0232	0.6635	RPLP0
P05386	0.0743	0.4635	0.0525	0.3625	RPLP1
P05387	0.0294	0.6406	-0.0221	0.7066	RPLP2
P04843	-0.0402	0.5249	-0.1135	0.0127	RPN1
P04844	-0.088	0.1543	-0.0922	0.0337	RPN2
P78346	0.0923	0.4897	-0.0433	0.7574	RPP30
P46783;Q9NQ39	0.0808	0.287	0.0642	0.4895	RPS10
P62280	0.028	0.6568	-0.0176	0.745	RPS11
P25398	-0.0148	0.776	0.0191	0.8028	RPS12
P62277	-0.0768	0.1829	0.0388	0.4237	RPS13
P62263	0.024	0.6297	0.1108	0.0168	RPS14
P62841	-0.1583	0.3934	0.0733	0.3736	RPS15
P62244	-0.0403	0.4626	0.0144	0.8408	RPS15A
P62249	0.0004	0.994	0.0092	0.8455	RPS16
P08708	0.0592	0.331	-0.0009	0.9904	RPS17

P62269	-0.0185	0.7356	-0.0324	0.4913	RPS18
P39019	0.0409	0.3642	0.0208	0.5282	RPS19
P15880	0.0461	0.1529	0.0088	0.8489	RPS2
P60866	-0.1041	0.2355	0.1006	0.2759	RPS20
P63220	0.0085	0.87	-0.0287	0.7004	RPS21
P62266	-0.0422	0.2814	0.0125	0.8818	RPS23
P62847	0.1073	0.1072	-0.0065	0.9125	RPS24
P62851	0.0194	0.6612	0.0812	0.1917	RPS25
P62854;Q5JNZ5	0.0144	0.8717	0.0188	0.882	RPS26
P42677;Q71UM5	-0.0701	0.503	-0.0242	0.7268	RPS27;RPS27L
P0CG47;P0CG48;P62979;P62987	-0.0943	0.1305	0.0227	0.3573	RPS27A
Q71UM5	-0.0866	0.263	-0.0443	0.481	RPS27L
P62857	0.0201	0.7779	0.0285	0.7065	RPS28
P23396	0.041	0.2336	0.0075	0.8989	RPS3
P61247	-0.0023	0.9745	-0.0282	0.7311	RPS3A
P62701	0.038	0.6036	0.0185	0.7895	RPS4X
P62701;P22090;Q8TD47	0.0108	0.9149	0.1459	0.0646	RPS4X;RPS4Y1
P46782	0.1441	0.128	0.0269	0.8111	RPS5
P62753	-0.0282	0.5806	-0.0035	0.9438	RPS6
P51812	0.1251	0.1841	-0.1171	0.1105	RPS6KA3
P62081	0.0927	0.4121	-0.0728	0.3463	RPS7
P62241	0.0353	0.452	0.0133	0.7887	RPS8
P46781	-0.0407	0.2952	-0.0323	0.6327	RPS9
P08865;A0A8I5KQE6	-0.0064	0.8749	0.0215	0.6232	RPSA
Q5VZM2;Q7L523	-0.1427	0.1592	-0.0442	0.5804	RRAGA;RRAGB
Q9HB90;Q9NQL2	-0.0169	0.8083	-0.0497	0.601	RRAGC
P10301	0.0627	0.7106	0.0157	0.7909	RRAS
P10301;P62070	0.178	0.2755	-0.1912	0.1806	RRAS;RRAS2
P62070	0.2705	0.1452	0.0027	0.9905	RRAS2
Q9P2E9	-0.0055	0.9039	-0.0326	0.5543	RRBP1
O76021	0.1067	0.1887	-0.0767	0.4714	RSL1D1
Q15404	-0.1614	0.1801	0.0507	0.6913	RSU1
O00442	0.1535	0.3636	0.0451	0.7679	RTCA
Q9Y3I0	0.0069	0.9143	0.032	0.5023	RTCB
Q9NQC3	-0.0148	0.8979	0.0628	0.4883	RTN4
Q9Y224	0.1546	0.3238	0.3554	0.002	RTRAF
Q96T51	-0.1641	0.1159	-0.0286	0.7483	RUFY1
Q9Y265	0.0334	0.5043	0.0017	0.9517	RUVBL1
Q9Y230	0.0573	0.1155	0.024	0.3429	RUVBL2
Q15413	0.0806	0.7965	-0.2924	0.1455	RYR3
P60903	-0.1286	0.3098	-0.1289	0.4679	S100A10
P31949	0.1613	0.0452	0.1915	0.0261	S100A11
Q99584	-0.006	0.9579	0.0593	0.5942	S100A13
Q96FQ6	0.1667	0.2574	0.1435	0.2774	S100A16
P26447	-0.1123	0.6301	-0.2679	0.4028	S100A4
Q9NTJ5	0.0013	0.982	-0.0125	0.8074	SACM1L
Q9UBE0	0.1156	0.4524	0.0524	0.7966	SAE1
Q15424	-0.0117	0.8497	-0.0266	0.7611	SAFB

Q9Y3Z3	-0.1858	0.6422	-0.0024	0.9913	SAMHD1
Q9Y512	0.0773	0.347	-0.0651	0.5127	SAMM50
Q9NR31	-0.0551	0.2778	-0.0066	0.937	SAR1A
Q9NR31;Q9Y6B6	-0.0708	0.3789	-0.079	0.0896	SAR1A;SAR1B
P82979	0.0285	0.8889	0.0054	0.972	SARNP
P49591	0.0069	0.9551	0.1181	0.5025	SARS1
Q9NP81	-0.0616	0.9067	0.4924	0.5817	SARS2
Q15020	0.0632	0.6447	-0.0284	0.8733	SART3
Q9Y3A5	-0.0563	0.5114	-0.0452	0.6375	SBDS
O15126	0.0134	0.8719	-0.0546	0.5435	SCAMP1
O14828	-0.0255	0.6864	0.0021	0.9798	SCAMP3
Q969E2	-0.2405	0.031	0.0077	0.9438	SCAMP4
Q14108	0.012	0.8879	-0.0558	0.7151	SCARB2
Q8NBX0	-0.1047	0.461	0.0402	0.7727	SCCPDH
Q8WVM8	0.0085	0.9117	-0.0214	0.7432	SCFD1
P22307	-0.0279	0.7585	-0.107	0.2854	SCP2
Q9HB40	0.0948	0.6068	0.0348	0.8816	SCPEP1
Q14160	-0.1363	0.3573	0.0116	0.9152	SCRIB
Q12765	0.1864	0.191	0.1046	0.2726	SCRN1
Q96KG9	0.017	0.8513	0.0389	0.7255	SCYL1
O00560	-0.2303	0.1966	-0.4276	0.1091	SDCBP
Q9HCN8	-0.1831	0.0655	0.1036	0.2299	SDF2L1
P31040	0.0085	0.9328	0.0194	0.8841	SDHA
P21912	0.0586	0.5446	0.1018	0.3675	SDHB
P67812	0.0467	0.6555	0.0012	0.9911	SEC11A
P55735	0.0356	0.7516	0.0005	0.9949	SEC13
O15027	0.102	0.2493	-0.1477	0.0176	SEC16A
O75396	0.1352	0.2737	0.0167	0.8593	SEC22B
Q15436	0.0001	0.9984	-0.0263	0.58	SEC23A
Q15436;Q15437	-0.0416	0.5471	0.0757	0.5813	SEC23A;SEC23B
P53992	0.0992	0.2305	-0.0266	0.6888	SEC24C
O94855	-0.0852	0.4167	-0.0141	0.8679	SEC24D
O94979	-0.0306	0.5437	-0.021	0.7677	SEC31A
P61619	0.0275	0.5598	0.0886	0.2829	SEC61A1
P60468	0.0095	0.9251	0.0399	0.7506	SEC61B
P60059	0.207	0.1543	0.2292	0.1489	SEC61G
Q99442	0.0653	0.487	0.075	0.4516	SEC62
Q9UGP8	-0.0296	0.7008	0.0339	0.6462	SEC63
Q93073	0.1546	0.6126	0.0575	0.7047	SECISBP2L
Q13228	-0.3021	0.2571	-0.3025	0.3865	SELENBP1
P49903	-0.3339	0.0429	-0.182	0.1479	SEPHS1
Q9P0V9	-0.2831	0.2884	-0.028	0.9076	SEPTIN10
Q9NVA2	0.0865	0.2959	0.0384	0.5579	SEPTIN11
Q14141;Q9NVA2	0.0526	0.5526	0.0654	0.151	SEPTIN11;SEPTIN6
Q15019	0.0213	0.7228	0.0135	0.7907	SEPTIN2
Q15019;Q16181	-0.0484	0.7787	-0.369	0.2039	SEPTIN2;SEPTIN7
Q14141	0.306	0.3724	0.257	0.2439	SEPTIN6
Q16181	0.0119	0.7642	0.0241	0.5249	SEPTIN7

Q92599	-0.0152	0.939	-0.0427	0.7399	SEPTIN8
Q9UHD8	-0.0289	0.8292	-0.0905	0.5905	SEPTIN9
Q8NC51	0.0128	0.8179	0.0118	0.8458	SERBP1
Q9NRX5	-0.2379	0.1246	-0.1122	0.3693	SERINC1
P30740	0.0505	0.632	-0.1382	0.2852	SERPINB1
P30740;P50452;P50453;O75830	0.0576	0.7496	-0.2575	0.1294	SERPINB1;SERPINB8;SER
P35237	-0.0534	0.6874	-0.1574	0.1876	SERPINB6
P50453	-0.0301	0.8767	-0.2807	0.1252	SERPINB9
P07093	0.0105	0.9807	-0.1369	0.4777	SERPINE2
P50454	0.1485	0.5078	0.0224	0.9109	SERPINH1
P0DME0;Q01105	0.0655	0.6665	0.002	0.9793	SET
Q8WTS6	0.0809	0.5227	0.0232	0.8129	SETD7
Q15637	-0.0654	0.6931	-0.0763	0.4026	SF1
Q15459	0.0585	0.4727	-0.03	0.6648	SF3A1
Q15428	-0.1202	0.2943	-0.019	0.7763	SF3A2
Q12874	-0.0816	0.6653	0.0513	0.7622	SF3A3
O75533	0.0882	0.2941	-0.0249	0.7184	SF3B1
Q13435	-0.038	0.5841	-0.0811	0.3268	SF3B2
Q15393	0.0339	0.6558	-0.0684	0.2741	SF3B3
Q15427	0.0462	0.7877	-0.3733	0.0158	SF3B4
P23246	0.0485	0.6218	-0.0821	0.1521	SFPQ
Q9H9B4	-0.059	0.4888	-0.0823	0.475	SFXN1
Q9BWM7;Q9H9B4	-0.304	0.1241	0.1832	0.5338	SFXN1;SFXN3
Q9BWM7	-0.1939	0.1201	-0.1179	0.4917	SFXN3
O43556	0.0289	0.9063	0.0277	0.8483	SGCE
P51688	0.2544	0.1437	0.0678	0.5875	SGSH
O43765	-0.0809	0.3603	0.2268	0.0234	SGTA
O75368	-0.0725	0.2333	-0.0277	0.712	SH3BGRL
Q9H299	-0.0837	0.5054	0.0387	0.742	SH3BGRL3
Q99961;Q99962	-0.178	0.021	-0.0737	0.4207	SH3GL1
Q9Y371	-0.2549	0.1558	-0.0604	0.6735	SH3GLB1
Q9NR46	0.0094	0.9535	0.0358	0.6566	SH3GLB2
Q96B97	-0.2579	0.0565	-0.0084	0.9569	SH3KBP1
Q5TCZ1	-0.0293	0.7925	-0.0427	0.8284	SH3PXD2A
A1X283	-0.2194	0.1737	-0.1727	0.0781	SH3PXD2B
P34896;P34897	0.1102	0.5488	0.1089	0.4935	SHMT1;SHMT2
P34897	-0.0123	0.9281	0.0211	0.8421	SHMT2
Q9UQ13	0.0769	0.7279	-0.3993	0.0504	SHOC2
P78324;Q5TFQ8	0.0515	0.7993	-0.0731	0.7463	SIRPA
Q15477	-0.0015	0.9951	0.0298	0.7961	SKIC2
Q6PGP7	0.0441	0.7652	-0.0292	0.7916	SKIC3
Q9GZS3	-0.1032	0.2755	0.0641	0.7456	SKIC8
P63208	0.0543	0.5378	-0.0925	0.2307	SKP1
Q9UP95	0.0895	0.4396	-0.0222	0.8794	SLC12A4
P53985	-0.2392	0.4639	0.1591	0.3355	SLC16A1
Q15758	-0.0379	0.8641	-0.1238	0.7253	SLC1A5
P53007	-0.0099	0.887	0.0408	0.6997	SLC25A1
Q02978	-0.0783	0.4373	-0.0182	0.841	SLC25A11

O75746	0.1033	0.2812	0.0101	0.9468	SLC25A12
O75746;Q9UJS0	0.0205	0.7399	-0.0543	0.6577	SLC25A12;SLC25A13
Q9H936	-0.1847	0.2047	-0.1332	0.4607	SLC25A22
Q6NUK1	-0.0536	0.4445	-0.0908	0.3102	SLC25A24
Q00325	-0.0222	0.4397	0.0132	0.8433	SLC25A3
P05141;P12235;P12236;Q9H0C2	0.2624	0.0157	0.0101	0.9163	SLC25A4;SLC25A5;SLC25
P12235;P12236	-0.0159	0.7939	0.0459	0.554	SLC25A4;SLC25A6
P05141	0.0894	0.2555	-0.075	0.3642	SLC25A5
P05141;P12235;P12236;Q9H0C2	0.1283	0.1178	0.06	0.4379	SLC25A5;SLC25A6
Q6PCB7	-0.0583	0.7903	-0.2761	0.163	SLC27A1
Q8NEW0	-0.0018	0.9872	-0.031	0.7514	SLC30A7
Q6PML9	-0.025	0.8872	0.0502	0.7047	SLC30A9
O00400	0.339	0.0522	0.3489	0.2941	SLC33A1
Q8TB61	-0.1408	0.2261	-0.0776	0.4891	SLC35B2
Q8N357	-0.2077	0.2218	0.071	0.7007	SLC35F6
P08195	0.538	0.1324	0.5739	0.33	SLC3A2
Q8WWI5	-0.0781	0.6973	-0.2208	0.2621	SLC44A1
Q8IWA5	0.1558	0.3998	0.1448	0.0574	SLC44A2
Q9GZT3	0.1074	0.394	0.0079	0.9663	SLIRP
Q9H2G2	-0.1471	0.1308	0.1074	0.1171	SLK
Q15797;Q99717	0.0069	0.9735	-0.2525	0.219	SMAD1;SMAD5
O15198;P84022;Q15796	-0.1794	0.2798	-0.129	0.6057	SMAD2;SMAD3
O60264;P28370	0.2514	0.0893	-0.1629	0.0165	SMARCA1;SMARCA5
Q8TAQ2;Q92922	0.0006	0.9961	-0.1603	0.2251	SMARCC2
Q969G3	0.0711	0.6483	0.149	0.2345	SMARCE1
Q14683	0.1892	0.32	-0.0305	0.7672	SMC1A
Q9UQE7	0.028	0.8522	-0.1819	0.3604	SMC3
A6NFE2	-0.3277	0.5127	-0.1776	0.3945	SMCO2
Q16637	0.3779	0.2968	0.2303	0.4458	SMN1
P17405	0.0821	0.752	-0.0787	0.7638	SMPD1
P52788	0.0176	0.8964	0.1047	0.3736	SMS
Q2TAY7	0.171	0.3819	-0.071	0.3499	SMU1
Q6GMV2	0.0865	0.6197	-0.0702	0.6169	SMYD5
O00161	0	0.9999	-0.1066	0.3005	SNAP23
O95295	-0.0498	0.6389	0.0705	0.2667	SNAPIN
Q7KZF4	-0.0014	0.9698	-0.072	0.3766	SND1
O75643	0.0187	0.8654	-0.0869	0.2384	SNRNP200
Q96DI7	-0.0159	0.8678	-0.076	0.2775	SNRNP40
P08621	0.0028	0.9712	-0.0721	0.4989	SNRNP70
P09012	-0.0281	0.7776	-0.0433	0.4248	SNRPA
P09661	-0.0663	0.5706	-0.1389	0.1232	SNRPA1
P14678;P63162	-0.0005	0.9976	-0.073	0.5095	SNRPB;SNRPN
P09234	-0.1425	0.1718	0.0736	0.503	SNRPC
P62314	-0.0085	0.9378	-0.1646	0.0898	SNRPD1
P62316	-0.0402	0.5603	0.0581	0.3637	SNRPD2
P62318	-0.063	0.4142	0.0171	0.7685	SNRPD3
P62304	0.1401	0.1295	-0.0332	0.7476	SNRPE
P62306	-0.1299	0.537	-0.1118	0.219	SNRPF

P62308;A8MWD9	-0.0399	0.7086	-0.0621	0.5009	SNRPG;SNRPGP15
Q13425	0.0669	0.4145	-0.0787	0.0689	SNTB2
P55769	-0.0118	0.8859	0.0367	0.6917	SNU13
Q13596	-0.0217	0.7504	-0.1006	0.0771	SNX1
O60749;Q13596	-0.0512	0.4371	-0.0269	0.6708	SNX1;SNX2
Q9UMY4	0.0905	0.2549	-0.2173	0.0069	SNX12
O60493;Q9UMY4	0.1707	0.2308	-0.0078	0.9298	SNX12;SNX3
Q15036	0.0141	0.8936	-0.022	0.8924	SNX17
Q96RF0	0.0452	0.7604	-0.0555	0.6517	SNX18
O60749	0.0167	0.8187	-0.079	0.1668	SNX2
Q96L92	-0.1394	0.2039	-0.0108	0.8864	SNX27
O60493	0.0134	0.8673	-0.0218	0.7565	SNX3
Q9Y5X3	-0.075	0.3684	-0.0874	0.4534	SNX5
Q9UNH7	-0.0581	0.5605	-0.0324	0.5173	SNX6
Q9Y5X1	-0.1063	0.4426	0.0244	0.8218	SNX9
P00441	-0.0385	0.4076	-0.0993	0.3476	SOD1
P04179	0.1947	0.412	-0.4325	0.1454	SOD2
P18583	-0.0916	0.5242	-0.2472	0.1255	SON
O60504	-0.1099	0.5171	0.2482	0.099	SORBS3
Q00796	0.1236	0.4447	-0.0219	0.9395	SORD
P23497;Q9H930	-0.0347	0.6426	-0.1958	0.1371	SP100
O60271	-0.0656	0.4745	-0.1338	0.2587	SPAG9
P09486	0.1578	0.6353	0.2393	0.6415	SPARC
Q8N0X7	-0.0599	0.7295	-0.1632	0.5172	SPART
Q9BXB7	0.1035	0.8559	0.0822	0.6334	SPATA16
Q9Y6A9	-0.1122	0.0348	-0.0739	0.2191	SPCS1
Q15005	0.0494	0.603	0.0149	0.8849	SPCS2
P61009	-0.0123	0.8506	0.1185	0.0143	SPCS3
Q9NZD8	-0.0333	0.7018	-0.0374	0.7028	SPG21
Q9H2V7	0.0369	0.8828	-0.3413	0.2391	SPNS1
P35270	-0.0035	0.9786	0.0149	0.9231	SPR
Q13813	-0.0943	0.3357	0.0158	0.894	SPTAN1
O15020;P11277;Q01082;Q9H254	-0.0901	0.4077	0.0017	0.9894	SPTBN1
O15269	-0.0524	0.7011	-0.0004	0.9977	SPTLC1
Q9Y6N5	-0.1878	0.5068	-0.1741	0.5667	SQOR
Q13501	-0.1629	0.4474	-0.1065	0.4753	SQSTM1
P12931	-0.0991	0.4016	0.1847	0.0946	SRC
P06241;P07947;P12931	0.0687	0.5114	0.0035	0.9633	SRC;YES1
O75044;Q7Z6B7	0.1324	0.3907	0.0607	0.5898	SRGAP1
P30626	0.0208	0.8903	0.0918	0.3931	SRI
P19623	-0.0412	0.5328	0.1327	0.0904	SRM
P37108	-0.1485	0.0414	-0.0159	0.8241	SRP14
P09132	-0.0497	0.4895	-0.0964	0.3878	SRP19
P61011	-0.0989	0.3694	-0.0535	0.7445	SRP54
Q9UHB9	0.0718	0.3564	-0.0652	0.3614	SRP68
O76094	-0.0022	0.9819	-0.0779	0.4397	SRP72
P49458	-0.0948	0.2702	-0.0288	0.7084	SRP9
P08240	0.0151	0.8405	0.0588	0.6107	SRPRA

Q9Y5M8	-0.0343	0.6412	-0.0437	0.4624	SRPRB
Q9GZT4	-0.1346	0.5109	0.1169	0.4096	SRR
Q9UQ35	0.1451	0.1774	0.0063	0.9138	SRRM2
Q9BXP5	-0.1965	0.3329	-0.0729	0.6982	SRRT
Q07955	0.0495	0.4829	-0.0248	0.6575	SRSF1
O75494	-0.0766	0.5891	-0.1206	0.1253	SRSF10
Q05519	0.1414	0.3546	-0.0185	0.8826	SRSF11
P84103	0.0704	0.4732	-0.0138	0.7188	SRSF3
P84103;Q16629	0.0311	0.8426	-0.0543	0.6628	SRSF3;SRSF7
Q08170	-0.0688	0.4749	-0.2466	0.1771	SRSF4
Q08170;Q13243;Q13247	-0.0579	0.4993	0.032	0.4068	SRSF4;SRSF5;SRSF6
Q08170;Q13247	0.0665	0.8617	0.0339	0.8701	SRSF4;SRSF6
Q13247	-0.1746	0.6943	-0.001	0.9976	SRSF6
Q16629	0.0075	0.9485	-0.0593	0.4031	SRSF7
Q13242	0.0004	0.9964	0.0411	0.3809	SRSF9
P05455	-0.0337	0.6149	-0.0476	0.389	SSB
Q04837	-0.0541	0.5589	-0.0417	0.7457	SSBP1
Q8TE77	0.0762	0.7176	-0.1345	0.4097	SSH3
P43307	-0.0546	0.5378	-0.1385	0.224	SSR1
Q9UNL2	-0.0593	0.6923	-0.0652	0.5815	SSR3
P51571	-0.0309	0.5263	-0.0022	0.9679	SSR4
Q08945	-0.1485	0.1904	-0.0514	0.585	SSRP1
P50502;Q8IZP2	-0.0632	0.5321	-0.059	0.2539	ST13
Q92783	0.1404	0.3419	0.1013	0.3392	STAM
O75886	-0.091	0.3346	-0.0071	0.9273	STAM2
P42224	0.203	0.3022	-0.0848	0.4518	STAT1
P52630	0.1189	0.0202	-0.0707	0.4814	STAT2
P40763	0.0288	0.7075	-0.0454	0.3874	STAT3
P51692	0.1073	0.3743	-0.1242	0.2437	STAT5B
P42226	-0.1307	0.3523	-0.0458	0.7012	STAT6
O95793	-0.0387	0.663	-0.0065	0.9363	STAU1
Q13586	-0.1295	0.3715	-0.1206	0.4782	STIM1
P31948	0.0047	0.8934	0.0379	0.3617	STIP1
Q9Y6E0	0.0775	0.5924	0.1996	0.2339	STK24
Q13043;Q13188	0.195	0.1575	0.0965	0.3846	STK3
Q15208	0.0327	0.7665	0.0193	0.8969	STK38
Q9UEW8	-0.1121	0.4984	0.4965	0.0149	STK39
P16949	0.0072	0.9615	0.0291	0.8576	STMN1
Q93045	-0.062	0.5853	-0.0027	0.9854	STMN2
P27105	-0.0714	0.6756	-0.36	0.2321	STOM
Q9UJZ1	-0.0225	0.814	-0.0416	0.671	STOML2
Q9Y3F4	0.0771	0.404	0.001	0.9908	STRAP
Q5VSL9	-0.052	0.7017	-0.1498	0.5532	STRIP1
Q13033	-0.0543	0.6534	0.0003	0.9958	STRN3
P46977	-0.0711	0.3446	-0.0685	0.171	STT3A
Q8TCJ2	0.0357	0.6119	-0.0433	0.5399	STT3B
Q86Y82	0.1332	0.1453	0.0482	0.5969	STX12
Q12846	0.0865	0.2924	-0.0023	0.9835	STX4

O15400	-0.0909	0.2328	-0.0086	0.903	STX7
Q9UNK0	0.1472	0.7186	0.0914	0.4536	STX8
P61764	-0.1179	0.214	0.0016	0.977	STXBP1
O00186	0.0227	0.7072	-0.0275	0.7694	STXBP3
Q5T5C0	0.1442	0.2299	0.2455	0.1439	STXBP5
P53999	-0.0043	0.9681	-0.0669	0.3952	SUB1
Q9P2R7	0.0365	0.6423	0.1057	0.4781	SUCLA2
P53597	-0.1157	0.3254	-0.0259	0.8906	SUCLG1
Q96I99	0.0121	0.8817	0.0133	0.9209	SUCLG2
Q9Y2Z0	0.0373	0.4272	-0.0501	0.5372	SUGT1
Q8NBK3	0.073	0.5902	0.0679	0.6723	SUMF1
Q8NBJ7	-0.0998	0.3629	-0.0683	0.3661	SUMF2
P61956	0.148	0.3991	-0.091	0.5552	SUMO2
P55854;P61956;Q6EEV6	-0.0807	0.3613	-0.0322	0.7598	SUMO2;SUMO3;SUMO4
O94901	0.1599	0.6021	-0.0669	0.6817	SUN1
Q9UH99	0.1071	0.4166	-0.0927	0.4059	SUN2
Q9Y5B9	0.0418	0.7098	-0.1124	0.2118	SUPT16H
Q7KZ85	-0.1309	0.3692	-0.2717	0.244	SUPT6H
O15260	-0.0643	0.354	-0.0374	0.3992	SURF4
O95425	-0.2031	0.4627	-0.0152	0.9468	SVIL
Q9UH65	-0.0282	0.6576	-0.0329	0.741	SWAP70
O60506	-0.0338	0.5502	-0.0344	0.462	SYNCRIP
Q6ZMZ3	-0.1005	0.6076	-0.079	0.6991	SYNE3
O43759	-0.2208	0.2332	-0.0535	0.7456	SYNGR1
P57105	-0.0525	0.6774	-0.093	0.5656	SYNJ2BP
Q16563	-0.0512	0.698	-0.0974	0.343	SYPL1
P21675;Q8IZX4	-0.2035	0.3175	-0.0932	0.6914	TAF1;TAF1L
Q92804	-0.0234	0.8643	-0.0508	0.4005	TAF15
Q01995	0.0381	0.9646	0.9477	0.3245	TAGLN
P37802	0.2798	0.0551	0.11	0.3965	TAGLN2
P37837	0.1809	0.0783	0.0864	0.3117	TALDO1
O15533	0.0925	0.2136	-0.1299	0.3847	TAPBP
Q13148	0.0372	0.6825	-0.0979	0.1838	TARDBP
P26639	0.0474	0.6828	0.1992	0.2721	TARS1
O14907	0.0332	0.7882	0.0491	0.645	TAX1BP3
Q8TC07	0.0352	0.7828	-0.0652	0.3726	TBC1D15
Q9UPU7	-0.0415	0.7745	-0.0354	0.833	TBC1D2B
Q66K14	-0.2004	0.1365	0.0201	0.884	TBC1D9B
O75347	-0.0352	0.5113	-0.0176	0.7551	TBCA
Q99426	-0.2075	0.1	0.0222	0.7708	TBCB
Q9BTW9	0.0729	0.5426	0.0485	0.6707	TBCD
Q5QJ74	0.2197	0.1053	-0.0896	0.5496	TBCEL
Q9UHD2	0.0545	0.733	0.0297	0.7873	TBK1
Q9BZK7	-0.0091	0.952	-0.1401	0.21	TBL1XR1
Q9Y4P3	-0.0096	0.9203	0.0931	0.2519	TBL2
P23193;Q15560	0.1091	0.604	0.1821	0.3758	TCEA1
Q6IPX3;Q5H9L2;Q969E4	0.1211	0.6166	-0.1286	0.2375	TCEAL3;TCEAL5;TCEAL6
O14776	-0.0594	0.7479	0.0564	0.5817	TCERG1

Q13488	0.2433	0.0484	0.0878	0.2768	TCIRG1
P17987	-0.0112	0.7422	0.013	0.6758	TCP1
Q9NZ01	-0.0388	0.6478	-0.2591	0.018	TECR
Q9UGI8	0.5174	0.0435	0.1427	0.5375	TES
Q92734	-0.0954	0.2806	-0.0398	0.5145	TFG
P02786	0.0842	0.2402	-0.0088	0.9655	TFRC
P21980	-0.0063	0.9863	0.5125	0.342	TGM2
P07996	-0.2713	0.5449	-0.138	0.8566	THBS1
Q96FV9	0.0743	0.3983	0.2444	0.0006	THOC1
P52888	-0.0882	0.6407	0.0526	0.5376	THOP1
Q9Y2W1	0.0209	0.8875	-0.0498	0.6552	THRAP3
Q9NXG2	-0.0109	0.9534	-0.0238	0.8474	THUMPD1
P04216	0.1936	0.2579	0.1204	0.4033	THY1
P31483;Q01085	0.0768	0.5032	-0.2305	0.0011	TIAL1
Q9NQ88	-0.1323	0.5112	0.0099	0.9674	TIGAR
Q9Y5L4	0.0393	0.7703	-0.0665	0.5051	TIMM13
Q3ZCQ8	0.0662	0.5414	0.0375	0.6811	TIMM50
P01033	-0.2484	0.2361	0.0971	0.5412	TIMP1
O75663	-0.0876	0.4476	-0.0332	0.6824	TIPRL
Q07157	0.1008	0.314	0.1058	0.545	TJP1
O00142	-0.1569	0.1346	0.0205	0.8847	TK2
P29401	0.1343	0.3258	-0.0131	0.9276	TKT
Q9Y490	0.0825	0.3197	0.0864	0.4806	TLN1
Q9Y490;Q9Y4G6	0.113	0.1808	0.0827	0.4887	TLN1;TLN2
Q9Y4G6	0.0593	0.3809	0.0461	0.5564	TLN2
Q99805	-0.1811	0.0661	-0.0983	0.2208	TM9SF2
Q9HD45	-0.0135	0.8257	0.0369	0.5909	TM9SF3
Q969X1	-0.3769	0.0199	-0.2056	0.1214	TMBIM1
Q13445	-0.1726	0.1908	-0.1499	0.252	TMED1
P49755	-0.1326	0.0953	-0.0709	0.1281	TMED10
Q15363	-0.1004	0.4597	-0.1784	0.1047	TMED2
Q9Y3Q3	-0.0281	0.8994	0.1648	0.4789	TMED3
Q7Z7H5	-0.0838	0.4168	-0.1148	0.2769	TMED4
Q7Z7H5;Q9BVK6	-0.139	0.5967	0.0267	0.8282	TMED4;TMED9
Q9Y3B3	-0.1159	0.2578	-0.0393	0.6983	TMED7
Q9BVK6	-0.1045	0.4784	-0.082	0.369	TMED9
Q9NUM4	-0.1921	0.1145	-0.2228	0.1137	TMEM106B
Q9BVC6	-0.0652	0.5448	-0.0195	0.8634	TMEM109
P17152	-0.3122	0.0508	-0.2974	0.0656	TMEM11
Q4V9L6	-0.1769	0.188	-0.0626	0.731	TMEM119
Q8N131	-0.0451	0.6756	-0.0591	0.6187	TMEM123
Q9H061	-0.0111	0.9185	0.0907	0.4916	TMEM126A
Q7Z7N9	0.0146	0.935	-0.3607	0.1057	TMEM179B
Q8IY95	0.0661	0.5039	-0.2453	0.0784	TMEM192
Q6UW68	0.0573	0.6582	-0.1646	0.2537	TMEM205
Q8WUH6	-0.0087	0.9456	0.0706	0.399	TMEM263
P57088	-0.136	0.238	-0.0412	0.6571	TMEM33
Q9BTV4	0.0211	0.804	0.0924	0.3729	TMEM43

O94886	-0.1839	0.4675	-0.2197	0.3701	TMEM63A
Q9NYL9	0.0169	0.7745	0.004	0.9625	TMOD3
P42166;P42167	-0.0735	0.627	0.0727	0.6333	TMPO
P42167	-0.1715	0.2999	0.0765	0.5785	TMPO_1
Q9H8H3	-0.1655	0.4199	-0.2282	0.4018	TMT1A
Q6ZXV5	0.037	0.6547	-0.2018	0.1386	TMTC3
Q9H3N1	0.0989	0.1963	-0.0531	0.5483	TMX1
Q96JJ7	0.0653	0.4725	-0.0807	0.4431	TMX3
Q9C0C2	0.0193	0.8271	0.0306	0.5926	TNKS1BP1
Q92973	0.0886	0.1181	0.0126	0.8598	TNPO1
Q9HBL0	-0.0053	0.9548	0.114	0.5387	TNS1
Q63HR2;Q68CZ2;Q9HBL0	0.0639	0.7326	0.2024	0.3719	TNS1;TNS2;TNS3
Q68CZ2	0.3731	0.3828	0.3181	0.5709	TNS3
Q9H0E2	-0.0414	0.5372	-0.0741	0.2316	TOLLIP
O60784	0.2402	0.0901	0.0103	0.9132	TOM1
Q9NS69	0.0791	0.64	0.2478	0.3371	TOMM22
O96008	-0.0009	0.9928	-0.0197	0.8258	TOMM40
O94826	-0.0066	0.9581	0.0975	0.2411	TOMM70
P11387	0.1156	0.365	-0.025	0.8774	TOP1
Q02880	0.0556	0.6143	-0.0847	0.2479	TOP2B
O14656	-0.0446	0.7944	0.1218	0.3252	TOR1A
Q5JTV8	0.0418	0.6347	0.0223	0.7614	TOR1AIP1
Q8NFQ8	-0.0175	0.8443	-0.0055	0.9598	TOR1AIP2
Q12888	-0.0912	0.561	-0.2064	0.0238	TP53BP1
O14683	0.0572	0.8279	-0.0918	0.4124	TP53I11
Q53FA7	0.0758	0.6191	0.1245	0.4373	TP53I3
O43399	-0.0407	0.6073	0.0629	0.1601	TPD52L2
P60174	0.0633	0.2637	0.0537	0.5511	TPI1
P09493	0.2772	0.6463	0.3867	0.6007	TPM1
P06753;P07951;P09493;P67936	0.1259	0.4329	0.1225	0.6582	TPM1;TPM2;TPM3;TPM4
P07951	0.1238	0.4396	0.1778	0.52	TPM2
P06753;P07951;P67936	0.0788	0.5117	0.0765	0.6506	TPM2;TPM3;TPM4
P07951;P67936	0.1163	0.4425	0.252	0.3935	TPM2;TPM4
P06753	0.1512	0.0753	0.0351	0.7762	TPM3
P67936	0.2279	0.3156	-0.0503	0.6826	TPM4
P29144	-0.0101	0.7502	0.0767	0.1331	TPP2
P12270	-0.0689	0.6073	-0.0394	0.568	TPR
P13693	-0.0391	0.7524	-0.1237	0.3682	TPT1
Q13595	0.0101	0.9368	0.04	0.6867	TRA2A
P62995	0.1097	0.4073	0.0395	0.6785	TRA2B
Q15628	-0.1875	0.1692	0.0716	0.5035	TRADD
Q15629	0.2028	0.1361	-0.3168	0.1701	TRAM1
Q12931	0.0037	0.973	0.1243	0.3093	TRAP1
O43617	0.203	0.0958	-0.1214	0.2911	TRAPPC3
Q9Y2L5	-0.148	0.4813	0.0796	0.5782	TRAPPC8
Q14258	0.0335	0.7607	-0.0061	0.9496	TRIM25
Q13263	-0.0221	0.8055	-0.0314	0.6684	TRIM28
Q9H2D6	-0.0032	0.9675	-0.02	0.8147	TRIOBP

Q15642	-0.0723	0.4975	-0.0605	0.5035	TRIP10
Q14669	-0.184	0.3893	-0.0479	0.7697	TRIP12
Q15654	0.1381	0.1469	-0.0264	0.8074	TRIP6
Q9UI30	-0.2058	0.0701	-0.0004	0.998	TRMT112
Q99816	0.0276	0.7673	0.1124	0.3232	TSG101
Q15631	0.0684	0.3753	-0.0324	0.7548	TSN
Q2NL82	0.2057	0.7502	-0.3264	0.1516	TSR1
Q16762	0.3843	0.107	0.0779	0.5622	TST
Q14166	0.0164	0.9036	-0.1437	0.2213	TTLL12
Q8WZ42	-0.1376	0.5192	-0.0935	0.4318	TTN
P0DPH7;P0DPH8;Q6PEY2;Q71U36;Q9BQE3;Q9NY65	0.0248	0.6216	0.0363	0.4867	TUBA1C
A6NHL2;P68363;P68366;Q71U36;Q9BQE3	0.0968	0.1898	0.1106	0.0795	TUBA1C;TUBA4A
P68363;P68366	0.0128	0.8667	0.0714	0.2346	TUBA4A
Q9H853	-0.0009	0.9921	0.0633	0.3367	TUBA4B
P07437	-0.0236	0.7536	-0.0117	0.7946	TUBB
A6NNZ2;P04350;P07437;P68371;Q13509;Q13885;Q3ZCM7;Q9BUF5;Q9BVA1;Q9H4B7	0.0005	0.9931	-0.0347	0.6006	TUBB;TUBB1;TUBB2A;TUBB2B;TUBB3;TUBB4B;TUBB6
A6NNZ2;P07437;P68371;Q13885;Q3ZCM7;Q9BVA1;Q9H4B7	-0.0183	0.8507	0.1539	0.0404	TUBB;TUBB1;TUBB2A;TUBB2B;TUBB4B
P07437;Q13509;Q13885;Q9BVA1	0.0476	0.5692	0.0205	0.799	TUBB;TUBB2A;TUBB2B;TU
P07437;P68371;Q13509;Q13885;Q9BVA1	-0.0694	0.4612	-0.0648	0.4877	TUBB;TUBB2A;TUBB2B;TUBB3;TUBB4B
P04350;P07437;P68371;Q13509;Q13885;Q9BUF5;Q9BVA1	0.01	0.8787	0.0881	0.1123	TUBB;TUBB2A;TUBB2B;TUBB3;TUBB4B;TUBB6
P04350;P07437;P68371;Q13885;Q9BVA1	0.0455	0.6009	-0.1244	0.3958	TUBB;TUBB2A;TUBB2B;TUBB4B
A6NNZ2;P04350;P07437;P68371;Q13885;Q3ZCM7;Q9BUF5;Q9BVA1	-0.0291	0.8182	-0.017	0.8621	TUBB;TUBB2A;TUBB2B;TUBB4B;TUBB6
P07437;Q13509;Q13885;Q9BVA1	-0.1323	0.6418	-0.1671	0.48	TUBB;TUBB2A;TUBB3
P04350;P07437;P68371;Q13509;Q13885;Q9BUF5;Q9BVA1	0.2307	0.1504	0.2572	0.0966	TUBB;TUBB2A;TUBB3;TUBB4B;TUBB6
P04350;P07437;P68371;Q13509	0.1634	0.3006	0.1013	0.4435	TUBB;TUBB3;TUBB4B
P04350;P07437;P68371	0.0586	0.6145	0.0337	0.7481	TUBB;TUBB4B
Q13885	0.1402	0.3382	0.045	0.7203	TUBB2A
Q13885;Q9BVA1	0.1655	0.3347	-0.0023	0.9878	TUBB2A;TUBB2B
Q13885;Q9BUF5;Q9BVA1	0.2024	0.0925	0.1511	0.3891	TUBB2A;TUBB2B;TUBB6
Q13509	-0.1414	0.4559	-0.0102	0.9575	TUBB3
P04350;P68371;Q13509	0.0852	0.4035	-0.118	0.522	TUBB3;TUBB4B
Q13509;Q9BUF5	0.1061	0.3826	0.1771	0.0584	TUBB3;TUBB6
P04350;P68371	0.0055	0.9092	-0.0546	0.4784	TUBB4B
Q9BUF5	0.0456	0.6617	0.1924	0.0735	TUBB6
P23258	0.0625	0.6282	0.0006	0.9964	TUBG1
P49411	-0.0055	0.9358	-0.0566	0.7011	TUFM
Q12792	0.047	0.5985	0.0562	0.2974	TWF1
Q6IBS0	-0.0733	0.6501	-0.1142	0.3375	TWF2

P40222	0.0895	0.4136	-0.0794	0.6072	TXLNA
P10599	-0.0459	0.7451	-0.0599	0.4664	TXN
O95881	0.0265	0.788	-0.1173	0.2283	TXNDC12
Q9BRA2	-0.0878	0.2582	-0.0309	0.7288	TXNDC17
Q8NBS9	-0.1945	0.0825	-0.0565	0.3384	TXNDC5
O43396	0.0562	0.422	-0.0033	0.9666	TXNL1
Q16881	0.3381	0.0787	0.1273	0.443	TXNRD1
P19971	-0.2076	0.1596	-0.1102	0.4997	TYMP
Q01081	0.0909	0.4458	0.0481	0.5881	U2AF1
P26368	0.1025	0.1545	0.0112	0.8637	U2AF2
Q9BZF9	0.0373	0.8503	0.0225	0.8279	UACA
Q16222	-0.0471	0.6864	0.1643	0.0597	UAP1
Q16222;Q3KQV9	0.1828	0.373	0.0259	0.8416	UAP1;UAP1L1
Q3KQV9	0.04	0.8556	-0.1391	0.3265	UAP1L1
P22314	0.0918	0.2984	0.0268	0.7072	UBA1
Q9UBT2	0.0358	0.638	-0.0439	0.3895	UBA2
Q8TBC4	0.0769	0.43	0.0808	0.2864	UBA3
Q9GZZ9	0.0171	0.858	0.0179	0.7562	UBA5
A0AVT1	-0.0543	0.4782	0.0398	0.2202	UBA6
Q14157	-0.0168	0.8078	-0.0814	0.3199	UBAP2L
P63146	0.0666	0.7431	0.0289	0.7267	UBE2B
P51668;P61077;P62837;Q9Y2X8	-0.2199	0.5478	-0.6293	0.0733	UBE2D1;UBE2D2;UBE2D3
P51668;Q9Y2X8	0.0707	0.5561	-0.1138	0.3786	UBE2D1;UBE2D4
P61077;P62837	0.0621	0.4072	0.0638	0.5371	UBE2D2;UBE2D3
P51965;Q969T4;Q96LR5	-0.0909	0.1325	-0.0365	0.728	UBE2E1
P62256	-0.0766	0.5624	0.2256	0.0718	UBE2H
P63279	0.1272	0.2483	-0.0839	0.3928	UBE2I
P61086	-0.0033	0.9514	-0.0989	0.2918	UBE2K
P68036;A0A1B0GUS4	-0.0171	0.7138	0.0598	0.3463	UBE2L3
P61081	-0.0173	0.7278	-0.1048	0.1724	UBE2M
P61088;Q5JXB2	0.0524	0.2971	0	0.9997	UBE2N
Q13404	-0.021	0.8721	0.1509	0.2718	UBE2V1
Q13404;Q15819	0.0803	0.1841	0.0589	0.3281	UBE2V1;UBE2V2
Q15819	-0.0359	0.8047	0.0387	0.7018	UBE2V2
Q9H832	0.0951	0.2398	0.0284	0.7638	UBE2Z
O14562	-0.0642	0.6165	-0.183	0.0783	UBFD1
Q8WVY7	0.0567	0.6727	0.003	0.9682	UBLCP1
Q9UHD9	0.1	0.4618	0.3084	0.0875	UBQLN2
Q9NRR5;Q9UHD9;Q9UMX0	-0.1384	0.2644	0.06	0.7783	UBQLN2;UBQLN4
Q9NRR5;Q9UMX0	0.0651	0.5885	0.1286	0.3945	UBQLN4
Q5T4S7	0.0598	0.5523	0.1656	0.0639	UBR4
P17480	0.2025	0.2556	0.039	0.71	UBTF
Q04323	-0.0055	0.9625	0.0153	0.9026	UBXN1
Q92575	0.0904	0.6365	0.0428	0.8586	UBXN4
Q9BZV1	-0.0371	0.7569	0.1002	0.1242	UBXN6
P09936	-0.3917	0.533	0.155	0.808	UCHL1
P15374	-0.0419	0.6052	-0.0152	0.8771	UCHL3
Q92890	0.0342	0.6036	-0.0969	0.1606	UFD1

O94874	0.0352	0.7302	0.0543	0.4646	UFL1
P61960	-0.0762	0.3833	-0.1605	0.0285	UFM1
O60701	-0.2301	0.0129	-0.0088	0.9256	UGDH
Q9NYU2	-0.0689	0.2073	-0.03	0.5562	UGGT1
Q16851	-0.0242	0.7983	0.0998	0.3732	UGP2
P11172	-0.0753	0.7163	0.0059	0.9513	UMPS
Q9H3U1	-0.1607	0.072	-0.016	0.8855	UNC45A
Q92900	0.0509	0.4395	0.0218	0.4821	UPF1
P14927	-0.0708	0.6627	-0.28	0.0806	UQCRB
P31930	0.023	0.7351	0.0115	0.9285	UQCRC1
P22695	-0.0516	0.527	0.0163	0.8911	UQCRC2
P47985;P0C7P4	-0.1159	0.3279	-0.0779	0.6283	UQCRFS1
O14949	-0.0618	0.6909	-0.184	0.2073	UQCRQ
P06132	-0.102	0.5153	0.0343	0.861	UROD
O60763	-0.1054	0.0992	0.0589	0.3892	USO1
P54578	0.1078	0.0266	-0.035	0.5612	USP14
Q9Y4E8	0.0087	0.9149	0.0834	0.3828	USP15
Q9UPU5	0.0349	0.8175	0.0394	0.8237	USP24
Q96K76	0.2905	0.0156	0.0049	0.935	USP47
P45974	-0.0088	0.8388	0.0016	0.9655	USP5
Q93009	0.4097	0.0004	0.0251	0.7957	USP7
P40818	-0.1574	0.1999	-0.0931	0.3609	USP8
O00507;Q93008	-0.0489	0.3733	-0.0466	0.4899	USP9X
P46939	0.0388	0.6092	-0.0296	0.6321	UTRN
Q08AM6	0.2312	0.1729	-0.0062	0.9655	VAC14
P63027;Q15836	0.3191	0.1577	0.0698	0.6014	VAMP2;VAMP3
Q15836	0.1329	0.1766	0.1577	0.083	VAMP3
O95183	0.1696	0.3097	0.0227	0.8981	VAMP5
P51809	-0.0537	0.6572	-0.0103	0.8965	VAMP7
Q9P0L0	0.0773	0.2377	-0.0864	0.2798	VAPA
O95292	-0.083	0.2037	-0.0028	0.9721	VAPB
P26640	0.0028	0.9613	-0.032	0.5131	VARS1
Q6EMK4	0.0262	0.8654	0.0323	0.8213	VASN
P50552	0.0285	0.8785	0.1907	0.5141	VASP
Q99536	-0.1494	0.3345	-0.0569	0.6405	VAT1
P61758	-0.0675	0.3793	0.0719	0.3089	VBP1
P18206	0.0774	0.3857	0.0682	0.433	VCL
P55072	-0.0437	0.3259	0.0142	0.8058	VCP
P21796	0.0547	0.1616	-0.1242	0.2099	VDAC1
P45880	0.0208	0.6421	-0.0229	0.779	VDAC2
Q9Y277	-0.0267	0.7582	-0.0646	0.5863	VDAC3
P08670	-0.0277	0.811	-0.1206	0.416	VIM
Q9BQB6	-0.3003	0.1312	-0.0896	0.3974	VKORC1
Q8N0U8	0.0196	0.9041	0.3261	0.0722	VKORC1L1
Q3ZAQ7	0.0812	0.3608	0.123	0.0343	VMA21
Q709C8	-0.1167	0.2405	0.067	0.3114	VPS13C
Q9H269	-0.0504	0.6957	0.0097	0.9296	VPS16
O75436	-0.0179	0.7772	-0.0591	0.453	VPS26A

Q4G0F5	0.018	0.7822	0.0465	0.3406	VPS26B
Q9UBQ0	-0.1056	0.2569	-0.17	0.0679	VPS29
Q9H267	-0.207	0.2883	0.1121	0.3184	VPS33B
Q96QK1	-0.0665	0.1452	-0.1156	0.0169	VPS35
Q7Z3J2	0.1512	0.0328	-0.0739	0.3652	VPS35L
Q86VN1	0.1721	0.3094	-0.1045	0.4387	VPS36
Q8NEZ2	0.4288	0.1307	0.0556	0.6649	VPS37A
Q96JC1	0.0006	0.9963	-0.1695	0.2057	VPS39
Q9NRW7	-0.055	0.63	0.0134	0.9193	VPS45
Q9UN37	0.0569	0.6664	-0.0345	0.7952	VPS4A
O75351	-0.0452	0.6675	-0.0826	0.306	VPS4B
Q9NP79	-0.1007	0.2879	0.059	0.5535	VTA1
Q9UEU0	-0.0633	0.4472	-0.0481	0.6886	VTI1B
P23381	0.1995	0.48	0.1714	0.4401	WARS1
Q9UGM6	-0.0282	0.652	0.0362	0.6111	WARS2
A8K0Z3;C4AMC7;Q6VEQ5;A8MWX3;Q9NQA3	0.0619	0.6296	-0.032	0.7905	WASH2P;WASH3P
Q641Q2;Q9Y4E1	-0.1659	0.2467	-0.1327	0.356	WASHC2A
Q9Y3C0	0.1306	0.2482	0.2085	0.0391	WASHC3
Q2M389	0.1431	0.1721	-0.0998	0.3194	WASHC4
Q12768	-0.0651	0.4879	-0.0476	0.634	WASHC5
Q8IWB7	-0.1169	0.1202	0.0456	0.6151	WDFY1
O75083	-0.0312	0.8161	0.0668	0.5184	WDR1
Q9BV38	-0.0537	0.7221	-0.035	0.7544	WDR18
Q5JSH3	0.024	0.8102	0.0716	0.3229	WDR44
P61964	-0.0107	0.9603	0.0602	0.5442	WDR5
Q9Y4E6	-0.467	0.0874	-0.4806	0.0808	WDR7
Q9BQA1	0.094	0.3393	0.0732	0.4311	WDR77
A4D1P6	-0.1426	0.3092	-0.1126	0.366	WDR91
O43516	0.0591	0.512	-0.1823	0.047	WIPF1
Q5MNZ9	0.0527	0.7427	-0.2583	0.0768	WIPI1
Q9H4A3	0.0016	0.992	-0.063	0.7102	WNK1
Q9NQW7	0.0283	0.6194	0.1392	0.0235	XPNPEP1
O14980	0.046	0.3219	-0.0004	0.9925	XPO1
Q9UIA9	-0.0065	0.9636	0.0326	0.7562	XPO7
O43592	0.1568	0.2523	0.0852	0.6556	XPOT
P13010	0.0584	0.4526	-0.0701	0.2224	XRCC5
P12956	-0.0255	0.7617	-0.0725	0.1852	XRCC6
Q9H0D6	0.0904	0.4927	0.0072	0.9253	XRN2
P46937	0.0465	0.59	-0.0385	0.5895	YAP1
P54577	0.103	0.4971	0.1502	0.3985	YARS1
P67809	0.004	0.9757	0.1992	0.2179	YBX1
P16989;P67809;Q9Y2T7	-0.0243	0.8275	0.0722	0.5631	YBX1;YBX3
P16989	-0.0109	0.9427	-0.0432	0.6266	YBX3
O95070	-0.0709	0.6215	-0.0852	0.4235	YIF1A
Q5BJH7	0.0675	0.6309	0.1602	0.2451	YIF1B
Q969M3	0.0626	0.628	0.0765	0.633	YIPF5
O15498	-0.0271	0.8114	0.0368	0.8142	YKT6

P49750	0.0178	0.8941	-0.0717	0.6639	YLPM1
Q7Z739	0.04	0.8495	0.0762	0.5773	YTHDF3
P31946	0.063	0.4443	-0.0308	0.7018	YWHAB
P27348;P31946;P31947;P61981;P62258;P63104;Q04917	0.1684	0.0464	0.0004	0.9965	YWHAB;YWHAE;YWHAG;YWHAH;YWHAQ;YWHAZ
P27348;P31946;P63104	0.0346	0.331	0.039	0.4662	YWHAB;YWHAQ;YWHAZ
P62258	0.0099	0.7998	-0.0377	0.2742	YWHAE
P61981	-0.1193	0.0512	0.0421	0.4341	YWHAG
Q04917	0.0183	0.8355	0.1085	0.2622	YWHAH
P27348	0.0136	0.866	-0.0073	0.9377	YWHAQ
P63104	-0.0768	0.0762	-0.0295	0.5013	YWHAZ
Q7Z2W4	-0.0312	0.7009	0.0407	0.5618	ZC3HAV1
Q96KR1	-0.0032	0.9789	-0.0095	0.9202	ZFR
O43670	-0.066	0.7021	-0.1564	0.1014	ZNF207
Q14590;Q86WZ6	0.0408	0.638	-0.0487	0.5595	ZNF227
Q8TD17	0.1688	0.1591	-0.036	0.8442	ZNF398
Q8N1G0	0.1243	0.4446	0.1723	0.486	ZNF687
O95218	-0.066	0.4067	-0.0636	0.3779	ZRANB2
Q15942	-0.0212	0.8972	0.1065	0.6418	ZYX
